# Supplementary material for: Genetic diversity and regulatory features of human-specific NOTCH2NL duplications
Source: Cell Genom. 2026 Mar 30;6(6):101194. doi: 10.1016/j.xgen.2026.101194 (PMC13261667; doi:10.1016/j.xgen.2026.101194)
Supplement: Document S1. Figures S1–S13 and Tables S1–S5 [file mmc1.pdf]

Cell Genomics, Volume *6*

Supplemental information

Genetic diversity and regulatory features of human-specific *NOTCH2NL* duplications

Taylor D. Real, Prajna Hebbar, DongAhn Yoo, Francesca Antonacci, Ivana Pačar, Danilo Dubocanin, Mark Diekhans, Gregory J. Mikol, Oyeronke G. Popoola, Benjamin J. Mallory, Mitchell R. Vollger, Philip C. Dishuck, Xavi Guitart, Allison N. Rozanski, Katherine M. Munson, Kendra Hoekzema, Jane E. Ranchalis, Shane J. Neph, Adriana E. Sedeño-Cortés, Benedict Paten, Sofie R. Salama, Andrew B. Stergachis, and Evan E. Eichler

SUPPLEMENTAL MATERIAL

ADDITIONAL TABLES & FIGURES

Table S1. Structural variants of *NOTCH2NL* paralogs in T2T-CHM13, related to Figure 1C.

query paralog	ref paralog	query start	query end	mapq	structural variant size	in. (I) / del (D)
NOTCH2	NOTCH2NLC	119846347	119900735	60	54389	I
NOTCH2	NOTCH2NLC	119582683	119582682	60	737	D
NOTCH2	NOTCH2NLC	119541422	119547699	60	6278	I
NOTCH2	NOTCH2NLC	119577460	119579275	60	1816	I
NOTCH2	NOTCH2NLB	119542857	119555494	60	12638	I
NOTCH2	NOTCH2NLB	119814488	119818620	60	4133	I
NOTCH2	NOTCH2NLB	119461254	119461253	60	5629	D
NOTCH2	NOTCH2NLB	119461388	119461387	60	4741	D
NOTCH2	NOTCH2NLB	119462558	119462557	60	996	D
NOTCH2	NOTCH2NLB	119677199	119677198	60	748	D
NOTCH2	NOTCH2NLB	119857611	119857610	60	3875	D
NOTCH2	NOTCH2NLB	119917835	119917834	60	1358	D
NOTCH2	NOTCH2NLB	120135864	120137057	60	1194	I
NOTCH2	NOTCH2NLB	120134461	120135831	60	1371	I
NOTCH2	NOTCH2NLA	119814488	119818620	60	4133	I
NOTCH2	NOTCH2NLA	119677199	119677198	60	748	D
NOTCH2	NOTCH2NLA	119857611	119857610	60	3852	D
NOTCH2	NOTCH2NLA	119917825	119917824	60	1360	D
NOTCH2	NOTCH2NLA	119542836	119555473	60	12638	I
NOTCH2	NOTCH2NLA	119462604	119462603	60	996	D
NOTCH2	NOTCH2NLA	119461389	119461388	60	4741	D
NOTCH2	NOTCH2NLA	119461253	119461252	60	5629	D
NOTCH2	NOTCH2NLA	120134365	120134947	31	583	I
NOTCH2	NOTCH2NLA	120135063	120135832	31	770	I
NOTCH2	NOTCH2NLA	120135863	120137056	31	1194	I
NOTCH2NLA	NOTCH2NLC	145263860	145265217	60	1358	I
NOTCH2NLA	NOTCH2NLC	145426571	145428098	60	1528	I
NOTCH2NLA	NOTCH2NLC	145431274	145434427	60	3154	I
NOTCH2NLA	NOTCH2NLC	144857035	144858873	60	1839	I
NOTCH2NLA	NOTCH2NLB	145373282	145374823	60	1542	I
NOTCH2NLA	NOTCH2NLB	145426775	145428316	60	1542	I
NOTCH2NLA	NOTCH2NLB	145357941	145357940	60	6296	D
NOTCH2NLA	NOTCH2NLB	145452444	145455266	60	2823	I
NOTCH2NLB	NOTCH2NLC	146024204	146083108	60	58905	I
NOTCH2NLB	NOTCH2NLC	146100183	146101538	60	1356	I
NOTCH2NLB	NOTCH2NLC	146196656	146202946	60	6291	I
NOTCH2NLB	NOTCH2NLC	146270637	146273780	60	3144	I
NOTCH2NLB	NOTCH2NLC	146282918	146295060	60	12143	I
NOTCH2NLB	NOTCH2NLC	146295069	146303567	60	8499	I
NOTCH2NLB	NOTCH2NLC	146215843	146215842	60	1549	D
NOTCH2NLB	NOTCH2NLC	146461653	146461652	60	2066	D
NOTCH2NLB	NOTCH2NLC	145759226	145761064	60	1839	I
NOTCH2NLC	NOTCH2NLR	148580984	148584007	60	3024	I
NOTCH2NLC	NOTCH2NLR	148623116	148626328	60	3213	I
NOTCH2NLC	NOTCH2NLR	148627877	148631089	60	3213	I
NOTCH2NLC	NOTCH2NLR	148635700	148678456	60	42757	I
NOTCH2NLC	NOTCH2NLR	148608665	148608664	60	6274	D
NOTCH2NLC	NOTCH2NLR	148687503	148687502	60	11467	D
NOTCH2NLC	NOTCH2NLR	148687511	148687510	60	8199	D
NOTCH2NLC	NOTCH2NLR	148867048	148870884	60	3837	I
NOTCH2NLC	NOTCH2NLR	148443146	148443145	60	11467	D
NOTCH2NLC	NOTCH2NLR	148443154	148443153	60	8199	D
NOTCH2NLC	NOTCH2NLR	148335672	148335671	60	1816	D
NOTCH2NLR	NOTCH2NLB	120452239	120464876	60	12638	I

NOTCH2NLR	NOTCH2NLB	120546480	120546479	60	5629	D
NOTCH2NLR	NOTCH2NLB	120546346	120546345	60	4741	D
NOTCH2NLR	NOTCH2NLB	120545176	120545175	60	996	D
NOTCH2NLR	NOTCH2NLB	120330535	120330534	60	748	D
NOTCH2NLR	NOTCH2NLB	120730144	120730143	60	1356	D
NOTCH2NLR	NOTCH2NLB	120798010	120798009	60	3025	D
NOTCH2NLR	NOTCH2NLB	120841724	120841723	60	3184	D
NOTCH2NLR	NOTCH2NLB	120843404	120843403	60	3172	D
NOTCH2NLR	NOTCH2NLB	120845053	120845052	60	3202	D
NOTCH2NLR	NOTCH2NLB	120849542	120849541	60	40814	D
NOTCH2NLR	NOTCH2NLB	120861687	120861686	60	654	D
NOTCH2NLR	NOTCH2NLB	120594342	120594341	60	748	D
NOTCH2NLR	NOTCH2NLA	120452260	120464897	60	12638	I
NOTCH2NLR	NOTCH2NLA	120545130	120545129	60	996	D
NOTCH2NLR	NOTCH2NLA	120546345	120546344	60	4741	D
NOTCH2NLR	NOTCH2NLA	120546481	120546480	60	5629	D
NOTCH2NLR	NOTCH2NLA	120594342	120594341	60	748	D
NOTCH2NLR	NOTCH2NLA	120820315	120826597	60	6283	I
NOTCH2NLR	NOTCH2NLA	120730144	120730143	60	1358	D
NOTCH2NLR	NOTCH2NLA	120798010	120798009	60	3025	D
NOTCH2NLR	NOTCH2NLA	120843404	120843403	60	3180	D
NOTCH2NLR	NOTCH2NLA	120844944	120844943	60	3168	D
NOTCH2NLR	NOTCH2NLA	120330535	120330534	60	748	D
NOTCH2NLR	NOTCH2	120798010	120798009	60	3028	D
NOTCH2NLR	NOTCH2	120838292	120846100	60	7809	I

Table S2. Additional structural variation between human and NHA, related to Figure 2.

Variant information				NOTCH2 mapping (primary hap)		INV BP mapping (T2T-space; kbp)		
Species	Variants	Count	Bases (kbp)	Count	Bases (kbp)	Count	Bases (kbp)	p-value
PPY	PAV-DEL	816	752.8	3	0.7	71	99.5	1.000
PPY	PAV-INS	829	1025.1	6	9.1	82	140.0	1.000
PPY	Syri/PAV-INV	6	930.4	-	-	-	-	-
PPY	Sedef-SD (h1/h2)	98/97	2588.9/2588.9	1	10.6	-	-	-
PAB	PAV-DEL	810	751.7	3	0.7	72	106.1	1.000
PAB	PAV-INS	812	1016.1	5	9.0	74	123.7	1.000
PAB	Syri/PAV-INV	4	1338.3	-	-	-	-	-
PAB	Sedef-SD (h1/h2)	97/95	2611.4/2576.7	1	10.3	-	-	-
PTR	PAV-DEL	343	341.3	3	5.2	29	69.3	0.987
PTR	PAV-INS	312	1146.2	2	604.9	40	883.4	**<0.001***
PTR	Syri/PAV-INV	3	8017.1	-	-	-	-	-
PTR	Sedef-SD (h1/h2)	109/111	6565.9/6582.8	10	3660.8	-	-	-
GGO	PAV-DEL	475	591.1	5	11.1	36	141.7	0.832
GGO	PAV-INS	422	1010.9	0	0.0	35	223.8	**0.034***
GGO	Syri/PAV-INV	6	8885.6	-	-	-	-	-
GGO	Sedef-SD (pri/alt)	114/118	7103.1/7049.3	6	3043.9	-	-	-
PPA	PAV-DEL	353	359.9	3	5.2	19	51.0	0.999
PPA	PAV-INS	308	537.7	1	3.0	25	262.5	**<0.001***
PPA	Syri/PAV-INV	6	9691.5	-	-	-	-	-
PPA	Sedef-SD (pri/alt)	110/111	6459.5/6059.9	10	3532.2	-	-	-
HSA	Sedef-SD	91	8315.6	3	3988.8	30	1918.8	**<0.001***
			**Average**	3.9	931.0	46.6	365.4	

Table S3. NHA *NOTCH2NL* FLNC characterization, related to Figure 3.

Name	Iso-Seq transcript	Chr	Start	End	Strand	# of exons	Predicted AA Lengths	Long-read transcript support (NEC & testis)	Intergenic distance N2NL-NBPF (bp)	Species	Comments
PTR-NOTCH2	NOTCH2	chr1	117131242	117288384	-	--	2432	--	--	PTR/PPA/GGO/HSA	--
PTR-NOTCH2NL-1	NOTCH2NLR	chr1	106135723	106156373	+	4	246	26	11,622	PPA/PTR	ORF has exons 1, 3-5 of NOTCH2NLR
	Fusion NOTCH2NL-NBPF	chr1	106135792	106185074		20	851	69			ORF has exons 1, 3, 4 of NOTCH2NLR & NBPF exons
PTR-NOTCH2NL-2	Fusion MAGI3-NOTCH2NL	chr1	106365562	106516651	+	4	324	6	11,398	PPA/PTR	ORF has MAGI3 exon 1 & exons 3-5 of NOTCH2NLR
PTR-NOTCH2NL-3	NOTCH2NLR	chr1	108282469	108322382	+	4	235	16	11,838	PPA/PTR/GGO	ORF has exons 2-5 of NOTCH2NLR
	Fusion NOTCH2NL-NBPF	chr1	108282319	108349249		13	672	11			ORF has exons 2, 3, 4 of NOTCH2NLR & NBPF exons
	Fusion PDE4DIP-NOTCH2NL-NBPF	chr1	108214852	108349197		--	--	53			No ORF goes through the whole transcript
PTR-NOTCH2NL-4	Fusion MAGI3-NOTCH2NL	chr1	109104609	109227579	-	13	845	12	11,410	PPA/PTR	ORF has exon 1 of NOTCH2NLR, 9 MAGI3, and 3-5 exons of NOTCH2NLR
	Fusion MAGI3-NOTCH2NL-NBPF	chr1	109078294	109227828		17	1,411	53			ORF has exon 1 of NOTCH2NLR, 9 MAGI3, NOTCH2NL exons 3, 4, and NBPF exons
PTR-NOTCH2NL-5	NOTCH2NLR	chr1	109873983	109910441	-	3	146	9	11,295	PPA/PTR	ORF has exons 2, 3 of NOTCH2NLR & partial exon 4 (TRUNCATION) - transcript goes through until NOTCH2NLR-like exon 5
	Fusion NOTCH2NL-NBPF	chr1	109846910	109910766		3	146	12			ORF has exons 2, 3 of NOTCH2NLR & partial exon 4 (TRUNCATION) - transcript goes through to NBPF exons
	Fusion TXNIP-NOTCH2NL-NBPF	chr1	109846504	109953395		4	230	62			ORF has exon 1 of TXNIP, exons 2,3 of NOTCH2NLR, and partial exon 4 (TRUNCATION) - transcript goes through until NOTCH2NLR-like exon 5
PTR-NOTCH2NL-6	NOTCH2NLR	chr1	110315936	110336372	+	4	243	11	11,504	PPA/PTR/GGO	ORF has exon 1, 3-5 of NOTCH2NLR
	Fusion NOTCH2NL-NBPF	chr1	110313408	110368080		20	856	22			ORF that has exons 1, 3, 4 of NOTCH2NLR & NBPF exons
	Fusion SORT1-LRIG2-NOTCH2NL-NBPF	chr1	110225344	110368328		--	--	14			No ORF goes through the whole transcript
PTR-NOTCH2NL-7	Fusion with MAGI3	chr1	117544406	117696496	-	4	324	7	11,693	PPA/PTR	ORF has exon 1 of MAGI3 & exons 3-5 of NOTCH2NLR
PTR-NOTCH2NL-8	NOTCH2NLR	chr1	118044677	118065257	-	4	246	9	11,295	PTR	ORF has exons 1, 3-5 of NOTCH2NLR
	Fusion NOTCH2NL-NBPF	chr1	117960416	118065110		58	3033	16			ORF has exons 1, 3, 4 of NOTCH2NLR & NBPF exons
	Fusion LRIG2-NOTCH2NL	chr1	118044029	118124464		--	--	5			No ORF goes through the whole transcript
	Fusion LRIG2-NOTCH2NL-NBPF	chr1	117960416	118125899		--	--	4			No ORF goes through the whole transcript
PTR-NOTCH2NL-9	NOTCH2NLR	chr1	121665898	121686392	+	4	245	11	11,486	PPA/PTR	ORF has exons 1, 3-5 of NOTCH2NLR
	Fusion NOTCH2NL-NBPF	chr1	121665622	121801595		88	4694	48			ORF has exons 1, 3, 4 of NOTCH2NLR & NBPF exons
PPA-NOTCH2	NOTCH2	chr1	112555608	112716322	-	--	2610	--	--	PTR/PPA/GGO/HSA	--
PPA-NOTCH2NL-1	NOTCH2NLR	chr1	101669793	101703165	-	4	235	46	11,607	PPA/PTR/GGO	ORF has exons 2-5 of NOTCH2NLR human
	Fusion NOTCH2NL-NBPF	chr1	101642931	101703233		17	656	132			ORF has exons 2, 3, 4 of NOTCH2NLR & NBPF exons
	Fusion PDE4DIP-NOTCH2NL-NBPF	chr1	101641810	101777882		--	--	155			No ORF goes through the whole transcript.
PPA-NOTCH2NL-2	Fusion MAGI3-NOTCH2NL	chr1	103485748	103636324	-	4	237	6	11,837	PPA/PTR	ORF has exon 1 of MAGI3 & exons 3-5 of NOTCH2NLR
PPA-NOTCH2NL-3	NOTCH2NLR	chr1	103841187	103861631	+	4	246	11	11,672	PPA/PTR	ORF has exons 1, 3-5 of NOTCH2NLR
	Fusion NOTCH2NL-NBPF	chr1	103815726	103861620		15	757	98			ORF has exons 1, 3, 4 of NOTCH2NLR & NBPF exons
PPA-NOTCH2NL-4	Fusion MAGI3-NOTCH2NL	chr1	104697226	104820526		13	845	201	--	PPA/PTR	ORF has exon 1 of NOTCH2NLR, 9 MAGI3, & 3-5 exons of NOTCH2NLR
PPA-NOTCH2NL-5	NOTCH2NLR	chr1	105315877	105349528	+	4	235	11	11,690	PPA/PTR	ORF has exons 2-5 of NOTCH2NLR
	Fusion NOTCH2NL-NBPF	chr1	105288382	105349479		15	743	69			ORF has exons 2, 3, 4 of NOTCH2NLR & NBPF exons
	Fusion TXNIP-NOTCH2NL-NBPF	chr1	105286533	105389706		5	319	21			ORF has exon 1 of TXNIP & exons 2, 3-5 of NOTCH2NLR
PPA-NOTCH2NL-6	NOTCH2NLR	chr1	105752206	105772648	-	4	246	7	11,773	PPA/PTR	ORF has exons 1, 3-5 of NOTCH2NLR
	Fusion NOTCH2NL-NBPF	chr1	105752087	105795054		14	682	81			ORF has exons 1, 3, 4 of NOTCH2NLR & NBPF exons
PPA-NOTCH2NL-7	NOTCH2NLR	chr1	112968284	112977175	-	4	246	0	11,194	PPA	ORF has exons 3-5 of NOTCH2NLR
PPA-NOTCH2NL-8	Fusion MAGI3-NOTCH2NL	chr1	113432163	113583978	-	4	324	12	11,233	PPA/PTR	ORF has exon 1 of MAGI3 & exons 3-5 of NOTCH2NLR
PPA-NOTCH2NL-9	NOTCH2NLR	chr1	113892494	113913027	-	4	246	56	11,785	PPA	ORF has exons 1, 3-5 of NOTCH2NLR
PPA-NOTCH2NL-10	NOTCH2NLR	chr1	117521012	117542196	+	4	246	12	11,731	PPA/PTR/GGO	ORF has exons 1, 3-5 of NOTCH2NLR
	Fusion NOTCH2NL-NBPF	chr1	117520465	117596479		50	2399	35			ORF has exons 1, 3, 4 of NOTCH2NLR & NBPF exons

	Fusion SORT1-LRIG2-NOTCH2NL-NBPF	chr1 117431222 117596326		--	--	4			No ORF goes through the whole transcript
GGO-NOTCH2	NOTCH2	chr1 124746752 124900741	-	--	2611	--	--	PTR/PPA/GGO/HSA	--
GGO-NOTCH2NL-1	NOTCH2NLR	chr1 116765261 116796179	+	--	--	2	11,618	PPA/PTR/GGO	Has exon 3-5 of NOTCH2NLR but no exon 1/2 and no M start
	Fusion NOTCH2NL-NBPF	chr1 116765192 116847395		--	--	13			Has exon 3-4 of NOTCH2NLR & NBPF exons but no exon 1/2 & no M start
	Fusion SORT1-LRIG2-NOTCH2NL-NBPF	chr1 116693440 116846869		--	--	6			No ORF goes through the whole transcript.
GGO-NOTCH2NL-2	NOTCH2NLR	chr1 117331132 117364496	-	4	235	5	11,825	PPA/PTR/GGO	ORF has exons 2-5 of NOTCH2NLR
	Fusion NOTCH2NL-NBPF	chr1 117294017 117373312		24	1114	11			ORF has exons 2-4 of NOTCH2NLR & NBPF exons
	Fusion PDE4DIP-NOTCH2NL-NBPF	chr1 117294017 117447550		--	--	16			No ORF goes through the whole transcript.
GGO-NOTCH2NL-3	Fusion LRIG2-NOTCH2NL-NBPF	chr1 125591450 125661487	-	--	1662	5	5385	GGO	Has exons of LRIG2, exon 3 of NOTCH2NLR, & NBPF exons but no M start
	Fusion NOTCH2NL-NBPF	chr1 125591876 125635112		--	--	8			Has exon 3 of NOTCH2NLR and NBPF exons but no exon 1/2 and no M start
GGO-NOTCH2NL-4	Fusion LRIG2-NOTCH2NL-NBPF	chr1 129364798 129435830	+	--	--	19	5,608	GGO	Has 2 LRIG2 exons, exon 3 of NOTCH2NLR, & NBPF exons but early stop in exon 2
	Fusion NOTCH2NL-NBPF	chr1 129389184 129435600		--	--	2			Has 3-4 of NOTCH2NLR & NBPF exons but no exon 1/2 and no M start
GGO-NOTCH2NL-5	Fusion MAGI3-NOTCH2NL-NBPF	chr1 129726316 129995372	+	--	1487	2	5,588	GGO	Has exons of MAGI3, exon 3 of NOTCH2NLR, & then NBPF exons but no M start
	Fusion NOTCH2NL-NBPF	chr1 129964606 129995476		--	--	6			Has 3-4 of NOTCH2NLR & NBPF exons but no exon 1/2 and no M start
GGO-NOTCH2NL-6	Fusion NOTCH2NL-NBPF	chr1 130013152 130080198	-	13	783	72	39,879	GGO	ORF has exon 1 of NOTCH2NLR & NBPF exons
GGO-NOTCH2NL-7	NOTCH2NLR	chr1 130265287 130274296	-	--	--	14	11,779	GGO	Has 3-5 of NOTCH2NLR but no exon 1/2 and no M start
	Fusion NOTCH2NL-NBPF	chr1 130222211 130280603		--	--	15			Has 3-4 of NOTCH2NLR & NBPF exons but no exon 1/2 and no M start
	Fusion BRD9-NOTCH2NL	chr1 130264240 130305169		--	--	8			No ORF that goes through the whole transcript.
	Fusion BRD9-NOTCH2NL-NBPF	chr1 130221951 130304952		--	--	4			No ORF that goes through the whole transcript.
HSA-NOTCH2	NOTCH2	chr1 119924936 120082923	-	--	2471	--	--	PTR/PPA/GGO/HSA	--
HSA-NOTCH2NLA	NOTCH2NLA	chr1 145272197 145345902	-	5	236	--	11,336	HSA	
HSA-NOTCH2NLB	NOTCH2NLB	chr1 146108509 146181500	+	5	249	--	11,375	HSA	
HSA-NOTCH2NLC	NOTCH2NLC	chr1 148535272 148596912	+	5	236	--	11,726	HSA	
HSA-NOTCH2NLR	NOTCH2NLR	chr1 120737165 120807117	+	5	274	--	11,635	HSA	
	[a]Fusion NOTCH2NL-NBPF	chr1 120737168 120851627		36	1673	--			

[a]Fusion exists in all human *NOTCH2NL* but only *NOTCH2NLR* fusion is used for comparison.

Table S4. Population origin of HPRC[a] haplotypes resolved[b] across the *NOTCH2NL* locus, related to Figures 4 and 5.

superpopulation	superpopulation total	population	population total
AFR	34	African Caribbean in Barbados	11
		Gambian in Western Divisions - Mandinka	15
		Mende in Sierra Leone	6
		Yoruba in Ibadan, Nigeria	2
		African Ancestry in Southwest USA	2
		Maasai in Kinyawa, Kenya	1
AMR	23	Puerto Rican in Puerto Rico	14
		Colombian in Medellin, Colombia	2
		Peruvian in Lima, Peru	7
EAS	7	Kinh in Ho Chi Minh City, Vietnam	1
		Southern Han Chinese in Hu Nan Province, China	5
		Chinese Ancestry in USA	1
EUR	1	Ashkenazim Jewish	1
SAS	1	Punjabi in Lahore, Pakistan	1

[a]HPRC: Human Pangenome Reference Consortium

[b]This includes haplotypes that are not completely assembled across the centromere but have no gaps or collapses between *NOTCH2* and *NOTCH2NLR*, and *NOTCH2NLA/B/C*, respectively.

Table S5. *NOTCH2NL/GFP* gBlocks for measuring protein expression, related to STAR Methods.

Sequence name	5' – sequence – 3'
HA-NOTCH2tv-IRES-NheI-FseI gBlock	GGTCTAGAGCTAGCGAATTCGCCGGTGCCACCATGTACCCATACGATGTTCCAGA<br>TTACGCTCCCGCCCTGCGCCCCGCTCTGCTGTGGGCGCTGCTGGCGCTCTGGCT<br>GTGCTGCGCGGCCCCCGCGCATGCATTGCAGTGTCGAGATGGCTATGAACCCTG<br>TGTAAATGAAGGAATGTGTGTTACCTACCACAATGGCACAGGATACTGCAAATGTC<br>CAGAAGGCTTCTTGGGGGAATATTGTCAACATCGAGACCCCTGTGAGAAGAACCG<br>CTGCCAGAATGGTGGGACTTGTGTGGCCCAGGCCATGCTGGGGAAAGCCACGTG<br>CCGATGTGCCTCAGGGTTTACAGGAGAGGACTGCCAGTACTCAACATCTCATCCA<br>TGCTTTGTGTCTCGACCCTGCCTGAATGGCGGCACATGCCATATGCTCAGCCGGG<br>ATACCTATGAGTGCACCTGTCAAGTCGGGTTTACAGGTAAGGAGTGCCAATGGAC<br>GGATGCCTGCCTGTCTCATCCCTGTGCAAATGGAAGTACCTGTACCACTGTGGCC<br>AACCAGTTCTCCTGCAAATGCCTCACAGGCTTCACAGGGCAGAAATGTGAGACTG<br>ATGTCAATGAGTGTGACATTCCAGGACACTGCCAGCATGGTGGAACCTGCCTCAA<br>CCTGCCTGGTTCCTACCAGTGCCAGTGCCCTCAGGGCTTCACAGGCCAGTACTGT<br>GACAGCCTGTATGTGCCCTGTGCACCCTCACCTTGTGTCAATGGAGGTACCTGTC<br>GGCAGACTGGTGACTTCACTTTTGAGTGCAACTGCCTTCCAGAAACAGTGAGAAAT<br>AAGAGGAACAGAGCTCTGGGAAAGGGACAGGCAAGTCTGGAATGGAAAAGAACA<br>TGAGTCGACCGCTTGGAATAAGGCCGGTGTGCGTTTGTCTATATGTTATTTTCCAC<br>CATATTGCCGTCTTTTGGCAATGTGAGGGCCCGGAAACCTGGCCCTGTCTTCTTG<br>ACGAGCATTCCTAGGGGTCTTTCCCCTCTCGCCAAAGGAATGCAAGGTCTGTTGA<br>ATGTCGTGAAGGAAGCAGTTCCTCTGGAAGCTTCTTGAAGACAAACAACGTCTGTA<br>GCGACCCTTTGCAGGCAGCGGAACCCCCCACCTGGCGACAGGTGCCTCTGCGGC<br>CAAAAGCCACGTGTATAAGATACACCTGCAAAGGCGGCACAACCCCAGTGCCACG<br>TTGTGAGTTGGATAGTTGTGGAAAGAGTCAAATGGCTCTCCTCAAGCGTATTCAAC<br>AAGGGGCTGAAGGATGCCCAGAAGGTACCCCATTGTATGGGATCTGATCTGGGG<br>CCTCGGTGCACATGCTTTACATGTGTTTAGTCGAGGTTAAAAAAACGTCTAGGCCC<br>CCCGAACCACGGGGACGTGGTTTTCCTTTGAAAAACACGATGATAAGATCTGCGA<br>TCTAAGTAAGCTTGGCATTCCGGTACTGTTGGTAAAGCCACCATGGAATCCGGCC<br>GGCCCGAATTCGGC
HA-NOTCH2NLB pEF1A Gibson gBlock	GATGTTCCAGATTACGCTTGTCGAGATGGCTATGAACCCTGTGTAAATGAAGGAAT<br>GTGTGTTACCTACCACAATGGCACAGGATACTGCAAATGTCCAGAAGGCTTCTTG<br>GGGGAATATTGTCAACATCGAGACCCCTGTGAGAAGAACCGCTGCCAGAATGGTG<br>GGACTTGTGTGGCCCAGGCCATGCTGGGGAAAGCCACGTGCCGATGTGCCTCAG<br>GGTTTACAGGAGAGGACTGCCAGTACTCGACATCTCATCCATGCTTTGTGTCTCGA<br>CCTTGCCTGAATGGCGGCACATGCCATATGCTCAGCCGGGATACCTATGAGTGCA<br>CCTGTCAGGTCGGGTTTACAGGTAAGGAGTGCCAATGGACCGATGCCTGCCTGTC<br>TCATCCCTGTGCAAATGGAAGTACCTGTACCACTGTGGCCAACCAGTTCTCCTGCA<br>AATGCCTCACAGGCTTCACAGGGCAGAAGTGTGAGACTGATGTCAATGAGTGTGA<br>CATTCCAGGACACTGCCAGCATGGTGGCATCTGCCTCAACCTGCCTGGTTCCTAC<br>CAGTGCCAGTGCCTTCAGGGCTTCACAGGCCAGTACTGTGACAGCCTGTATGTGC<br>CCTGTGCACCCTCGCCTTGTGTCAATGGAGGCACCTGTCGGCAGACTGGTGACTT<br>CACTTTTGAGTGCAACTGCCTTCCAGAAACAGTGAGAAGAGGAACAGAGCTCTGG<br>GAAAGAGACAGGGAAGTCTGGAATGGAAAAGAACACGATGAGAATTAGGTCGACC<br>GCTTGGAATAA
HA-NOTCH2NLR pEF1A Gibson gBlock	GATGTTCCAGATTACGCTCCCGCCCTGCGTCCCGCTCTGCTGTGGGCGCTGCTG<br>GCGCTCTGGCTGTGCTGGGCGGCCCCCGCGCATGCATTGCAGTGTCGAGATGGC<br>TATGAACCCTGTGTAAATAAAGGAATGTGTGTTACCTACCACAGTGGCACAGGATA<br>CTGCAAATGTCCAGAAGGCTTCTTGGGGGAATATTGTCAACATCGAGACCCCTGT<br>GAGAAGAACCGCTGCCAGAATGGTGGGACTTGTGTGGCCCAGGCCATGCTGGGG<br>AAAGCCACGTGCCGGTGTGCCTCAGGGTTTACAGGAGAGGACTGCCAGTACTCG<br>ACACCTCATCCATGCTTTGTGTCTCGACCTTGCCTGAATGGCGGCACATGCCATAT<br>GCTCAGCCGGGATACCTATGAGTGCACCTGTCAAGTCGGGTTTACAGGTAAGGAG<br>TGCCAATGGACCGATGCCTGCCTGTCTCATCTCTGTGCAAATGGAAGTACCTGTA<br>CCACTGTGGCCAACCAGTTCTCCTGCAAATGCCTCACAGGCTTCACAGGGCAGAA<br>GTGTGAGACTGATGTCAATGAGTGTGACATTCCAGGACACTGCCAGCATGGTGGC<br>ACCTGCCTCAACCTGCCTGGTTCCTACCAGTGCCAGTGCCTTCAGGGCTTCACAG<br>GCCAGTACTGTGACAGACTGTATGTGCCCTGTGCACACTCGCCTTGTGTCAATGG<br>AGGCACCTGTCGGCAGACTGGTGACTTCACTTTTGAGTGCAACTGCCTTCCAGAA<br>ACAGTGAGAAATAAGAGGAACAGAGCTCTGGGAAAGAGACAGGCAAGTCTGGAAT<br>GGAAAAGAACACGATGAGTCGACCGCTTGGAATAA

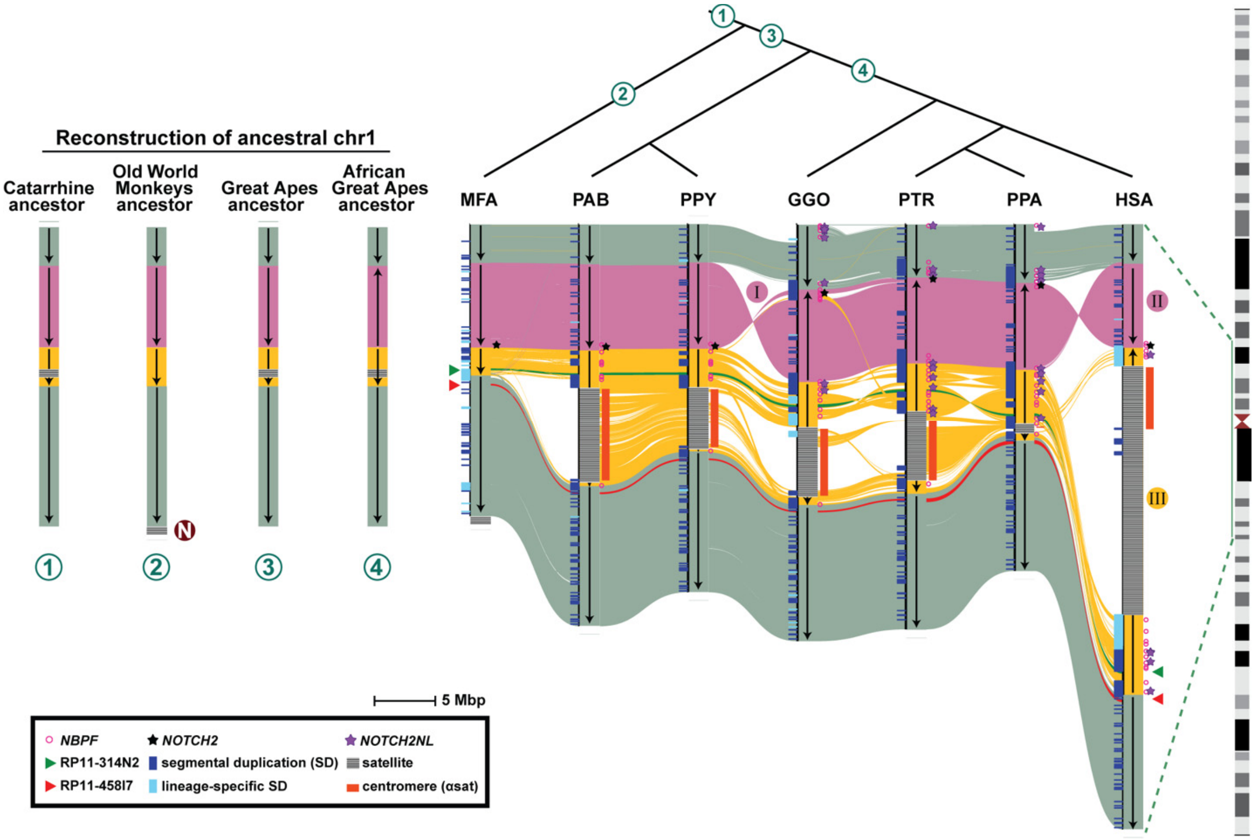

Figure S1. Ape evolutionary rearrangement and expansion of human chromosome 1p21.2-q23.2, related to Figure 2. The genomic structure of chromosome 1p21.2-q23.2 region is compared among macaque (MFA), Sumatran orangutan (PAB), Bornean orangutan (PPY), gorilla (GGO), chimpanzee (PTR), bonobo (PPA), and human (HSA) with annotations that include ancestral *NOTCH2* (black stars), *NOTCH2NL* duplications (purple stars), *NBPF* duplications (pink circles), and the centromere (orange bars). The circled numbers represent previous ancestral states of chromosome 1. The circled N represents a centromere repositioning event (N, neocentromere). Three distinct evolutionary inversions are predicted (I, II, III). Two probes (RP11-314N2, green, and RP11-458I7, red) used in FISH analyses from Szamalek et al. (2006)[S1] are shown (green and red triangles). Both probes map to the q-arm in humans, with the green probe located inside the inverted region and the red probe outside. FISH data from Szamalek et al. (2006)[S1] revealed that in chimpanzee the green probe maps to the region homologous to the human p-arm, while the red probe maps to the q-arm. Sequence analysis supports the FISH mapping and shows that in great apes the sequence of the two probes (represented as red and green lines in the SVbyEye) map on opposite sides of the centromere.

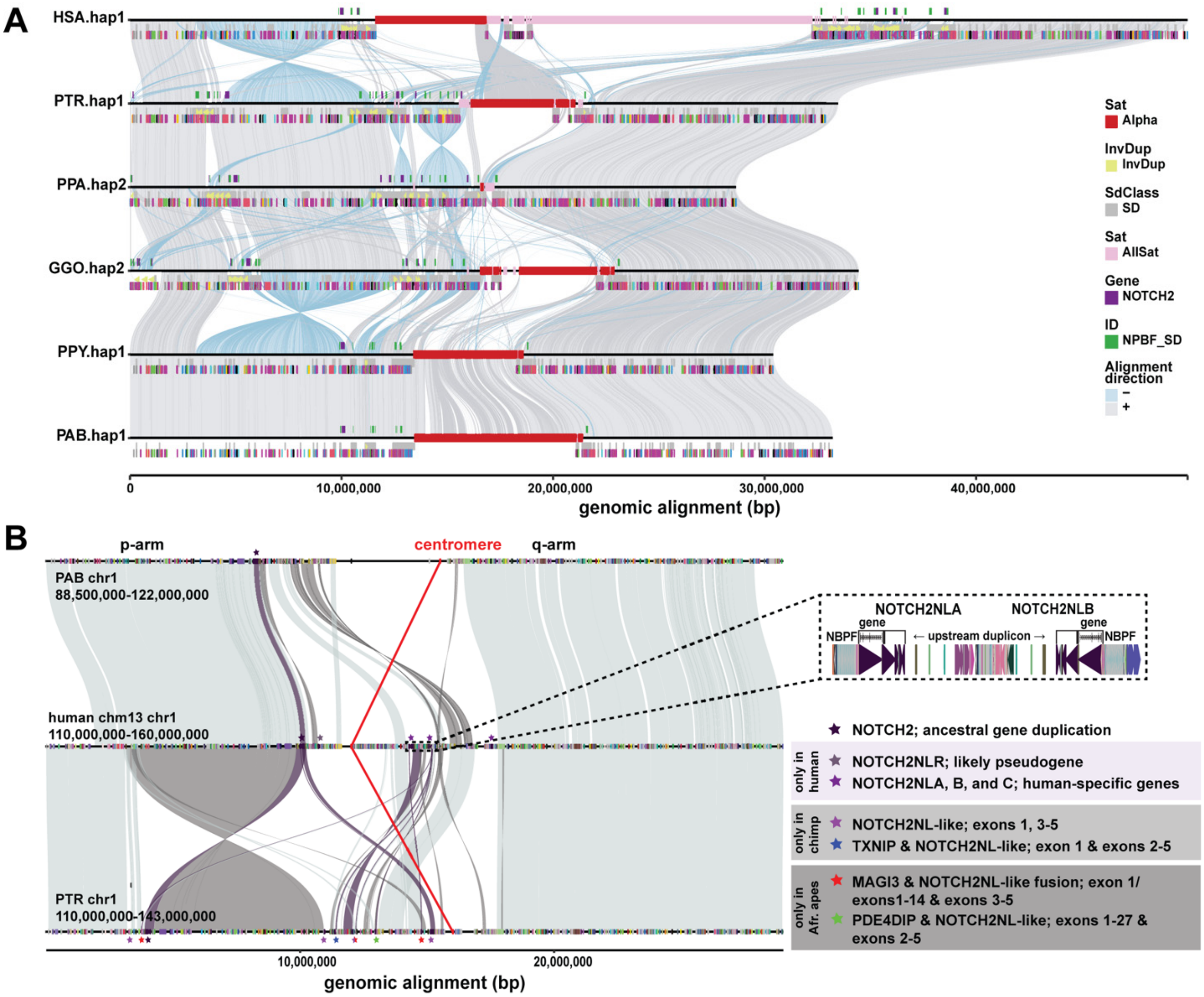

Figure S2. NHP and human chromosome 1 evolutionary rearrangements, related to Figure 2.
A) Stacked SVbyEye plot of human chromosome 1 compared to syntenic chromosomes in other apes over 30-50 Mbp of the pericentromeric region. Directly aligned sequence (gray) is compared to inversion rearrangements (light blue) with various annotations including *NOTCH2NL* and *NBPF* genes, satellites, and others. **B)** A zoomed-in comparison including only chimpanzee, orangutan, and human where centromeric sequence has been masked to highlight the non-orthologous location of NHA *NOTCH2NL* transcripts (colored stars) as described by Fiddes et al. (2018)[S2].

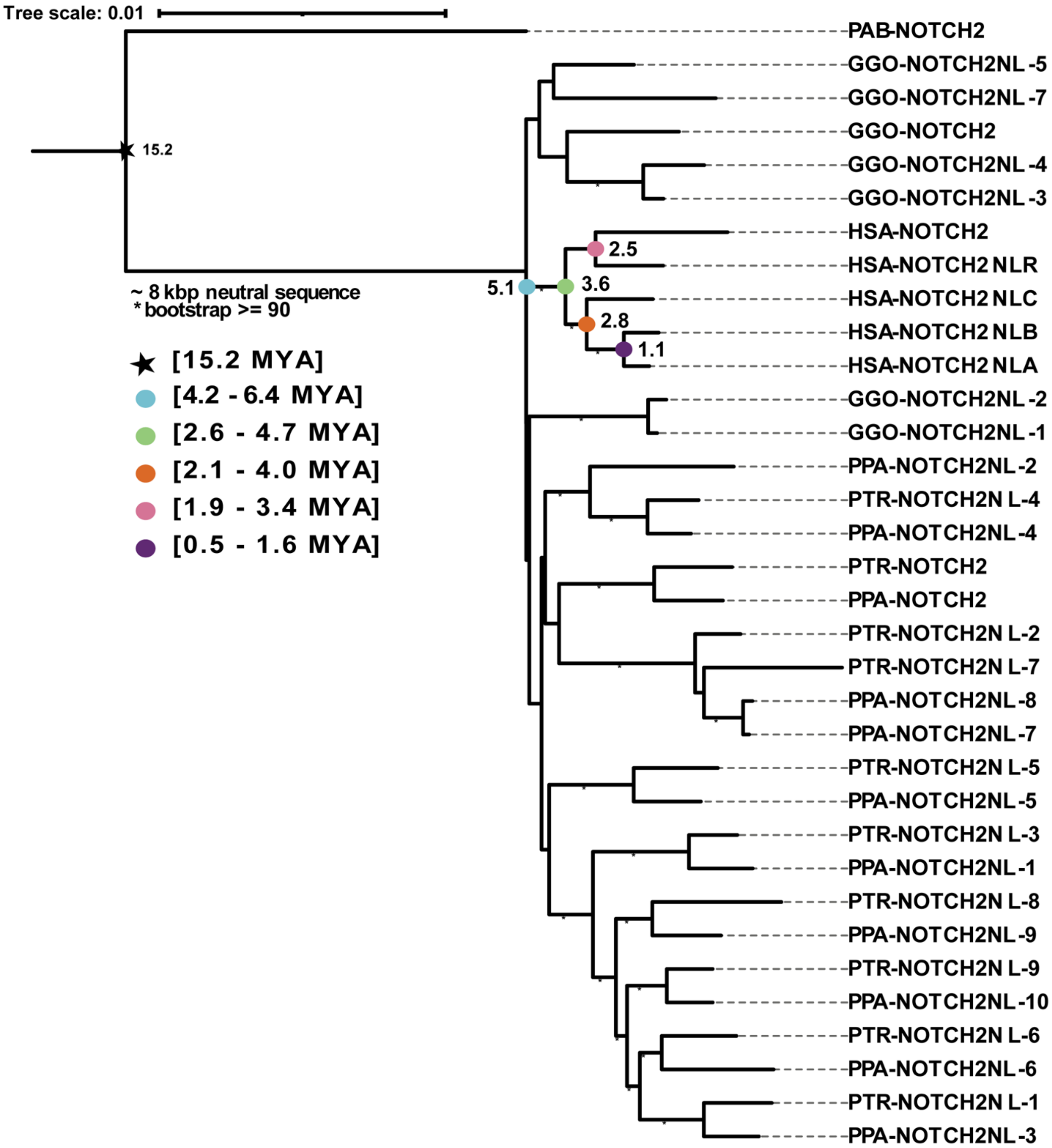

Figure S3. Extended NHA phylogeny, related to Figure 3. An alternate maximum likelihood phylogeny based on an MSA of 8 kbp from intron 3 of *NOTCH2/NL* sequence from paralogs of five ape species, using Sumatran orangutan as an outgroup. Boots representing 25/26 NHA homologs. Bootstrap support (>90%) is indicated (asterisk) and is less robust than the phylogeny in Figure 3A but contains more taxa. Estimated divergence times of human paralogs and their confidence intervals are indicated (multicolored dots). Timings were based on human–orangutan divergence time of 15.2 MYA (Methods).

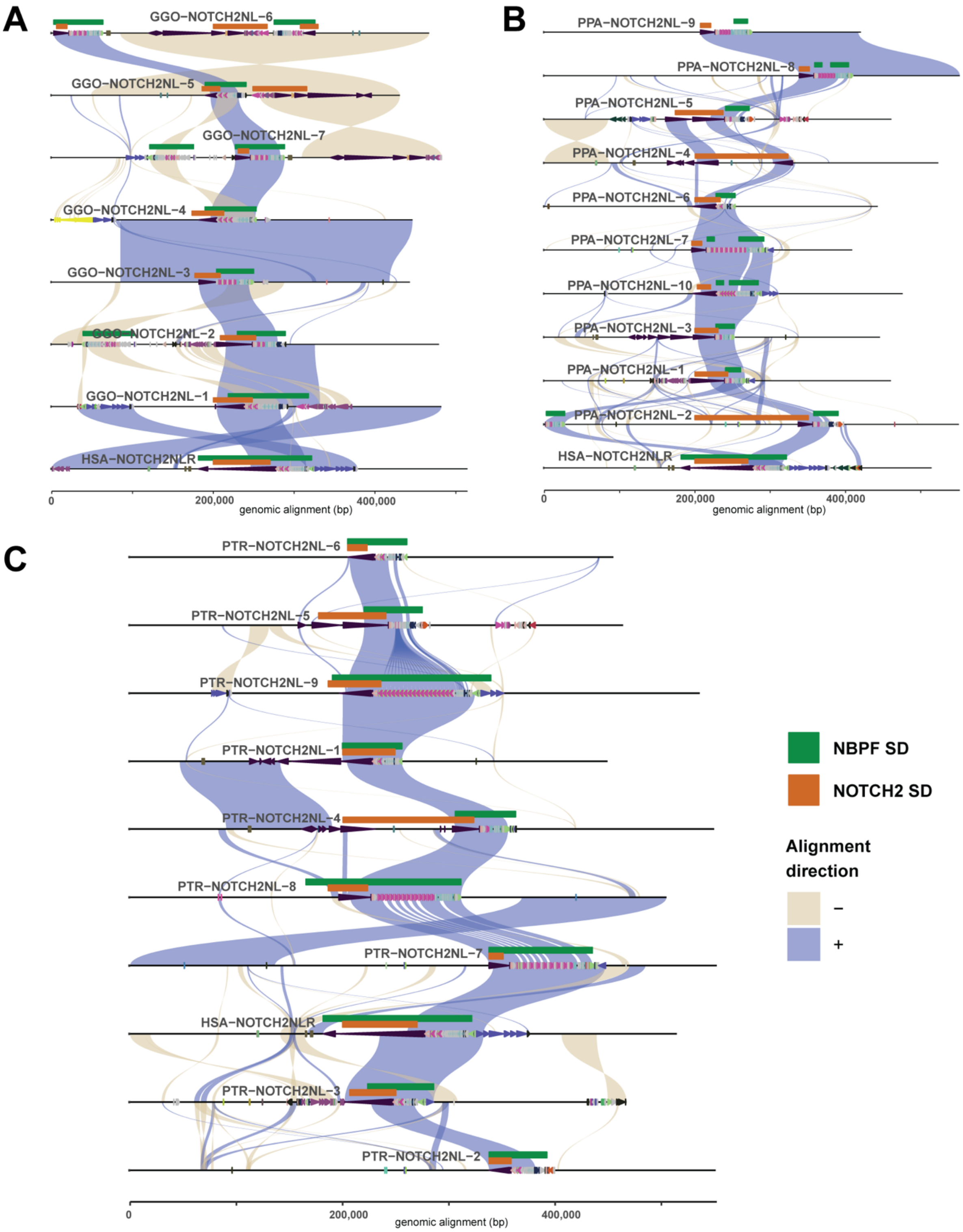

Figure S4. Ladder alignment of NHA *NOTCH2NL* homologs, related to Figure 3. Gorilla **(A)**, bonobo **(B)**, and chimpanzee **(C)** self-alignments (with human *NOTCH2NLR*) show a consistent association between the *NOTCH2/NL* duplication (purple) and the core duplicon *NBPF* (green). However, the breakpoints of these alignments are largely different not just from humans but also each other.

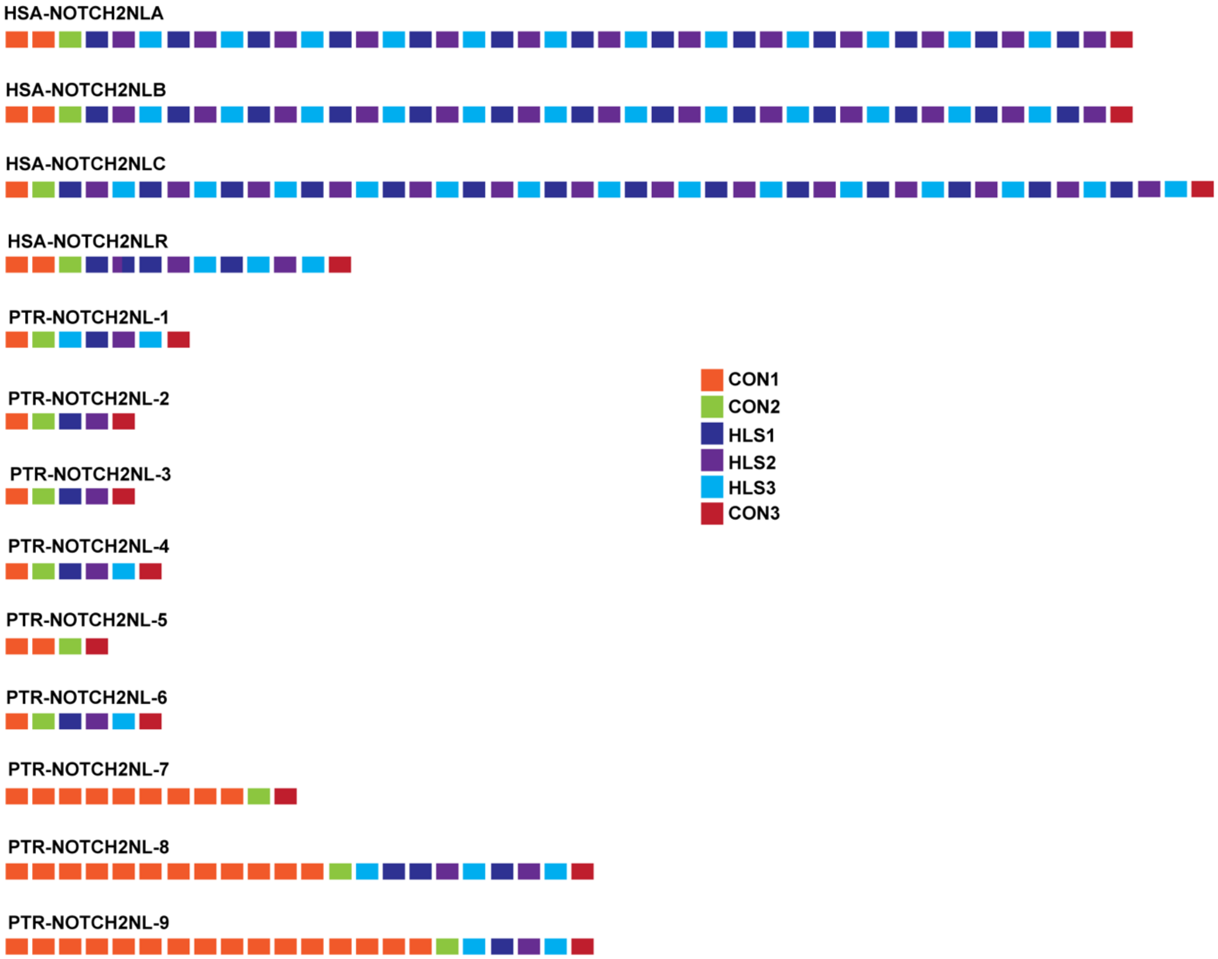

Figure S5. NBPF duplicon analysis, related to Figure 3. For each of the HSA and PTR *NOTCH2NL* copies, the *NBPF* directly downstream is represented in terms of the DUF1220 domains present—CON1, CON2, CON3, HLS1, HLS2, HLS3, based on Fiddes et al. (2019)[S3]. The *NBPF* copies downstream of *NOTCH2NL* copies in PPA and GGO show similar patterns of DUF1220 repeats as well.

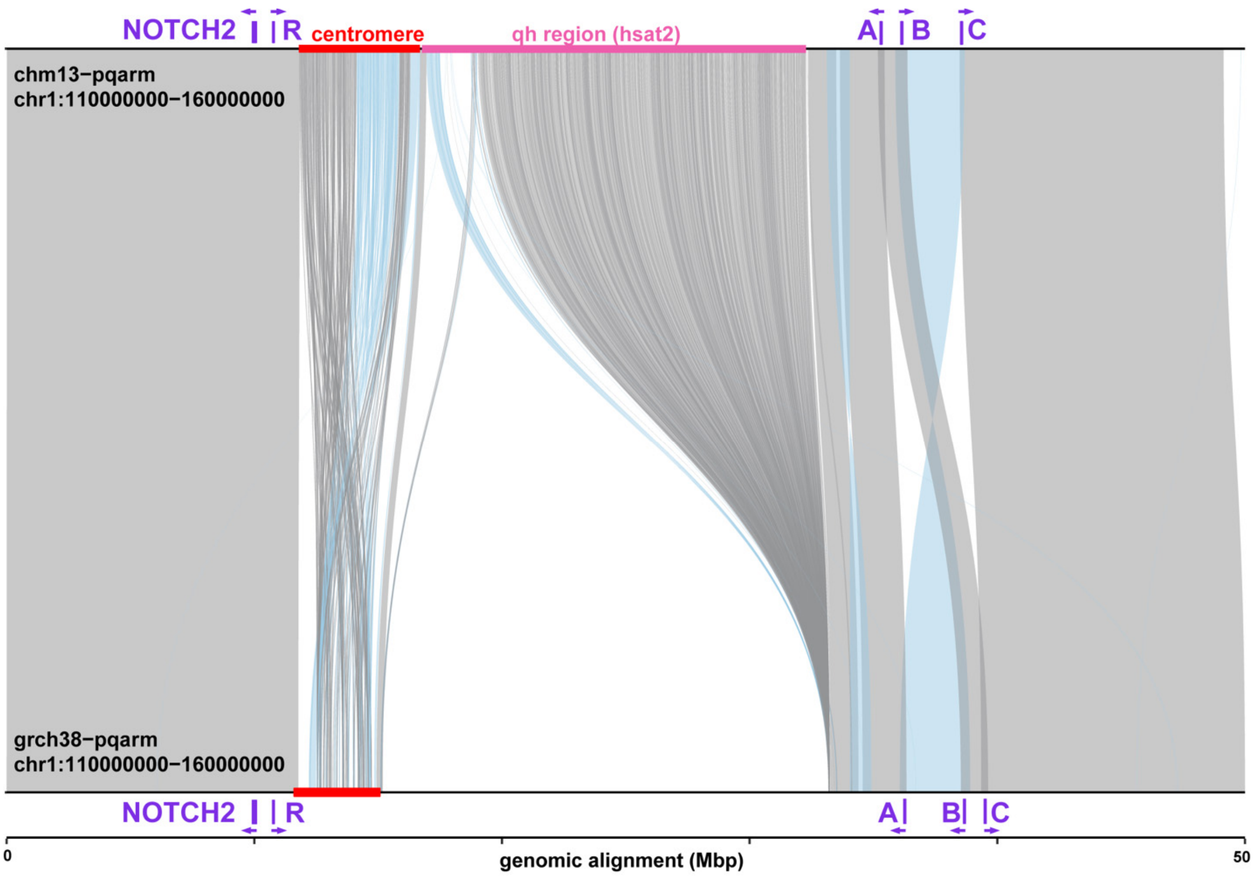

Figure S6. T2T-CHM13 comparison to GRCh38, related to STAR Methods. Organization of the *NOTCH2/NL* region in the completed T2T haploid assembly CHM13 against the previous reference, GRCh38. The T2T-CHM13 assembly has expanded the sequence and annotations that cover the centromere (red) and qh region (pink), which are two large satellite sequences that separate the two *NOTCH2/NL* (purple) loci. A long inversion around *NOTCH2NLB* that may include both *NOTCH2NLA/C* on either side changes the gene's orientation between the two assemblies (relevant alignments have increased color opacity).

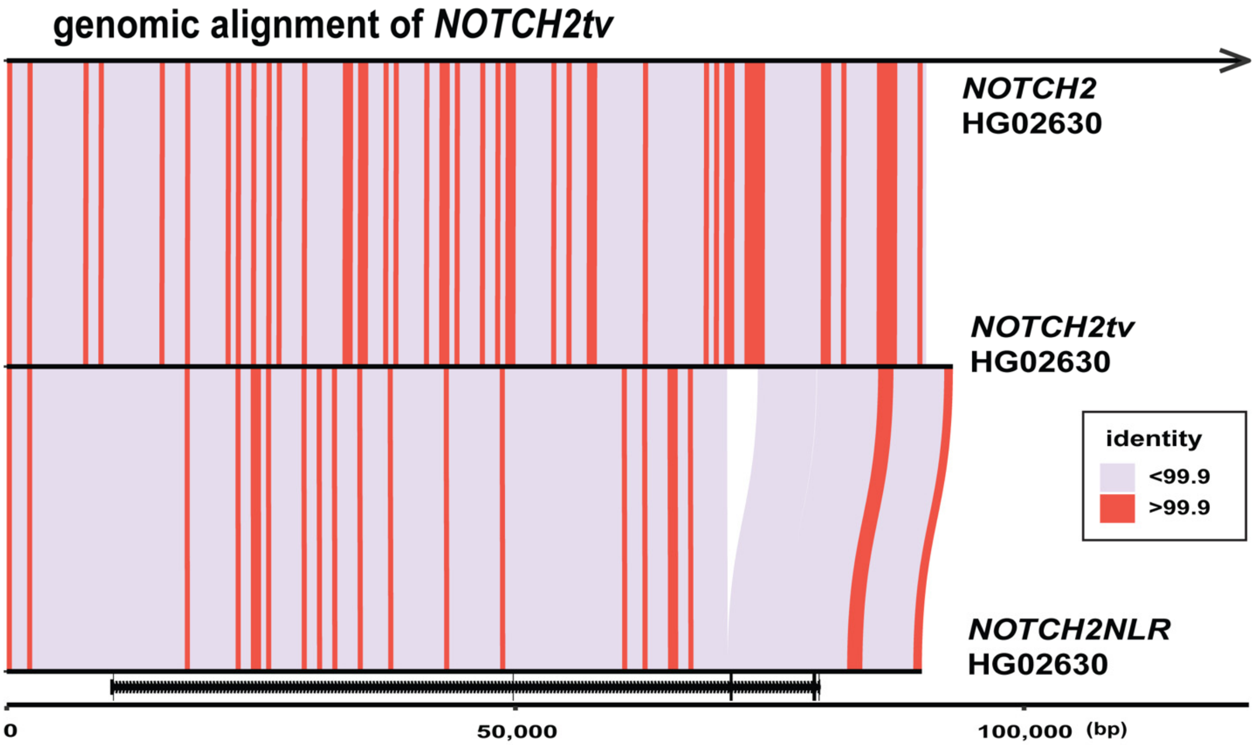

Figure S7. NOTCH2NLR to NOTCH2tv gene conversion, related to Figure 5. Nucleotide alignment of *NOTCH2tv* (middle) to ancestral *NOTCH2* (top) and *NOTCH2NLR* (bottom) confirms larger stretches of near perfect sequence identity (red≥99.9%) between *NOTCH2tv and NOTCH2*, consistent with IGC.

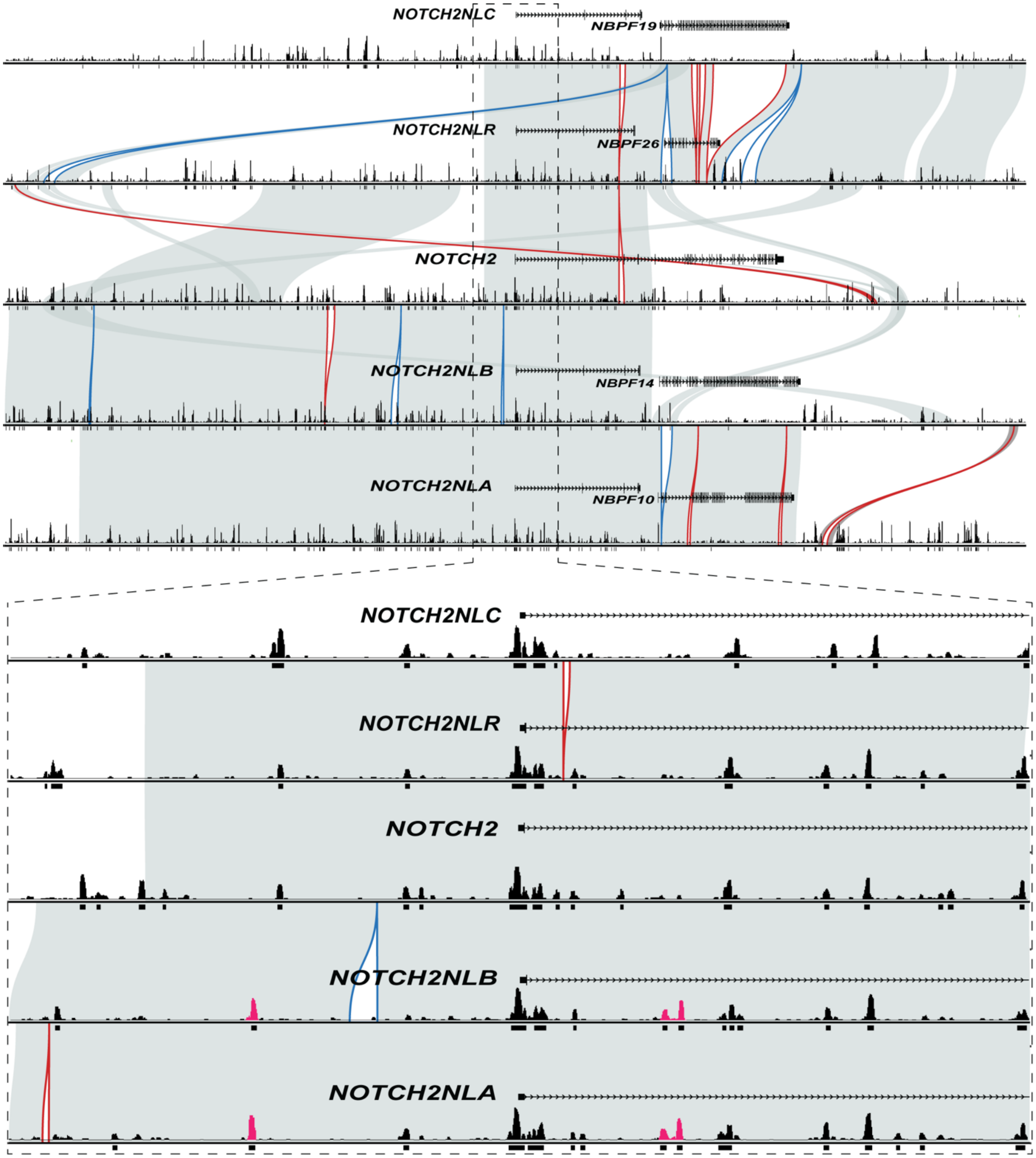

Figure S8. Regulatory architecture of T2T-CHM13, related to STAR Methods. Fiber-seq chromatin actuation peaks for each T2T-CHM13 *NOTCH2/NL* paralog in the context of homology (gray) and gene model 300 kbp on either side of the TSS. Examples of signals of paralog-specific actuation (pink) are shown in pop-out panel (25 kbp on either side of TSS) in *NOTCH2NLA* and *NOTCH2NLB*. Structural variants like insertions (blue) and deletions (red) are outlined.

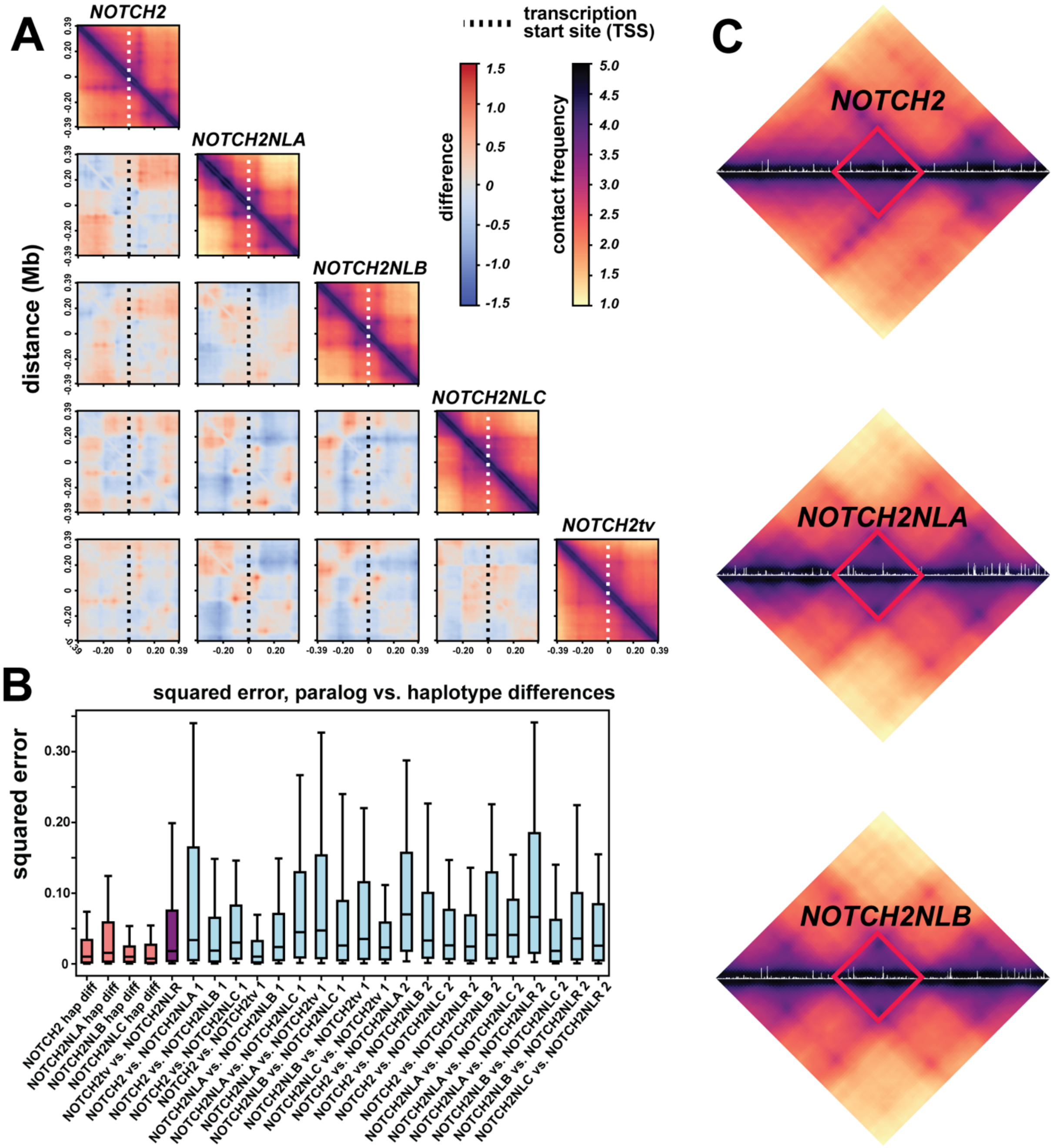

Figure S9. Predicted TADs and associated regulatory architectures in *NOTCH2NL* paralog regions, related to Figure 6. A) FiberFold TAD *NOTCH2NL* paralog predictions and pairwise heatmap of contact similarities and differences. Dotted lines represent gene TSS. **B)** Squared error chromatin accessibility differences between haplotypes and pairwise paralog comparisons. **C)** Fiber-seq chromatin actuation overlaid on TAD predictions for *NOTCH2*, *NOTCH2NLA*, and *NOTCH2NLB* to show which regulatory peaks are included in contact regions.

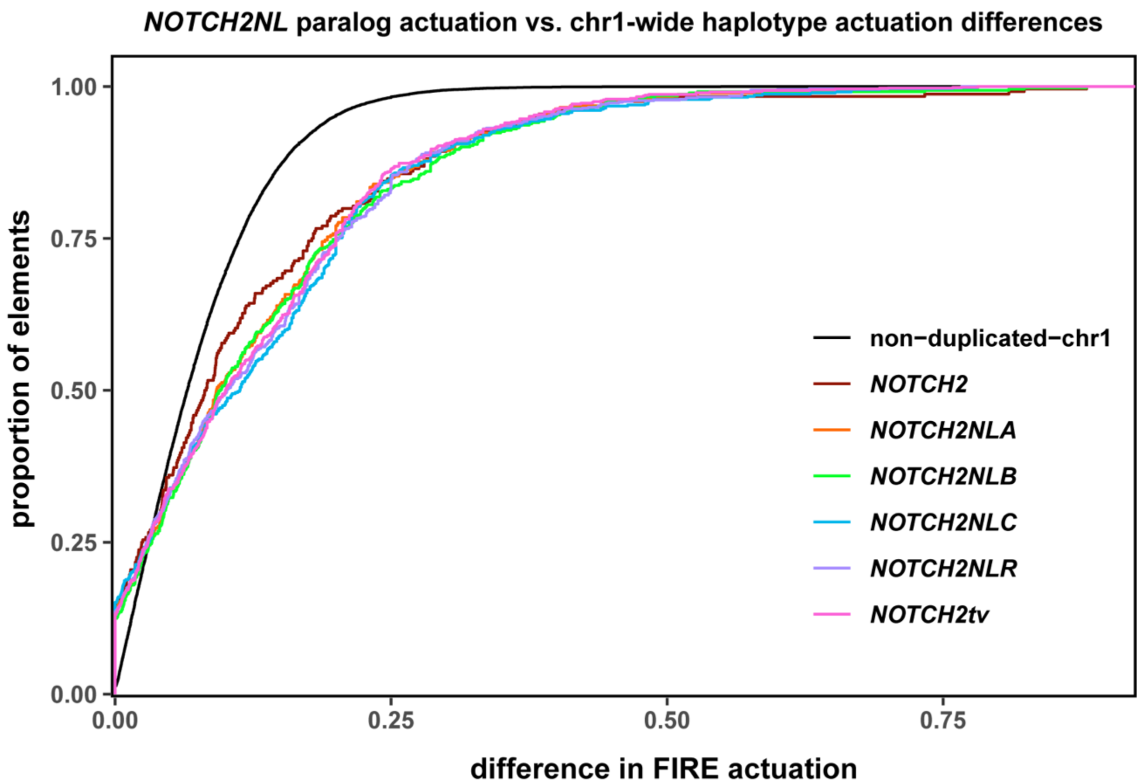

Figure S10. Comparison of *NOTCH2NL* paralog and haplotype actuation, related to Figure 6. The model of expected haplotype differences on chromosome 1 using brain organoid data mapped to T2T-CHM13 (black) shows a higher proportion of regulatory elements with less actuation difference than what is seen for the different *NOTCH2NL* paralogs.

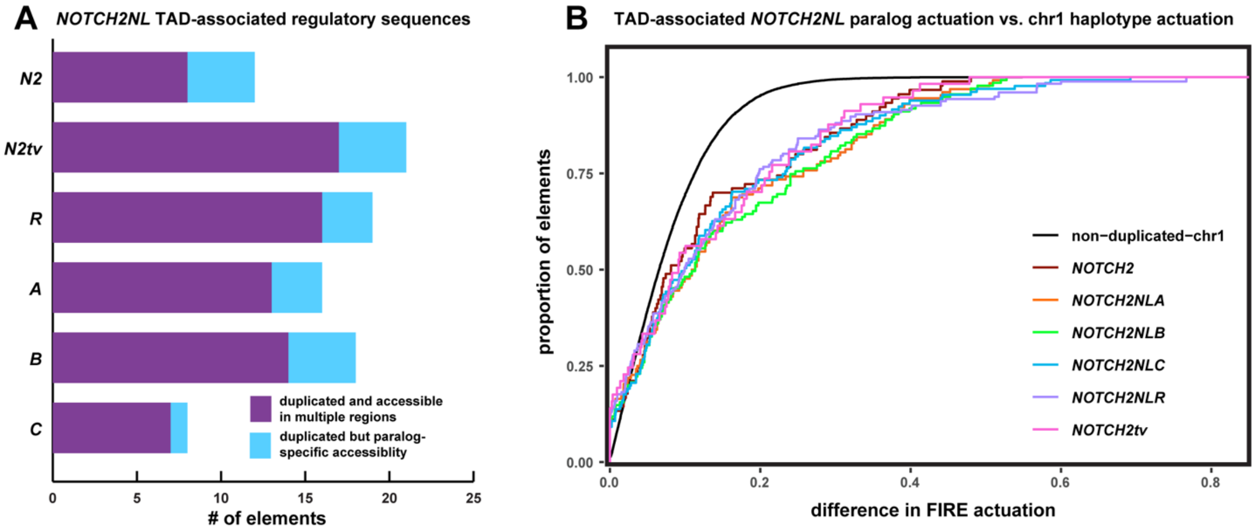

Figure S11. TAD-restricted regulatory analysis, related to Figure 6. A) Bar graph showing categorization of the TAD-restricted accessible elements surrounding each *NOTCH2NL* paralog based on the presence of duplicate sequence and accessibility at that sequence on the different paralogs.
B) A TAD-restricted model of expected haplotype differences on chromosome 1 using brain organoid data mapped to T2T-CHM13 (black) also shows a higher proportion of regulatory elements with less actuation difference than what is seen for the different *NOTCH2NL* paralogs.

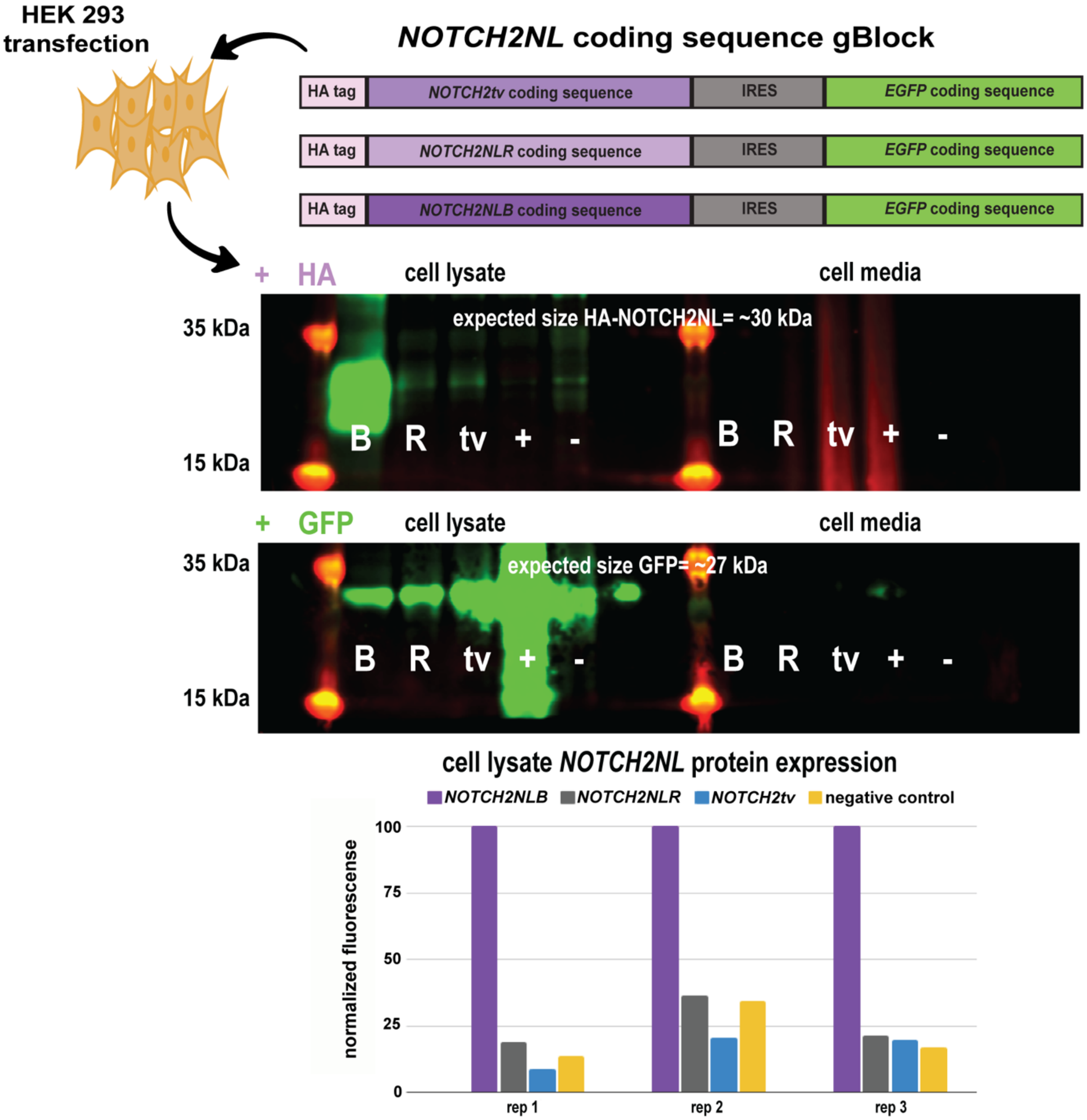

Figure S12. NOTCH2tv protein expression, related to STAR Methods. gBlocks containing an HA antibody tag, *NOTCH2NL* CDS, and *EGFP* CDS were cloned into vector DNA and transfected into HEK293 cells. Antibody staining for HA (NOTCH2NL) and GFP were done on both the cell lysate and media. HA expression fluorescence was normalized using GFP for cell lysate, which shows stable expression of NOTCH2NLB only.

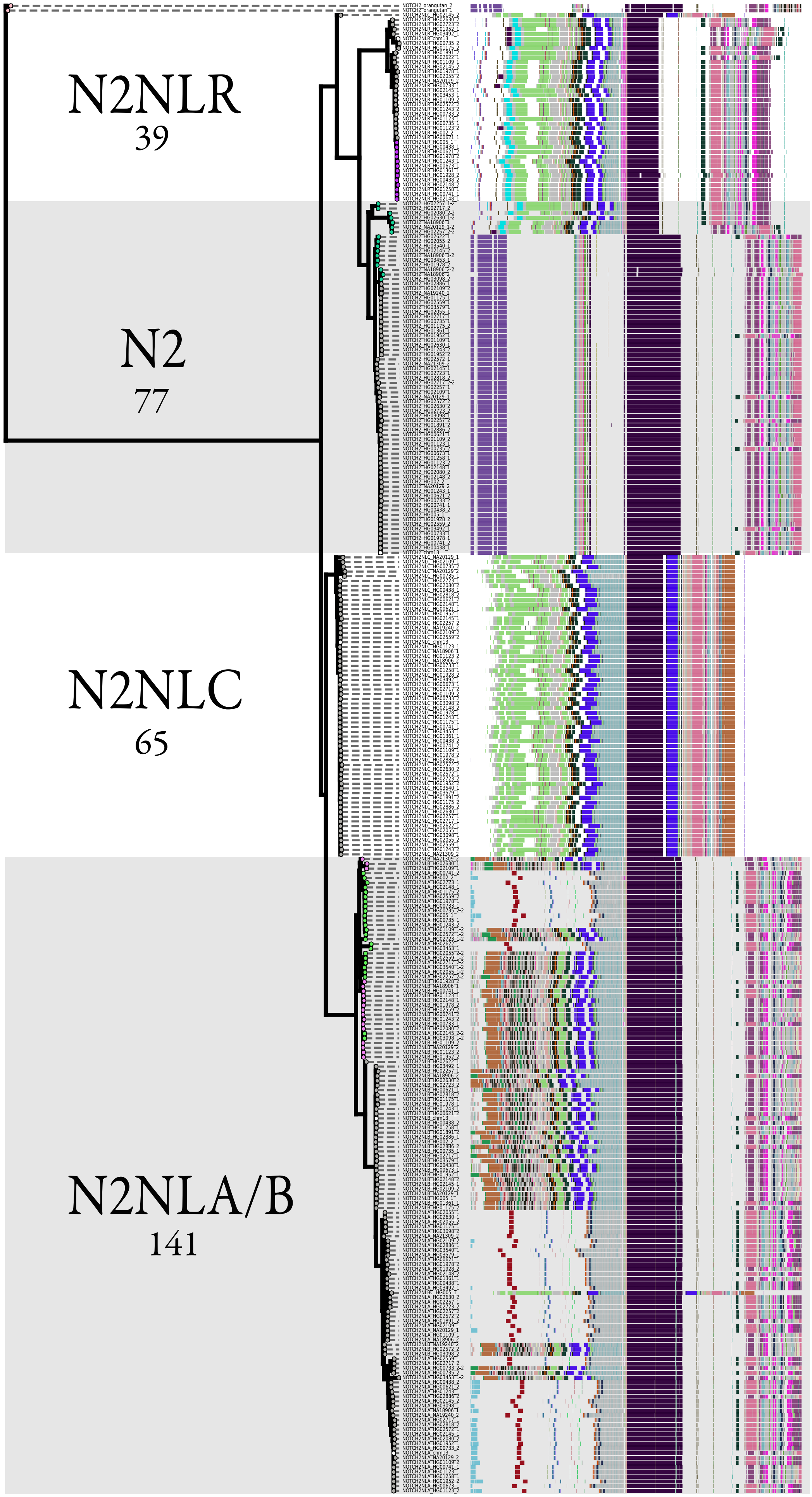
N2NLR
39
N2
77
N2NLC
65
N2NLA/B
141

Figure S13. Phylogeny of human *NOTCH2NL* genetic variation, related to Figures 4 and 5.
(figure on previous page)

SUPPLEMENTAL REFERENCES

[S1] Szamalek, J.M., Goidts, V., Cooper, D.N., Hameister, H., and Kehrer-Sawatzki, H. (2006). Characterization of the human lineage-specific pericentric inversion that distinguishes human chromosome 1 from the homologous chromosomes of the great apes. Hum. Genet. *120*, 126–138. https://doi.org/10.1007/s00439-006-0209-y.

[S2] Fiddes, I.T., Lodewijk, G.A., Mooring, M., Bosworth, C.M., Ewing, A.D., Mantalas, G.L., Novak, A.M., van den Bout, A., Bishara, A., Rosenkrantz, J.L., et al. (2018). Human-Specific NOTCH2NL Genes Affect Notch Signaling and Cortical Neurogenesis. Cell *173*, 1356-1369.e22. https://doi.org/10.1016/j.cell.2018.03.051.

[S3] Fiddes, I.T., Pollen, A.A., Davis, J.M., and Sikela, J.M. (2019). Paired involvement of human-specific Olduvai domains and NOTCH2NL genes in human brain evolution. Hum. Genet. *138*, 715–721. https://doi.org/10.1007/s00439-019-02018-4.
