# Supplementary material for: Genetic diversity and regulatory features of human-specific NOTCH2NL duplications
Source: Cell Genom. 2026 Mar 30;6(6):101194. doi: 10.1016/j.xgen.2026.101194 (PMC13261667; doi:10.1016/j.xgen.2026.101194)
Supplement: Document S2. Article plus supplemental information [file mmc4.pdf]

Cell Genomics

Article

Genetic diversity and regulatory features of human-specific *NOTCH2NL* duplications

Graphical abstract

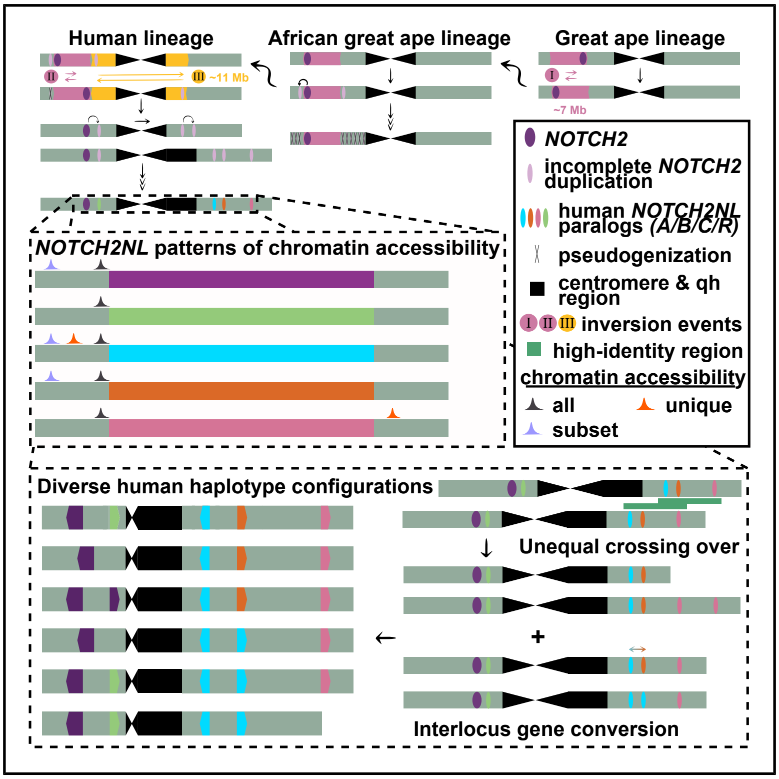

Authors

Taylor D. Real, Prajna Hebbar, DongAhn Yoo, ..., Sofie R. Salama, Andrew B. Stergachis, Evan E. Eichler

Correspondence

absterga@uw.edu (A.B.S.),
ee3@uw.edu (E.E.E.)

In brief

Human-specific *NOTCH2NL* segmental duplications are implicated in brain cortical expansion. Analysis of 82 long-read assemblies from apes and humans shows independent duplications. Human-specific *NOTCH2NL* genes are shaped by large-scale structural variation and gene conversion. Long-read transcriptomic/epigenetic technologies discover rapid changes in gene structure and paralog-specific regulatory landscapes across duplications.

Highlights

- Recurrent *NOTCH2NL* duplications occurred in great apes, including humans (~3 mya)
- All tested human haplotypes have a *NOTCH2NLA* gene
- *NOTCH2NLB* and *NOTCH2NLR/C* are variably present due to gene conversion and deletion
- Paralog-specific accessible elements are candidate drivers of *NOTCH2NL* expression

Real et al., 2026, Cell Genomics *6*, 101194
June 10, 2026 © 2026 The Authors. Published by Elsevier Inc.
https://doi.org/10.1016/j.xgen.2026.101194

CellPress

Cell Genomics

CellPress
OPEN ACCESS

Article

Genetic diversity and regulatory features of human-specific *NOTCH2NL* duplications

Taylor D. Real,[1] Prajna Hebbar,[2,3] DongAhn Yoo,[1] Francesca Antonacci,[4] Ivana Pačar,[2,3] Danilo Dubocanin,[5] Mark Diekhans,[3] Gregory J. Mikol,[6] Oyeronke G. Popoola,[7] Benjamin J. Mallory,[1] Mitchell R. Vollger,[8] Philip C. Dishuck,[1] Xavi Guitart,[1] Allison N. Rozanski,[1] Katherine M. Munson,[1] Kendra Hoekzema,[1] Jane E. Ranchalis,[8] Shane J. Neph,[8] Adriana E. Sedeño-Cortés,[8] Benedict Paten,[2,3] Sofie R. Salama,[3,9] Andrew B. Stergachis,[1,8,10,*] and Evan E. Eichler[1,10,11,12,*]

[1]Department of Genome Sciences, University of Washington School of Medicine, Seattle, WA 98195, USA
[2]Department of Biomolecular Engineering, University of California, Santa Cruz, Santa Cruz, CA 95064, USA
[3]UC Santa Cruz Genomics Institute, University of California, Santa Cruz, Santa Cruz, CA 95060, USA
[4]Department of Biosciences, Biotechnology and Environment, University of Bari, Bari 70125, Italy
[5]Department of Genetics, School of Medicine, Stanford University, Palo Alto, CA 94305, USA
[6]College of Natural & Agricultural Sciences, University of California, Riverside, Riverside, CA 92521, USA
[7]Department of Psychology and Neuroscience, University of North Carolina, Chapel Hill, Chapel Hill, NC 27514, USA
[8]Division of Medical Genetics, Department of Medicine, University of Washington School of Medicine, Seattle, WA 98195, USA
[9]Department of Molecular, Cell and Developmental Biology, University of California, Santa Cruz, Santa Cruz, CA 95064, USA
[10]Brotman Baty Institute for Precision Medicine, Seattle, WA 98195, USA
[11]Howard Hughes Medical Institute, University of Washington, Seattle, WA 98195, USA
[12]Lead contact
*Correspondence: absterga@uw.edu (A.B.S.), ee3@uw.edu (E.E.E.)
https://doi.org/10.1016/j.xgen.2026.101194

SUMMARY

NOTCH2NL (*NOTCH2-N-terminus-like*) genes arose from ape-specific chromosome 1 segmental duplications implicated in human brain cortical expansion, including an incomplete *NOTCH2* gene. Genetic characterization of these loci and their regulation is complicated because they are embedded in large, nearly identical duplications that predispose to recurrent microdeletion syndromes. Using near-complete long-read assemblies generated from 70 human and 12 ape haploid genomes, we show independent recurrent duplication among apes with protein-coding copies emerging in humans 2.2–3.7 million years ago. We distinguish *NOTCH2NL* paralogs present in every human haplotype (*NOTCH2NLA*) from copy-number-variable ones. We also characterize large-scale structural variation, including gene conversion, for 28% of haplotypes, leading to a previously undescribed paralog, *NOTCH2tv*. Finally, we apply Fiber-seq and long-read transcript sequencing to human dorsal forebrain organoids to characterize the regulatory landscape and find that the most fixed paralogs, *NOTCH2* and *NOTCH2NLA*, harbor the greatest number of paralog-specific elements potentially driving their regulation.

INTRODUCTION

Notch signaling, a mechanism of cell communication conserved throughout the metazoan kingdom, is uniquely altered in humans due to a recent ape segmental duplication (SD) of the *NOTCH2* gene.[1,2] SDs have both restructured primate genomes and led to the emergence of lineage-specific gene families, resulting in potentially new functions, including distinct developmental fates.[3,4] In the case of *NOTCH2NL* (*NOTCH2-N-terminus-like*), the gene family is a ~70 kbp SD encompassing the four N-terminal exons of *NOTCH2* and includes a unique final fifth exon exapted from the fourth intron of *NOTCH2*. While *NOTCH2NL*-like sequences exist in several primates, the NOTCH2NL protein appears to be expressed only in the developing human brain, suggesting it is functionally human specific.[1] In humans, there are three truncated paralogs, *NOTCH2NLA*, *NOTCH2NLB*, and *NOTCH2NLC*; all are expressed, and the predicted proteins only differ by a few amino acids (aa).[1] Experimental work has shown that NOTCH2NL interacts with NOTCH2 and modulates the NOTCH2 signaling pathway.[1,2] Specifically, NOTCH2NL increases the number of self-renewal divisions of progenitor radial glia while delaying differentiation of these cells into neurons. Prioritizing self-renewal over differentiation has been proposed to enable the human brain to increase neuronal mass during cortical neurogenesis.[1,2] In addition to its potential role in the expansion of the human brain, mutations in the *NOTCH2/NL* gene family or their associated SDs underlie four distinct genetic disorders, including Alagille syndrome,[5,6] neuronal intranuclear inclusion disease,[7,8] and chromosome 1q21.1 distal duplication/deletion and TAR (thrombocytopenia-absent radius) syndromes.[9–11]

Historically, complex high-identity regions enriched in SDs, such as the *NOTCH2NL* locus from chromosome 1p12-1q21,

This is an open access article under the CC BY license (http://creativecommons.org/licenses/by/4.0/).

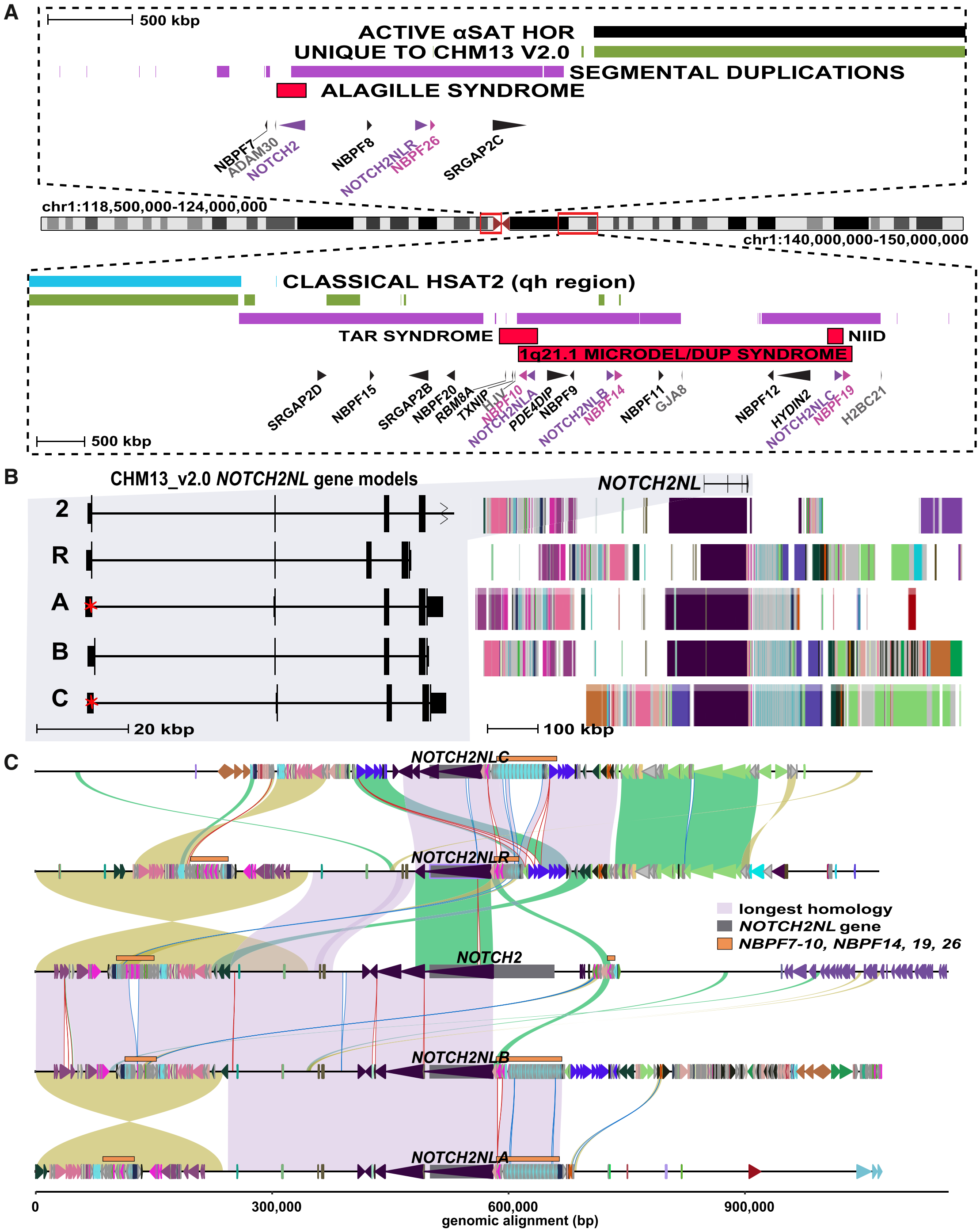

Figure 1. Genome structure and organization of the *NOTCH2NL* gene family

(A) Long-range organization of *NOTCH2*/*NOTCH2NL* loci in the T2T-CHM13 reference genome, including centromere satellite annotations of active alpha satellite (αSat) higher-order repeats (HORs) (black) and classical human satellite 2 (hsat2; secondary constriction [qh] region[26]) (blue), regions unique to the T2T-CHM13 assembly (green), intervals of segmental duplications (SDs) (purple), and Mendelian and genomic disorders associated with specific regions/paralogs (red). A subset of genes is depicted, including *NOTCH2NL* (purple), *NBPF* genes that are directly downstream of *NOTCH2NL* (pink), non-duplicated genes (gray) immediately adjacent to the SD blocks, and others (black). Scale bars in their respective regions represent 500 kbp.

(B) Duplicon organization as defined by DupMasker (STAR Methods; Table S6) flanking the *NOTCH2NL* region and intron/exon structure of genes in T2T-CHM13 v.2.0[27] (http://genome.ucsc.edu). Red asterisks mark the nontraditional CTG start that the browser annotations do not take into consideration. Scale bar for gene models represents 20 kbp while the duplicon scale bar represents 100 kbp.

(legend continued on next page)

Table 1. Greater *NOTCH2NL* region pairwise homology matrix in T2T-CHM13

	NOTCH2	*NOTCH2NLR*	*NOTCH2NLA*	*NOTCH2NLB*	*NOTCH2NLC*
NOTCH2	–	115,690	326,052	556,583	118,987
NOTCH2NLR	99.7	–	342,476	328,624	217,296
NOTCH2NLA	99.3	99.3	–	416,088	182,193
NOTCH2NLB	99.3	99.3	99.7	–	265,598
NOTCH2NLC	99.2	99.0	99.1	99.2	–

have been difficult to sequence and assemble with short-read technologies. Duplicated *NOTCH2NL* copies are embedded in much larger blocks (often hundreds of kbp to a few Mbp in length) of SDs associated with other genes, such as the core duplicon *NBPF* (neuroblastoma breakpoint family).[1,12–16] These regions, subject to different mutational processes such as interlocus gene conversion (IGC),[17] make paralogous sequence variants unreliable as tags unless such common IGC patterns in diverse humans are characterized. As a result, studies of human genetic variation have frequently excluded these regions; standard genome-wide association studies and attempts to functionally characterize via ENCODE and GTEx are almost nonexistent due to their dependence on short-read sequencing platforms.[18,19]

In this study, we address these limitations by using long-read sequencing data and associated pangenome and telomere-to-telomere (T2T) resources generated from nonhuman primates (NHPs) and a diverse set of humans (Human Pangenome Reference Consortium [HPRC]).[3,20,21] Our goal was to annotate structural differences, expression, and regulatory changes in the context of human genetic variation—a feat only possible with a completely resolved haplotype sequence. Understanding variation in patterns of the human haplotypes will have the added benefit of helping to define breakpoints associated with patients harboring chromosome 1q21 deletions and duplications in the future.[10,11]

RESULTS

Structure of the *NOTCH2NL* gene family in a complete human genome assembly

In the fully assembled T2T-CHM13 haploid genome,[22] *NOTCH2NLA*, *NOTCH2NLB,* and *NOTCH2NLC* map to the q-arm (Figure 1A) and define the functional human-specific duplications. All daughter duplications are approximately 10–11 kbp proximal upstream of an *NPBF* gene, which has been implicated as a partner in creating *NOTCH2NL* fusion transcripts (Figure 1A).[16,23] In T2T-CHM13, *NOTCH2NLA* and *NOTCH2NLC* gene models are distinct from the other paralogs because their first exon is suggested by gene annotation to be untranslated (Figure 1B), due to unique mutations that remove the canonical *NOTCH2* initiator methionine and secretory signal.[1] We annotated all structural variants >500 bp among the paralogs (Table S1) and defined synteny stretches extending beyond the *NOTCH2NL* genes (Table 1; Figure 1C). We also used the order and orientation of duplicons flanking each *NOTCH2NL* copy as defined by DupMasker[24] to generate a "barcode" of each locus to readily identify regions in other human genomes when compared to T2T-CHM13 (Figure 1B; STAR Methods). Representing the surrounding sequence in terms of the higher-order duplication content over 1 Mbp regions helped define orthologous locations in the presence of the homogenizing effects of IGC.[25] Importantly, all copies of *NOTCH2NL* show one breakpoint with respect to the ancestral *NOTCH2* corresponding to the *NBPF* duplicon that demarcates the 3′ end of each derived-duplicated gene (Figure 1C). Additionally, the presumptive pseudogene *NOTCH2NLR* is missing the upstream nongenic ancestral *NOTCH2* sequence present in *NOTCH2NLA* and *NOTCH2NLB*.

Independent *NOTCH2NL* duplications and large-scale restructuring of ape chromosome 1

We compared the extent of synteny of the corresponding *NOTCH2NL* loci among finished NHP genomes[3,20] (Figures S1 and S2A). We identified 26 distinct *NOTCH2NL* SDs among nonhuman apes (NHAs) (average of 215 kbp) (Table S2). We find that the mean SD length between *NOTCH2NL* SDs in humans (311 kbp) and other NHAs (215 kbp) is not significantly different (p = 0.42; two-sided t test), indicating that this locus was unstable and began to duplicate in the common ancestor of the great apes. Consistent with Fiddes et al.,[1] all NHA homologs appear truncated with respect to *NOTCH2*. All are missing different canonical exons relative to the known human *NOTCH2NL* gene models (Figure 1B). Our comparative analysis (Figure 2) indicates that three distinct inversion events occurred during the evolution of the chromosome 1p21.2-q23.2 region. The first inversion (I) occurred in the ancestor of African great apes, flipping the region into its current orientation in gorilla, chimpanzee, and bonobo. Subsequently, in the human lineage, this region reverted to its ancestral configuration (inversion II). Additionally, an expansion of SDs in humans coincided with a human-specific pericentric inversion (III), corresponding to the

(C) Stacked SVbyEye plot of 1 Mbp regions flanking human *NOTCH2NL* genes (gray squares), contrasting syntenic regions in direct orientation (green/lavender) versus inverted alignments (yellow). Annotations include different *NBPF* genes in the region (orange). Note: the two large inversions between *NOTCH2*/*NOTCH2NLR* and *NOTCH2NLA*/*NOTCH2NLB*, respectively, are the result of proximity due to overlapping sequence. Duplicons are defined by DupMasker (colored triangles).
See also Tables S1 and S6.

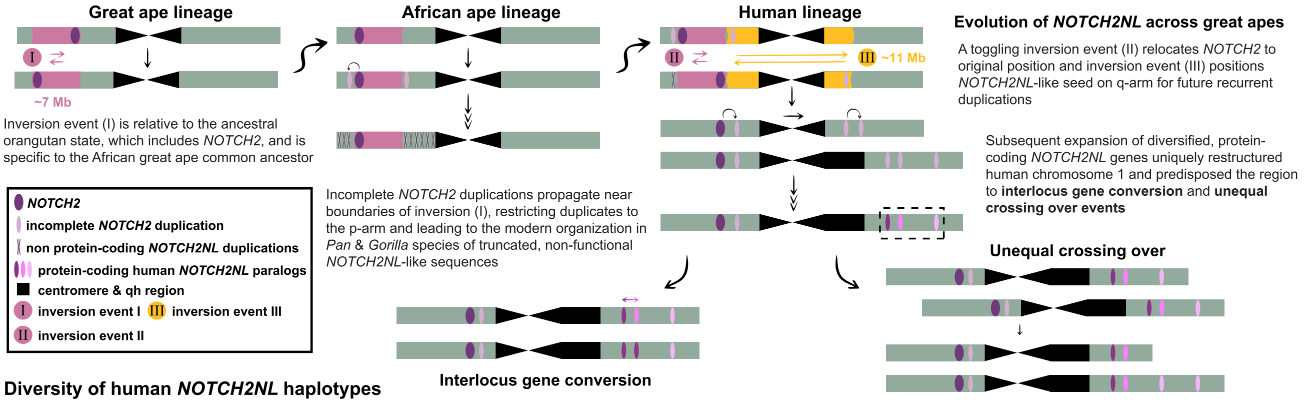

Figure 2. Summary of chromosome 1 rearrangement and *NOTCH2NL* evolution

NOTCH2NL-like genes have duplicated ancestrally and independently in three separate ape lineages, with protein-coding ability exclusively in the human lineage. Three inversion events, beginning in the African ape common ancestor, in addition to the subsequent duplication events, helped position *NOTCH2NL* to the current human genomic configuration. The organization and high homology maintained between human *NOTCH2NL* paralogs and surrounding regions promote structural variation associated with gene conversion and unequal crossing over.

See also Figures S1 and S2 and Table S2.

rearrangement originally described by Yunis and Prakash[28] and subsequently refined to 154 and 562 kbp breakpoint intervals at chromosome 1p11.2 and 1q21.3, respectively.[29] We estimate that the region encompassing inversions II and III is 17 Mbp larger than the syntenic region in chimpanzee and orangutan. In humans, this event uniquely positioned *NOTCH2NLA*, *NOTCH2NLB*, and *NOTCH2NLC* on the long arm of chromosome 1, splitting the *NOTCH2NL* locus across the centromere when it had previously always existed on a single chromosome arm (Figure S2B). The region encompassing *NOTCH2NL* paralogs has undergone significant restructuring via duplication and inversion among all great apes but especially in the human genome.

Next, we constructed a maximum likelihood (ML) phylogeny using shared intronic sequence (intron 2) from a subset (8/29) of African ape *NOTCH2/NL* paralogs, the five human paralogs, and *NOTCH2* from Sumatran orangutan (STAR Methods). We observe a distinct monophyletic clade populated only by the human T2T-CHM13-*NOTCH2NL* paralogs (Figures 3A and S3), suggesting independent duplication or recent human-specific IGC. The topology of the tree further suggests independent expansions in the gorilla and *Pan* ape lineages, including lineage-specific expansions. In contrast, bonobo and chimpanzee share ancestral copies prior to their divergence (1–2 mya).[3] Using orangutan divergence and the species as an outgroup (STAR Methods), our analysis predicts that the human lineage of *NOTCH2NL* copies emerged early in human evolution, around 4.9 mya (4.3–5.7 mya), after African ape speciation, and that such duplications were also occurring among the other ape lineages (albeit independently or subsequently derived from a larger initiating ancestral ape duplication). Approximately 3.0 mya (2.2–3.7 mya), the human-specific copies begin to diverge, distinguishing *NOTCH2NLC* from *NOTCH2NLA/B*. *NOTCH2NLA* and *NOTCH2NLB* appear to have diverged around 1.7 mya (1.2–2.3 mya), although once again, IGC may have homogenized these loci, as there is ample evidence of ongoing gene conversion (see patterns of *NOTCH2NL* human genetic variation) in present-day humans for these two copies, which map in closest proximity to one another.

NHA *NOTCH2NL* copies and fusion transcripts

Like humans, nearly all homologs in NHA map 11–12 kbp upstream of *NBPF* genes (25/26) (Table S3; Figures S4 and S5). We leveraged long-read RNA sequencing (RNA-seq) data primarily from testis and fibroblast/lymphoblastoid cell lines of chimpanzee, bonobo, and gorilla[3] to annotate valid open reading frames (ORFs) across the majority of *NOTCH2NL*-like loci (23/26) in a set of NHA transcripts. While most transcripts predict fusions of *NOTCH2NL* with other genes (24/26), some transcripts (14/26) predict proteins most similar to the human *NOTCH2NLR* sequence with ORF lengths ranging between 235 and 246 aa. The vast majority (19/26) of NHA *NOTCH2NL*-like loci create fusion transcripts where the site of fusion is at the 3′ end of *NOTCH2NL* and the 5′ end of *NBPF* (Figure 3B), which is similar to what is seen in all human paralogs (not including *NOTCH2*) but especially in human *NOTCH2NLR* (see transcriptional expression and protein stability of *NOTCH2NL* paralogs). This included previously unreported gene fusions between *NOTCH2NL* and other genes, including *LRIG2* and *SORT1* (Table S3). All transcripts and predicted proteins similar in structure to *NOTCH2NLR* (specifically without additional gene fusion) in NHAs are distinct from human *NOTCH2NLR* because they have lost either exon 1 (containing the secretory pathway signal sequence) or exon 2 (Figure 3C). The phylogeny, different duplication architecture, and varying gene structures all support a largely independent evolutionary expansion among the great apes. Humans appear to be the only species with *NOTCH2NL* transcripts that are predicted to make a stable protein, most likely because NHA copies lack the 4 bp deletion that was found to be essential for *NOTCH2NLA/B/C* protein expression.[1] We confirm this 4 bp deletion, which is after the fourth aa in the fifth exon, modifies the final 19–20 aa of the C terminus in

CellPress
OPEN ACCESS

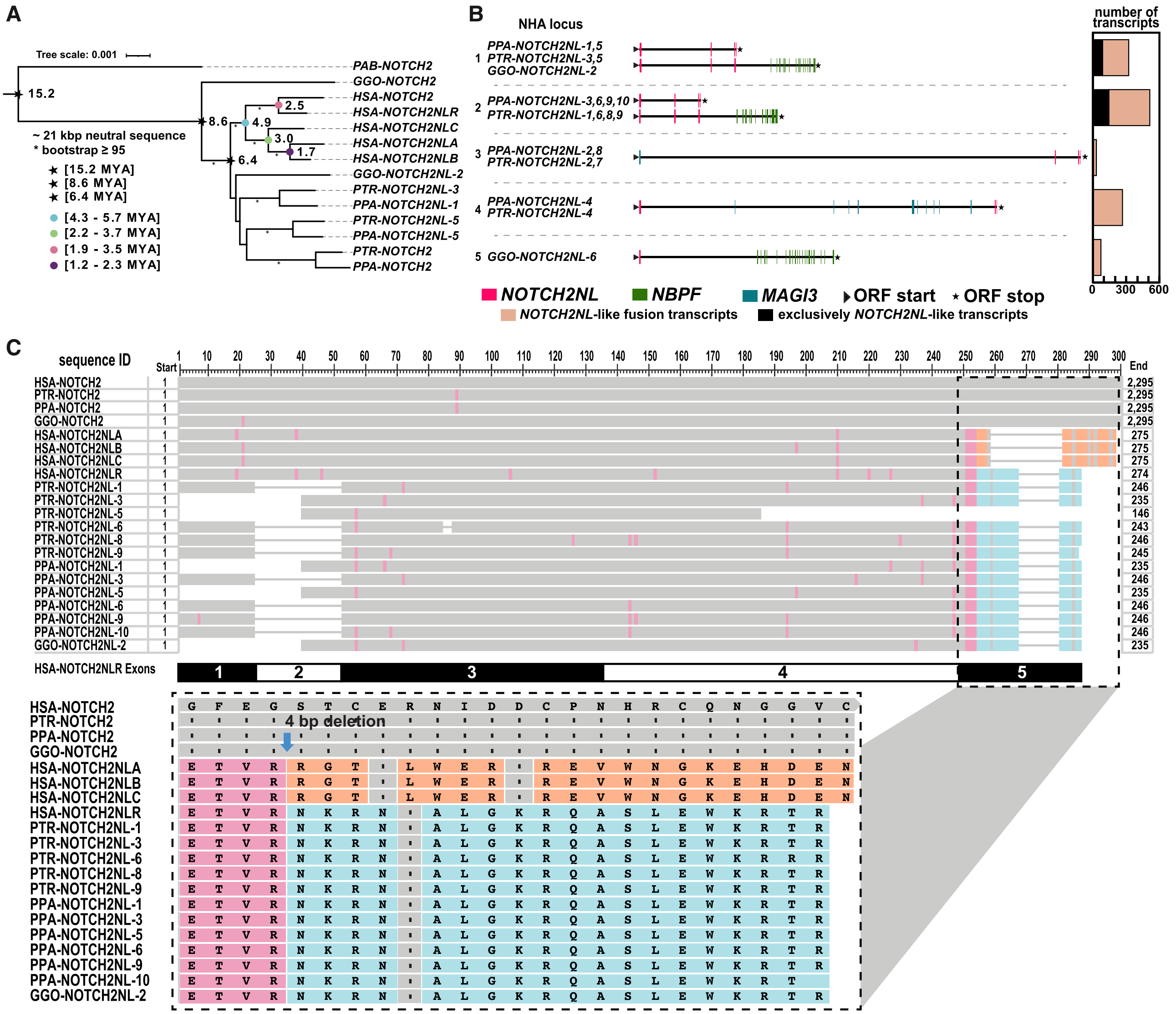

Figure 3. *NOTCH2/NL* duplications across multiple ape species

(A) A maximum likelihood phylogeny based on a multiple sequence alignment (MSA) of 21 kbp of intronic *NOTCH2/NL* sequence from a subset of paralogs of five ape species, using the Sumatran orangutan as an outgroup. Bootstrap support (>95%) is indicated (asterisk). Estimated divergence times of human paralogs and their confidence intervals are indicated (multicolored dots). Timings were based on a human-orangutan divergence time of 15.2 mya (STAR Methods). Tree scale bar represents 0.001 substitutions per site (0.1% divergence).

(B) Fusion transcript models expressed in different NHA grouped by species (GGO, *Gorilla gorilla*; PPA, *Pan paniscus*; PTR, *Pan troglodytes*; left), a schematic of the exon organization (middle), and transcript abundance (right) with the relative proportion of *NOTCH2NL*-like fusions (orange) compared to exclusive *NOTCH2NL*-like transcripts (black). These sets are representative of 20/26 *NOTCH2NL*-like loci in NHA from testis, fibroblast/lymphoblastoid cell lines, iPSCs, neuroepithelium, and neural progenitor cells.

(C) MSA of predicted protein sequences from 13/26 NHA NOTCH2NL-like loci, NOTCH2 from the NHAs, and all five NOTCH2/NL paralogs from human. The pop-out of exon 5 alignment shows that all NHAs possess the same unmodified C terminus as NOTCH2NLR, which lacks a 4 bp deletion necessary for expression.[1] See also Figures S3–S5 and Table S3.

not just a paralog-specific but also a human-specific fashion (Figure 3C).

Patterns of *NOTCH2NL* human genetic variation

To understand human *NOTCH2NL* genetic variation, including structural differences among human haplotypes, we initially selected 94 haploid genome assemblies recently generated by the HPRC.[21] We manually validated each assembly for these loci by assessing contiguity, annotated gaps, the presence of collapses, and verifying true structural variants. Of these assemblies, 69 (73%) passed quality control for sequence and structural accuracy; 54% of these genomes were of African origin, while the remaining 46% were of non-African origin, representing in total 14 distinct population groups (Table S4). Among these 69

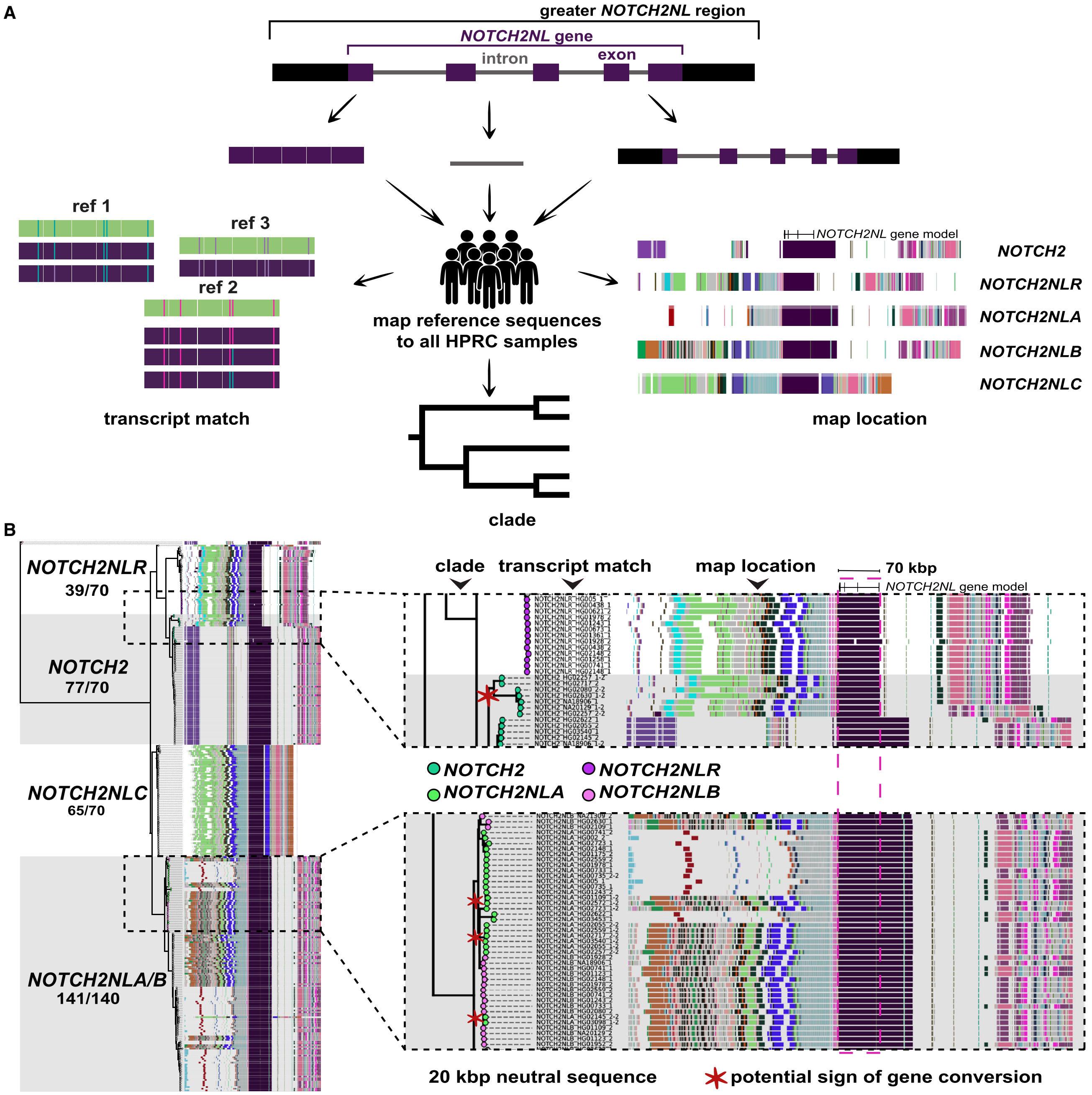

Figure 4. Patterns of human *NOTCH2NL* structural variation and gene conversion

(A) Workflow to characterize *NOTCH2NL* paralog identity based on (1) best transcript match (defined as the fewest mismatches with respect to T2T-CHM13 reference coding sequence annotation), (2) phylogenetic clade (assignment to nearest monophyletic grouping based on *NOTCH2* intronic ML tree), and (3) map location (defined here as the long-range genomic context based on DupMasker barcodes).

(B) Analysis of 70 human haplotypes depicts the clade assignment based on the phylogenetic tree, then the best transcript match, and finally the long-range duplicon organization based on the assembled HPRC genomes. Disagreements in paralog identity suggest potential gene conversion; examples are marked with red asterisks. *NOTCH2NL* gene model scale bar represents 70 kbp.

See also Figure S13 for phylogeny without gene conversion panels and Table S4.

genomes, we distinguished 11 different structural configurations operationally defining H1 based on the T2T-CHM13 reference configuration described above (70 haplotypes in total). Given the anticipated high degree of IGC,[1,30] we developed a tripartite workflow (Figure 4A) to assign *NOTCH2NL* identity. First, we examined the best transcript match by identifying which *NOTCH2NL* coding sequence best matches *NOTCH2NL* copies assigned in the T2T-CHM13 reference (STAR Methods). Second,

CellPress
OPEN ACCESS

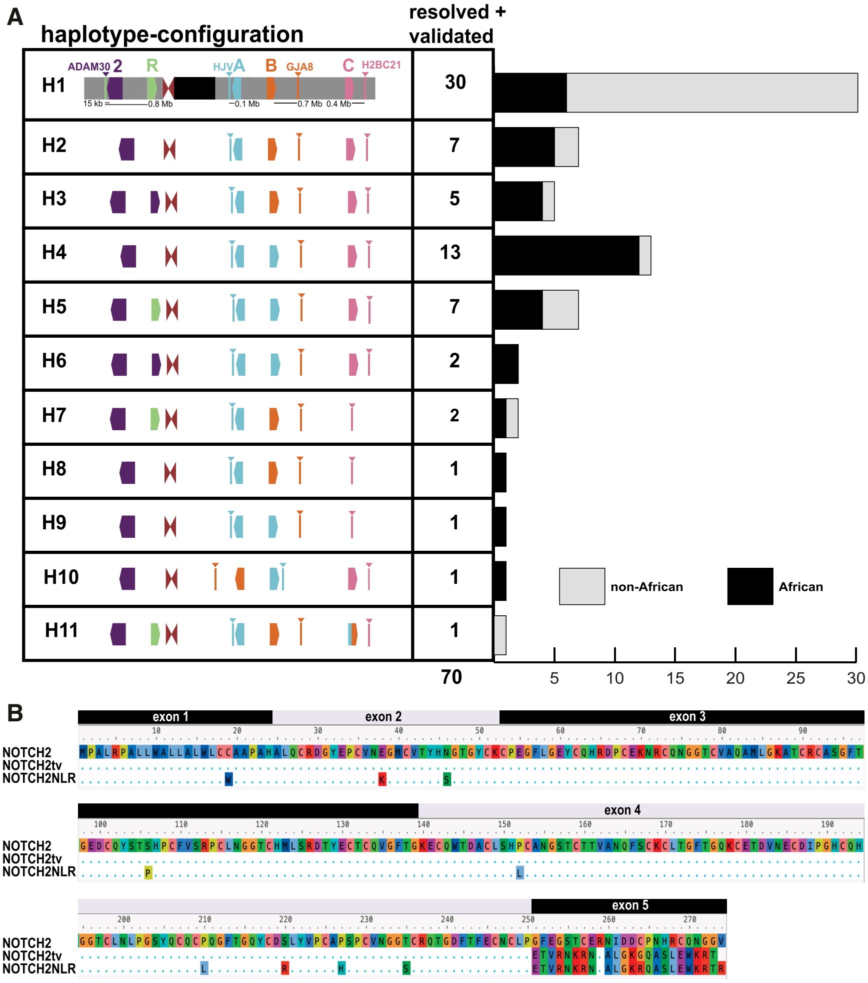

Figure 5. *NOTCH2NL* structural diversity and *NOTCH2tv*

(A) A simplified schematic summary of the *NOTCH2NL* haplotype organization and frequency based on 69 sequence-resolved HPRC genomes and the T2T-CHM13 reference. The proportion of African and non-African samples associated with each haplotype configuration suggests some may be ancestral.

(B) Alignment of predicted aa for the three paralogs suggests that *NOTCH2tv* arose as a result of an interlocus gene conversion (IGC) of *NOTCH2NLR* from *NOTCH2.*

See also Figure S7 and Table S4.

we used *NOTCH2NL* intronic sequence to construct a tree identifying a phylogenetic framework for each *NOTCH2NL* haplotype, assigning different haplotypes to related clades. Third, we used the extended duplication organization as defined by the DupMasker barcode described above (Figure 1B) to examine the long-range organization of the region flanking *NOTCH2NL*. The combination of these results (Figure 4B) was used to delineate IGC events and further define 11 distinct human haplotype configurations (H1–H11) (Figure 5A).

Of the haplotype-resolved genomes, 42% share the canonical haplotype configuration (H1) observed in T2T-CHM13, thus representing the major human haplotype (Figure 5A). Among the remaining ten configurations, six are observed more than once in this subset from the HPRC (Figure 5A). Notably, the haplotype configuration currently represented in the standard human reference, GRCh38, is characterized by a nearly 2.5 Mbp inversion that reverses the orientation of *NOTCH2NLB* relative to T2T-CHM13 (Figure S6), yet it has not been observed in any other human haplotype. Thus, GRCh38 either represents a minor variant or a misassembly. Our analysis indicates that the *NOTCH2NLR* pseudogene is not present in 43% (30/69) of all haploid assemblies (H2–H4, H6, and H8–H10). H2, H4, and H8–H10 configurations strictly represent deletion events, which occur in 33% (23/69) of haploid assemblies. *NOTCH2* is invariant with respect to copy number; however, so too is *NOTCH2NLA*, which is present in all sequenced human haplotypes but sometimes as more than one copy due to gene conversion. *NOTCH2NLC* is deleted in four haploid assemblies with three different configurations (H7–H9), while in another (H11), it appears to have been converted to a *NOTCH2NLA/B* hybrid. Other than *NOTCH2NLR*, a likely pseudogene, *NOTCH2NLB* appears to be absent from human haplotypes based on sequence homology due to IGC between *NOTCH2NLA* and *NOTCH2NLB*. As a result, only 46 out of the expected 69 haplotypes actually carry it.

Overall, in this study, 33% (23/69) of haplotypes (H4, H5, H6, and H9) appear to have a *NOTCH2NLB*-to-*NOTCH2NLA* conversion event (Figure 5A). This IGC event is shown by our workflow, where both the phylogeny and duplication barcode are disrupted between *NOTCH2NLA* and *NOTCH2NLB* (Figure 4B), and has been confirmed by multiple sequence alignment (MSA) of predicted protein sequences from our samples. In addition, we observed a second gene conversion event that had not been previously characterized in depth: direct conversion of the *NOTCH2NLR* pseudogene from the *NOTCH2* ancestral locus (Figure 4B), which is 654 kbp away. 10% of haplotypes (H3 and H6) harbor a copy of the gene at this locus that resembles a truncated version of *NOTCH2* rather than *NOTCH2NLR*; this includes two H6 haplotypes that exhibit both gene conversion events (making the combined amount of IGC across haplotypes 42% instead of 43%). As a result, all eight aa changes associated with NOTCH2NLR now match the ancestral NOTCH2 (Figure 5B). When surveying gene conversion at the gene level, we see more >99% identity bins between *NOTCH2* and the gene conversion product than between the product and *NOTCH2NLR* (Figure S7). Notably, the H3 and H6 haplotypes are significantly enriched in African samples (p = 0.007, Fisher's exact test), suggesting that *NOTCH2NLR*-to-*NOTCH2*

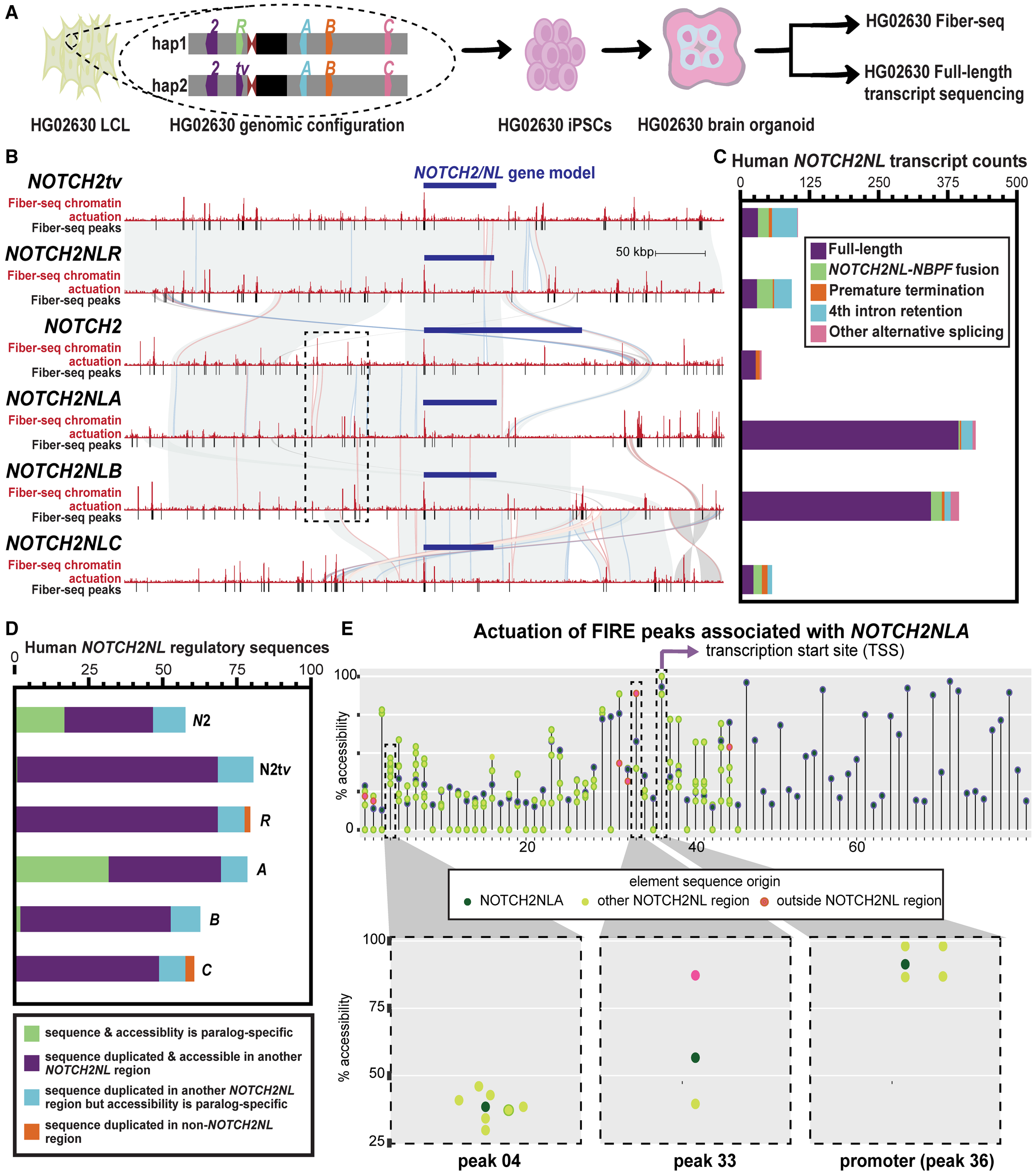

Figure 6. Regulatory architecture and transcription of *NOTCH2NL* in brain organoids

(A) HG02360 was reprogrammed into iPSCs, differentiated into brain organoids, and then subjected to Fiber-seq and Iso-Seq to define putative regulatory elements and generate full-length transcripts.

(B) Fiber-seq peaks and chromatin actuation sites for each *NOTCH2/NL* paralog in the context of homology (gray), gene model, and transcription start site (TSS). Dotted black boxes surround elements specific to a region only shared across *NOTCH2*, *NOTCH2NLA*, and *NOTCH2NLB*; although the underlying sequence is nearly identical, we show paralog-specific actuation signals. Scale bar of the alignment represents 50 kbp.

(C) The absolute abundance of full-length transcripts compared to other premature termination, fusion, and intron retention products in brain organoids.

(legend continued on next page)

conversion could be an ancestral gene conversion event. Because of its sequence similarity to *NOTCH2*, we renamed this version of *NOTCH2NLR* as *NOTCH2tv* (*NOTCH2*-truncated version), a sixth paralog in the gene family.

Accessible chromatin architecture surrounding the *NOTCH2NL* paralogs

Having established the genetic architecture of *NOTCH2NL* paralogs and their surrounding loci, we next sought to determine how the structure of SDs influences the gene regulatory landscape surrounding *NOTCH2NL* paralogs. Gene regulatory landscapes are often defined using techniques such as assay for transposase-accessible chromatin using sequencing (ATAC-seq) and DNase-seq,[31,32] which can detect accessible chromatin elements. However, SDs have been historically excluded from these short-read-based techniques, as it is largely impossible to unambiguously assign short reads to large, highly identical SD regions. Fiber-seq, in contrast, is a long-read-based approach for mapping chromatin architecture,[33] and we previously demonstrated that this approach can be used to map chromatin accessibility to complex genomic regions, such as SDs.[34]

To determine whether there are any accessible chromatin elements within the vicinity of *NOTCH2NL* paralogs, we mapped CHM13 Fiber-seq data[35] to the 300 kbp regions surrounding each *NOTCH2NL* paralog transcription start site (TSS) in T2T-CHM13 and compared the accessible chromatin maps from each paralog based on genetic synteny (Figure S8). Overall, this revealed that each *NOTCH2NL* paralog shares a promoter with accessible chromatin within CHM13 cells. Notably, each paralog shows a largely unique surrounding accessible chromatin landscape along regions that lack synteny, suggesting that the non-syntenic sequence contributes to regulatory differences among the five *NOTCH2NL* paralog regions.

To further investigate the accessible chromatin landscape surrounding all the *NOTCH2NL* paralogs, including *NOTCH2tv*, in more relevant tissue, we generated Fiber-seq and long-read full-length transcript sequencing data from brain organoids of an HPRC sample that contains *NOTCH2tv* in addition to the other four copies of *NOTCH2NL* (Figures 6A–6C; STAR Methods). Specifically, lymphoblast cell lines from HG02630 were reprogrammed into induced pluripotent stem cells (iPSCs), which were then differentiated toward dorsal forebrain tissue. Such cerebral cortex organoids are used to model early fetal brain development, when *NOTCH2NL* is most highly expressed.[1] HG02630 has both a *NOTCH2tv* haplotype and a canonical *NOTCH2NLR* haplotype, enabling the evaluation of the gene regulatory landscape of all the *NOTCH2NL* paralogs within the same individual. Overall, as in CHM13, we observe that *NOTCH2* and all *NOTCH2NL* paralogs, including *NOTCH2tv*, have accessible promoter elements that show a similar degree of chromatin accessibility (Figure 6B). In contrast to the promoter, accessible elements in the vicinity of these genes, once again, exhibit marked paralog-specific patterns in terms of both their location and accessibility.

Comparison of these accessible Fiber-seq-based chromatin maps (STAR Methods) with underlying synteny maps reveals that nearly 87% of all accessible chromatin elements within 300 kbp of the *NOTCH2NL* TSSs share duplicated sequence with at least one other *NOTCH2NL* paralog region. This suggests the prevalent reuse of genomic sequences with putative gene regulatory potential across these SDs (Figure 6D). Furthermore, 84% of those elements near multiple *NOTCH2NL* paralogs also show some level of accessible chromatin at more than one of their paralogous sites, demonstrating that, by and large, these elements retain their ability to form accessible chromatin when rearranged in different genomic positions. Overall, the accessible chromatin landscape surrounding each *NOTCH2NL* paralog appears predominantly populated by these multi-paralog accessible chromatin elements. However, these paralogs often diverged in their magnitude of chromatin accessibility (Figures 6B and 6E) and in their rearrangement relative to the *NOTCH2NL* promoters, indicating that position effects within these different SDs may impact these putative regulatory elements in a quantitative manner rather than simply abrogating their chromatin accessibility.

We also observe that 10% of all accessible chromatin elements within 300 kbp of the *NOTCH2/NL* TSSs map to duplicated sequences on a different chromosome. Five elements exclusively share duplicated sequence with regions on a different chromosome (about 1% of all elements) (Figure 6D). This suggests that the creation of the *NOTCH2NL* SDs was associated with the potential repurposing of accessible chromatin elements from elsewhere in the genome.

In total, we find that 12% of all accessible chromatin elements surrounding *NOTCH2/NL* paralogs are specific to only one paralog. These paralog-specific unique elements are concentrated in the *NOTCH2* and *NOTCH2NLA* regions. Notably, these paralogs are the most fixed for copy-number variation within the human population.

To further refine the scope of regulatory elements more directly associated with each *NOTCH2NL* paralog, or at least their respective promoters, we applied FiberFold[36] to our brain organoid data to predict topologically associated domains (TADs) in *NOTCH2NL* paralog regions in a haplotype-aware manner. FiberFold predicts that each *NOTCH2NL* paralog occupies a distinct 3D genomic environment despite their highly identical underlying sequence, with each *NOTCH2NL* TSS surrounded by a unique 3D landscape (Figure S9A). These paralog-specific 3D landscape patterns are largely consistent across haplotypes, with differences between paralogs more pronounced than differences between haplotypes (Figure S9B). Consistent with this, we also find that chromatin actuation differences between paralogs are more pronounced than differences between haplotypes (Figure S10), despite the high sequence

(D) Bar graph showing categorization of accessible elements surrounding each *NOTCH2NL* paralog based on the presence of duplicate sequence and accessibility at that sequence on the different paralogs. Note that *NOTCH2* and *NOTCH2NLA* have the greatest proportion of paralog-specific sites (dark green).
(E) Percentage of actuation of each accessible regulatory element surrounding the *NOTCH2NLA* paralog (dark green), as well as the percentage of actuation of duplicate sequences for each element that are present surrounding other *NOTCH2NL* paralogs (light green) or outside of the *NOTCH2NL* paralogs (pink).
See also Figures S9–S11.

identity between paralogs. Limiting this analysis to accessible chromatin elements that fall within the predicted TADs for each of the *NOTCH2NL* paralogs confirmed the above findings (Figure S11) and exposed a cluster of elements 63 kbp upstream of the *NOTCH2*, *NOTCH2NLA*, and *NOTCH2NLB* TSSs, which are selectively active on the *NOTCH2NLA/B* SDs but inactive on the *NOTCH2* SD (Figure S9C). Overall, this analysis uncovers marked SD-specific accessible chromatin architectures and nominates specific elements that may result in paralog-specific regulation of the *NOTCH2NL* genes. However, further experiments will be needed to characterize the functional role of elements within TAD boundaries.

Transcriptional expression and protein stability of *NOTCH2NL* paralogs

We found distinct differences within the transcript abundance of each of the *NOTCH2/NL* paralogs (Figure 6C), indicating that these paralog-specific accessible chromatin elements may be creating unique gene regulatory environments for each of the *NOTCH2/NL* paralogs. Specifically, *NOTCH2NLA* and *NOTCH2NLB* had ~3-fold higher steady-state transcript abundance than the other *NOTCH2/NL* paralogs. Ancestral *NOTCH2* has a low abundance of full-length transcripts; however, *NOTCH2* also has very few other transcript types, including fusions. In fact, NHAs appear to show higher levels of fusion transcripts with the ancestor than seen in humans (Figures 3B and 6C). Furthermore, we observed that although the promoter and transcript sequence of *NOTCH2tv* mirrors that of *NOTCH2*, the transcript abundance and composition of *NOTCH2tv* appeared to mirror most closely that of *NOTCH2NLR*. Specifically, *NOTCH2tv* and *NOTCH2NLR* had 102 and 91 transcripts, respectively. However, only ~30% of these transcripts represented canonical full-length transcripts, with the majority arising from fusion transcripts and an incorrectly spliced exon. Surprisingly, the fusion transcripts of all *NOTCH2NL* copies do maintain ORFs predicted to be 1,179–1,574 aa long. Overall, this indicates that, despite the transcript identity of *NOTCH2tv* matching the first four exons of *NOTCH2*, the surrounding gene regulatory architecture in fact mirrors that of *NOTCH2NLR*, potentially impacting the overall function of *NOTCH2tv.* This is likely a result of the gene conversion event being bounded by a 75 kbp syntenic block between *NOTCH2* and *NOTCH2NLR* that spans from just upstream of their promoters to their fourth introns.

Although the gene conversion event results in NOTCH2tv adopting the exact same protein sequence as NOTCH2 for its first 250 aa, NOTCH2tv ends in a distinct 23 aa sequence arising from its terminal fifth exon. NOTCH2NLR was previously shown to form an unstable protein product, which is thought to be driven by its C-terminal sequence. As such, we sought to evaluate whether NOTCH2tv similarly forms an unstable protein product, as its C-terminal sequence shares 91% aa similarity to NOTCH2NLR. We transfected HEK293 cells with a constitutive reporter system containing *NOTCH2tv*, *NOTCH2NLR*, *NOTCH2NLB*, or a negative control and demonstrated that, despite the ability of *NOTCH2tv*, *NOTCH2NLR*, and *NOTCH2NLB* to produce sufficient transcripts in this reporter, only *NOTCH2NLB* resulted in a stable protein product (Figure S12). Together, these data indicate that although gene conversion has generated a paralog of *NOTCH2NL* that contains sequence and promoter features consistent with *NOTCH2*, this paralog retains the overall gene regulatory architecture and transcript patterns of *NOTCH2NLR* and is similarly unable to form a stable protein product and thus likely represents a pseudogene.

DISCUSSION

The rapid expansion of interspersed SDs in the ancestral genome of African apes around 8–15 mya[37] provided the substrate for the human genome to evolve both ape- and species-specific genes. *NOTCH2NL* is one of at least five human- and ape-specific SD gene families that has been implicated in the expansion of the human frontal cortex. This includes genes associated with delayed maturation of synapses and increasing synaptic density (*SRGAP2C*),[38,39] genes such as *NOTCH2NL*[1,2] implicated in cortical progenitor self-renewal, and genes directly promoting cortical and basal progenitor amplification (*TBC1D3*, *ARHGAP11B*, and *CROCCP2*).[40–43] Like *NOTCH2NL*, it is noteworthy that most of these human-specific gene innovations originated from an incomplete duplication that truncated the ancestral gene model, leading to human-specific isoforms. In fact, the incomplete duplication appears to have been a first critical step in either neofunctionalization (*ARHGAP11B*) or dominant-negative effects (*SRGAP2C* and *NOTCH2NL*), where shorter, derived proteins either interfere or modulate ancestral protein function through protein-protein interactions.[1,2,38,39,41,42,44]

We demonstrate that *NOTCH2NL*, like other recently characterized primate gene families, most likely independently expanded in human, chimpanzee, and gorilla (*TBC1D3*, *LRRC37*, and *NPIP*).[45–48] The basis for this recurrence or genomic instability is unknown, but it is interesting that most *NOTCH2NL* ape copies are also associated with the *NBPF* duplicon—an association that is postulated to have co-evolved both in terms of structure and transcriptional regulation, especially in humans.[16] *NBPF* is one of about a dozen core duplicons (along with *TBC1D3*, *LRRC37*, and *NPIP*) implicated as a potential driver of interspersed SDs in the primate lineage.[13–15] Of note, a comparative analysis of *NBPF* associated with *NOTCH2NL* reveals species-specific expansions of different portions of the *NBPF* DUF1220 in different apes (Figure S5), so it is possible that *NBPF* plays a more general role in gene innovation in ape species other than humans.

Notwithstanding this proclivity to duplicate in the common ancestor of great apes, the apparently functional human copies of *NOTCH2NL* arose much later in human evolution. We estimate that the human-specific expansions (or IGC events) occurred around 4.9 mya and diversified over a range of 3.0–1.7 mya (Figure 3A). It is worth noting that other duplicate gene families implicated in the expansion of the human frontal cortex (*SRGAP2C* and *TBC1D3*) show similar evolutionary trajectories beginning to emerge 2–3 mya.[45,49] This is significant in the context of fossil record evidence, which suggests divergence of the genus *Homo* from *Australopithecus* ~2 mya and a subsequent initial increase in archaic hominin cranial volume. There is also evidence of subsequent increases in cranial volume taking

place between 2.0 and 1.5 mya, consistent with the diversification of *NOTCH2NL* genes in humans.[50]

Among human haplotypes, we report polarized signals of gene conversion, which seem especially significant in the case of *NOTCH2NLA*, the only paralog present in all assemblies and that has expanded, seemingly at the expense of *NOTCH2NLB*. This may be a consequence of either an unknown mechanistic or selective bias favoring *NOTCH2NLA* as a donor. In Fiddes et al.,[1] it was also postulated that having a combined dosage of A/B was more important than having two of each paralog. Like *SRGAP2C*,[49] *NOTCH2NLA* represents the most fixed paralog, suggesting functional constraint. Gene birth can be accomplished through duplication,[51] but there is a gap in the research on how IGC may influence this process. When first investigating the understudied paralog *NOTCH2tv*, we hypothesized it was a case of gene conversion reviving a nonfunctional gene, since, so far, *NOTCH2tv* has acquired a promoter and 4-exon N terminus identical to *NOTCH2*. This, in theory, could enable *NOTCH2tv* to regulate expression similar to *NOTCH2*.[23] However, whereas *NOTCH2NLA/B/C* contain a protein-stabilizing 4 bp deletion in their terminal exon (Figure 3C), neither *NOTCH2NLR* nor *NOTCH2tv* has this same 4 bp deletion in its terminal exon (Figure 5B). Consistent with this, we found that like *NOTCH2NLR*, *NOTCH2tv* does not produce a stable protein (Figure S12) and thus is not a fully functional paralog.

By leveraging Fiber-seq and FiberFold, we identify marked paralog-specific gene regulatory patterns surrounding each *NOTCH2NL* SD and paralog. Overall, we find that the different *NOTCH2NL* regions frequently retain duplicated sequences that encompass an accessible chromatin element on at least one paralog region. However, ~14% of elements present within these duplicated sequences exclusively show chromatin accessibility in only one paralog. Furthermore, even for those elements that do show some chromatin accessibility across two or more duplicate sequences, we find that the degree of chromatin accessibility can vary quite substantially between the two duplicates (Figure 6E). This suggests that putative regulatory elements within SDs are subject to positional effects, with the predominant effect being quantitative differences in chromatin accessibility rather than drastic changes to on/off actuation.

In summary, we hypothesize that the dramatic restructuring of the *NOTCH2NL* loci during human evolution led to the only ape lineage with protein-coding copies. This was made possible by a dynamic set of large- and small-scale changes associated with non-allelic homologous recombination, recurrent duplications/deletions,[52] and IGC.[25] Many genes embedded in these regions, including *NOTCH2NL*, are associated with neurologic and developmental phenotypes, including copy-number-variation syndromes, such as 1q21.1 distal duplication/deletion syndrome[11] or TAR syndrome.[9] The fact that this region is among the most frequently rearranged regions of the human genome[53] is a testament to the evolutionary instability that continues to persist in the human population. It is well established that the presence of these human-specific duplications has phenotypic consequences associated with recurrent rearrangement and developmental delay.[10,11] Consistent with the core duplicon hypothesis[54] and population studies that suggest large duplications should be under purifying selection unless some other type of selective force is acting,[55–58] the mutational lability of chromosome 1q21.1 and the emergence of *NOTCH2NL* genes likely represent a significant trade-off between positive and negative selection during human evolution. In the case of *NOTCH2NL*, we hypothesize that the benefits of expanding the cortex must have outweighed the mutational burden of increasing the proportion of high-identity-duplicated sequences in the genome. Our findings suggest that this trade-off is still ongoing. The biased gene conversion that is potentially driving the fixation of *NOTCH2NLA,* as well as the high level of fourth-intron-retention transcripts of *NOTCH2tv* and *NOTCH2NLR*, may be examples of ongoing evolution in this gene family, such as the specification of the functional NOTCH2NL protein and potential acquisition of a novel C terminus.

Limitations of the study

Gene conversion biases

Gene conversion exists at varying degrees throughout this locus, even in T2T-CHM13, which is used as a reference and may be a source of error for analysis of human genetic variation. Although we attempted to identify the largest gene conversion events, both reference effects and smaller events may remain undetected. IGC obscures the timing of duplication events and signals of selection. Additionally, homogenization between highly similar loci where only a few SNV differences distinguish paralogous segments complicates breakpoint definition.

Phylogeny and duplication timing

Although we assess robustness of the phylogenies using bootstrap support, several sources of error remain. The largest source is the timing of speciation events used to calibrate the molecular clock, which are estimates themselves. As noted previously, IGC is also a concern, as any duplication timing estimates we report may capture the time since gene conversion versus the time since the original duplication event.

Gene regulation and Fiber-seq

Fiber-seq is a measure of chromatin accessibility, which is only a proxy for noncoding regulatory regions; it does not distinguish between element types, such as repressors and enhancers, nor address which genes these elements would be regulating. Further work is needed to resolve how paralog-specific regulatory architectures diverge relative to SD sequence identity. Although FiberFold will generate predictions of which genes may be regulated by these accessible chromatin peaks, further efforts are needed to functionally characterize these predictions.

Iso-Seq analyses

Iso-Seq is a measure of steady-state transcripts, which is influenced by both the potency of regulatory elements and the stability of the full-length transcript. Given the marked divergence between *NOTCH2NL* paralogs in terms of the surrounding regulatory elements as well as their 3′ UTR structure, it is anticipated that both of these are contributing to the final steady-state measurements of transcript abundance.

RESOURCE AVAILABILITY

Lead contact

Requests for further information and resources should be directed to and will be fulfilled by the lead contact, Evan E. Eichler (ee3@uw.edu).

Materials availability

The DNA sequence of plasmids generated in this study for NOTCH2NL protein expression has been deposited to GitHub: https://github.com/tdreal/NOTCH2NL-0325/tree/main and Zenodo: https://zenodo.org/records/15022214.

Data and code availability

PacBio HiFi Fiber-seq and Kinnex full-length Iso-Seq from HG02630 brain organoids generated for this study have been made available on NCBI with the BioProject ID: PRJNA1236375. Full-length Iso-Seq from NHAs generated by the Pollen lab for another study that is not yet published are available on NCBI with the BioProject ID: (pending approval). Original western blot images are deposited to GitHub: https://github.com/tdreal/NOTCH2NL-0325/tree/main and Zenodo: https://zenodo.org/records/15022214. No original code is reported in this study. Any additional information required to reanalyze the data reported in this paper is available from the lead contact upon request.

ACKNOWLEDGMENTS

We would like to thank T. Brown for editing this manuscript. We would like to thank Nicolas Altemose at Stanford University for additional support in generating and validating the FiberFold tool. We would like to thank the UW ISCRM facility for performing HG02630 iPSC generation. We would also like to thank the HPRC and Primate T2T Consortium for providing numerous high-quality assemblies for analysis. A.B.S. holds a Career Award for Medical Scientists from the Burroughs Wellcome Fund and is a Pew Biomedical Scholar. This work was supported, in part, by US National Institutes of Health (NIH) grants R01MH120295 to S.R.S., 1DP5OD029630 and 1U01HG013744 to A.B.S., and R01HG010169 and R01HG002385 to E.E.E. E.E.E. is an investigator of the Howard Hughes Medical Institute. This article is subject to HHMI's Open Access to Publications policy. HHMI lab heads have previously granted a nonexclusive CC BY 4.0 license to the public and a sublicensable license to HHMI in their research articles. Pursuant to those licenses, the author-accepted manuscript of this article can be made freely available under a CC BY 4.0 license immediately upon publication.

AUTHOR CONTRIBUTIONS

This work was conceptualized by T.D.R., A.B.S., and E.E.E. Chromosome 1 visual alignment of the *NOTCH2NL* region between human and NHP and NHA *NOTCH2NL* SD analyses were done by D.Y., with additional visualization and genomic rearrangement analysis by F.A. Annotation of *NOTCH2NL* gene fusions in NHA and *NBPF* DUF1220 domain sequence analysis was performed by P.H., assisted by M.D. A subset of HPRC assemblies was re-run with Verkko by X.G. NucFreq validation of HPRC assemblies was processed by A.N.R. Human *NOTCH2NL* phylogeny was constructed and visualized by T.D.R. with support from P.C.D. Human SD and IGC analyses were performed by T.D.R., assisted by M.R.V. Dorsal forebrain organoid generation of HG02630 was performed by I.P. FiberFold TAD prediction analysis was done by D.D. NOTCH2NL protein expression cloning, tissue culture, and western blot experiments were done by G.J.M., O.G.P., and B.J.M. Genomic and transcriptomic data for this study were generated by K.M.M., K.H., and J.E.R. Data were processed by K.M.M., S.J.N., and A.E.S.-C. The manuscript was written by T.D.R., P.H., F.A., D.Y., I.P., D.D., B.J.M., A.B.S., and E.E.E. All additional analyses not specifically listed in this section were done by T.D.R. B.P., S.R.S., A.B.S., and E.E.E. advised on the project.

DECLARATION OF INTERESTS

E.E.E. is a scientific advisory board (SAB) member of Variant Bio, Inc. A.B.S. is a co-inventor on a patent relating to the Fiber-seq method (US17/995,058).

STAR★METHODS

Detailed methods are provided in the online version of this paper and include the following:

- KEY RESOURCES TABLE
- METHOD DETAILS
  - *NOTCH2NL*-CHM13 region identity matrix
  - *NOTCH2NL*-CHM13 region duplication barcodes
  - Visual alignment of *NOTCH2NL*-CHM13 regions
  - Chromosome 1 visual alignment of *NOTCH2NL* region between human and NHPs
  - NHA SD and *NOTCH2NL* analysis
  - Simulation test for enrichment of SVs and SDs at inversion breakpoints
  - *NOTCH2NL* NHA phylogeny and duplication timing
  - Annotation of *NOTCH2NL* gene fusions in NHA
  - NBPF DUF1220 domain sequence analysis
  - Assembly validation of *NOTCH2NL* regions in HPRC assemblies
  - Determining *NOTCH2NL* identity and uncovering IGC in HPRC assemblies
  - iPSC generation of HG02630
  - Making brain organoids of HG02630
  - Fiber-seq and identification of chromatin accessibility in HG02630 brain organoids
  - Long-read RNA-seq in HG02630 brain organoids
  - Protein expression of NOTCH2tv in HEK293 cells
  - Predicting TADs associated with *NOTCH2NL* paralogs using FiberFold

SUPPLEMENTAL INFORMATION

Supplemental information can be found online at https://doi.org/10.1016/j.xgen.2026.101194.

Received: March 15, 2025
Revised: November 6, 2025
Accepted: March 4, 2026
Published: March 30, 2026

REFERENCES

1. Fiddes, I.T., Lodewijk, G.A., Mooring, M., Bosworth, C.M., Ewing, A.D., Mantalas, G.L., Novak, A.M., van den Bout, A., Bishara, A., Rosenkrantz, J.L., et al. (2018). Human-Specific NOTCH2NL Genes Affect Notch Signaling and Cortical Neurogenesis. Cell *173*, 1356–1369.e22. https://doi.org/10.1016/j.cell.2018.03.051.
2. Suzuki, I.K., Gacquer, D., Van Heurck, R., Kumar, D., Wojno, M., Bilheu, A., Herpoel, A., Lambert, N., Cheron, J., Polleux, F., et al. (2018). Human-Specific NOTCH2NL Genes Expand Cortical Neurogenesis through Delta/Notch Regulation. Cell *173*, 1370–1384.e16. https://doi.org/10.1016/j.cell.2018.03.067.
3. Yoo, D., Rhie, A., Hebbar, P., Antonacci, F., Logsdon, G.A., Solar, S.J., Antipov, D., Pickett, B.D., Safonova, Y., Montinaro, F., et al. (2025). Complete sequencing of ape genomes. Nature *641*, 401–418. https://doi.org/10.1038/s41586-025-08816-3.
4. Florio, M., Heide, M., Pinson, A., Brandl, H., Albert, M., Winkler, S., Wimberger, P., Huttner, W.B., and Hiller, M. (2018). Evolution and cell-type specificity of human-specific genes preferentially expressed in progenitors of fetal neocortex. eLife *7*, e32332. https://doi.org/10.7554/eLife.32332.
5. Rajagopalan, R., Gilbert, M.A., McEldrew, D.A., Nassur, J.A., Loomes, K.M., Piccoli, D.A., Krantz, I.D., Conlin, L.K., and Spinner, N.B. (2021). Genome sequencing increases diagnostic yield in clinically diagnosed Alagille syndrome patients with previously negative test results. Genet. Med. *23*, 323–330. https://doi.org/10.1038/s41436-020-00989-8.
6. Li, Z.-D., Abuduxikuer, K., Wang, L., Hao, C.-Z., Zhang, J., Wang, M.-X., Li, L.-T., Qiu, Y.-L., Xie, X.-B., Lu, Y., and Wang, J.S. (2022). Defining pathogenicity of NOTCH2 variants for diagnosis of Alagille syndrome type 2 using a large cohort of patients. Liver Int. *42*, 1836–1848. https://doi.org/10.1111/liv.15292.

7. Ishiura, H., Shibata, S., Yoshimura, J., Suzuki, Y., Qu, W., Doi, K., Almansour, M.A., Kikuchi, J.K., Taira, M., Mitsui, J., et al. (2019). Noncoding CGG repeat expansions in neuronal intranuclear inclusion disease, oculopharyngodistal myopathy and an overlapping disease. Nat. Genet. *51*, 1222–1232. https://doi.org/10.1038/s41588-019-0458-z.

8. Sone, J., Mitsuhashi, S., Fujita, A., Mizuguchi, T., Hamanaka, K., Mori, K., Koike, H., Hashiguchi, A., Takashima, H., Sugiyama, H., et al. (2019). Long-read sequencing identifies GGC repeat expansions in NOTCH2NLC associated with neuronal intranuclear inclusion disease. Nat. Genet. *51*, 1215–1221. https://doi.org/10.1038/s41588-019-0459-y.

9. Klopocki, E., Schulze, H., Strauß, G., Ott, C.-E., Hall, J., Trotier, F., Fleischhauer, S., Greenhalgh, L., Newbury-Ecob, R.A., Neumann, L.M., et al. (2007). Complex Inheritance Pattern Resembling Autosomal Recessive Inheritance Involving a Microdeletion in Thrombocytopenia–Absent Radius Syndrome. Am. J. Hum. Genet. *80*, 232–240. https://doi.org/10.1086/510919.

10. Brunetti-Pierri, N., Berg, J.S., Scaglia, F., Belmont, J., Bacino, C.A., Sahoo, T., Lalani, S.R., Graham, B., Lee, B., Shinawi, M., et al. (2008). Recurrent reciprocal 1q21.1 deletions and duplications associated with microcephaly or macrocephaly and developmental and behavioral abnormalities. Nat. Genet. *40*, 1466–1471. https://doi.org/10.1038/ng.279.

11. Mefford, H.C., Sharp, A.J., Baker, C., Itsara, A., Jiang, Z., Buysse, K., Huang, S., Maloney, V.K., Crolla, J.A., Baralle, D., et al. (2008). Recurrent rearrangements of chromosome 1q21.1 and variable pediatric phenotypes. N. Engl. J. Med. *359*, 1685–1699. https://doi.org/10.1056/NEJMoa0805384.

12. Vandepoele, K., Van Roy, N., Staes, K., Speleman, F., and van Roy, F. (2005). A Novel Gene Family NBPF: Intricate Structure Generated by Gene Duplications During Primate Evolution. Mol. Biol. Evol. *22*, 2265–2274. https://doi.org/10.1093/molbev/msi222.

13. Jiang, Z., Tang, H., Ventura, M., Cardone, M.F., Marques-Bonet, T., She, X., Pevzner, P.A., and Eichler, E.E. (2007). Ancestral reconstruction of segmental duplications reveals punctuated cores of human genome evolution. Nat. Genet. *39*, 1361–1368. https://doi.org/10.1038/ng.2007.9.

14. Dumas, L., and Sikela, J.M. (2009). DUF1220 Domains, Cognitive Disease, and Human Brain Evolution. Cold Spring Harb. Symp. Quant. Biol. *74*, 375–382. https://doi.org/10.1101/sqb.2009.74.025.

15. O'Bleness, M.S., Dickens, C.M., Dumas, L.J., Kehrer-Sawatzki, H., Wyckoff, G.J., and Sikela, J.M. (2012). Evolutionary history and genome organization of DUF1220 protein domains. G3 (Bethesda). *2*, 977–986. https://doi.org/10.1534/g3.112.003061.

16. Fiddes, I.T., Pollen, A.A., Davis, J.M., and Sikela, J.M. (2019). Paired involvement of human-specific Olduvai domains and NOTCH2NL genes in human brain evolution. Hum. Genet. *138*, 715–721. https://doi.org/10.1007/s00439-019-02018-4.

17. Chen, J.-M., Cooper, D.N., Chuzhanova, N., Férec, C., and Patrinos, G.P. (2007). Gene conversion: mechanisms, evolution and human disease. Nat. Rev. Genet. *8*, 762–775. https://doi.org/10.1038/nrg2193.

18. Dunham, I., Kundaje, A., Aldred, S.F., Collins, P.J., Davis, C.A., Doyle, F., Epstein, C.B., Frietze, S., Harrow, J., Kaul, R., et al. (2012). An integrated encyclopedia of DNA elements in the human genome. Nature *489*, 57–74. https://doi.org/10.1038/nature11247.

19. GTEx Consortium; Laboratory Data Analysis &Coordinating Center LDACC—Analysis Working Group; Statistical Methods groups—Analysis Working Group; Enhancing GTEx eGTEx groups; NIH Common Fund; Jo, B., Mohammadi, P., Park, Y., Parsana, P., et al.; Biospecimen Collection Source Site—NDRI (2017). Genetic effects on gene expression across human tissues. Nature *550*, 204–213. https://doi.org/10.1038/nature24277.

20. Mao, Y., Harvey, W.T., Porubsky, D., Munson, K.M., Hoekzema, K., Lewis, A.P., Audano, P.A., Rozanski, A., Yang, X., Zhang, S., et al. (2024). Structurally divergent and recurrently mutated regions of primate genomes. Cell *187*, 1547–1562.e13. https://doi.org/10.1016/j.cell.2024.01.052.

21. Liao, W.-W., Asri, M., Ebler, J., Doerr, D., Haukness, M., Hickey, G., Lu, S., Lucas, J.K., Monlong, J., Abel, H.J., et al. (2023). A draft human pangenome reference. Nature *617*, 312–324. https://doi.org/10.1038/s41586-023-05896-x.

22. Nurk, S., Koren, S., Rhie, A., Rautiainen, M., Bzikadze, A.V., Mikheenko, A., Vollger, M.R., Altemose, N., Uralsky, L., Gershman, A., et al. (2022). The complete sequence of a human genome. Science *376*, 44–53. https://doi.org/10.1126/science.abj6987.

23. Dougherty, M.L., Underwood, J.G., Nelson, B.J., Tseng, E., Munson, K.M., Penn, O., Nowakowski, T.J., Pollen, A.A., and Eichler, E.E. (2018). Transcriptional fates of human-specific segmental duplications in brain. Genome Res. *28*, 1566–1576. https://doi.org/10.1101/gr.237610.118.

24. Jiang, Z., Hubley, R., Smit, A., and Eichler, E.E. (2008). DupMasker: A tool for annotating primate segmental duplications. Genome Res. *18*, 1362–1368. https://doi.org/10.1101/gr.078477.108.

25. Vollger, M.R., Dishuck, P.C., Harvey, W.T., DeWitt, W.S., Guitart, X., Goldberg, M.E., Rozanski, A.N., Lucas, J., Asri, M., et al.; Human Pangenome Reference Consortium (2023). Increased mutation and gene conversion within human segmental duplications. Nature *617*, 325–334. https://doi.org/10.1038/s41586-023-05895-y.

26. Patil, S.R., and Lubs, H.A. (1977). Classification of qh regions in human chromosomes 1, 9, and 16 by C-banding. Hum. Genet. *38*, 35–38. https://doi.org/10.1007/BF00295805.

27. Perez, G., Barber, G.P., Benet-Pages, A., Casper, J., Clawson, H., Diekhans, M., Fischer, C., Gonzalez, J.N., Hinrichs, A.S., Lee, C.M., et al. (2025). The UCSC Genome Browser database: 2025 update. Nucleic Acids Res. *53*, D1243–D1249. https://doi.org/10.1093/nar/gkae974.

28. Yunis, J.J., and Prakash, O. (1982). The Origin of Man: A Chromosomal Pictorial Legacy. Science *215*, 1525–1530. https://doi.org/10.1126/science.7063861.

29. Szamalek, J.M., Goidts, V., Cooper, D.N., Hameister, H., and Kehrer-Sawatzki, H. (2006). Characterization of the human lineage-specific pericentric inversion that distinguishes human chromosome 1 from the homologous chromosomes of the great apes. Hum. Genet. *120*, 126–138. https://doi.org/10.1007/s00439-006-0209-y.

30. Vollger, M.R., Guitart, X., Dishuck, P.C., Mercuri, L., Harvey, W.T., Gershman, A., Diekhans, M., Sulovari, A., Munson, K.M., Lewis, A.P., et al. (2022). Segmental duplications and their variation in a complete human genome. Science *376*, eabj6965. https://doi.org/10.1126/science.abj6965.

31. Buenrostro, J.D., Giresi, P.G., Zaba, L.C., Chang, H.Y., and Greenleaf, W.J. (2013). Transposition of native chromatin for multimodal regulatory analysis and personal epigenomics. Nat. Methods *10*, 1213–1218. https://doi.org/10.1038/nmeth.2688.

32. Thurman, R.E., Rynes, E., Humbert, R., Vierstra, J., Maurano, M.T., Haugen, E., Sheffield, N.C., Stergachis, A.B., Wang, H., Vernot, B., et al. (2012). The accessible chromatin landscape of the human genome. Nature *489*, 75–82. https://doi.org/10.1038/nature11232.

33. Stergachis, A.B., Debo, B.M., Haugen, E., Churchman, L.S., and Stamatoyannopoulos, J.A. (2020). Single-molecule regulatory architectures captured by chromatin fiber sequencing. Science *368*, 1449–1454. https://doi.org/10.1126/science.aaz1646.

34. Vollger, M.R., Swanson, E.G., Neph, S.J., Ranchalis, J., Munson, K.M., Ho, C.-H., Sedeño-Cortés, A.E., Fondrie, W.E., Bohaczuk, S.C., Mao, Y., et al. (2024). A haplotype-resolved view of human gene regulation. Preprint at bioRxiv. https://doi.org/10.1101/2024.06.14.599122.

35. Dubocanin, D., Cortes, A.E.S., Ranchalis, J., Real, T., Mallory, B., and Stergachis, A.B. (2022). Single-molecule architecture and heterogeneity of human telomeric DNA and chromatin. Preprint at bioRxiv. https://doi.org/10.1101/2022.05.09.491186.

36. Dubocanin, D., Kalygina, A., Franklin, J.M., Chittenden, C., Vollger, M.R., Neph, S., Stergachis, A.B., and Altemose, N. (2025). Integrating Single-Molecule Sequencing and Deep Learning to Predict Haplotype-Specific 3D Chromatin Organization in a Mendelian Condition. Preprint at bioRxiv. https://doi.org/10.1101/2025.02.26.640261.

37. Marques-Bonet, T., Girirajan, S., and Eichler, E.E. (2009). The origins and impact of primate segmental duplications. Trends Genet. *25*, 443–454. https://doi.org/10.1016/j.tig.2009.08.002.

38. Charrier, C., Joshi, K., Coutinho-Budd, J., Kim, J.-E., Lambert, N., de Marchena, J., Jin, W.-L., Vanderhaeghen, P., Ghosh, A., Sassa, T., and Polleux, F. (2012). Inhibition of SRGAP2 function by its human-specific paralogs induces neoteny during spine maturation. Cell *149*, 923–935. https://doi.org/10.1016/j.cell.2012.03.034.

39. Schmidt, E.R.E., Kupferman, J.V., Stackmann, M., and Polleux, F. (2019). The human-specific paralogs SRGAP2B and SRGAP2C differentially modulate SRGAP2A-dependent synaptic development. Sci. Rep. *9*, 18692. https://doi.org/10.1038/s41598-019-54887-4.

40. Ju, X.-C., Hou, Q.-Q., Sheng, A.-L., Wu, K.-Y., Zhou, Y., Jin, Y., Wen, T., Yang, Z., Wang, X., and Luo, Z.-G. (2016). The hominoid-specific gene TBC1D3 promotes generation of basal neural progenitors and induces cortical folding in mice. eLife *5*, e18197. https://doi.org/10.7554/eLife.18197.

41. Florio, M., Albert, M., Taverna, E., Namba, T., Brandl, H., Lewitus, E., Haffner, C., Sykes, A., Wong, F.K., Peters, J., et al. (2015). Human-specific gene ARHGAP11B promotes basal progenitor amplification and neocortex expansion. Science *347*, 1465–1470. https://doi.org/10.1126/science.aaa1975.

42. Fischer, J., Fernández Ortuño, E., Marsoner, F., Artioli, A., Peters, J., Namba, T., Eugster Oegema, C., Huttner, W.B., Ladewig, J., and Heide, M. (2022). Human-specific ARHGAP11B ensures human-like basal progenitor levels in hominid cerebral organoids. EMBO Rep. *23*, e54728. https://doi.org/10.15252/embr.202254728.

43. Van Heurck, R., Bonnefont, J., Wojno, M., Suzuki, I.K., Velez-Bravo, F.D., Erkol, E., Nguyen, D.T., Herpoel, A., Bilheu, A., Beckers, S., et al. (2023). CROCCP2 acts as a human-specific modifier of cilia dynamics and mTOR signaling to promote expansion of cortical progenitors. Neuron *111*, 65–80.e6. https://doi.org/10.1016/j.neuron.2022.10.018.

44. Dennis, M.Y., Harshman, L., Nelson, B.J., Penn, O., Cantsilieris, S., Huddleston, J., Antonacci, F., Penewit, K., Denman, L., Raja, A., et al. (2017). The evolution and population diversity of human-specific segmental duplications. Nat. Ecol. Evol. *1*, 0069. https://doi.org/10.1038/s41559-016-0069.

45. Guitart, X., Porubsky, D., Yoo, D., Dougherty, M.L., Dishuck, P.C., Munson, K.M., Lewis, A.P., Hoekzema, K., Knuth, J., Chang, S., et al. (2024). Independent expansion, selection, and hypervariability of the TBC1D3 gene family in humans. Genome Res. *34*, 1798–1810. https://doi.org/10.1101/gr.279299.124.

46. Giannuzzi, G., Siswara, P., Malig, M., Marques-Bonet, T., NISC Comparative Sequencing Program; Mullikin, J.C., Ventura, M., and Eichler, E.E. (2013). Evolutionary dynamism of the primate LRRC37 gene family. Genome Res. *23*, 46–59. https://doi.org/10.1101/gr.138842.112.

47. Cantsilieris, S., Sunkin, S.M., Johnson, M.E., Anaclerio, F., Huddleston, J., Baker, C., Dougherty, M.L., Underwood, J.G., Sulovari, A., Hsieh, P., et al. (2020). An evolutionary driver of interspersed segmental duplications in primates. Genome Biol. *21*, 202. https://doi.org/10.1186/s13059-020-02074-4.

48. Dishuck, P.C., Munson, K.M., Lewis, A.P., Dougherty, M.L., Underwood, J.G., Harvey, W.T., Hsieh, P., Pastinen, T., and Eichler, E.E. (2025). Structural variation, selection, and diversification of the NPIP gene family from the human pangenome. Cell Genom. *5*, 100977. https://doi.org/10.1016/j.xgen.2025.100977.

49. Dennis, M.Y., Nuttle, X., Sudmant, P.H., Antonacci, F., Graves, T.A., Nefedov, M., Rosenfeld, J.A., Sajjadian, S., Malig, M., Kotkiewicz, H., et al. (2012). Evolution of Human-Specific Neural SRGAP2 Genes by Incomplete Segmental Duplication. Cell *149*, 912–922. https://doi.org/10.1016/j.cell.2012.03.033.

50. Tattersall, I. (2023). Endocranial volumes and human evolution. F1000Res. *12*, 565. https://doi.org/10.12688/f1000research.131636.1.

51. Ohno, S., Wolf, U., and Atkin, N.B. (1968). Evolution from fish to mammals by gene duplication. Hereditas *59*, 169–187. https://doi.org/10.1111/j.1601-5223.1968.tb02169.x.

52. Sasaki, M., Lange, J., and Keeney, S. (2010). Genome destabilization by homologous recombination in the germ line. Nat. Rev. Mol. Cell Biol. *11*, 182–195. https://doi.org/10.1038/nrm2849.

53. Cooper, G.M., Coe, B.P., Girirajan, S., Rosenfeld, J.A., Vu, T.H., Baker, C., Williams, C., Stalker, H., Hamid, R., Hannig, V., et al. (2011). A copy number variation morbidity map of developmental delay. Nat. Genet. *43*, 838–846. https://doi.org/10.1038/ng.909.

54. Marques-Bonet, T., and Eichler, E.E. (2009). The Evolution of Human Segmental Duplications and the Core Duplicon Hypothesis. Cold Spring Harb. Symp. Quant. Biol. *74*, 355–362. https://doi.org/10.1101/sqb.2009.74.011.

55. Itsara, A., Wu, H., Smith, J.D., Nickerson, D.A., Romieu, I., London, S.J., and Eichler, E.E. (2010). De novo rates and selection of large copy number variation. Genome Res. *20*, 1469–1481. https://doi.org/10.1101/gr.107680.110.

56. Reams, A.B., Kofoid, E., Savageau, M., and Roth, J.R. (2010). Duplication Frequency in a Population of Salmonella enterica Rapidly Approaches Steady State With or Without Recombination. Genetics *184*, 1077–1094. https://doi.org/10.1534/genetics.109.111963.

57. Langley, C.H., Stevens, K., Cardeno, C., Lee, Y.C.G., Schrider, D.R., Pool, J.E., Langley, S.A., Suarez, C., Corbett-Detig, R.B., Kolaczkowski, B., et al. (2012). Genomic Variation in Natural Populations of Drosophila melanogaster. Genetics *192*, 533–598. https://doi.org/10.1534/genetics.112.142018.

58. Nuttle, X., Giannuzzi, G., Duyzend, M.H., Schraiber, J.G., Narvaiza, I., Sudmant, P.H., Penn, O., Chiatante, G., Malig, M., Huddleston, J., et al. (2016). Emergence of a Homo sapiens-specific gene family and chromosome 16p11.2 CNV susceptibility. Nature *536*, 205–209. https://doi.org/10.1038/nature19075.

59. Makova, K.D., Pickett, B.D., Harris, R.S., Hartley, G.A., Cechova, M., Pal, K., Nurk, S., Yoo, D., Li, Q., Hebbar, P., et al. (2024). The complete sequence and comparative analysis of ape sex chromosomes. Nature *630*, 401–411. https://doi.org/10.1038/s41586-024-07473-2.

60. Li, H. (2018). Minimap2: pairwise alignment for nucleotide sequences. Bioinformatics *34*, 3094–3100. https://doi.org/10.1093/bioinformatics/bty191.

61. Porubsky, D., Guitart, X., Yoo, D., Dishuck, P.C., Harvey, W.T., and Eichler, E.E. (2025). SVbyEye: A visual tool to characterize structural variation among whole-genome assemblies. Bioinforma. Oxf. Engl. *41*, btaf332. https://doi.org/10.1093/bioinformatics/btaf332.

62. Numanagic, I., Gökkaya, A.S., Zhang, L., Berger, B., Alkan, C., and Hach, F. (2018). Fast characterization of segmental duplications in genome assemblies. Bioinforma. Oxf. Engl. *34*, i706–i714. https://doi.org/10.1093/bioinformatics/bty586.

63. Benson, G. (1999). Tandem repeats finder: a program to analyze DNA sequences. Nucleic Acids Res. *27*, 573–580. https://doi.org/10.1093/nar/27.2.573.

64. Tempel, S. (2012). Using and understanding RepeatMasker. Methods Mol. Biol. *859*, 29–51. https://doi.org/10.1007/978-1-61779-603-6_2.

65. Morgulis, A., Gertz, E.M., Schäffer, A.A., and Agarwala, R. (2006). WindowMasker: window-based masker for sequenced genomes. Bioinforma. Oxf. Engl *22*, 134–141. https://doi.org/10.1093/bioinformatics/bti774.

66. Goel, M., Sun, H., Jiao, W.-B., and Schneeberger, K. (2019). SyRI: finding genomic rearrangements and local sequence differences from whole-genome assemblies. Genome Biol. *20*, 277. https://doi.org/10.1186/s13059-019-1911-0.

67. Ebert, P., Audano, P.A., Zhu, Q., Rodriguez-Martin, B., Porubsky, D., Bonder, M.J., Sulovari, A., Ebler, J., Zhou, W., Serra Mari, R., et al. (2021). Haplotype-resolved diverse human genomes and integrated analysis of structural variation. Science *372*, eabf7117. https://doi.org/10.1126/science.abf7117.

68. Katoh, K., and Standley, D.M. (2013). MAFFT Multiple Sequence Alignment Software Version 7: Improvements in Performance and Usability. Mol. Biol. Evol. *30*, 772–780. https://doi.org/10.1093/molbev/mst010.

69. Hoang, D.T., Chernomor, O., von Haeseler, A., Minh, B.Q., and Vinh, L.S. (2018). UFBoot2: Improving the Ultrafast Bootstrap Approximation. Mol. Biol. Evol. *35*, 518–522. https://doi.org/10.1093/molbev/msx281.

70. Minh, B.Q., Schmidt, H.A., Chernomor, O., Schrempf, D., Woodhams, M.D., von Haeseler, A., and Lanfear, R. (2020). IQ-TREE 2: New Models and Efficient Methods for Phylogenetic Inference in the Genomic Era. Mol. Biol. Evol. *37*, 1530–1534. https://doi.org/10.1093/molbev/msaa015.

71. Sievers, F., and Higgins, D.G. (2018). Clustal Omega for making accurate alignments of many protein sequences. Protein Sci. *27*, 135–145. https://doi.org/10.1002/pro.3290.

72. Blum, M., Andreeva, A., Florentino, L.C., Chuguransky, S.R., Grego, T., Hobbs, E., Pinto, B.L., Orr, A., Paysan-Lafosse, T., Ponamareva, I., et al. (2025). InterPro: the protein sequence classification resource in 2025. Nucleic Acids Res. *53*, D444–D456. https://doi.org/10.1093/nar/gkae1082.

73. Pendleton, A.L., Shen, F., Taravella, A.M., Emery, S., Veeramah, K.R., Boyko, A.R., and Kidd, J.M. (2018). Comparison of village dog and wolf genomes highlights the role of the neural crest in dog domestication. BMC Biol. *16*, 64. https://doi.org/10.1186/s12915-018-0535-2.

74. Rautiainen, M., Nurk, S., Walenz, B.P., Logsdon, G.A., Porubsky, D., Rhie, A., Eichler, E.E., Phillippy, A.M., and Koren, S. (2023). Telomere-to-telomere assembly of diploid chromosomes with Verkko. Nat. Biotechnol. *41*, 1474–1482. https://doi.org/10.1038/s41587-023-01662-6.

75. Vollger, M.R., Korlach, J., Eldred, K.C., Swanson, E., Underwood, J.G., Bohaczuk, S.C., Mao, Y., Cheng, Y.-H.H., Ranchalis, J., Blue, E.E., et al. (2025). Synchronized long-read genome, methylome, epigenome and transcriptome profiling resolve a Mendelian condition. Nat. Genet. *57*, 469–479. https://doi.org/10.1038/s41588-024-02067-0.

76. Seiler, S.T., Mantalas, G.L., Selberg, J., Cordero, S., Torres-Montoya, S., Baudin, P.V., Ly, V.T., Amend, F., Tran, L., Hoffman, R.N., et al. (2022). Modular automated microfluidic cell culture platform reduces glycolytic stress in cerebral cortex organoids. Sci. Rep. *12*, 20173. https://doi.org/10.1038/s41598-022-20096-9.

77. Al'Khafaji, A.M., Smith, J.T., Garimella, K.V., Babadi, M., Popic, V., Sade-Feldman, M., Gatzen, M., Sarkizova, S., Schwartz, M.A., Blaum, E.M., et al. (2024). High-throughput RNA isoform sequencing using programmed cDNA concatenation. Nat. Biotechnol. *42*, 582–586. https://doi.org/10.1038/s41587-023-01815-7.

STAR★METHODS

KEY RESOURCES TABLE

REAGENT or RESOURCE	SOURCE	IDENTIFIER
Antibodies		
HA-Tag (C29F4) Rabbit mAb #3724	Cell Signaling Technology	Cat#3724T; RRID:AB_1549585
GFP (D5.1) Rabbit mAb #2956	Cell Signaling Technology	Cat#2956T; RRID:AB_3713179
IRDye 800CW Donkey anti-Rabbit IgG Secondary Ab	LI-COR Biosciences	P/N: 925–32213; RRID AB_2715510
Chemicals, peptides, and recombinant proteins		
Hia5 MTase	Stergachis et al.[34]	N/A
Critical commercial assays		
PacBio MAS-seq Kinnex kit	PacBio	PN: 103-072-000
Gibson Assembly Cloning kit	NEB	Cat#E5510S
High Efficiency Transformation Protocol	NEB	Cat#C2987H
Monarch Plasmid Miniprep kit	NEB	Cat#T10104
Deposited data		
NHA (multispecies) Iso-Seq transcript data from testis	Makova et al.[59]	BioProject ID: PRJNA902025
NHA (multispecies) Iso-Seq transcript data from fibroblast and lymphoblastoid cells	NHGRI	BioProject ID: PRJNA1016395
Chimpanzee Iso-Seq transcript data from iPSCs	Alex Pollen, unpublished	N/A
Chimpanzee Iso-Seq transcript data from neuroepithelium	Alex Pollen, unpublished	N/A
Bonobo Iso-Seq transcript data from neural progenitor cells	Alex Pollen, unpublished	N/A
HG02630 Iso-Seq transcript data from dorsal forebrain organoids	This paper	BioProject ID: PRJNA1236375
HG02630 Fiber-seq sequencing data from dorsal forebrain organoids	This paper	BioProject ID: PRJNA1236375
CHM13 Fiber-seq sequencing data from fibroblast	Dubocanin et al.[35]	NCBI GEO: GSE186009
Experimental models: Cell lines		
HG02630 cultured lymphoblastoids	Coriell	N/A
HG02630 cultured iPSCs	This paper	N/A
HG02630 dorsal forebrain organoids	This paper	N/A
Oligonucleotides		
NOTCH2NL gBlocks for protein expression (Refer to Table S5)	IDT	N/A
Recombinant DNA		
pEF-HA-NOTCH2NLB-IRES-GFP	This paper	https://zenodo.org/records/15022214
pEF-HA-NOTCH2NLR-IRES-GFP	This paper	https://zenodo.org/records/15022214
pEF-HA-NOTCH2tv-IRES-GFP	This paper	https://zenodo.org/records/15022214
Software and algorithms		
Minimap2	Li[60]	https://github.com/lh3/minimap2
DupMasker	Jiang et al.[24]	https://www.repeatmasker.org/DupMaskerDownload.html
SVbyEye	Porubsky et al.[61]	https://github.com/daewoooo/SVbyEye
SEDEF	Numanagic et al.[62]	https://github.com/vpc-ccg/sedef
TRF	Benson[63]	https://github.com/Benson-Genomics-Lab/TRF
RepeatMasker	Tempel[64]	https://www.repeatmasker.org/RepeatMasker/
WindowMasker	Morgulis et al.[65]	https://github.com/goeckslab/WindowMasker

(*Continued on next page*)

CellPress
OPEN ACCESS

Continued

REAGENT or RESOURCE	SOURCE	IDENTIFIER
Syri	Goel et al.[66]	https://github.com/schneebergerlab/syri
PAV	Ebert et al.[67]	https://github.com/EichlerLab/pav
MAFFT	Katoh and Standley[68]	https://mafft.cbrc.jp/alignment/software/
IQ-TREE2 w/Ultrafast Bootstrap	Hoang et al.,[69] Minh et al.[70]	https://iqtree.github.io/#download
Clustal Omega	Sievers and Higgins[71]	http://www.clustal.org/omega/
InterPro	Blum et al.[72]	https://www.ebi.ac.uk/interpro/
rustybam	Mitchell Vollger	https://github.com/mrvollger/rustybam
NucFreq	Vollger et al.[30]	https://github.com/mrvollger/NucFreq
fastCN	Pendleton et al.[73]	https://github.com/KiddLab/fastCN
Verkko	Rautiainen et al.[74]	https://github.com/marbl/verkko
fiberseq-FIRE pipeline	Vollger et al.,[34] Vollger et al.[75]	https://github.com/fiberseq/fiberseq-fire
pbmm2	PacBio	https://github.com/PacificBiosciences/pbmm2
Iso-Seq	PacBio	https://isoseq.how/
FiberFold	Dubocanin et al.[36]	https://github.com/altemoselab/fiberFold

METHOD DETAILS

NOTCH2NL-CHM13 region identity matrix

NOTCH2NL paralog sequences from T2T-CHM13 V2.0[27] (http://genome.ucsc.edu) plus 1 Mbp surrounding each gene were all aligned to each other simultaneously and allowing for secondary alignments (paralog pairs with less than 1 Mbp between them had overlapping sequence removed), using minimap2[60] and the parameters

*-x asm*20 *-s* 1000 *--eqx -a --secondary* = *yes -p* 0.05

The longest nonoverlapping stretch of syntenic sequence that can be aligned between each paralog pair is represented in the matrix. The percent identity shared between each paralog pair is calculated by the number of base matches.

NOTCH2NL-CHM13 region duplication barcodes

The duplicon locus barcoding is a computational approach that can readily be used to assess the content and organization of the SDs for any given paralogous locus. It takes advantage of the fact that all SDs can be decomposed into smaller evolutionary units that have accumulated into larger higher-order structures based on their juxtaposition and accumulation over time.[13] Based on this repeat graph annotation, the tool DupMasker[24] will encode any SD region into these evolutionary subunits—the orientation and juxtaposition of each becomes a unique identifier that can be color-coded to create a visual track of the pattern of duplications in a given region. Even though *NOTCH2NL* paralogs are in SD regions that share high identity, the accumulation pattern is effectively unique allowing assignment to specific copies based on the majority-rule consensus for this DupMasker "barcode".

In this study *NOTCH2NL* paralog sequences plus 1 Mbp surrounding each gene were used as input for DupMasker. The.duplicons output file was processed into a.txt file to create visual tracks of duplicon barcodes for each region.

Visual alignment of *NOTCH2NL*-CHM13 regions

NOTCH2NL paralog sequences plus 1 Mbp surrounding each gene were aligned to each other one by one and allowed for secondary alignments, using minimap2 parameters

*-x asm*20 *-s* 1000 *--eqx -c --secondary* = *yes*

The output of each gene pair alignment was compiled into a single output.paf file that was used as input for SVbyEye.[61] The ladder plot alignment figure was visualized using the plotAVA function. Each *NOTCH2NL* region's corresponding duplication barcodes are included as an additional annotation track. The output.paf CIGAR string was also used to return a table of insertion/deletion SVs for each paralog.

Chromosome 1 visual alignment of *NOTCH2NL* region between human and NHPs

NHP assemblies used in this study from previous research can be found by the following GenBank accession IDs[3,20]: GCA_028858775.2 (chimpanzee), GCA_029289425.2 (bonobo), GCA_029281585.2 (gorilla), GCA_028885625.2 and GCA_028885655.2 (Bornean and

Sumatran orangutans) and GCA_030222085.1 (macaque). Homologous sequence of the *NOTCH2NL* region in human (chr1:110,000,000–160,00,000) was identified by aligning human sequence against each of the NHP genomes. The alignment was performed using minimap2 with the parameters

$$-x\ asm20\ -c\ --eqx\ --secondary = no$$

Alignment blocks equal to or larger than 10 kbp were retained. After locating the corresponding sequences of NHPs, the alignment was performed allowing for secondary alignment using the parameter

$$-x\ asm20\ -c\ --eqx\ --secondary = yes$$

progressively in the order from human to macaque. The alignment was visualized using SVbyEye. Similar alignments between T2T-CHM13 and the previous reference genome, GRCh38, were performed using the same command (Figure S6).

NHA SD and *NOTCH2NL* analysis

SD tracks generated by Yoo et al. 2025[3] were used. Briefly, the SD track was annotated using SEDEF (v1.1),[62] after masking the repeats using TRF (v4.1.0),[63] RepeatMasker (v4.1.5),[64] and WindowMasker (v2.2.22).[65] The SDs were filtered for length >1 kbp, pairwise sequence identity >90%, and satellite content <70%.

Simulation test for enrichment of SVs and SDs at inversion breakpoints

Coordinates of inversions and SVs identified by a previous study were utilized,[3] which used Syri (v1.6.3)[66] and PAV (v2.3.2)[67] pipelines. We first quantified SVs/SDs at the inversion breakpoints found within 110–160 Mbp regions by intersecting 100 kbp of inversion coordinates with SVs/SDs to get observed bp of SVs/SDs at the inversion breakpoints. To test enrichment of variants at the inversion breakpoints, we generated random distribution of SVs/SDs by shuffling the breakpoints, while restricting the region to 110–160 Mbp, and excluding centromere satellites. The null statistics distribution was generated by quantifying the SVs/SDs at the randomly shuffled regions. The one-sided simulation p-value was computed by quantifying the proportion of null distribution that show more extreme statistics (greater than observed SVs/SDs).

NOTCH2NL NHA phylogeny and duplication timing

Multiple intronic sequences of *NOTCH2* were mapped to each NHA primate assembly using minimap2. The coordinates of all the *NOTCH2NL*-like regions found were used to pull out the sequence and construct a multiple sequence alignment (MSA) using MAFFT[68] with the parameters

$$--anysymbol\ --reorder\ --maxiterate\ 1000\ --thread\ 16$$

The MSA was then processed with IQ-TREE2, an ML phylogeny building program, and bootstrapped using the ultrafast bootstrap.[69,70] IQTREE estimated phylogenetic dating using LSD2 to build a time tree based on orangutan–human divergence time being 15.2 MYA, gorilla–human divergence time being 8.6 MYA, and chimpanzee–human divergence being 6.4 MYA. IQ-TREE2 was run with the parameters

$$--date\ \{divergencetime\_file\}\ --keep\text{-}ident\ --date\text{-}tip\ 0\ --date\text{-}ci\ 100\ \text{-}B\ 1000\ \text{-}T\ 36$$

The most robustly bootstrapped tree was used in the main text.

Annotation of *NOTCH2NL* gene fusions in NHA

NCBI RefSeq and CAT2.0. annotations from the T2T NHA genomes were used to study copies of *NOTCH2NL*-like genes across the NHA assemblies. First, the genomic annotations for any gene from the *NOTCH2NL* family were extracted from both these annotation sets. Next, any copies that were missed by either of the two methods were extracted with BLAT, using the genomic sequence of the first four exons of *NOTCH2* on the T2T NHA genomes. The full set of copies were then manually investigated on the UCSC Genome Browser with the added NHA Iso-Seq transcript data from testis, fibroblast/lymphoblastoid cell lines (available under NCBI BioProject IDs: PRJNA902025 and PRJNA1016395), as well as iPSCs, neuroepithelium, and neural progenitor cells (Pollen Lab, unpublished). The exact boundaries of the genes and fusions were determined. Blat was used to describe the exon structures of these fused genes. The predicted proteins for these copies were aligned using Clustal Omega[71] and visualized using the NCBI Multiple Sequence Alignment Viewer (1.25.3).

NBPF DUF1220 domain sequence analysis

The NBPF protein sequence for all the gene copies downstream of *NOTCH2NL* in human and NHA were extracted and InterPro[72] was run to annotate all protein domains on the sequences. DUF1220 domains were then extracted and matched against the canonical sequences (UniProt) for HLS1, HLS2, HLS3, CON1, CON2, and CON3 using BLAT.

Assembly validation of *NOTCH2NL* regions in HPRC assemblies

Except for T2T-CHM13, the human assemblies used in this study were originally released as a part of HPRC year 1[21] without complete validation of every region. These assemblies are available under BioProject ID: PRJNA730822. To validate our set of assemblies, we first confirmed the correct assembly of contiguous sequence between *NOTCH2NL* paralogs on the same chromosome arms. This was done by evaluating the number of copies in each assembly and how many contigs they covered using rustybam (mrvollger.github.io/rustybam/). Assemblies with copies across three or more contigs were removed and those with no more than two contigs (allowing for gaps across the centromere) were assessed with NucFreq.[30] Assemblies with collapses (excess of secondary bases in a single haplotype assembly) within *NOTCH2NL*, collapses greater than 10 kbp outside *NOTCH2NL*, gaps (low-quality, N-based sequence) within *NOTCH2NL*, and gaps greater than 10 kbp outside *NOTCH2NL* over the 1 Mbp region surrounding each *NOTCH2NL* paralog were considered incorrectly assembled. Finally, assemblies were also removed from the completed set if they were missing specific unique gene marks outside *NOTCH2NL* SD regions, which are used as anchors for rearrangement boundaries. Deletions were further validated using fastCN[73] read depth. A subset of incorrectly assembled samples was attempted to be rescued using Verkko (1.1 and 1.2)[74] and added to the validated set: 69/94 haploid assemblies in total were correctly assembled and validated.

Determining *NOTCH2NL* identity and uncovering IGC in HPRC assemblies

To determine *NOTCH2NL* identity we took a three-pronged approach: (1) identifying which T2T-CHM13 *NOTCH2NL* CDS reference or ‘transcript’ best matches the *NOTCH2NL* sequence being queried using BLAT; (2) identifying the phylogenetic clade the *NOTCH2NL* sequence being queried best groups with—sequences from intron 2 of *NOTCH2/NL* from all samples queried were used to construct an MSA using MAFFT, from which an ML phylogeny was built using IQ-TREE2; and (3) identifying the mapping location of the *NOTCH2NL* sequence being queried using the greater *NOTCH2NL* region DupMasker barcode—*NOTCH2NL* intron 2 sequences plus 1 Mbp surrounding each intron were used as input for DupMasker and duplicon barcodes for each sequence were added to the tree. IGC events were first found through conflicts between the coding sequence identity, clade grouping, and/or mapping location. A final visualization of protein-coding changes caused by gene conversion can be confirmed using MSA software.

iPSC generation of HG02630

iPSCs were generated from HG02630 cultured lymphoblastoid cells obtained from Coriell using the method described in Vollger et al. (2025)[75]: StemFlex media (Thermo Fisher Scientific, A3349401) on Matrigel (Corning, CLS354277)-coated plates at 37°C in 5% CO_2 was used to maintain iPSCs and ReLeSR (STEMCELL Technologies, 100–0484) was used for passage of cell colonies.

Making brain organoids of HG02630

HG02630 iPSC line maintenance and cerebral organoid generation until day 21 were done using the methods described in Seiler et al. (2022).[76] To achieve uniform basal ECM coating on days 6 and 7, a combination of 0.2% alginate and 0.6mg/mL Geltrex LDEV-Free Reduced Growth Factor Basement Membrane Matrix (ThermoFisher) coating was used (Hoffman et al., in preparation), and the alginate was crosslinked with CaCl2.

On day 21, the organoids were broken apart using Trypsin-EDTA (0.25%), placed in the centrifuge at 250g for 5 min, resuspended in 0.5 mL of PBS, and centrifuged for 5 more minutes at 250g. The supernatant was aspirated, and the cells were resuspended in 180 μl of Buffer A (components missing). The sample was transferred to a PCR tube, and 180 μl of 2× lysis buffer was added. Cells were spun at 350g for 5 min, after which the supernatant was removed. The remaining nuclei pellets were resuspended in (Buffer A, 32mM SAM, Hia4 (200U/ul)) at 25°C for 10 min. Finally, 9 μl of 1% SDS was added to the sample and transferred to 1.5mL tubes using wide-bore pipette tips.

Fiber-seq and identification of chromatin accessibility in HG02630 brain organoids

PacBio HiFi Fiber-seq data were generated from HG02630 brain organoid nuclei pellets treated with Hia5 enzyme using the method described in Vollger et al. 2025.[75] The data were analyzed using the fiberseq-FIRE pipeline[34,75] (https://github.com/fiberseq/fiberseq-fire), which identifies single-molecule sites of chromatin actuation as well as peaks of chromatin actuation with a false discovery rate 5% threshold. Percent actuation was calculated as the percentage of fibers mapping to a given location that were classified as having a Fiber-seq Inferred Regulatory Element (FIRE).

For proof-of-concept unique mapping at this locus before HG02630 data was generated, previously published T2T-CHM13 Fiber-seq data was mapped to the NOTCH2NL region (Figure S8).

Long-read RNA-seq in HG02630 brain organoids

PacBio MAS-seq[77] (PN: 103-072-000) data were generated from HG02630 brain organoid nuclei pellets using the method described in the RNA preparation section of the STAR Methods in Vollger et al. (2025).[75] The data were processed and mapped to the HG02630 diploid assembly using pbmm2 (https://github.com/PacificBiosciences/pbmm2) and isoforms were defined using the Iso-Seq pipeline (https://isoseq.how/) and annotated using Pigeon.

Protein expression of NOTCH2tv in HEK293 cells

gBlocks were designed to contain an HA tag, *NOTCH2NL* CDS, IRES sequence, and *E-GFP* CDS (Table S5) and ordered using IDT (https://www.idtdna.com/page). Gibson Assembly (NEB, E5510) was used to clone constructs into a pEF-GFP vector. Each vector construct was cloned using NEB High Efficiency Transformation Protocol (C2987H/C2987I) with NEB 5-alpha Competent E. coli. Colonies were picked, inoculated, and plasmid DNA was extracted using the Monarch Plasmid Miniprep kit (NEB, T10104).

Four wells of a 6-well plate were seeded with $6.25 \times 10^{\hat{}5}$ HEK293 cells and grown in DMEM (Gibco) supplemented with 10% FBS and 1% Pen-Strep at 37°C in a humidified incubator with 5% CO_2. 24 h after seeding, cells were transiently transfected with 2.5 μg of the *NOTCH2tv*, *NOTCH2NLR, NOTCH2NLB*, or pEF-GFP (for GFP Ab control) expression plasmid construct using a 3:1 μL/μg ratio of Lipofectamine LTX Reagent (ThermoFisher) according to the manufacturer's protocol. Cells were harvested 48 h after transfection, washed in 1mL of cold PBS, resuspended in 250 μl of cold RIPA buffer (5M NaCl, 1M Tris-HCl pH 8.0, 1% NP40, 10% sodium deoxycholate, 10% SDS, 1mM PMSF, 1× Protease Inhibitor tablet (Pierce)) and incubated in a thermomixer at 4°C and 500 rpm for 20 min. The lysis was then spun down at 16,000 rpm for 20 min and the supernatant collected. 12μL of the cleared lysate was supplemented with 4μL of 4× LDS Sample Buffer (Invitrogen) and boiled at 70°C for 10 min. A 4–12% Bis-Tris gel (Invitrogen) was loaded with 15μL of each sample in duplicate and run in MOPS buffer at 200V for 50 min. The gels were then transferred onto a 0.45 μm nitrocellulose membrane (Bio-Rad) using a genie transfer apparatus (Idea Scientific) at 12V for 90 min. The membrane was incubated in blocking buffer (5% milk in TBST) at room temperature for 1 h before cutting the membrane in half and incubating with a 1:1000 dilution of either primary anti-HA (Cell Signaling Technology, 3724T) or primary anti-GFP (Cell Signaling Technology, 29565) in blocking buffer and incubated at 4°C overnight. The following day membranes were washed 3× with 10mL of blocking buffer followed by a 1-h incubation in a 1:20,000 dilution of IRDye 800CW secondary Ab (LI-COR, 926–32213) in blocking buffer. The membrane was washed 3× in TBST and imaged on an Odyssey imaging system (LI-COR) (Figure S12).

Predicting TADs associated with *NOTCH2NL* paralogs using FiberFold

To generate predicted contact maps surrounding *NOTCH2/NL* promoters, we used the recently released tool FiberFold.[36] We first collapsed single-molecule Fiber-seq data into one-dimensional bigWig tracks. These single-base-pair resolution bigWigs, which represent FIRE density, CpG methylation state, and CTCF footprinting state, were generated following the protocol described at https://github.com/altemoselab/fiberFold. Phased Fiber-seq data were used to produce haplotype-specific bigWigs, and all maps were oriented to maintain consistent directionality relative to the *NOTCH2* promoter. To compute mean squared error (MSE) plots, we calculated the element-wise difference between the two contact maps and squared the resulting values to generate the distribution of differential contacts between the loci.

Cell Genomics, Volume *6*

Supplemental information

Genetic diversity and regulatory features of human-specific *NOTCH2NL* duplications

Taylor D. Real, Prajna Hebbar, DongAhn Yoo, Francesca Antonacci, Ivana Pačar, Danilo Dubocanin, Mark Diekhans, Gregory J. Mikol, Oyeronke G. Popoola, Benjamin J. Mallory, Mitchell R. Vollger, Philip C. Dishuck, Xavi Guitart, Allison N. Rozanski, Katherine M. Munson, Kendra Hoekzema, Jane E. Ranchalis, Shane J. Neph, Adriana E. Sedeño-Cortés, Benedict Paten, Sofie R. Salama, Andrew B. Stergachis, and Evan E. Eichler

SUPPLEMENTAL MATERIAL

ADDITIONAL TABLES & FIGURES

Table S1. Structural variants of *NOTCH2NL* paralogs in T2T-CHM13, related to Figure 1C.

query paralog	ref paralog	query start	query end	mapq	structural variant size	in. (I) / del (D)
NOTCH2	NOTCH2NLC	119846347	119900735	60	54389	I
NOTCH2	NOTCH2NLC	119582683	119582682	60	737	D
NOTCH2	NOTCH2NLC	119541422	119547699	60	6278	I
NOTCH2	NOTCH2NLC	119577460	119579275	60	1816	I
NOTCH2	NOTCH2NLB	119542857	119555494	60	12638	I
NOTCH2	NOTCH2NLB	119814488	119818620	60	4133	I
NOTCH2	NOTCH2NLB	119461254	119461253	60	5629	D
NOTCH2	NOTCH2NLB	119461388	119461387	60	4741	D
NOTCH2	NOTCH2NLB	119462558	119462557	60	996	D
NOTCH2	NOTCH2NLB	119677199	119677198	60	748	D
NOTCH2	NOTCH2NLB	119857611	119857610	60	3875	D
NOTCH2	NOTCH2NLB	119917835	119917834	60	1358	D
NOTCH2	NOTCH2NLB	120135864	120137057	60	1194	I
NOTCH2	NOTCH2NLB	120134461	120135831	60	1371	I
NOTCH2	NOTCH2NLA	119814488	119818620	60	4133	I
NOTCH2	NOTCH2NLA	119677199	119677198	60	748	D
NOTCH2	NOTCH2NLA	119857611	119857610	60	3852	D
NOTCH2	NOTCH2NLA	119917825	119917824	60	1360	D
NOTCH2	NOTCH2NLA	119542836	119555473	60	12638	I
NOTCH2	NOTCH2NLA	119462604	119462603	60	996	D
NOTCH2	NOTCH2NLA	119461389	119461388	60	4741	D
NOTCH2	NOTCH2NLA	119461253	119461252	60	5629	D
NOTCH2	NOTCH2NLA	120134365	120134947	31	583	I
NOTCH2	NOTCH2NLA	120135063	120135832	31	770	I
NOTCH2	NOTCH2NLA	120135863	120137056	31	1194	I
NOTCH2NLA	NOTCH2NLC	145263860	145265217	60	1358	I
NOTCH2NLA	NOTCH2NLC	145426571	145428098	60	1528	I
NOTCH2NLA	NOTCH2NLC	145431274	145434427	60	3154	I
NOTCH2NLA	NOTCH2NLC	144857035	144858873	60	1839	I
NOTCH2NLA	NOTCH2NLB	145373282	145374823	60	1542	I
NOTCH2NLA	NOTCH2NLB	145426775	145428316	60	1542	I
NOTCH2NLA	NOTCH2NLB	145357941	145357940	60	6296	D
NOTCH2NLA	NOTCH2NLB	145452444	145455266	60	2823	I
NOTCH2NLB	NOTCH2NLC	146024204	146083108	60	58905	I
NOTCH2NLB	NOTCH2NLC	146100183	146101538	60	1356	I
NOTCH2NLB	NOTCH2NLC	146196656	146202946	60	6291	I
NOTCH2NLB	NOTCH2NLC	146270637	146273780	60	3144	I
NOTCH2NLB	NOTCH2NLC	146282918	146295060	60	12143	I
NOTCH2NLB	NOTCH2NLC	146295069	146303567	60	8499	I
NOTCH2NLB	NOTCH2NLC	146215843	146215842	60	1549	D
NOTCH2NLB	NOTCH2NLC	146461653	146461652	60	2066	D
NOTCH2NLB	NOTCH2NLC	145759226	145761064	60	1839	I
NOTCH2NLC	NOTCH2NLR	148580984	148584007	60	3024	I
NOTCH2NLC	NOTCH2NLR	148623116	148626328	60	3213	I
NOTCH2NLC	NOTCH2NLR	148627877	148631089	60	3213	I
NOTCH2NLC	NOTCH2NLR	148635700	148678456	60	42757	I
NOTCH2NLC	NOTCH2NLR	148608665	148608664	60	6274	D
NOTCH2NLC	NOTCH2NLR	148687503	148687502	60	11467	D
NOTCH2NLC	NOTCH2NLR	148687511	148687510	60	8199	D
NOTCH2NLC	NOTCH2NLR	148867048	148870884	60	3837	I
NOTCH2NLC	NOTCH2NLR	148443146	148443145	60	11467	D
NOTCH2NLC	NOTCH2NLR	148443154	148443153	60	8199	D
NOTCH2NLC	NOTCH2NLR	148335672	148335671	60	1816	D
NOTCH2NLR	NOTCH2NLB	120452239	120464876	60	12638	I

NOTCH2NLR	NOTCH2NLB	120546480	120546479	60	5629	D
NOTCH2NLR	NOTCH2NLB	120546346	120546345	60	4741	D
NOTCH2NLR	NOTCH2NLB	120545176	120545175	60	996	D
NOTCH2NLR	NOTCH2NLB	120330535	120330534	60	748	D
NOTCH2NLR	NOTCH2NLB	120730144	120730143	60	1356	D
NOTCH2NLR	NOTCH2NLB	120798010	120798009	60	3025	D
NOTCH2NLR	NOTCH2NLB	120841724	120841723	60	3184	D
NOTCH2NLR	NOTCH2NLB	120843404	120843403	60	3172	D
NOTCH2NLR	NOTCH2NLB	120845053	120845052	60	3202	D
NOTCH2NLR	NOTCH2NLB	120849542	120849541	60	40814	D
NOTCH2NLR	NOTCH2NLB	120861687	120861686	60	654	D
NOTCH2NLR	NOTCH2NLB	120594342	120594341	60	748	D
NOTCH2NLR	NOTCH2NLA	120452260	120464897	60	12638	I
NOTCH2NLR	NOTCH2NLA	120545130	120545129	60	996	D
NOTCH2NLR	NOTCH2NLA	120546345	120546344	60	4741	D
NOTCH2NLR	NOTCH2NLA	120546481	120546480	60	5629	D
NOTCH2NLR	NOTCH2NLA	120594342	120594341	60	748	D
NOTCH2NLR	NOTCH2NLA	120820315	120826597	60	6283	I
NOTCH2NLR	NOTCH2NLA	120730144	120730143	60	1358	D
NOTCH2NLR	NOTCH2NLA	120798010	120798009	60	3025	D
NOTCH2NLR	NOTCH2NLA	120843404	120843403	60	3180	D
NOTCH2NLR	NOTCH2NLA	120844944	120844943	60	3168	D
NOTCH2NLR	NOTCH2NLA	120330535	120330534	60	748	D
NOTCH2NLR	NOTCH2	120798010	120798009	60	3028	D
NOTCH2NLR	NOTCH2	120838292	120846100	60	7809	I

Table S2. Additional structural variation between human and NHA, related to Figure 2.

Variant information				NOTCH2 mapping (primary hap)		INV BP mapping (T2T-space; kbp)		
Species	Variants	Count	Bases (kbp)	Count	Bases (kbp)	Count	Bases (kbp)	p-value
PPY	PAV-DEL	816	752.8	3	0.7	71	99.5	1.000
PPY	PAV-INS	829	1025.1	6	9.1	82	140.0	1.000
PPY	Syri/PAV-INV	6	930.4	-	-	-	-	-
PPY	Sedef-SD (h1/h2)	98/97	2588.9/2588.9	1	10.6	-	-	-
PAB	PAV-DEL	810	751.7	3	0.7	72	106.1	1.000
PAB	PAV-INS	812	1016.1	5	9.0	74	123.7	1.000
PAB	Syri/PAV-INV	4	1338.3	-	-	-	-	-
PAB	Sedef-SD (h1/h2)	97/95	2611.4/2576.7	1	10.3	-	-	-
PTR	PAV-DEL	343	341.3	3	5.2	29	69.3	0.987
PTR	PAV-INS	312	1146.2	2	604.9	40	883.4	**<0.001***
PTR	Syri/PAV-INV	3	8017.1	-	-	-	-	-
PTR	Sedef-SD (h1/h2)	109/111	6565.9/6582.8	10	3660.8	-	-	-
GGO	PAV-DEL	475	591.1	5	11.1	36	141.7	0.832
GGO	PAV-INS	422	1010.9	0	0.0	35	223.8	**0.034***
GGO	Syri/PAV-INV	6	8885.6	-	-	-	-	-
GGO	Sedef-SD (pri/alt)	114/118	7103.1/7049.3	6	3043.9	-	-	-
PPA	PAV-DEL	353	359.9	3	5.2	19	51.0	0.999
PPA	PAV-INS	308	537.7	1	3.0	25	262.5	**<0.001***
PPA	Syri/PAV-INV	6	9691.5	-	-	-	-	-
PPA	Sedef-SD (pri/alt)	110/111	6459.5/6059.9	10	3532.2	-	-	-
HSA	Sedef-SD	91	8315.6	3	3988.8	30	1918.8	**<0.001***
			**Average**	3.9	931.0	46.6	365.4	

Table S3. NHA *NOTCH2NL* FLNC characterization, related to Figure 3.

Name	Iso-Seq transcript	Chr	Start	End	Strand	# of exons	Predicted AA Lengths	Long-read transcript support (NEC & testis)	Intergenic distance N2NL-NBPF (bp)	Species	Comments
PTR-NOTCH2	NOTCH2	chr1	117131242	117288384	-	--	2432	--	--	PTR/PPA/GGO/HSA	--
PTR-NOTCH2NL-1	NOTCH2NLR	chr1	106135723	106156373	+	4	246	26	11,622	PPA/PTR	ORF has exons 1, 3-5 of NOTCH2NLR
	Fusion NOTCH2NL-NBPF	chr1	106135792	106185074		20	851	69			ORF has exons 1, 3, 4 of NOTCH2NLR & NBPF exons
PTR-NOTCH2NL-2	Fusion MAGI3-NOTCH2NL	chr1	106365562	106516651	+	4	324	6	11,398	PPA/PTR	ORF has MAGI3 exon 1 & exons 3-5 of NOTCH2NLR
PTR-NOTCH2NL-3	NOTCH2NLR	chr1	108282469	108322382	+	4	235	16	11,838	PPA/PTR/GGO	ORF has exons 2-5 of NOTCH2NLR
	Fusion NOTCH2NL-NBPF	chr1	108282319	108349249		13	672	11			ORF has exons 2, 3, 4 of NOTCH2NLR & NBPF exons
	Fusion PDE4DIP-NOTCH2NL-NBPF	chr1	108214852	108349197		--	--	53			No ORF goes through the whole transcript
PTR-NOTCH2NL-4	Fusion MAGI3-NOTCH2NL	chr1	109104609	109227579	-	13	845	12	11,410	PPA/PTR	ORF has exon 1 of NOTCH2NLR, 9 MAGI3, and 3-5 exons of NOTCH2NLR
	Fusion MAGI3-NOTCH2NL-NBPF	chr1	109078294	109227828		17	1,411	53			ORF has exon 1 of NOTCH2NLR, 9 MAGI3, NOTCH2NL exons 3, 4, and NBPF exons
PTR-NOTCH2NL-5	NOTCH2NLR	chr1	109873983	109910441	-	3	146	9	11,295	PPA/PTR	ORF has exons 2, 3 of NOTCH2NLR & partial exon 4 (TRUNCATION) - transcript goes through until NOTCH2NLR-like exon 5
	Fusion NOTCH2NL-NBPF	chr1	109846910	109910766		3	146	12			ORF has exons 2, 3 of NOTCH2NLR & partial exon 4 (TRUNCATION) - transcript goes through to NBPF exons
	Fusion TXNIP-NOTCH2NL-NBPF	chr1	109846504	109953395		4	230	62			ORF has exon 1 of TXNIP, exons 2,3 of NOTCH2NLR, and partial exon 4 (TRUNCATION) - transcript goes through until NOTCH2NLR-like exon 5
PTR-NOTCH2NL-6	NOTCH2NLR	chr1	110315936	110336372	+	4	243	11	11,504	PPA/PTR/GGO	ORF has exon 1, 3-5 of NOTCH2NLR
	Fusion NOTCH2NL-NBPF	chr1	110313408	110368080		20	856	22			ORF that has exons 1, 3, 4 of NOTCH2NLR & NBPF exons
	Fusion SORT1-LRIG2-NOTCH2NL-NBPF	chr1	110225344	110368328		--	--	14			No ORF goes through the whole transcript
PTR-NOTCH2NL-7	Fusion with MAGI3	chr1	117544406	117696496	-	4	324	7	11,693	PPA/PTR	ORF has exon 1 of MAGI3 & exons 3-5 of NOTCH2NLR
PTR-NOTCH2NL-8	NOTCH2NLR	chr1	118044677	118065257	-	4	246	9	11,295	PTR	ORF has exons 1, 3-5 of NOTCH2NLR
	Fusion NOTCH2NL-NBPF	chr1	117960416	118065110		58	3033	16			ORF has exons 1, 3, 4 of NOTCH2NLR & NBPF exons
	Fusion LRIG2-NOTCH2NL	chr1	118044029	118124464		--	--	5			No ORF goes through the whole transcript
	Fusion LRIG2-NOTCH2NL-NBPF	chr1	117960416	118125899		--	--	4			No ORF goes through the whole transcript
PTR-NOTCH2NL-9	NOTCH2NLR	chr1	121665898	121686392	+	4	245	11	11,486	PPA/PTR	ORF has exons 1, 3-5 of NOTCH2NLR
	Fusion NOTCH2NL-NBPF	chr1	121665622	121801595		88	4694	48			ORF has exons 1, 3, 4 of NOTCH2NLR & NBPF exons
PPA-NOTCH2	NOTCH2	chr1	112555608	112716322	-	--	2610	--	--	PTR/PPA/GGO/HSA	--
PPA-NOTCH2NL-1	NOTCH2NLR	chr1	101669793	101703165	-	4	235	46	11,607	PPA/PTR/GGO	ORF has exons 2-5 of NOTCH2NLR human
	Fusion NOTCH2NL-NBPF	chr1	101642931	101703233		17	656	132			ORF has exons 2, 3, 4 of NOTCH2NLR & NBPF exons
	Fusion PDE4DIP-NOTCH2NL-NBPF	chr1	101641810	101777882		--	--	155			No ORF goes through the whole transcript.
PPA-NOTCH2NL-2	Fusion MAGI3-NOTCH2NL	chr1	103485748	103636324	-	4	237	6	11,837	PPA/PTR	ORF has exon 1 of MAGI3 & exons 3-5 of NOTCH2NLR
PPA-NOTCH2NL-3	NOTCH2NLR	chr1	103841187	103861631	+	4	246	11	11,672	PPA/PTR	ORF has exons 1, 3-5 of NOTCH2NLR
	Fusion NOTCH2NL-NBPF	chr1	103815726	103861620		15	757	98			ORF has exons 1, 3, 4 of NOTCH2NLR & NBPF exons
PPA-NOTCH2NL-4	Fusion MAGI3-NOTCH2NL	chr1	104697226	104820526		13	845	201	--	PPA/PTR	ORF has exon 1 of NOTCH2NLR, 9 MAGI3, & 3-5 exons of NOTCH2NLR
PPA-NOTCH2NL-5	NOTCH2NLR	chr1	105315877	105349528	+	4	235	11	11,690	PPA/PTR	ORF has exons 2-5 of NOTCH2NLR
	Fusion NOTCH2NL-NBPF	chr1	105288382	105349479		15	743	69			ORF has exons 2, 3, 4 of NOTCH2NLR & NBPF exons
	Fusion TXNIP-NOTCH2NL-NBPF	chr1	105286533	105389706		5	319	21			ORF has exon 1 of TXNIP & exons 2, 3-5 of NOTCH2NLR
PPA-NOTCH2NL-6	NOTCH2NLR	chr1	105752206	105772648	-	4	246	7	11,773	PPA/PTR	ORF has exons 1, 3-5 of NOTCH2NLR
	Fusion NOTCH2NL-NBPF	chr1	105752087	105795054		14	682	81			ORF has exons 1, 3, 4 of NOTCH2NLR & NBPF exons
PPA-NOTCH2NL-7	NOTCH2NLR	chr1	112968284	112977175	-	4	246	0	11,194	PPA	ORF has exons 3-5 of NOTCH2NLR
PPA-NOTCH2NL-8	Fusion MAGI3-NOTCH2NL	chr1	113432163	113583978	-	4	324	12	11,233	PPA/PTR	ORF has exon 1 of MAGI3 & exons 3-5 of NOTCH2NLR
PPA-NOTCH2NL-9	NOTCH2NLR	chr1	113892494	113913027	-	4	246	56	11,785	PPA	ORF has exons 1, 3-5 of NOTCH2NLR
PPA-NOTCH2NL-10	NOTCH2NLR	chr1	117521012	117542196	+	4	246	12	11,731	PPA/PTR/GGO	ORF has exons 1, 3-5 of NOTCH2NLR
	Fusion NOTCH2NL-NBPF	chr1	117520465	117596479		50	2399	35			ORF has exons 1, 3, 4 of NOTCH2NLR & NBPF exons

	Fusion SORT1-LRIG2-NOTCH2NL-NBPF	chr1 117431222 117596326		--	--	4			No ORF goes through the whole transcript
GGO-NOTCH2	NOTCH2	chr1 124746752 124900741	-	--	2611	--	--	PTR/PPA/GGO/HSA	--
GGO-NOTCH2NL-1	NOTCH2NLR	chr1 116765261 116796179	+	--	--	2	11,618	PPA/PTR/GGO	Has exon 3-5 of NOTCH2NLR but no exon 1/2 and no M start
	Fusion NOTCH2NL-NBPF	chr1 116765192 116847395		--	--	13			Has exon 3-4 of NOTCH2NLR & NBPF exons but no exon 1/2 & no M start
	Fusion SORT1-LRIG2-NOTCH2NL-NBPF	chr1 116693440 116846869		--	--	6			No ORF goes through the whole transcript.
GGO-NOTCH2NL-2	NOTCH2NLR	chr1 117331132 117364496	-	4	235	5	11,825	PPA/PTR/GGO	ORF has exons 2-5 of NOTCH2NLR
	Fusion NOTCH2NL-NBPF	chr1 117294017 117373312		24	1114	11			ORF has exons 2-4 of NOTCH2NLR & NBPF exons
	Fusion PDE4DIP-NOTCH2NL-NBPF	chr1 117294017 117447550		--	--	16			No ORF goes through the whole transcript.
GGO-NOTCH2NL-3	Fusion LRIG2-NOTCH2NL-NBPF	chr1 125591450 125661487	-	--	1662	5	5385	GGO	Has exons of LRIG2, exon 3 of NOTCH2NLR, & NBPF exons but no M start
	Fusion NOTCH2NL-NBPF	chr1 125591876 125635112		--	--	8			Has exon 3 of NOTCH2NLR and NBPF exons but no exon 1/2 and no M start
GGO-NOTCH2NL-4	Fusion LRIG2-NOTCH2NL-NBPF	chr1 129364798 129435830	+	--	--	19	5,608	GGO	Has 2 LRIG2 exons, exon 3 of NOTCH2NLR, & NBPF exons but early stop in exon 2
	Fusion NOTCH2NL-NBPF	chr1 129389184 129435600		--	--	2			Has 3-4 of NOTCH2NLR & NBPF exons but no exon 1/2 and no M start
GGO-NOTCH2NL-5	Fusion MAGI3-NOTCH2NL-NBPF	chr1 129726316 129995372	+	--	1487	2	5,588	GGO	Has exons of MAGI3, exon 3 of NOTCH2NLR, & then NBPF exons but no M start
	Fusion NOTCH2NL-NBPF	chr1 129964606 129995476		--	--	6			Has 3-4 of NOTCH2NLR & NBPF exons but no exon 1/2 and no M start
GGO-NOTCH2NL-6	Fusion NOTCH2NL-NBPF	chr1 130013152 130080198	-	13	783	72	39,879	GGO	ORF has exon 1 of NOTCH2NLR & NBPF exons
GGO-NOTCH2NL-7	NOTCH2NLR	chr1 130265287 130274296	-	--	--	14	11,779	GGO	Has 3-5 of NOTCH2NLR but no exon 1/2 and no M start
	Fusion NOTCH2NL-NBPF	chr1 130222211 130280603		--	--	15			Has 3-4 of NOTCH2NLR & NBPF exons but no exon 1/2 and no M start
	Fusion BRD9-NOTCH2NL	chr1 130264240 130305169		--	--	8			No ORF that goes through the whole transcript.
	Fusion BRD9-NOTCH2NL-NBPF	chr1 130221951 130304952		--	--	4			No ORF that goes through the whole transcript.
HSA-NOTCH2	NOTCH2	chr1 119924936 120082923	-	--	2471	--	--	PTR/PPA/GGO/HSA	--
HSA-NOTCH2NLA	NOTCH2NLA	chr1 145272197 145345902	-	5	236	--	11,336	HSA	
HSA-NOTCH2NLB	NOTCH2NLB	chr1 146108509 146181500	+	5	249	--	11,375	HSA	
HSA-NOTCH2NLC	NOTCH2NLC	chr1 148535272 148596912	+	5	236	--	11,726	HSA	
HSA-NOTCH2NLR	NOTCH2NLR	chr1 120737165 120807117	+	5	274	--	11,635	HSA	
	[a]Fusion NOTCH2NL-NBPF	chr1 120737168 120851627		36	1673	--			

[a]Fusion exists in all human *NOTCH2NL* but only *NOTCH2NLR* fusion is used for comparison.

Table S4. Population origin of HPRC[a] haplotypes resolved[b] across the *NOTCH2NL* locus, related to Figures 4 and 5.

superpopulation	superpopulation total	population	population total
		African Caribbean in Barbados	11
		Gambian in Western Divisions - Mandinka	15
AFR	34	Mende in Sierra Leone	6
		Yoruba in Ibadan, Nigeria	2
		African Ancestry in Southwest USA	2
		Maasai in Kinyawa, Kenya	1
		Puerto Rican in Puerto Rico	14
AMR	23	Colombian in Medellin, Colombia	2
		Peruvian in Lima, Peru	7
		Kinh in Ho Chi Minh City, Vietnam	1
EAS	7	Southern Han Chinese in Hu Nan Province, China	5
		Chinese Ancestry in USA	1
EUR	1	Ashkenazim Jewish	1
SAS	1	Punjabi in Lahore, Pakistan	1

[a]HPRC: Human Pangenome Reference Consortium

[b]This includes haplotypes that are not completely assembled across the centromere but have no gaps or collapses between *NOTCH2* and *NOTCH2NLR*, and *NOTCH2NLA/B/C*, respectively.

Table S5. *NOTCH2NL/GFP* gBlocks for measuring protein expression, related to STAR Methods.

Sequence name	5' – sequence – 3'
HA-NOTCH2tv-IRES-NheI-FseI gBlock	GGTCTAGAGCTAGCGAATTCGCCGGTGCCACCATGTACCCATACGATGTTCCAGA<br>TTACGCTCCCGCCCTGCGCCCCGCTCTGCTGTGGGCGCTGCTGGCGCTCTGGCT<br>GTGCTGCGCGGCCCCCGCGCATGCATTGCAGTGTCGAGATGGCTATGAACCCTG<br>TGTAAATGAAGGAATGTGTGTTACCTACCACAATGGCACAGGATACTGCAAATGTC<br>CAGAAGGCTTCTTGGGGGAATATTGTCAACATCGAGACCCCTGTGAGAAGAACCG<br>CTGCCAGAATGGTGGGACTTGTGTGGCCCAGGCCATGCTGGGGAAAGCCACGTG<br>CCGATGTGCCTCAGGGTTTACAGGAGAGGACTGCCAGTACTCAACATCTCATCCA<br>TGCTTTGTGTCTCGACCCTGCCTGAATGGCGGCACATGCCATATGCTCAGCCGGG<br>ATACCTATGAGTGCACCTGTCAAGTCGGGTTTACAGGTAAGGAGTGCCAATGGAC<br>GGATGCCTGCCTGTCTCATCCCTGTGCAAATGGAAGTACCTGTACCACTGTGGCC<br>AACCAGTTCTCCTGCAAATGCCTCACAGGCTTCACAGGGCAGAAATGTGAGACTG<br>ATGTCAATGAGTGTGACATTCCAGGACACTGCCAGCATGGTGGAACCTGCCTCAA<br>CCTGCCTGGTTCCTACCAGTGCCAGTGCCCTCAGGGCTTCACAGGCCAGTACTGT<br>GACAGCCTGTATGTGCCCTGTGCACCCTCACCTTGTGTCAATGGAGGTACCTGTC<br>GGCAGACTGGTGACTTCACTTTTGAGTGCAACTGCCTTCCAGAAACAGTGAGAAAT<br>AAGAGGAACAGAGCTCTGGGAAAGGGACAGGCAAGTCTGGAATGGAAAAGAACA<br>TGAGTCGACCGCTTGGAATAAGGCCGGTGTGCGTTTGTCTATATGTTATTTTCCAC<br>CATATTGCCGTCTTTTGGCAATGTGAGGGCCCGGAAACCTGGCCCTGTCTTCTTG<br>ACGAGCATTCCTAGGGGTCTTTCCCCTCTCGCCAAAGGAATGCAAGGTCTGTTGA<br>ATGTCGTGAAGGAAGCAGTTCCTCTGGAAGCTTCTTGAAGACAAACAACGTCTGTA<br>GCGACCCTTTGCAGGCAGCGGAACCCCCCACCTGGCGACAGGTGCCTCTGCGGC<br>CAAAAGCCACGTGTATAAGATACACCTGCAAAGGCGGCACAACCCCAGTGCCACG<br>TTGTGAGTTGGATAGTTGTGGAAAGAGTCAAATGGCTCTCCTCAAGCGTATTCAAC<br>AAGGGGCTGAAGGATGCCCAGAAGGTACCCCATTGTATGGGATCTGATCTGGGG<br>CCTCGGTGCACATGCTTTACATGTGTTTAGTCGAGGTTAAAAAAACGTCTAGGCCC<br>CCCGAACCACGGGGACGTGGTTTTCCTTTGAAAAACACGATGATAAGATCTGCGA<br>TCTAAGTAAGCTTGGCATTCCGGTACTGTTGGTAAAGCCACCATGGAATCCGGCC<br>GGCCCGAATTCGGC
HA-NOTCH2NLB pEF1A Gibson gBlock	GATGTTCCAGATTACGCTTGTCGAGATGGCTATGAACCCTGTGTAAATGAAGGAAT<br>GTGTGTTACCTACCACAATGGCACAGGATACTGCAAATGTCCAGAAGGCTTCTTG<br>GGGGAATATTGTCAACATCGAGACCCCTGTGAGAAGAACCGCTGCCAGAATGGTG<br>GGACTTGTGTGGCCCAGGCCATGCTGGGGAAAGCCACGTGCCGATGTGCCTCAG<br>GGTTTACAGGAGAGGACTGCCAGTACTCGACATCTCATCCATGCTTTGTGTCTCGA<br>CCTTGCCTGAATGGCGGCACATGCCATATGCTCAGCCGGGATACCTATGAGTGCA<br>CCTGTCAGGTCGGGTTTACAGGTAAGGAGTGCCAATGGACCGATGCCTGCCTGTC<br>TCATCCCTGTGCAAATGGAAGTACCTGTACCACTGTGGCCAACCAGTTCTCCTGCA<br>AATGCCTCACAGGCTTCACAGGGCAGAAGTGTGAGACTGATGTCAATGAGTGTGA<br>CATTCCAGGACACTGCCAGCATGGTGGCATCTGCCTCAACCTGCCTGGTTCCTAC<br>CAGTGCCAGTGCCTTCAGGGCTTCACAGGCCAGTACTGTGACAGCCTGTATGTGC<br>CCTGTGCACCCTCGCCTTGTGTCAATGGAGGCACCTGTCGGCAGACTGGTGACTT<br>CACTTTTGAGTGCAACTGCCTTCCAGAAACAGTGAGAAGAGGAACAGAGCTCTGG<br>GAAAGAGACAGGGAAGTCTGGAATGGAAAAGAACACGATGAGAATTAGGTCGACC<br>GCTTGGAATAA
HA-NOTCH2NLR pEF1A Gibson gBlock	GATGTTCCAGATTACGCTCCCGCCCTGCGTCCCGCTCTGCTGTGGGCGCTGCTG<br>GCGCTCTGGCTGTGCTGGGCGGCCCCCGCGCATGCATTGCAGTGTCGAGATGGC<br>TATGAACCCTGTGTAAATAAAGGAATGTGTGTTACCTACCACAGTGGCACAGGATA<br>CTGCAAATGTCCAGAAGGCTTCTTGGGGGAATATTGTCAACATCGAGACCCCTGT<br>GAGAAGAACCGCTGCCAGAATGGTGGGACTTGTGTGGCCCAGGCCATGCTGGGG<br>AAAGCCACGTGCCGGTGTGCCTCAGGGTTTACAGGAGAGGACTGCCAGTACTCG<br>ACACCTCATCCATGCTTTGTGTCTCGACCTTGCCTGAATGGCGGCACATGCCATAT<br>GCTCAGCCGGGATACCTATGAGTGCACCTGTCAAGTCGGGTTTACAGGTAAGGAG<br>TGCCAATGGACCGATGCCTGCCTGTCTCATCTCTGTGCAAATGGAAGTACCTGTA<br>CCACTGTGGCCAACCAGTTCTCCTGCAAATGCCTCACAGGCTTCACAGGGCAGAA<br>GTGTGAGACTGATGTCAATGAGTGTGACATTCCAGGACACTGCCAGCATGGTGGC<br>ACCTGCCTCAACCTGCCTGGTTCCTACCAGTGCCAGTGCCTTCAGGGCTTCACAG<br>GCCAGTACTGTGACAGACTGTATGTGCCCTGTGCACACTCGCCTTGTGTCAATGG<br>AGGCACCTGTCGGCAGACTGGTGACTTCACTTTTGAGTGCAACTGCCTTCCAGAA<br>ACAGTGAGAAATAAGAGGAACAGAGCTCTGGGAAAGAGACAGGCAAGTCTGGAAT<br>GGAAAAGAACACGATGAGTCGACCGCTTGGAATAA

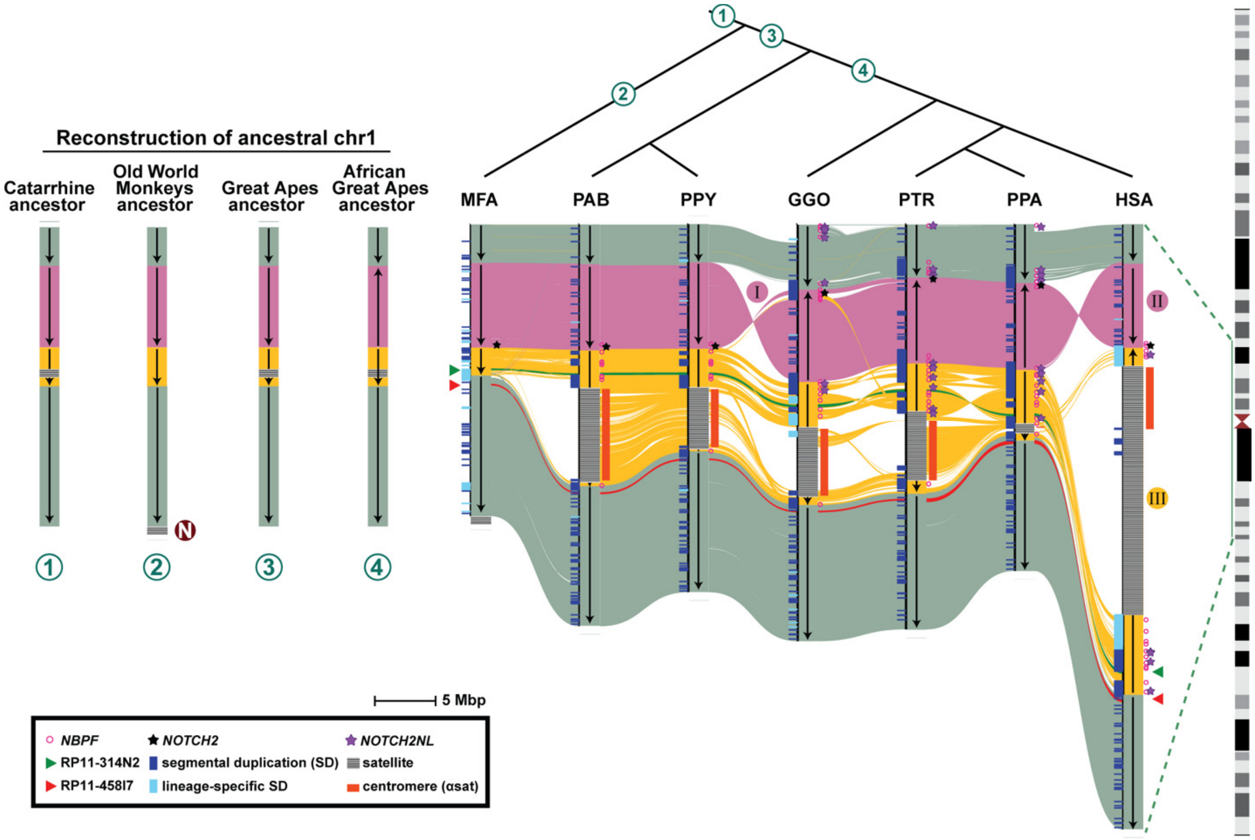

Figure S1. Ape evolutionary rearrangement and expansion of human chromosome 1p21.2-q23.2, related to Figure 2. The genomic structure of chromosome 1p21.2-q23.2 region is compared among macaque (MFA), Sumatran orangutan (PAB), Bornean orangutan (PPY), gorilla (GGO), chimpanzee (PTR), bonobo (PPA), and human (HSA) with annotations that include ancestral *NOTCH2* (black stars), *NOTCH2NL* duplications (purple stars), *NBPF* duplications (pink circles), and the centromere (orange bars). The circled numbers represent previous ancestral states of chromosome 1. The circled N represents a centromere repositioning event (N, neocentromere). Three distinct evolutionary inversions are predicted (I, II, III). Two probes (RP11-314N2, green, and RP11-458I7, red) used in FISH analyses from Szamalek et al. (2006)[S1] are shown (green and red triangles). Both probes map to the q-arm in humans, with the green probe located inside the inverted region and the red probe outside. FISH data from Szamalek et al. (2006)[S1] revealed that in chimpanzee the green probe maps to the region homologous to the human p-arm, while the red probe maps to the q-arm. Sequence analysis supports the FISH mapping and shows that in great apes the sequence of the two probes (represented as red and green lines in the SVbyEye) map on opposite sides of the centromere.

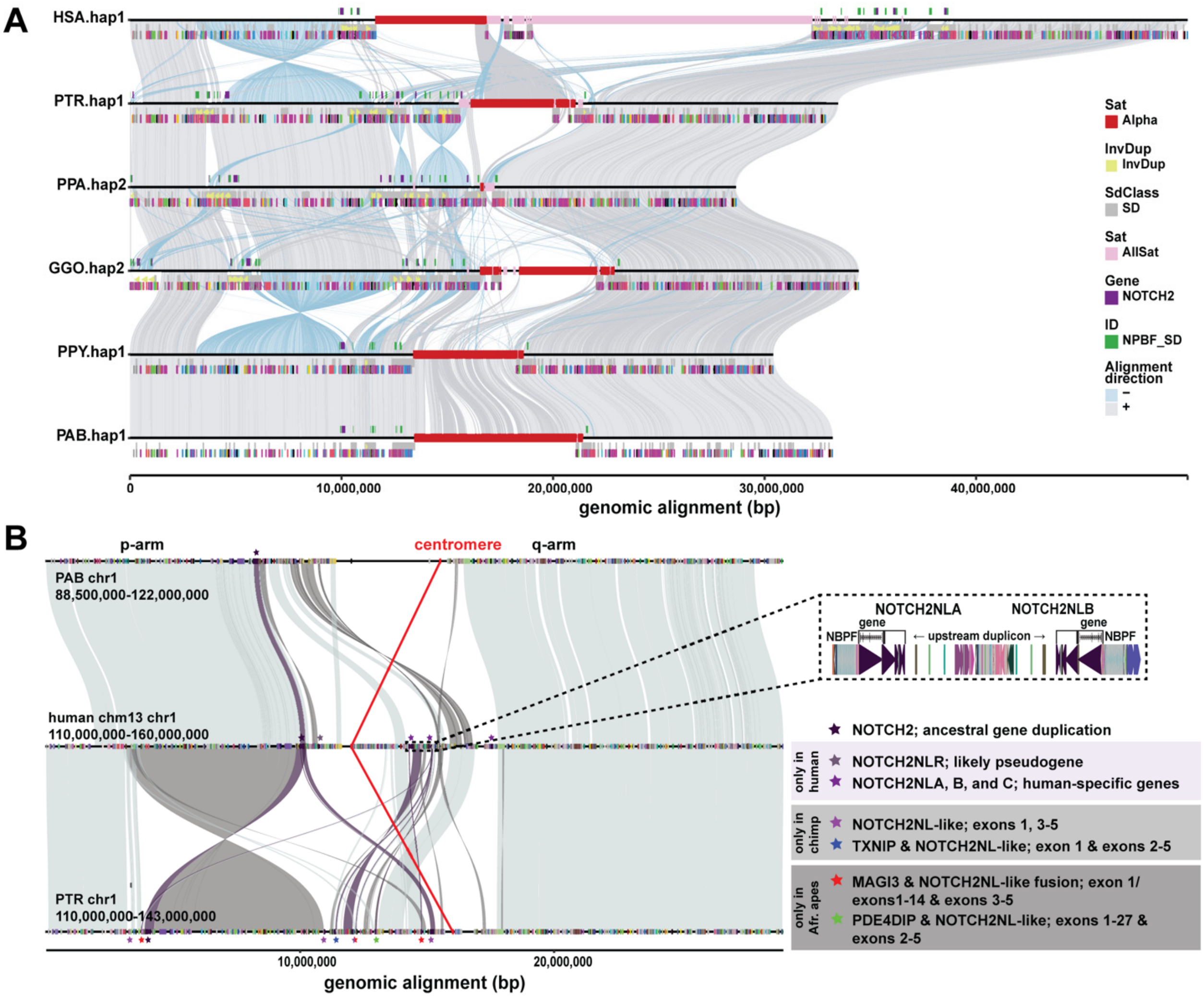

Figure S2. NHP and human chromosome 1 evolutionary rearrangements, related to Figure 2.
A) Stacked SVbyEye plot of human chromosome 1 compared to syntenic chromosomes in other apes over 30-50 Mbp of the pericentromeric region. Directly aligned sequence (gray) is compared to inversion rearrangements (light blue) with various annotations including *NOTCH2NL* and *NBPF* genes, satellites, and others. **B)** A zoomed-in comparison including only chimpanzee, orangutan, and human where centromeric sequence has been masked to highlight the non-orthologous location of NHA *NOTCH2NL* transcripts (colored stars) as described by Fiddes et al. (2018)[S2].

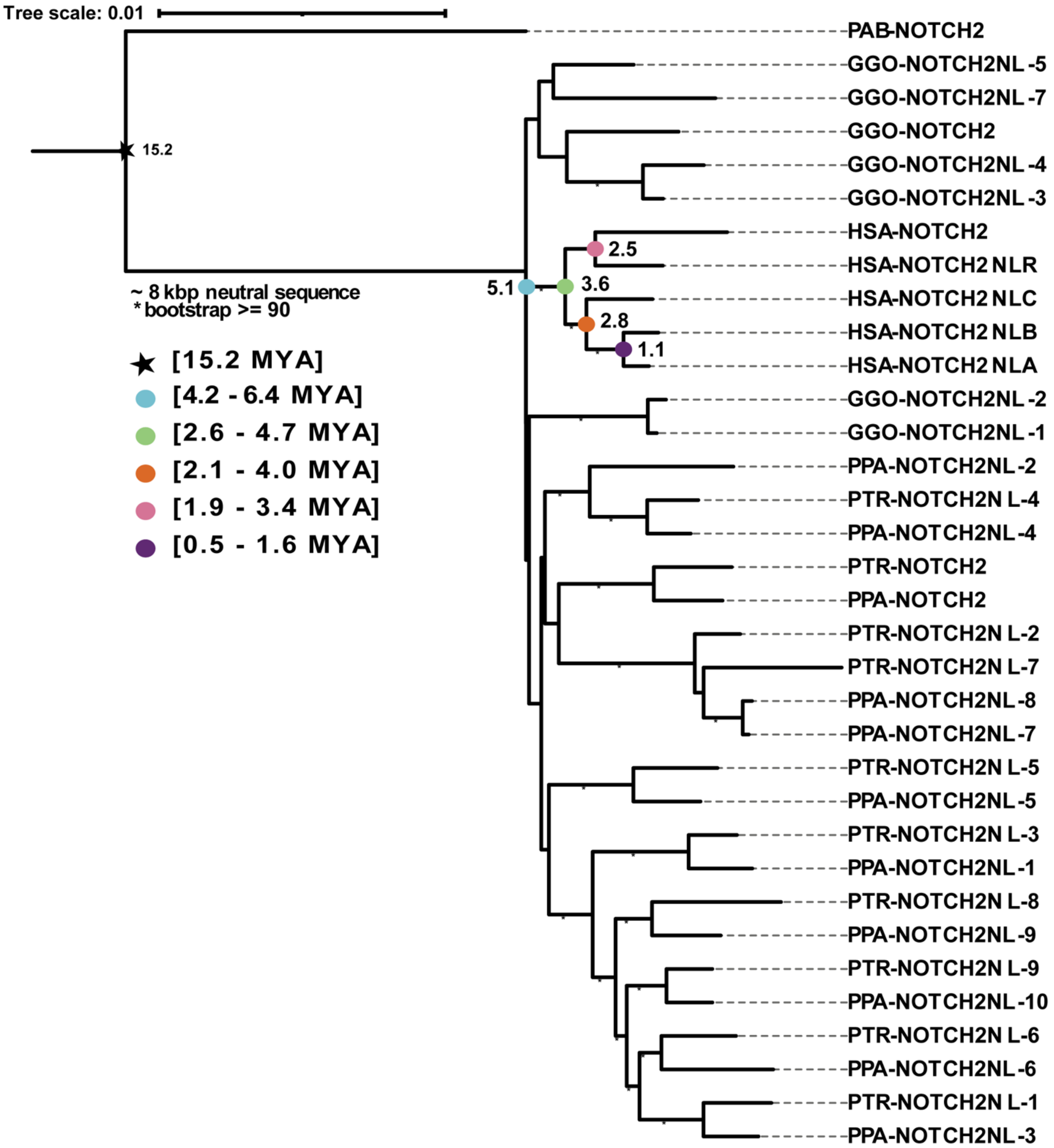

Figure S3. Extended NHA phylogeny, related to Figure 3. An alternate maximum likelihood phylogeny based on an MSA of 8 kbp from intron 3 of *NOTCH2/NL* sequence from paralogs of five ape species, using Sumatran orangutan as an outgroup. Boots representing 25/26 NHA homologs. Bootstrap support (>90%) is indicated (asterisk) and is less robust than the phylogeny in Figure 3A but contains more taxa. Estimated divergence times of human paralogs and their confidence intervals are indicated (multicolored dots). Timings were based on human–orangutan divergence time of 15.2 MYA (Methods).

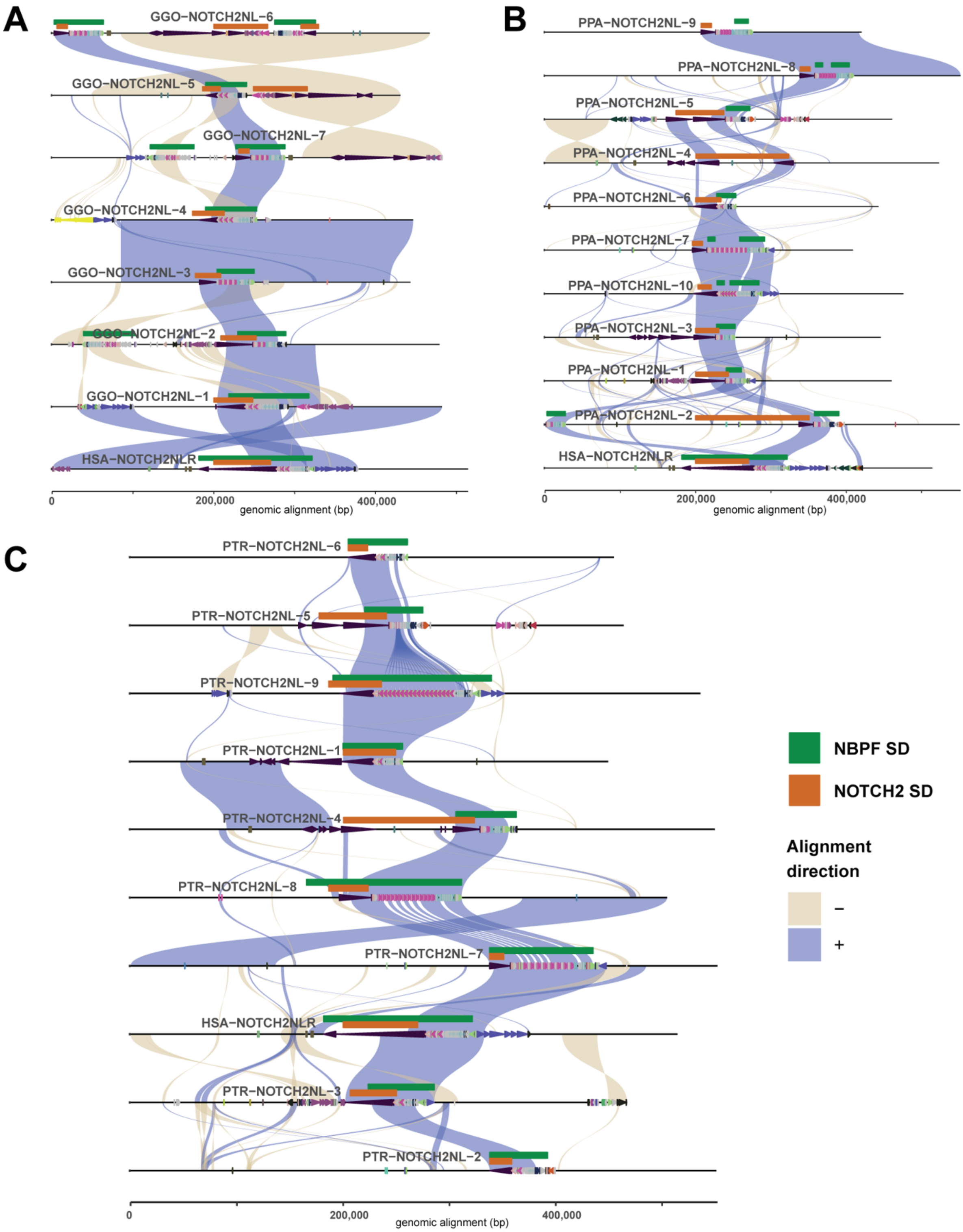

Figure S4. Ladder alignment of NHA *NOTCH2NL* homologs, related to Figure 3. Gorilla **(A)**, bonobo **(B)**, and chimpanzee **(C)** self-alignments (with human *NOTCH2NLR*) show a consistent association between the *NOTCH2/NL* duplication (purple) and the core duplicon *NBPF* (green). However, the breakpoints of these alignments are largely different not just from humans but also each other.

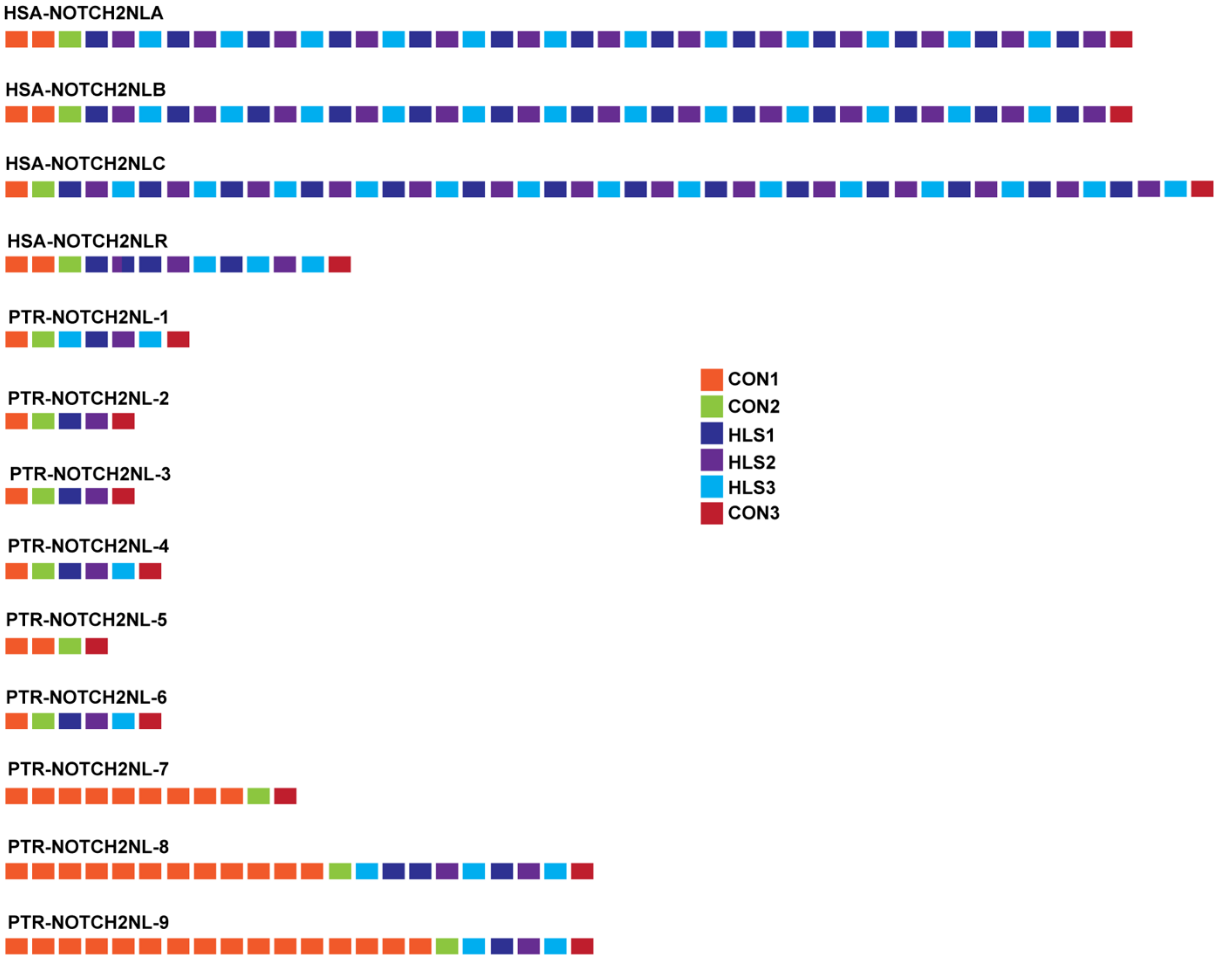

Figure S5. NBPF duplicon analysis, related to Figure 3. For each of the HSA and PTR *NOTCH2NL* copies, the *NBPF* directly downstream is represented in terms of the DUF1220 domains present—CON1, CON2, CON3, HLS1, HLS2, HLS3, based on Fiddes et al. (2019)[S3]. The *NBPF* copies downstream of *NOTCH2NL* copies in PPA and GGO show similar patterns of DUF1220 repeats as well.

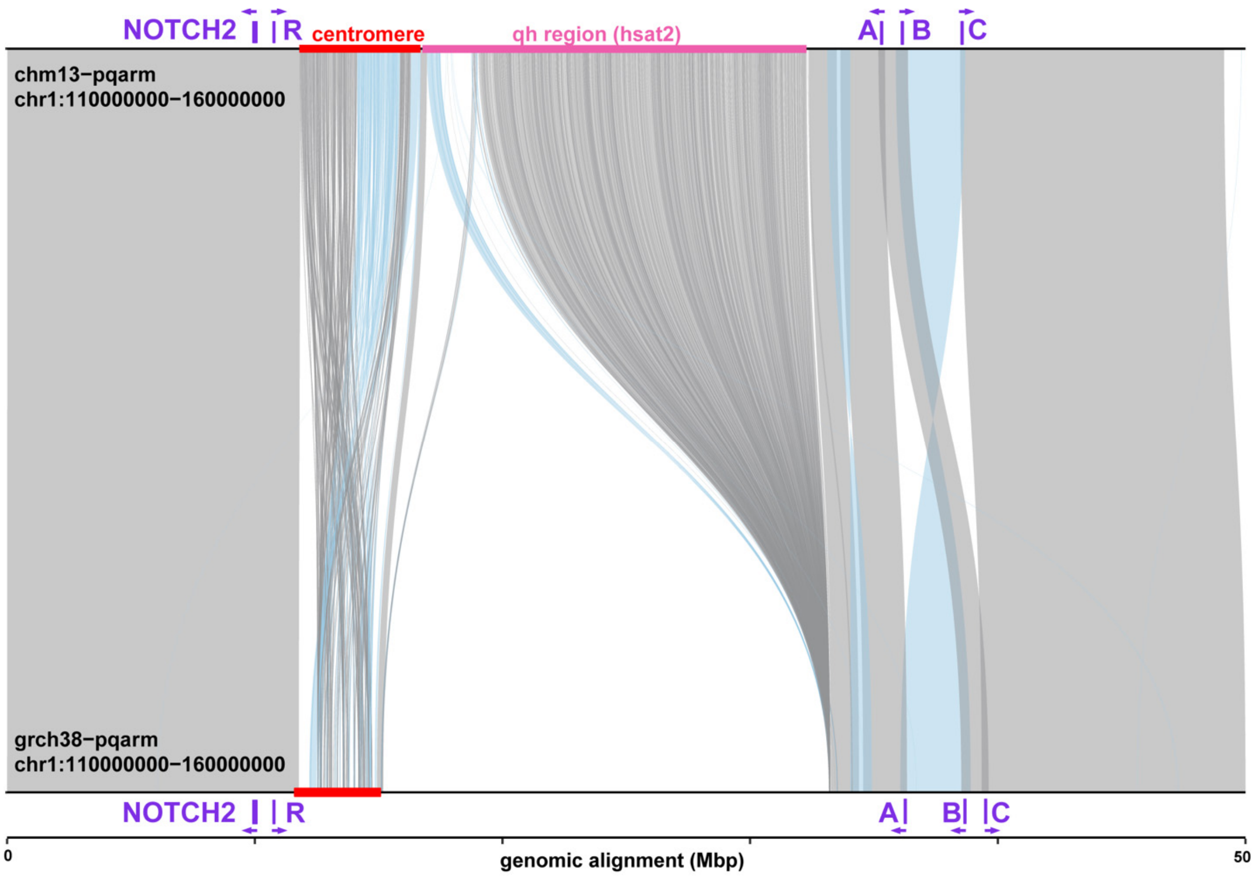

Figure S6. T2T-CHM13 comparison to GRCh38, related to STAR Methods. Organization of the *NOTCH2/NL* region in the completed T2T haploid assembly CHM13 against the previous reference, GRCh38. The T2T-CHM13 assembly has expanded the sequence and annotations that cover the centromere (red) and qh region (pink), which are two large satellite sequences that separate the two *NOTCH2/NL* (purple) loci. A long inversion around *NOTCH2NLB* that may include both *NOTCH2NLA/C* on either side changes the gene's orientation between the two assemblies (relevant alignments have increased color opacity).

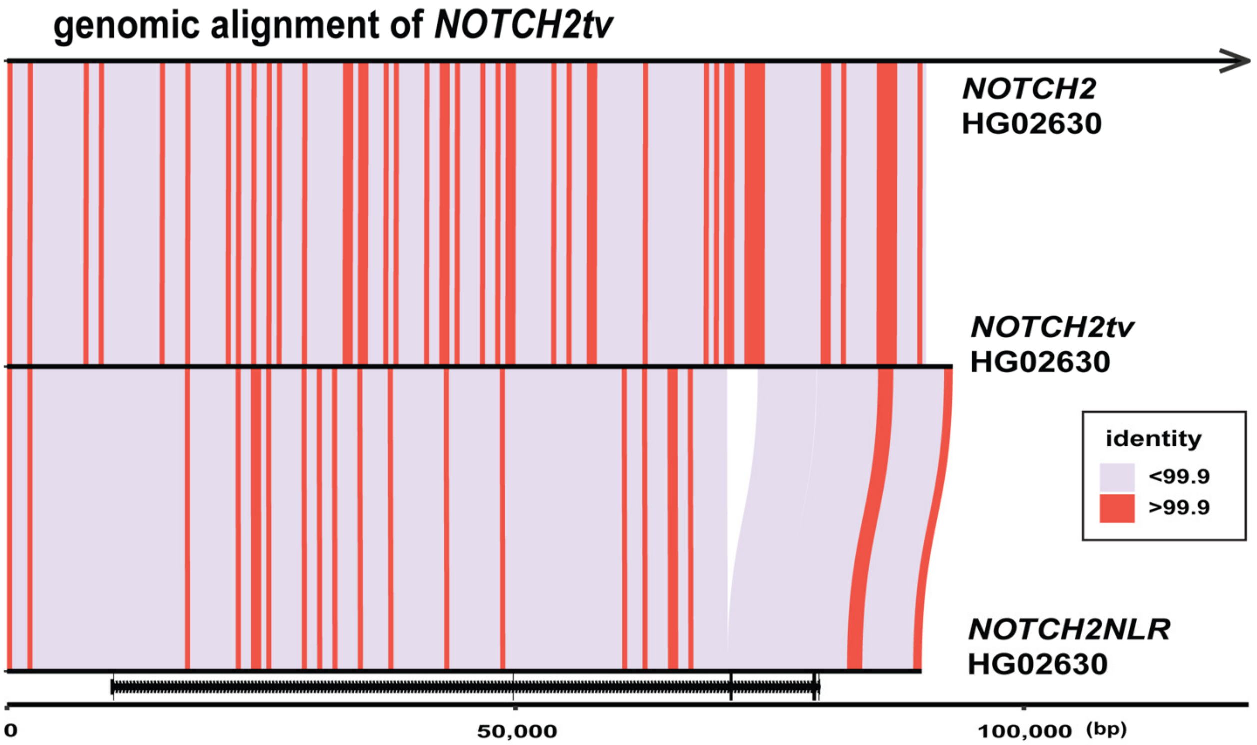

Figure S7. NOTCH2NLR to NOTCH2tv gene conversion, related to Figure 5. Nucleotide alignment of *NOTCH2tv* (middle) to ancestral *NOTCH2* (top) and *NOTCH2NLR* (bottom) confirms larger stretches of near perfect sequence identity (red≥99.9%) between *NOTCH2tv and NOTCH2*, consistent with IGC.

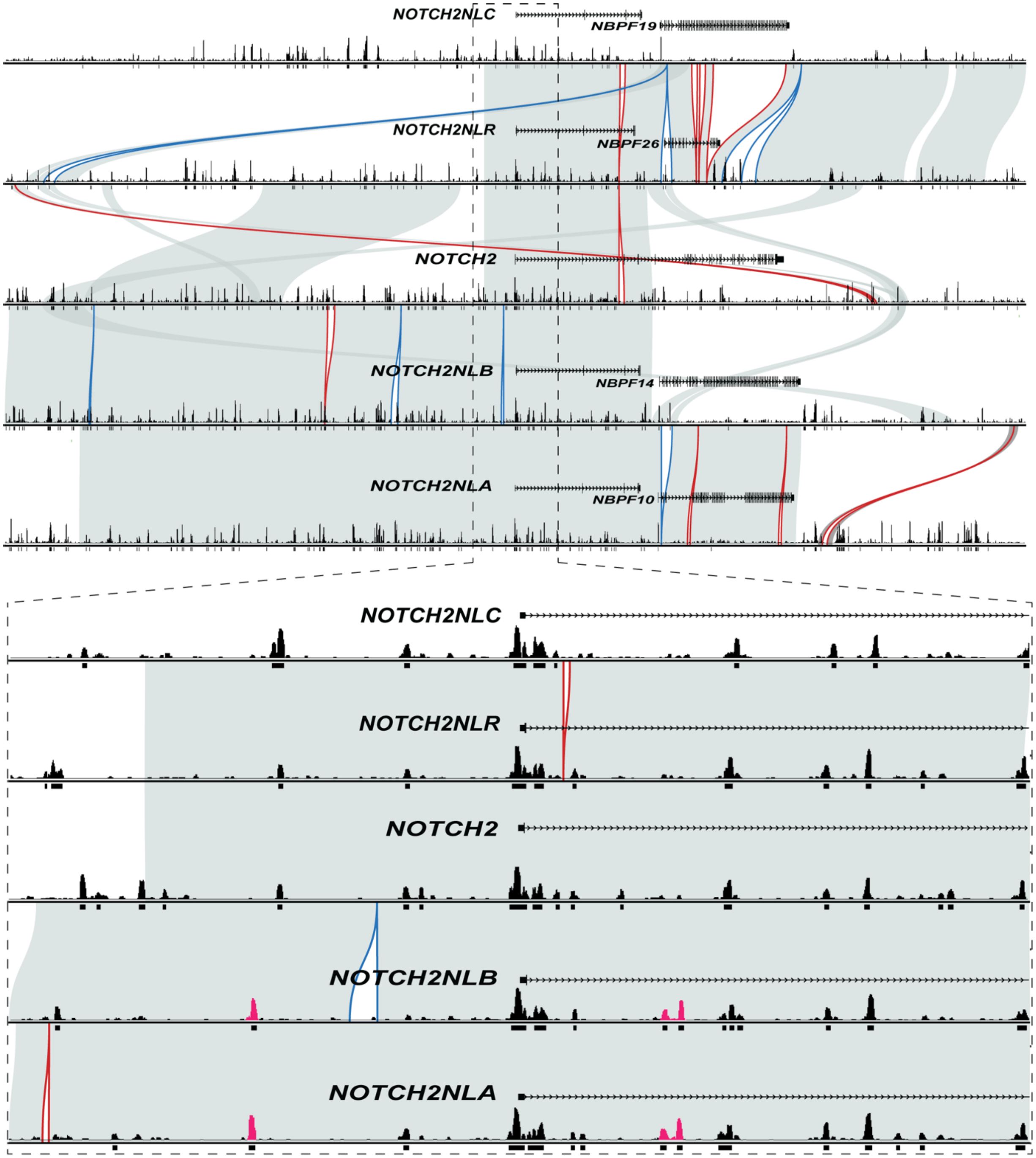

Figure S8. Regulatory architecture of T2T-CHM13, related to STAR Methods. Fiber-seq chromatin actuation peaks for each T2T-CHM13 *NOTCH2/NL* paralog in the context of homology (gray) and gene model 300 kbp on either side of the TSS. Examples of signals of paralog-specific actuation (pink) are shown in pop-out panel (25 kbp on either side of TSS) in *NOTCH2NLA* and *NOTCH2NLB*. Structural variants like insertions (blue) and deletions (red) are outlined.

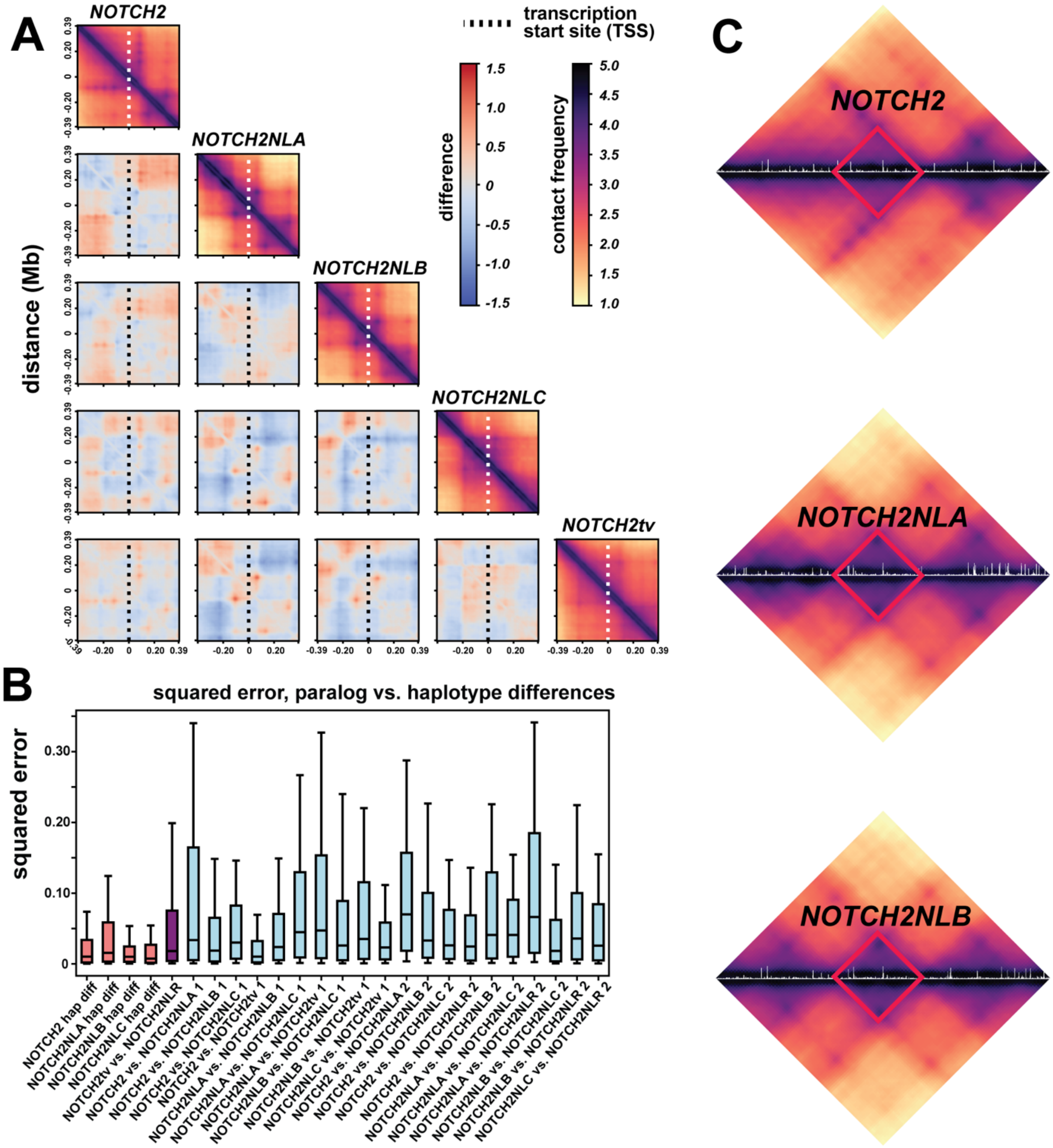

Figure S9. Predicted TADs and associated regulatory architectures in *NOTCH2NL* paralog regions, related to Figure 6. A) FiberFold TAD *NOTCH2NL* paralog predictions and pairwise heatmap of contact similarities and differences. Dotted lines represent gene TSS. **B)** Squared error chromatin accessibility differences between haplotypes and pairwise paralog comparisons. **C)** Fiber-seq chromatin actuation overlaid on TAD predictions for *NOTCH2*, *NOTCH2NLA*, and *NOTCH2NLB* to show which regulatory peaks are included in contact regions.

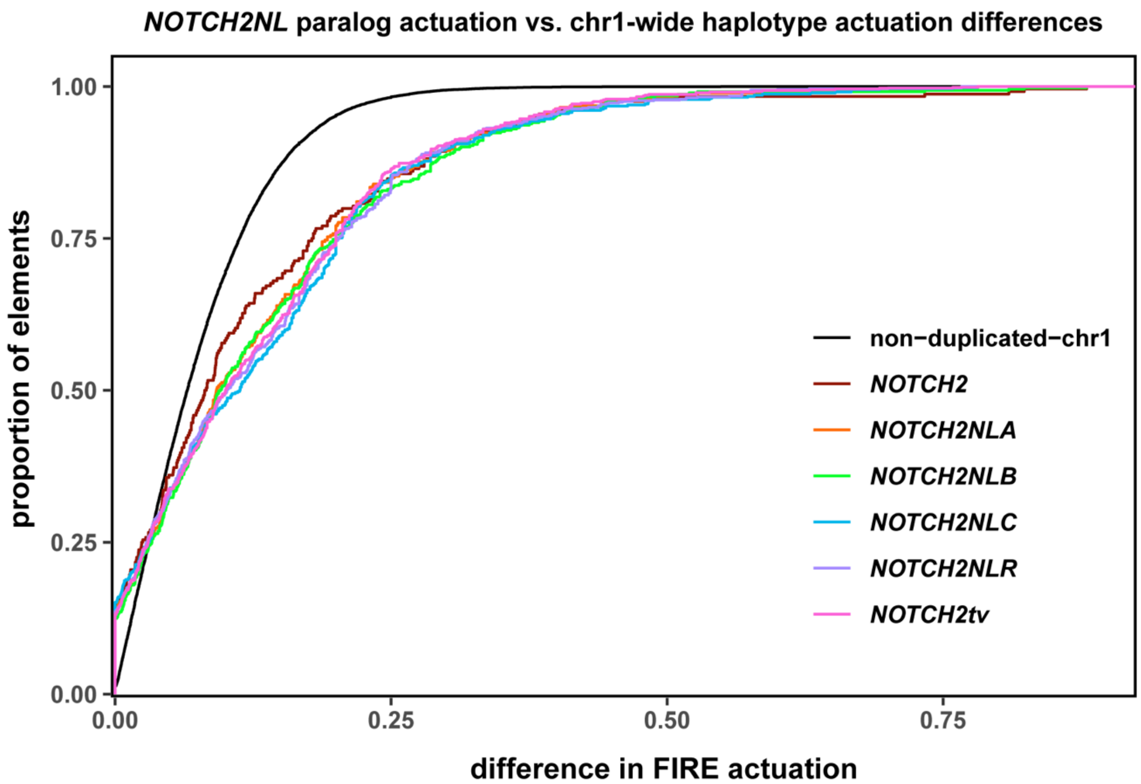

Figure S10. Comparison of *NOTCH2NL* paralog and haplotype actuation, related to Figure 6. The model of expected haplotype differences on chromosome 1 using brain organoid data mapped to T2T-CHM13 (black) shows a higher proportion of regulatory elements with less actuation difference than what is seen for the different *NOTCH2NL* paralogs.

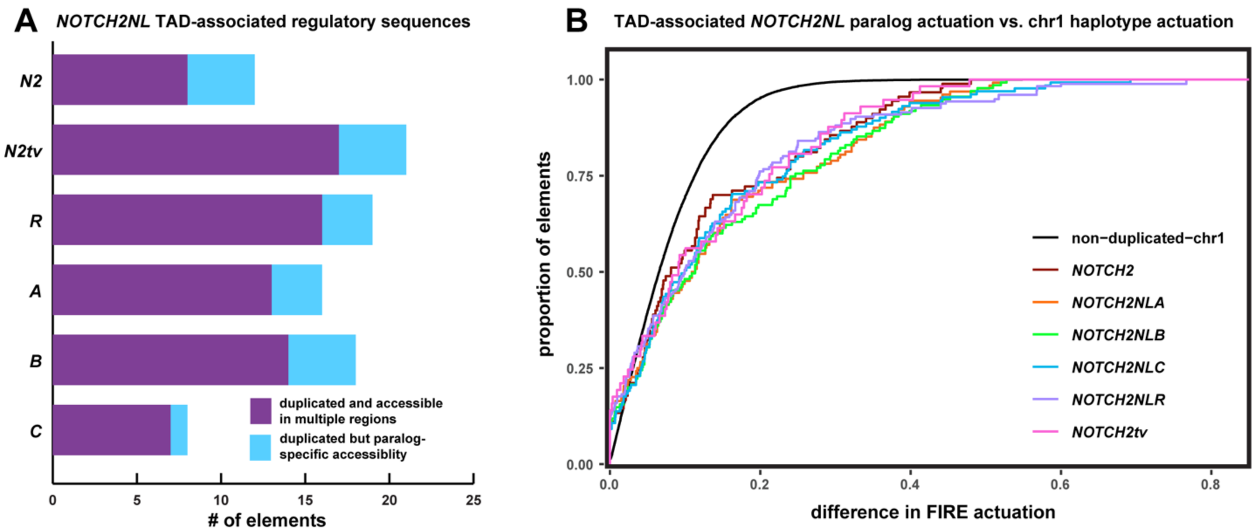

Figure S11. TAD-restricted regulatory analysis, related to Figure 6. A) Bar graph showing categorization of the TAD-restricted accessible elements surrounding each *NOTCH2NL* paralog based on the presence of duplicate sequence and accessibility at that sequence on the different paralogs.
B) A TAD-restricted model of expected haplotype differences on chromosome 1 using brain organoid data mapped to T2T-CHM13 (black) also shows a higher proportion of regulatory elements with less actuation difference than what is seen for the different *NOTCH2NL* paralogs.

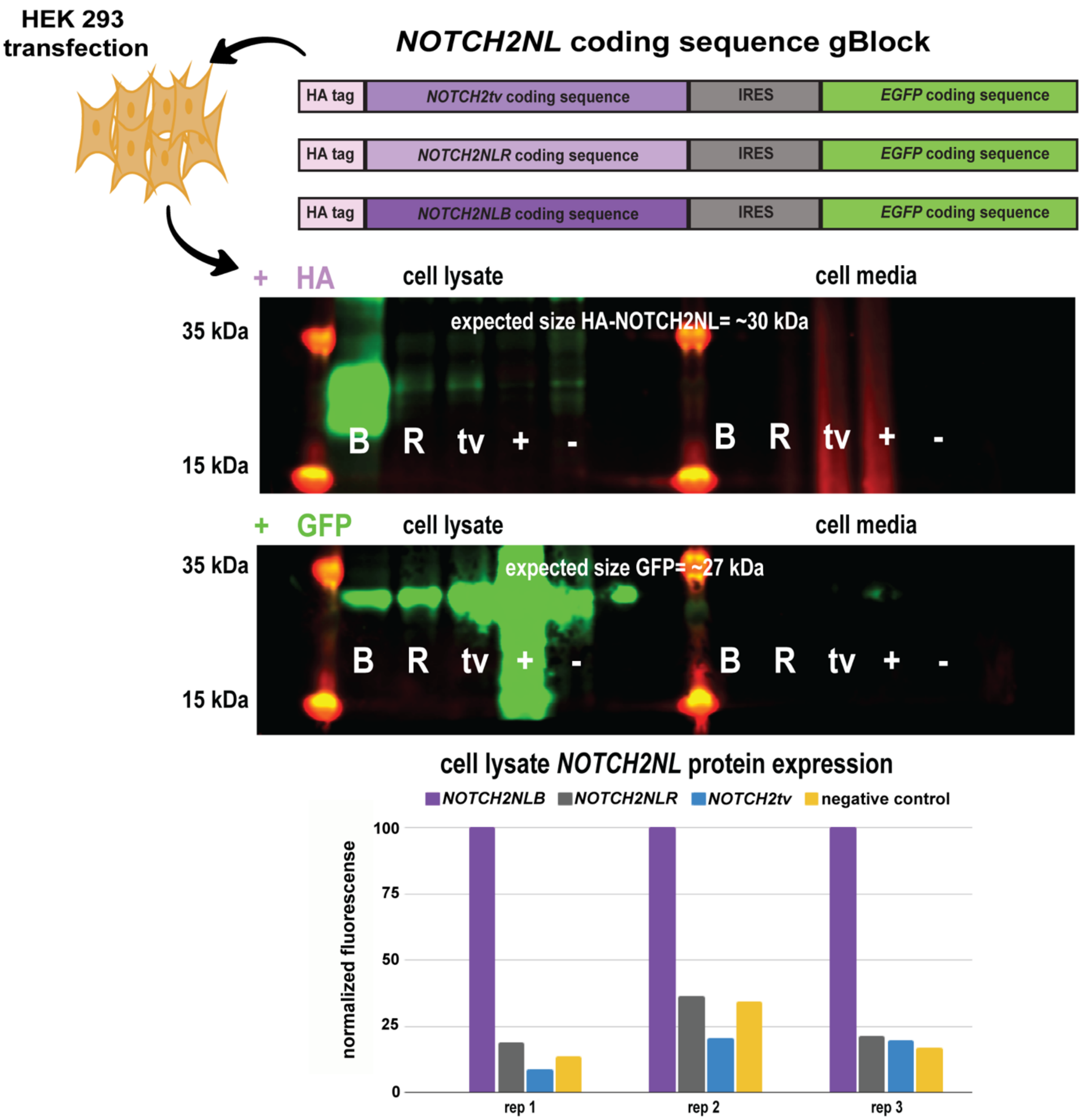

Figure S12. NOTCH2tv protein expression, related to STAR Methods. gBlocks containing an HA antibody tag, *NOTCH2NL* CDS, and *EGFP* CDS were cloned into vector DNA and transfected into HEK293 cells. Antibody staining for HA (NOTCH2NL) and GFP were done on both the cell lysate and media. HA expression fluorescence was normalized using GFP for cell lysate, which shows stable expression of NOTCH2NLB only.

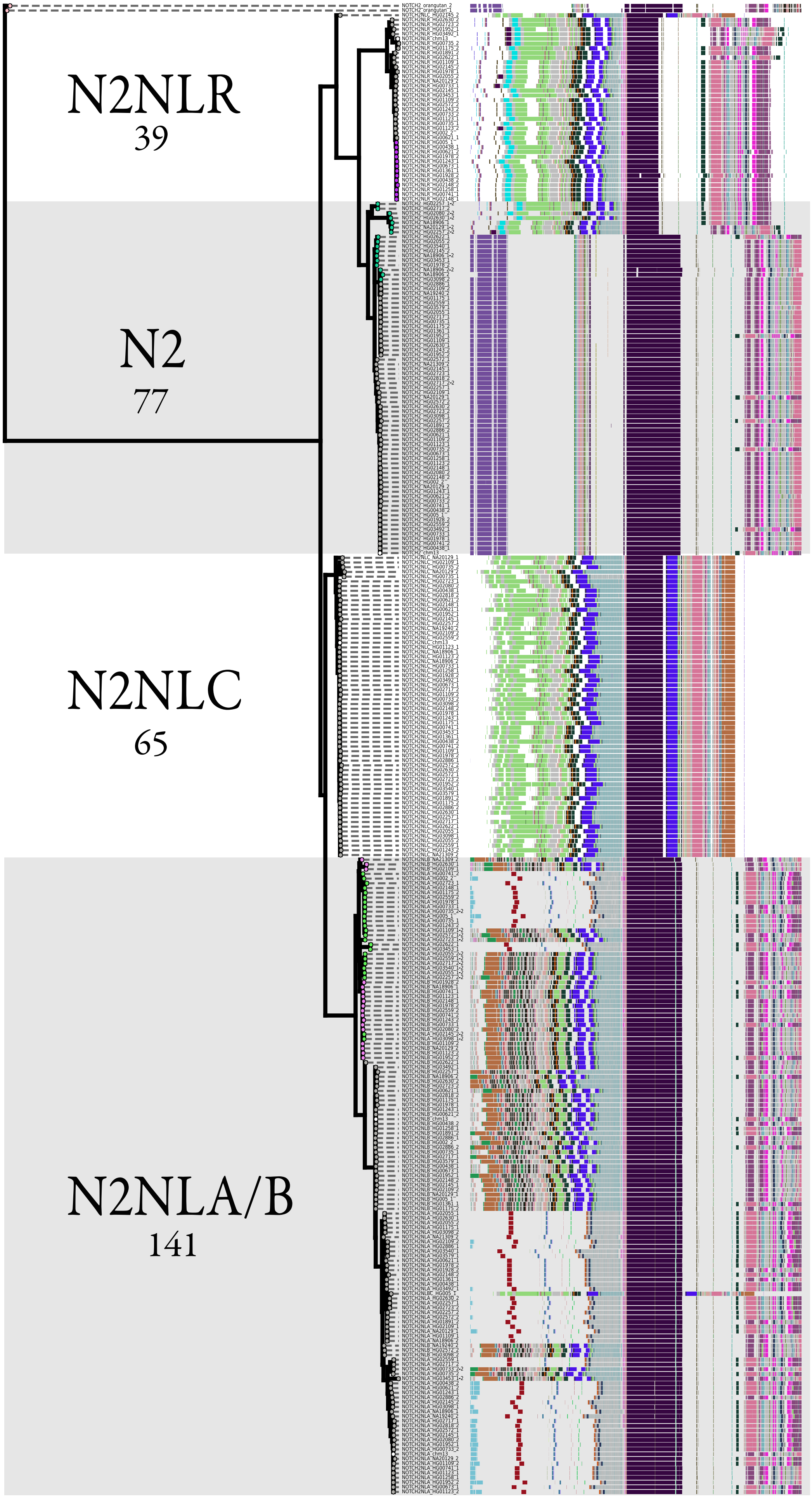
N2NLR
39
N2
77
N2NLC
65
N2NLA/B
141

Figure S13. Phylogeny of human *NOTCH2NL* genetic variation, related to Figures 4 and 5.
(figure on previous page)

SUPPLEMENTAL REFERENCES

[S1] Szamalek, J.M., Goidts, V., Cooper, D.N., Hameister, H., and Kehrer-Sawatzki, H. (2006). Characterization of the human lineage-specific pericentric inversion that distinguishes human chromosome 1 from the homologous chromosomes of the great apes. Hum. Genet. *120*, 126–138. https://doi.org/10.1007/s00439-006-0209-y.

[S2] Fiddes, I.T., Lodewijk, G.A., Mooring, M., Bosworth, C.M., Ewing, A.D., Mantalas, G.L., Novak, A.M., van den Bout, A., Bishara, A., Rosenkrantz, J.L., et al. (2018). Human-Specific NOTCH2NL Genes Affect Notch Signaling and Cortical Neurogenesis. Cell *173*, 1356-1369.e22. https://doi.org/10.1016/j.cell.2018.03.051.

[S3] Fiddes, I.T., Pollen, A.A., Davis, J.M., and Sikela, J.M. (2019). Paired involvement of human-specific Olduvai domains and NOTCH2NL genes in human brain evolution. Hum. Genet. *138*, 715–721. https://doi.org/10.1007/s00439-019-02018-4.
