# Supplementary material for: Genetic diversity and regulatory features of human-specific NOTCH2NL duplications
Source: Cell Genom. 2026 Mar 30;6(6):101194. doi: 10.1016/j.xgen.2026.101194 (PMC13261667; doi:10.1016/j.xgen.2026.101194)
Supplement: Data S1. Transparent peer review records for Real et al. [file mmc3.pdf]

Summary

Initial submission:	Received : 3/14/2025
	Scientific editors: Sara Rohban and Laura Zahn
First round of review:	Number of reviewers: 3<br>Revision invited : 4/29/2025<br>Revision received : 11/6/2025
Second round of review:	Number of reviewers: 3
Third round of review:	Number of reviewers:1<br>Accepted : 3/4/2026
Data freely available:	Yes
Code freely available:	Yes

This transparent peer review record is not systematically proofread, type-set, or edited. Special characters, formatting, and equations may fail to render properly. Standard procedural text within the editor's letters has been deleted for the sake of brevity, but all official correspondence specific to the manuscript has been preserved.

Referees' reports, first round of review

Reviewer #1: Real et al. tackle the evolutionary and functional impacts of a particularly challenging genomic region in primate genomes using an impressive collection of mostly previously published datasets. They present extensive analyses, ranging from deep cross-species comparisons to resolution of haplotypes in multiple human genomes, as well as functional analyses involving Iso-seq and Fiber-seq data. The utilization of high-quality genomes and recently available long-read datasets provides an exciting analytical framework and opens new avenues for future research. Thus, in principle, the manuscript is interesting, provides a valuable resource, and serves as a "how-to" guide for resolving complex genomic regions. Despite these strengths, I have several concerns that dampen the potential impact of the study:

1. The presentation of the results is challenging to follow. Admittedly, I am not an expert on this locus, and the variation presented is extensive, encompassing multiple levels (structural variation, splicing differences, SNV-indel variation, etc.). However, given the introduction of numerous datasets, multiple isoforms, different genes, and at least two genomic loci, the paper becomes difficult to navigate for a broad readership. Considering the intended audience of Cell Genomics, this issue needs addressing. Possible improvements include adding a glossary for gene names, additional figure panels summarizing key findings, and general revision of the manuscript to simplify the narrative and minimize confusion. As an example of vague statements, the first sentence of the abstract states: "...genes arose from incomplete, recent chromosome 1 segmental duplications..." It is unclear what "incomplete" or "recent" means in this context. Only in the main text can one infer that "recent" might mean human-specific and "incomplete" may mean pseudogenized. Such ambiguities must be clarified.

2. Several claims lack explicit empirical support, or, if evidence exists, it is not clearly articulated. This issue is not necessarily a deal-breaker, and empirical analysis may sometimes be challenging or impossible in such complex loci and there are many interesting observations presented with robust support. However, the limitations should be explicitly acknowledged. Concerns include details about the exact assembly of human haplotypes (I have confidence based on the authors' previous work, but how and why certain accuracy thresholds for structural assemblies were chosen are unclear), the actual extent of gene conversion events (the exact locations, how are they identified), exact locations of the breakpoints and the breakpoint contents for structural rearrangements, methods of determination, limitations of Iso-seq and Fiber-seq analyses, and clarification on how fusion versus incompleteness is determined lack enough detail. Ideally, each new finding or statement should ideally include statistical support (p-values, figures, or tables). As an example, two sentences starting with "Other than pseudogene copies, NOTCH2NLB appears to be..." should be clarified, as the basis for these conclusions is not clear.

3. How do the specific tissues or cell types used for RNA-seq and Fiber-seq data collection affect the results? Would additional tissues alter the findings? Are the current findings consistent with other databases (e.g., GTEx) regarding tissue specificity of expression among different paralogs? Additionally, how does the expression of incomplete copies compare to genome-wide averages?

4. Clarification is needed on how ancestral sequences for primate haplotypes were constructed. Are duplication ages calculated accurately using orangutan divergence for normalization, and what potential issues might arise (e.g., is the mutation rate for single nucleotide variants expected to be uniform across the genome)? What are the error margins associated with these estimates? I am particularly interested in the estimated coding sequence alignments—are there signatures of selection (negative or positive, dN/dS)? Is there an accumulation of loss-of-function or nonsynonymous mutations in the proposed pseudogenized copies? Are there regions showing greater conservation than expected by chance or drift?

5. The argument regarding trade-offs and selective forces presented in the conclusion is interesting. However, is there quantitative or model-based evidence supporting the assertion that genomic instability from gene duplications generates a significant fitness cost? If such studies exist, they should be cited.

6. The term "functional copies" should be clearly defined. Does this refer specifically to protein-coding copies? If so, "protein-coding copies" would be a more accurate term. Indeed, the authors' own previous work has shown that long non-coding RNAs derived from incomplete duplications can have important functional roles and evolutionary impacts.

7. The term "first unique genes" in the figure legend of Figure 1 is unclear, representing another example of the vague expressions present throughout the manuscript.

Reviewer #2: The recurrent incomplete duplications of the NOTCH2/NOTCH2NL genes are an illuminating example of a human-specific evolutionary change that contributed to the adaptation of a hallmark human feature - our large brain. By integrating long-read sequencing data generated from both non-human apes and individuals of diverse human populations, the authors uncover the timing, order, and structure of these duplications. They further reveal that the copy number of various NOTCH2NL paralogs varies within humans. Through investigation of the regulatory features and expression patterns of these paralogs, the authors attempt to shed light on their functionality and suggest selective constraint.

Major points
From my perspective, the study of NOTCH2/NOTCH2NL has the potential to deepen our understanding in three key ways: (a) shedding light on the role and function of these genes in human evolution, (b) serving as a case study to understand the broader mechanisms and impacts of SDs, and (c) introducing novel approaches and tools to advance the study of SDs. While the authors conduct thorough and impressive analyses of these loci, resulting in a high-resolution map of their evolutionary history, I am not convinced that their findings advance any of the three aspects mentioned above in a meaningful way, especially in light of the findings of the Fiddes et al. 2018 paper.
More specifically, it remains unclear whether the various NOTCH2NL paralogs and their combinations (haplotypes) truly differ in function and phenotypic impact. The authors propose that NOTCH2NLA may be particularly important, given its fixation in multiple haplotypes. To support this claim—as well as the idea that NOTCH2NLA repeatedly serves as the preferred donor locus—an ancestry-aware analysis is warranted, rather than just counting the number of individuals with each haplotype. I suggest examining whether the losses and biased conversions are recurrent events versus arising from a single origin, which would help clarify whether these patterns truly indicate biased directions or simply reflect one ancestral event.
The authors put a special emphasis on mapping the regulatory differences of these loci. While this is an important direction, the analyses fall short of providing meaningful insight into regulatory evolution and differences.
Given that these are large duplications that on one hand included the potential regulatory elements of these genes, but on the other hand, now exist in a new genomic context, one could expect both similarities and differences from the original chromatin pattern. The authors describe both but do not quantify whether these similarities and differences are more or less than expected by chance. Moreover, given the observation of transcription from these paralogs, the accessibility of their promoters is again expected. Thus, it is unclear what these comparisons teach us.
The authors put a special emphasis on the number of peaks around these paralogs (503-634). Most studies estimate fewer than 10 regulatory elements per gene. Thus, the vast majority of these peaks (>98%) are expected to be non-functional and the focus on the number of peaks has little meaning. This is further exacerbated by the fact that only two samples are used for this, and thus, the differences observed could simply reflect transient dynamics, power differences or noise. To identify the likely regulatory elements of these genes, and to gain insight into the evolutionary differences that are of potential importance, more in-depth analyses of these loci are called for. I acknowledge the fact that given the nature of these loci, reliable chromatin maps are hard to find/generate, and I appreciate the use of Fiber-seq. However, without further regulatory data, such as histone modification marks, TF binding, reporter assays, 3D contact maps etc, there is not much one can conclude from the data.
I acknowledge that the findings of the authors are complex and challenging to visualize. However, the figures are currently unclear and lacking important information. The first sections of the text describe a very long list of genetic changes between the lineages. While these findings are important, they are very hard to follow and could be described much more succinctly

and largely replaced with clearer figures. More specifically: (a) The paper would especially benefit from a timecourse fig depicting the full story of duplications and conversions. The current figs show some of these aspects, but a summary fig is missing, and (b) Many figs are hard to follow, packed, and visually messy.

Minor points

The introduction is very clear and covers the key points. The only thing missing there, in my opinion, is a clearer presentation of the motivation and goal of the study. The authors describe the importance of NOTCH2 and NOTCH2NL in evolution and disease, and the challenges in studying SDs, but I think readers would benefit from a sentence summarizing precisely how solving these challenges would advance our understanding of human evolution and disease.

"NOTCH2NLC appears the most structurally divergent; most paralogs share the bulk of synteny upstream of the gene model, yet the opposite is true for NOTCH2NLC, which has the most homologous alignments with NOTCH2NLR." - What is the point the authors are trying to convey here? That C is most homologous to R or that its synteny is mainly downstream as opposed to upstream? In addition, according to Table 1, the lowest pairwise homology is actually with NOTCH2NLR. Figure 3 also shows that C is closer to A and B than it is to R or NOTCH2.

"Though the difference in average SD lengths between NHA and human (311 kbp) is not significant (p=0.21; t-test one-sided)..." - Are they checking the overall difference in size or specifically if NHA SDs are shorter? If it's the former, it should be a two-sided test, if it's latter, it should be one-sided and the text should be changed accordingly. In any event, it is not clear what the authors aim to achieve by conducting this test. Is it to determine whether there is a common source for these SDs?

"Subsequently, in the human lineage, this region reverted to its ancestral configuration (inversion II) but expanded in size. We estimate that this region is 17 Mbp larger than the syntenic region in chimpanzee and orangutan." - Do the authors mean to refer to inversion III here? Because according to figure 2, it does not seem as if inversion II has an addition of this size.

"In addition to the inversions, structural variants, including 308-829 insertions and 312-816 deletions (>50 bp), were observed across the NHAs (Supplementary Table 2), significantly enriched at the breakpoints of inversions (p<0.05), in chimpanzee, bonobo, and gorilla (Supplementary Table 2). Assessing the region in terms of duplicated sequences, we also found that the locus is enriched with SDs (p<0.001, in T2T-CHM13 space);" - p-values are stated but not the statistical test/s that were used.

"All species harbor four shared NOTCH2NL homologs..." - According to figure 2, seems like orangutans do not.

"In contrast, all the NHA NBPF genes have a high copy number of the CON1, CON2 and CON3 Olduvai domains." - What is considered high? Many have 3, which doesn't seem like a lot in comparison to others and in comparison to HLS copy number in humans.

"While most transcripts predict fusions of NOTCH2NL with other genes (24/26), some transcripts (14/26) predict proteins most similar to human NOTCH2NLR-like sequence with ORF lengths ranging between 235-246 amino acids (AAs). These predicted proteins are similar (p=0.1; t-test one-sided) to human NOTCH2NL ORF lengths (236-274 AAs) (Supplementary Table 3)." - Why do the authors propose the test should be one-sided? In addition, do the authors have sufficient statistical power for this test? (sample size of the human group contains 5 observations). Most importantly, a non-significant P-value does not mean that H0 is accepted ("these proteins are similar"), only that H0 cannot be rejected. Moreover, according to Table 3, PTR-NOTCH2NL-5 is predicted to be 146 aa long. In another issue, is the use of the word "like" needed here?

"We confirmed that these SDs and gene copies are present in the alternate haplotype of each NHA individual with a small amount of expected copy number variation due to the complexity and large number of duplications in the region (n=9 in chimpanzee, n=9 in bonobo, and n=9 in gorilla)." - It is not clear to me what these numbers represent. Is it the number of different NOTCH2NL SDs in African great apes? If yes, these numbers don't sum up to 26 and differ from the numbers stated earlier in the text.

"The vast majority (19/26) of these NHA NOTCH2NL-like loci create fusion transcripts between NOTCH2NL at the 5' end and NBPF at the 3' end (Figure 3B), which is similar to what is seen in all human paralogs..." - But in humans it seems as if NOTCH2NL is fused at its 3' to NBPF's 5' (opposite fusion).

The authors use the terms NOTCH2NL-like and NOTCH2NLR-like interchangeably and it's not clear whether the latter is used purposely to refer to a subset of the NOTCH2NL-like transcripts.

"H2, H4, H8, and H9 configurations strictly represent deletion events, which occur in 30% (21/69) of haploid assemblies..." - Seems like there are 23 total individuals in these 4 populations, making it 23/69=33%.

"with the majority arising from fusion transcripts, and an incorrectly spliced d exon." - Is the "d" here purposely? If yes, then I'm not sure this sentence is clear.

"The biased gene conversion that appears to be driving the fixation of NOTCH2NLA and the high level of fourth intron retention among transcripts of NOTCH2tv and NOTCH2NLR may be examples of both refinement and continued evolution of a novel carboxy terminus." - What do the authors mean by "refinement"? And what do they mean by "novel carboxy terminus", if it has been shown that these two paralogs are pseudogenes?

Figure 1B - It is not clear what the colors represent.

Figure 1C - What is the meaning of the direction of the long black arrows that are within the gene? Is it its direction? According to the CHM13-T2T assembly on the genome browser, the NBPF gene is downstream of the NOTCH2NLA gene.

Figure 2 - What is the meaning of the "N" in the dark red circle? In addition, the figure is a little overwhelming. I suggest removing the NBPF circles, and possibly the red & green arrows.

Figure 3B - It is not always clear which gene name corresponds to which gene schematic.
And if NBPF is fused at its 3' end to NOTCH2's 5' end, then shouldn't the ORF start and end be reversed? And what is the size of each window? Units should be added to the right bar plot. And what is the meaning of the right bar plot if each bar represents a different number of genes? And the use of the exact same colors as in A, when there's no connection between the two, can be confusing.

Figure 4B - I think this is better suited as a supplementary figure.

Figure 5A - How do these configurations distribute among the various populations?
In the figure text - "A simplified schematic summary of the NOTCH2NL haplotype organization and frequency based on 66 sequence-resolved HPRC genomes and the T2T-CHM13 reference." - Isn't it 69 though?

Reviewer #3: We found the manuscript, "Genetic diversity of regulatory features of human-specific NOTCH2NL duplications," to be an informative and enjoyable read. This manuscript represents a thorough analysis of the gene family containing NOTCH2NL, using a variety of recently published and nearly complete genomes from the non-human apes, and scores of humans. Additionally, the authors use Fiber-seq to annotate regulatory elements in the paralogs of this family, which could not be done efficiently with previous methods. The authors: find that this family independently expanded in great ape lineages, discovered a new member of the family (NOTCH2tv), and showed that the degree of open chromatin over a regulatory element is often variable across paralogs in the family.

Our overall enthusiasm for this work is high, but we do have some comments that we think should be addressed in a revised version of the manuscript.

Comments:
1) As mentioned in the manuscript (Figure 6), the different NOTCH2 paralogs have very different transcript abundances, with NLA and NLB being the highest. However, all of the NOTCH paralogs

have similar numbers of open chromatin regions in the Fiber-seq data. Can you speculate on why this might be (e.g. mixture of enhancers and repressors, some regulatory elements don't act on a NOTCH2 paralog)?
2) It is not clear from figure 1B how the locus barcoding works.
3) It appears by cross-referencing Figure 3B and Figure 6C, that NHA NOTCH2 paralog transcripts are fusion transcripts more often than human NOTCH2 paralog transcripts?
4) On page 9, the authors reference Figure 3B to make the point that either exon 1 or 2 of the NOTCH2NL transcripts are lost. Perhaps the authors should reference Figure 3C here instead?
5) The start of the results section describes the similarities and differences between the genetic structure of the paralogs. This is well described using figures and tables, and the authors can likely make this part of the text more concise.
6) Is it possible to speculate on the function/consequence of the fusion proteins between NOTCH2NL and LRIG2 or SORT1?
7) When the authors state that polarized signals of gene conversion suggest selection, is the idea that gene conversion is likely to be happening in both directions, but we only observe one direction because one is deleterious/advantageous? If so, perhaps that could be explicitly stated.

Authors' response to the first round of review

Reviewer #1: Real et al. tackle the evolutionary and functional impacts of a particularly challenging genomic region in primate genomes using an impressive collection of mostly previously published datasets. They present extensive analyses, ranging from deep cross-species comparisons to resolution of haplotypes in multiple human genomes, as well as functional analyses involving Iso-seq and Fiber-seq data. The utilization of high-quality genomes and recently available long-read datasets provides an exciting analytical framework and opens new avenues for future research. Thus, in principle, the manuscript is interesting, provides a valuable resource, and serves as a "how-to" guide for resolving complex genomic regions. Despite these strengths, I have several concerns that dampen the potential impact of the study:

We'd like to thank Reviewer #1 for thoroughly reviewing our manuscript and commenting on the strengths as well as providing constructive feedback on how to strengthen our data presentation and analysis. We have edited the manuscript to accommodate their feedback.

1. The presentation of the results is challenging to follow. Admittedly, I am not an expert on this locus, and the variation presented is extensive, encompassing multiple levels (structural variation, splicing differences, SNV-indel variation, etc.). However, given the introduction of numerous datasets, multiple isoforms, different genes, and at least two genomic loci, the paper becomes difficult to navigate for a broad readership. Considering the intended audience of Cell Genomics, this issue needs addressing. Possible improvements include adding a glossary for gene names, additional figure panels summarizing key findings, and general revision of the manuscript to simplify the narrative and minimize confusion. As an example of vague statements, the first sentence of the abstract states: "...genes arose from incomplete, recent chromosome 1 segmental duplications..." It is unclear what "incomplete" or "recent" means in this context. Only in the main text can one infer that "recent" might mean human-specific and "incomplete" may mean pseudogenized. Such ambiguities must be clarified.

We carefully reviewed the manuscript, removed or defined jargon where possible, streamlined the text, and added additional figures where appropriate to help guide readers, including a summary figure and additional section predicting TADs using FiberFold (see below). We edited the manuscript to be more specific adjusting vague statements such as:

Edited:

"NOTCH2NL (NOTCH2-N-terminus-like) genes arose from ape-specific chromosome 1 segmental duplications implicated in human brain cortical expansion that included an incomplete NOTCH2 gene."

Based on the referee's suggestion, we also prepared the following glossary and added it to the Supplementary materials:

GLOSSARY

Brain organoid: 3-D, stem-cell derived, "organ-like" tissue of the dorsal forebrain

Breakpoint: specific region of the genome/chromosome that is mediating a site of (often recurrent) genome rearrangement (see: rearrangement)

DUF1220 domain: domain of unknown function 1220 is a tandem repeat of sequences embedded within

the NBPF genes that encodes ~65 amino acids; it shows one of the largest human-specific copy number increases when compared to apes (also referred to as the Olduvai domain)
Duplicon: a chromosome-specific duplicated sequence whose ancestral origin can be defined usually based on comparison to outgroup species where the sequence exists as a single copy
Response to Reviewers
2
Core duplicon: duplicon that is significantly overrepresented in a repeat-graph constructed by human SDs and thought to represent the focal point for the accumulation of larger SDs made up of many duplicons; core duplicons are frequently reused in other primate lineages but have different flanking SDs and map to unique locations.
Duplicon "barcode": a computational visualization of the duplicon content and organization in a specific SD region, the patterning of which can be used to distinguish the long-range higher order structure of complex SDs and therefore useful for classifying the structure of the locus
Fusion transcript: chimeric RNA transcript resulting from two or more gene bodies that are duplicated and juxtaposed resulting in a novel transcript product predicted to create a unique protein
Haploid genome assembly: Either the paternal or maternal (1n) assembly from a diploid (2n) individual
Haplotype configuration: set of (in this study) NOTCH2NL paralogs inherited together from each haplotype-phased genome assembly
Homologs: genes shared among species that possess a common ancestral origin
Interlocus gene conversion (IGC): nonreciprocal transfer of donor sequence to paralogous acceptor locus thought to be mediated by double-stranded break repair mechanisms
Non-allelic homologous recombination (NAHR): unequal crossing over caused by misalignment of and recombination between paralogous sequences, leading to genomic rearrangements (see rearrangement)
NBPF: neuroblastoma breakpoint family (NBPF)—a recently expanded gene family that was dispersed by SDs primarily on human chromosome 1 and subsequently classified as a core duplicon (see core duplicon); the function of the gene family is unknown although it was originally discovered as expressed in neuroblastoma genes and implicated in neurodevelopmental and neuropsychiatric disease (see DUF1220)
NOTCH2: conserved ancestral gene that encodes a Notch receptor expressed on the surface of cells; important for signaling in mammals
NOTCH2NL: NOTCH2-n-terminus-like (NOTCH2NL)—genes whose first four exons (in humans) are highly homologous to NOTCH2 and encode for the signal sequence and first few EGF-like repeats of the NOTCH2 extracellular domain; includes only the NOTCH2NL paralogs when referring to the NOTCH2NL family
NOTCH2/NL: includes ancestral NOTCH2 and incomplete NOTCH2NL duplicates
NOTCH2NLA-C: presumed functional human-specific duplicates
NOTCH2NLR: presumed nonfunctional/pseudogene duplicate that may not be human-specific
NOTCH2tv: gene conversion product from a NOTCH2NLR to NOTCH2 event
Paralogs: homologs created through gene duplication
Rearrangement: typically larger structural variants that rearrange long-range order or structure of a sequence through duplication, deletion, translocation, and inversion often mediated by NAHR between repeat sequences
Segmental duplication (SD): duplicated sequence that is >1 kbp and >90% identical within a species
Structural variation: a form of genetic variation involving deletion, insertion, duplication, translocation or inversion where events are 50 bp, distinguished from SNVs and insertions/deletions <50 bp
Synteny: presence of genes or loci on the same chromosome in different species based on shared common ancestry
3

2. Several claims lack explicit empirical support, or, if evidence exists, it is not clearly articulated. This issue is not necessarily a deal-breaker, and empirical analysis may sometimes be challenging or impossible in such complex loci and there are many interesting observations presented with robust support. However, the limitations should be explicitly acknowledged. Concerns include details about the exact assembly of human haplotypes (I have confidence based on the authors' previous work, but how and why certain accuracy thresholds for structural assemblies were chosen are unclear), the actual extent of gene conversion events (the exact locations, how are they identified), exact locations of the

breakpoints and the breakpoint contents for structural rearrangements, methods of determination, limitations of Iso-seq and Fiber-seq analyses, and clarification on how fusion versus incompleteness is determined lack enough detail. Ideally, each new finding or statement should ideally include statistical support (p-values, figures, or tables). As an example, two sentences starting with "Other than pseudogene copies, NOTCH2NLB appears to be..." should be clarified, as the basis for these conclusions is not clear.

The reviewer raised several important points, and we agree more detail regarding QC, how we defined breakpoints, mapped Iso-Seq, performed Fiber-seq, and defined fusion genes were needed. We discuss each of the points in more detail below, including caveats and limitations, and add empirical data wherever possible to support claims made in the main text.

As an example, we edited the statement "Other than pseudogene copies, NOTCH2NLB appears to be...", for clarification, provided more detail regarding the thresholds for validated structural assemblies in the methods section, and added a section regarding limitations:

Edited:

"Other than NOTCH2NLR, a likely pseudogene, NOTCH2NLB appears to be absent from human haplotypes based on sequence homology due to IGC between NOTCH2NLA and NOTCH2NLB. As a result, only 46 out of the expected 69 haplotypes actually carry it."

We implemented a rigorous set of criteria to assess both collapses and misassemblies and if a contig or haplotype failed these tests it was excluded from further analysis. We added these details to the assembly validation section:

Edited:

"Assembly validation of NOTCH2NL regions in HPRC assemblies

Except for T2T-CHM13, the human assemblies used in this study were originally released as a part of HPRC year 1 (Liao et al. 2023) without complete validation of every region. These assemblies are available under BioProject ID: PRJNA730822. To validate our set of assemblies, we first confirmed the correct assembly of contiguous sequence between NOTCH2NL paralogs on the same chromosome arms. This was done by evaluating the number of copies in each assembly and how many contigs they covered using rustybam (mrvollger.github.io/rustybam/). Assemblies with copies across three or more contigs were removed and those with no more than two contigs (allowing for gaps across the centromere) were assessed with NucFreq (Vollger et al. 2022). Assemblies with collapses (excess of secondary bases in a single haplotype assembly) within the NOTCH2NL gene, collapses greater than 10 kbp outside the NOTCH2NL gene, gaps (low-quality, N-based sequence) within the NOTCH2NL gene, and gaps greater than 10 kbp outside the NOTCH2NL gene over the 1 Mbp region surrounding each NOTCH2NL paralog were considered incorrectly assembled. Finally, assemblies were also removed from the completed set if they were missing specific unique gene marks outside NOTCH2NL SD regions, which are used as anchors for rearrangement boundaries. Deletions were further validated using fastCN (Pendelton et al. 2018) read depth. A subset of incorrectly assembled samples was attempted to

be rescued using Verkko (1.1 and 1.2) (Rautiainen et al. 2023) and added to the validated set: 69/94 haploid assemblies in total were correctly assembled and validated."

We also added a section to the end of the discussion explicitly acknowledging limitations or caveats and their potential impact on our overall analyses:

"LIMITATIONS

Gene conversion biases. Gene conversion exists at varying degrees throughout this locus, even in T2TCHM13, which is used as a reference and may be a source of error for analysis of human genetic variation. Although we attempted to identify the largest gene conversion events, both reference effects and smaller events may remain undetected. IGC obscures timing of duplication events and signals of selection. Additionally, homogenization between highly similar loci where only a few SNV differences distinguishes paralogous segments complicates breakpoint definition.

Phylogeny & duplication timing. Although we assess robustness of the phylogenies using bootstrap support, several sources of error remain. The largest source is the timing of speciation events used to calibrate the molecular clock, which are estimates themselves with relatively wide upper and lower bounds. As noted previously, IGC is also a concern, as any duplication timing estimates we report may capture time since gene conversion versus time since the original duplication event.

Gene regulation and Fiber-seq. Fiber-seq is a measure of chromatin accessibility, which is only a proxy

for noncoding regulatory regions; it does not distinguish between element types, such as repressors and enhancers, nor address which genes these elements would be regulating. Although FiberFold will generate predictions of which genes may be regulated by these accessible chromatin peaks, further efforts are needed to functionally characterize these predictions.

Iso-Seq analyses. Iso-Seq is a measure of steady state transcripts, which is influenced by both the potency of regulatory elements as well as the stability of the full-length transcript. Given the marked divergence between NOTCH2NL paralogs in terms of the surrounding regulatory elements as well as their 3' UTR structure, it is anticipated that both of these are contributing to the final steady state measurements of transcript abundance."

With respect to interlocus gene conversion (IGC) and breakpoints, IGC segments were detected using our workflow for determining NOTCH2NL identity, which is detailed in the Methods. Specifically, when we see conflicts between the clade grouping, the mapping location, and/or the coding sequence identity, we consider it evidence for gene conversion. However, we condition on IGC which affects protein-coding sequence of NOTCH2NL, then considered all aspects as described above. At the protein level, the boundaries of these events were confirmed using an MSA, and this is shown in Figure 5B for NOTCH2NLR to NOTCH2tv events. We included a visual here of NOTCH2NLB to NOTCH2NLA because it represents a single amino acid change (Review Figure 1). We added some additional details to the Methods to clarify:

Edited:

"Determining NOTCH2NL identity and uncovering IGC in HPRC assemblies

To determine NOTCH2NL identity we took a three-pronged approach: (1) identifying which T2T-CHM13 NOTCH2NL CDS reference or 'transcript' best matches the NOTCH2NL sequence being queried using BLAT; (2) identifying the phylogenetic clade the NOTCH2NL sequence being queried best groups with—sequences from intron 2 of NOTCH2/NL from all samples queried were used to construct an MSA using MAFFT, from which an ML phylogeny was built using IQ-TREE2; and (3) identifying the mapping location

of the NOTCH2NL sequence being queried using the greater NOTCH2NL region DupMasker barcode—NOTCH2NL intron 2 sequences plus 1 Mbp surrounding each intron were used as input for DupMasker and duplicon barcodes for each sequence were added to the tree. IGC events were first found through conflicts between the coding sequence identity, clade grouping, and/or mapping location. A final visualization of protein-coding changes caused by gene conversion can be confirmed using MSA software."

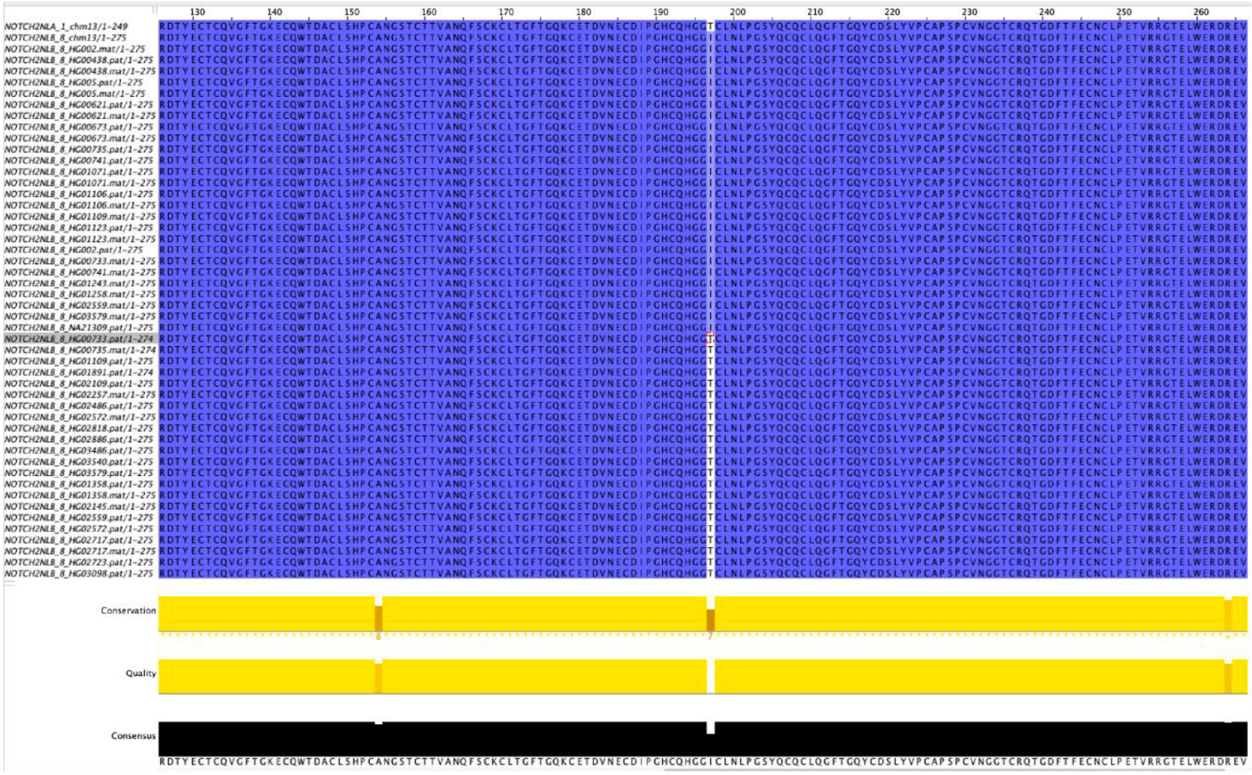

Review Figure 1. MSA of reference NOTCH2NLA and NOTCH2NLB predicted proteins aligned to predicted protein from every NOTCH2NLB mapping location across the validated HPRC sample list. While there may be a few other amino acid changes segregating in the population, only this single amino acid site defines gene conversion between NOTCH2NLA and NOTCH2NLB.

In an attempt to further refine the breakpoints, we focused on a more detailed analysis of NOTCH2NLB to NOTCH2NLA gene conversion events. For this, we created two phylogenies, one 50 kbp upstream of the gene region (from the point of NOTCH2NLA/B conversion) and one 50 kbp downstream of the gene region of NOTCH2NL. The two regions show slightly different tree topologies (Review Figure 2), including polyphyletic clades for gene conversions. However, only one group reaches significance for bootstrap support. Further, visual alignments of these regions from samples that showed gene conversion and ones that were not predicted to have gene conversion failed to define the boundaries of the breakpoints (Review Figure 3). The lack of sequence divergence makes it impossible to refine further in this case.

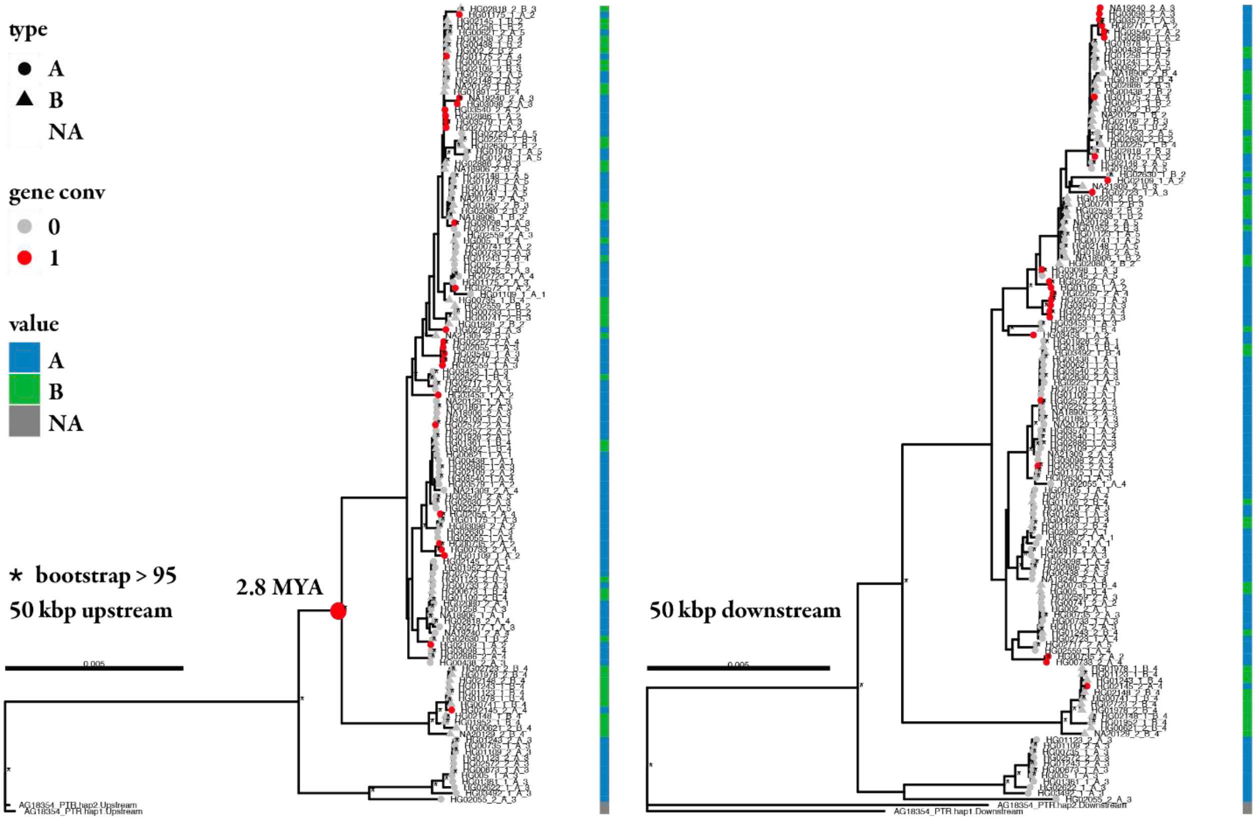

Review Figure 2. Phylogenetic reconstruction 50 kbp upstream and downstream of the NOTCH2NL genes. Shape icons represent the original mapping location identity of NOTCH2NLA (circle) and NOTCH2NLB (triangle) genes. Icon colors represent likely unconverted sequences (gray) and those with CDS-altering gene conversion events (red). Labels represent the best CDS match.

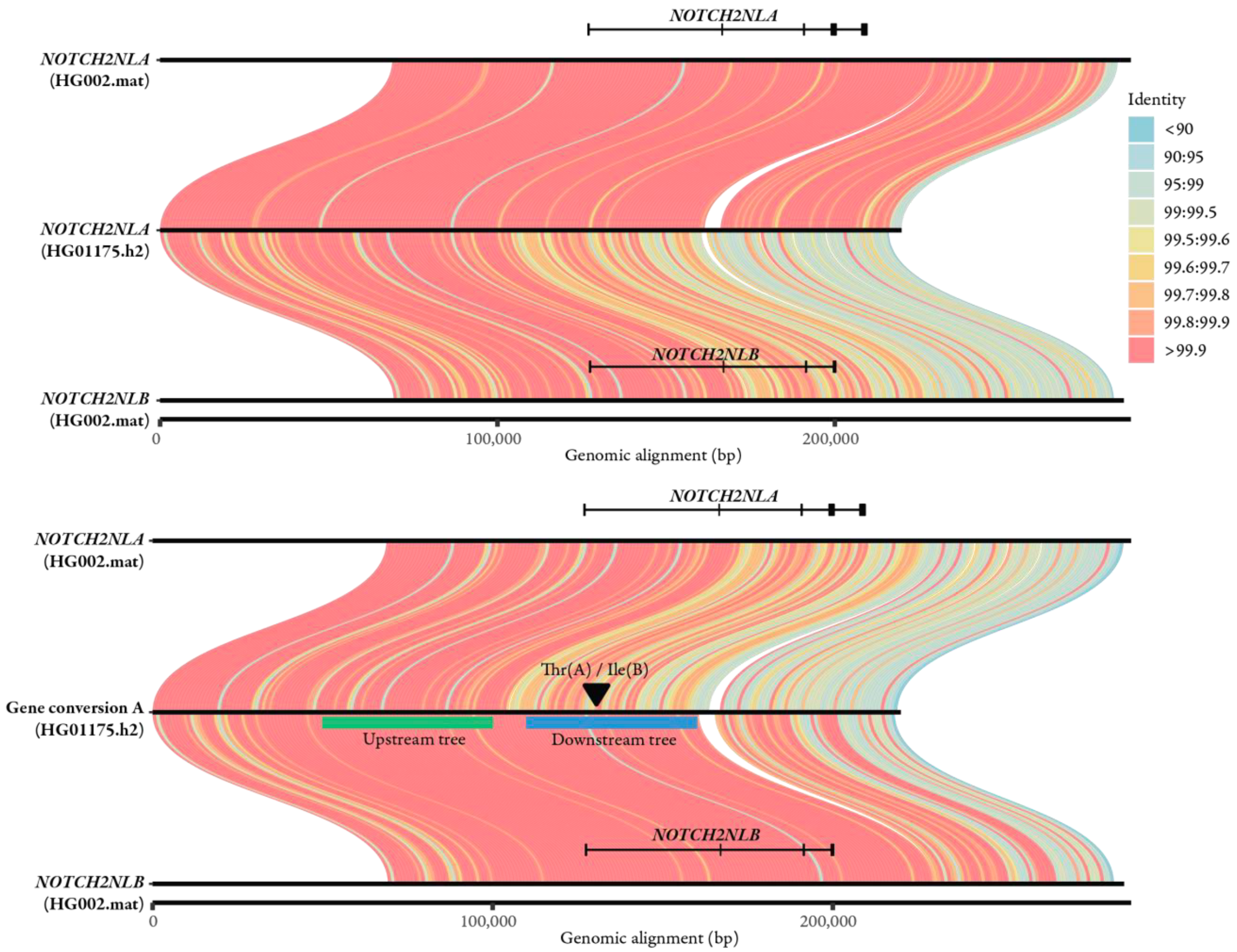

Review Figure 3. Visual alignments between non-converted and gene-converted NOTCH2NLA and NOTCH2NLB paralog regions. Transition from high-identity to low-identity regions can be readibly observed (heatmap), yet breakpoint definition is unclear. Upstream and downstream tree labels correspond to regions analyzed in Review Figure 2.

Additionally, we updated our gene conversion analysis because the original analysis included in the manuscript used CDS from the UCSC Genome Browser, which does not correct for the nonstandard CTG initiation NOTCH2NL paralogs A and C are predicted to have (see Structure of the NOTCH2NL gene family in a complete human genome assembly). Due to coding sequence mismatches that likely obscured some of the gene conversion signals, we reanalyzed with the corrected CDS. This had no effect on the classification of NOTCH2NLR/NOTCH2tv events but did increase the instance of NOTCH2NLB to NOTCH2NLA conversion from 14 events to 23 events (23/69, 33%). This also increased the total configurations to 11 (Figure 5A), as well as altered the total counts of particular haplotype configurations. Interestingly, we find that out of 23 total NOTCH2NLB to NOTCH2NLA gene conversion events, the majority are found in African assemblies (19/23, 83%) compared to non-Africans. This difference appears statistically significant (p=0.0008, Fisher's exact test). This suggests the NOTCH2NLB to NOTCH2NLA conversion is likely an ancient event or occurred more recently after separation of African and out-of-Africa populations. What we can conclude from phylogenetic analysis (Review Figure 2) is that NOTCH2NLB to NOTCH2NLA IGC occurred ~2.8 MYA. This is interesting because this timing corresponds to when the human-specific NOTCH2NLC copy emerged.

8

In addition to moving Figure 5C to the supplement (now Figure S7, shifting the figures that follow), we edited the manuscript and Figure 5 to reflect haplotype-configuration quantification changes due to gene conversion and CDS correction, particularly in these instances:

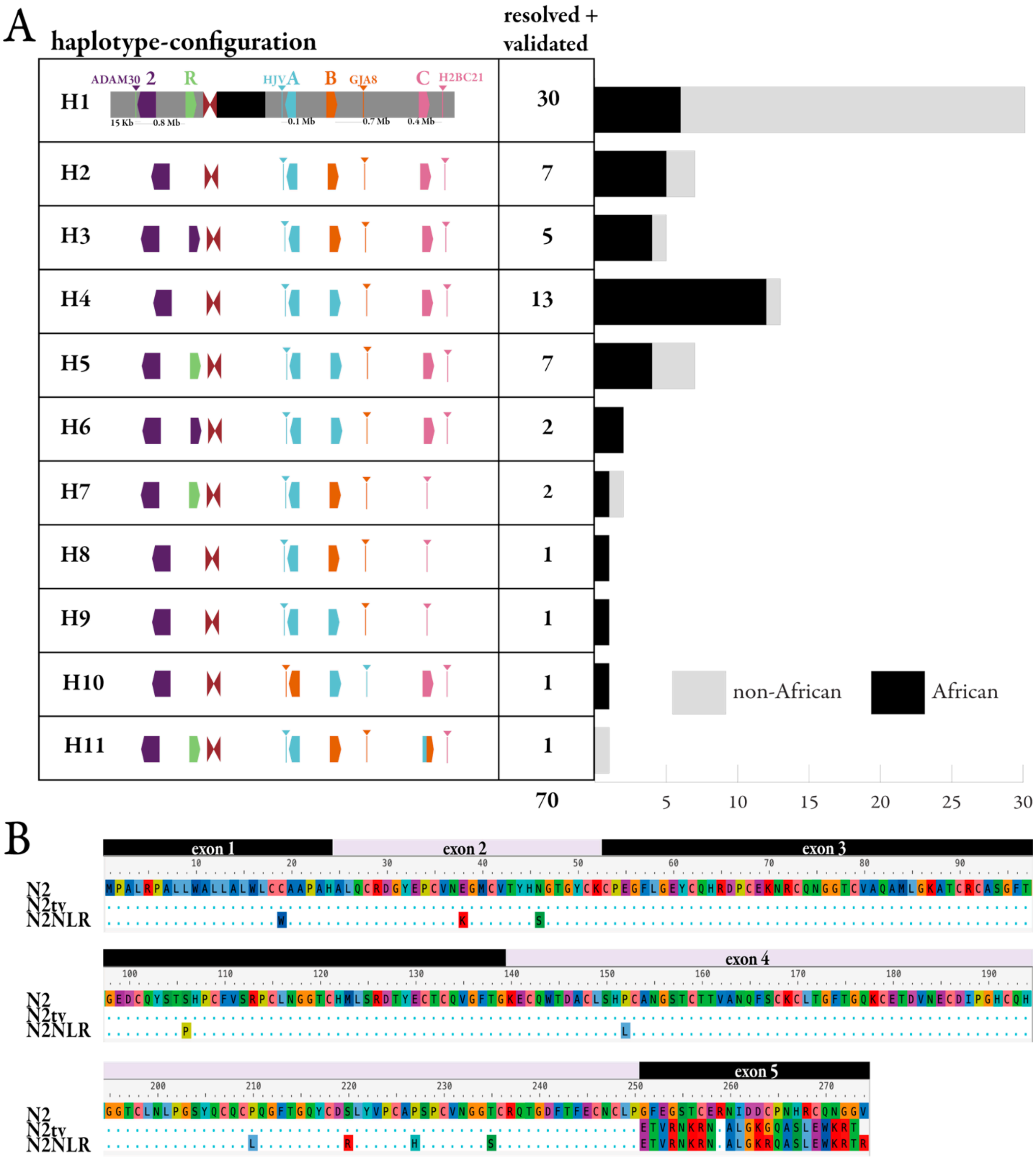

Updated Figure 5. NOTCH2NL structural diversity and NOTCH2tv. a) A simplified schematic summary of the NOTCH2NL haplotype organization and frequency based on 69 sequence-resolved HPRC genomes and the T2T-CHM13 reference. Proportion of African and non-African samples associated with each haplotype configuration suggests some may be ancestral. b) Alignment of predicted AAs for the three

9

paralogs suggests that NOTCH2tv arose as a result of an interlocus gene conversion (IGC) of NOTCH2NLR from NOTCH2.

Edited:

"Among these 69 genomes, we distinguished 11 different structural configurations operationally defining

H1 based on the T2T-CHM13 reference configuration described above (70 haplotypes in total)."
Edited:
"The combination of these results (Figure 4B) was used to delineate IGC events and to further define 11 distinct human haplotype configurations (H1-11) (Figure 5A)."
Edited:
"Of the haplotype-resolved genomes, 42% share the canonical haplotype configuration (H1) observed in T2T-CHM13, thus, representing the major human haplotype (Figure 5A). Among the remaining ten configurations, six are observed more than once in this subset from the HPRC (Figure 5A)."
Edited:
"Our analysis indicates that the NOTCH2NLR pseudogene is not present in 43% (30/69) of all haploid assemblies (H2-4, H6, and H8-10). H2, H4, and H8-H10 configurations strictly represent deletion events, which occur in 33% (23/69) of haploid assemblies."
**Also edited for comment by Reviewer #2*
Edited:
"NOTCH2NLC is deleted in four haploid assemblies with three different configurations (H7-9) while in another (H11) it appears to have been converted to a NOTCH2NLA/B hybrid."
Edited:
"For example, a NOTCH2NL paralog with the same mapping location as NOTCH2NLB, but a coding sequence that is identical to NOTCH2NLA (where T197I is the single protein-coding change that differentiates between the NOTCH2NLA and NOTCH2NLB), defines an IGC event between NOTCH2NLA and NOTCH2NLB resulting in two copies of NOTCH2NLA. In past studies the standard initiator AUG was considered NOTCH2NLB-like, while the ATA mutation was considered NOTCH2NLA-like. However, in our study we find that neither type is restricted to a specific paralog. Overall, in this study, 33% (23/69) of haplotypes (H4, H5, H6, H9) appear to have a NOTCH2NLB to NOTCH2NLA conversion event (Figure 5A). This IGC event is shown by our workflow where both the phylogeny and duplication barcode are disrupted between NOTCH2NLA and NOTCH2NLB (Figure 4B) and has been confirmed by MSA of predicted protein sequences from our samples."
Edited:
"Ten percent of haplotypes (H3 and H6) harbor a copy of the gene at this locus that resembles a truncated version of NOTCH2 rather than NOTCH2NLR; this includes two H6 haplotypes that exhibit both gene conversion events (making the combined amount of IGC across haplotypes 42% instead of 43%)."
3. How do the specific tissues or cell types used for RNA-seq and Fiber-seq data collection affect the results? Would additional tissues alter the findings? Are the current findings consistent with other databases (e.g., GTEx) regarding tissue specificity of expression among different paralogs? Additionally, how does the expression of incomplete copies compare to genome-wide averages?
10
To be clear, we did not perform an exhaustive survey of expression across different tissues, and we would expect that the regulatory landscape would differ. We wanted to focus on early brain development which is when and where NOTCH2NL genes are thought to have an effect. As a result, we generated data from dorsal forebrain organoids using a human-induced pluripotent stem cell (hiPSC) line we derived from one of the HPRC lymphoblastoid cell lines. These organoids generate cerebral cortex cell types and are, as such, often used to model the developing brain. This is the preferred cellular context for studying genes like NOTCH2NL, which are expressed early in human brain development. We added additional text to clarify the role of brain organoids:
Original:
"Specifically, lymphoblast cell lines from HG02630 were reprogrammed into iPSCs, which were then differentiated into cortical neurospheres (Figure 6A, Methods)."
Edited:
"Specifically, lymphoblast cell lines from HG02630 were reprogrammed into iPSCs, which were then differentiated towards dorsal forebrain tissue. Such organoids of the cerebral cortex are used to model early fetal brain development, when NOTCH2NL is most highly expressed (Fiddes et al. 2018) (Figure 6A, Methods)."
Resources such as GTEx are especially challenging to compare. The most identical segmental duplications (such as NOTCH2NL) are excluded or collapsed to a single locus such as NOTCH2NLA due

to the inability to distinguish transcripts from single paralogs. The use of this organoid model and longread data let us overcome these limitations of the GTEx catalog, as we had a paired near telomere-totelomere (T2T) genome assembly from the same individual, thereby permitting us to uniquely map the long-read chromatin (i.e., Fiber-seq) and transcript (i.e., Kinnex) data to the NOTCH2NL loci. We anticipate that applying these methods to other tissues with a paired assembly across the NOTCH2NL locus would permit us to identify NOTCH2NL paralogs that have tissue-specific expression. In the absence of paired assemblies, we can analyze large multi-tissue Iso-Seq datasets (Dishuck et al. 2025) for evidence of NOTCH2NL transcription in areas other than the brain. Looking at blood, iPSC, and soft tissue datasets, we see little evidence of full-length NOTCH2NL transcripts across paralogs, and a larger proportion of alternative and fusion transcripts in general (Review Figure 4, NOTCH2NLA). Blood seems to have more full-length transcripts than other tissue types, but fewer compared to brain.

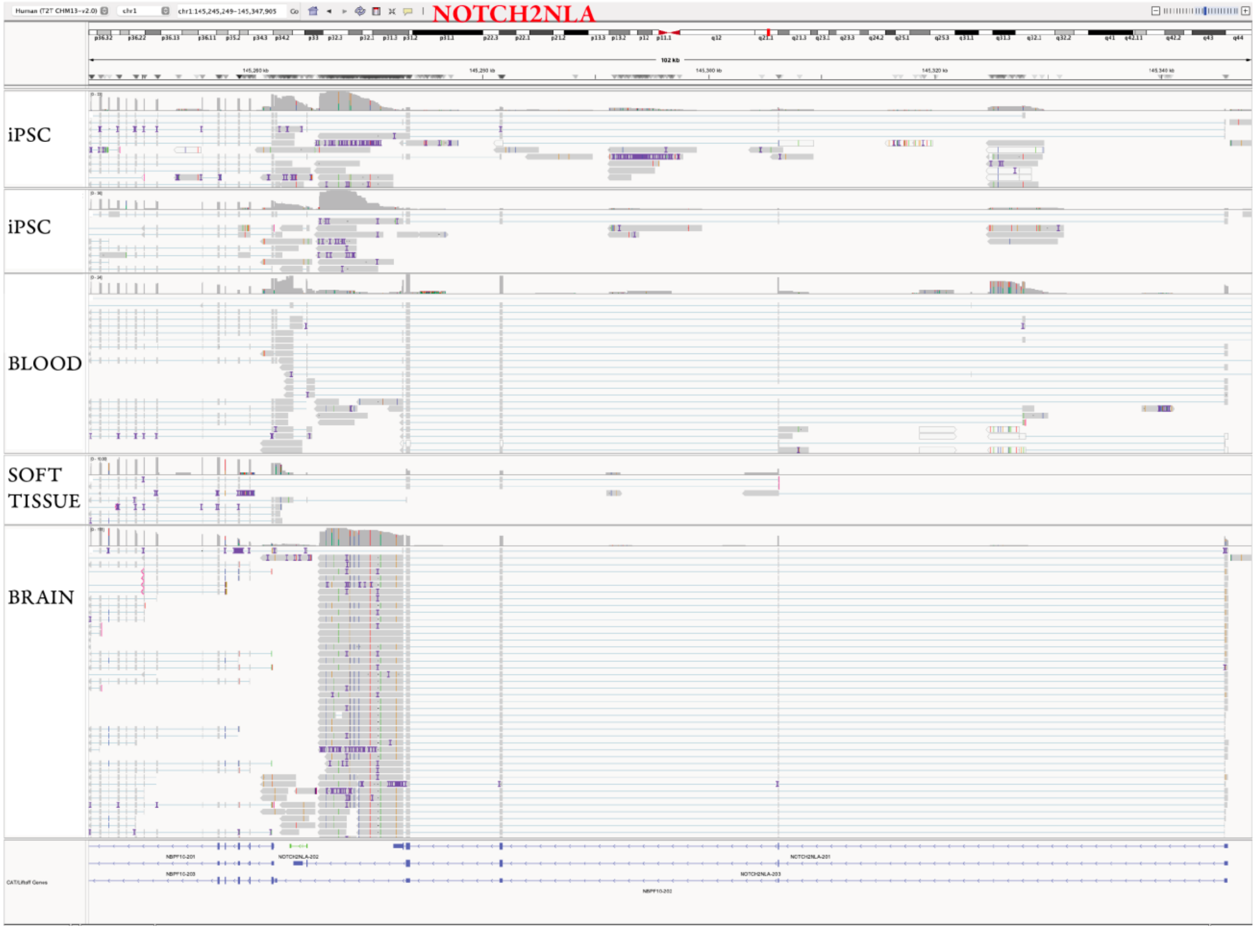

Review Figure 4. IGV view of Iso-Seq data for iPSC, blood, brain, and soft tissue at the NOTCH2NLA locus, where expectedly brain has the most full-length NOTCH2NLA transcripts.

4. Clarification is needed on how ancestral sequences for primate haplotypes were constructed. Are duplication ages calculated accurately using orangutan divergence for normalization, and what potential issues might arise (e.g., is the mutation rate for single nucleotide variants expected to be uniform across the genome)? What are the error margins associated with these estimates? I am particularly interested in the estimated coding sequence alignments—are there signatures of selection (negative or positive, dN/dS)? Is there an accumulation of loss-of-function or nonsynonymous mutations in the proposed pseudogenized copies? Are there regions showing greater conservation than expected by chance or drift?

The ancestral sequences for primate haplotypes used in the phylogeny from Figure 3A were constructed with the tool LSD2, which based age estimation from IQtree using an orangutan–human divergence time of 15.2 MYA. We note this in the NOTCH2NL NHA phylogeny and duplication timing section. Confidence intervals for each timing estimate are also included in Figure 3A. In general, the largest

margins of error with phylogenetic trees such as this are the timing of the speciation events themselves; to address this and strengthen our reconstructions, we included additional speciation events for human–chimpanzee (6.4 MYA) and human–gorilla (8.6 MYA). Adding these new constraints altered the exact timing predictions slightly but overall reinforced our claims that the human lineage of NOTCH2NL began duplicating approximately 4-5 MYA after humans and chimpanzees diverged and further diversified beginning around 3 MYA. Additionally, as the reviewer alludes, we do not expect a constant mutation rate

across the genome and in each lineage, which we now acknowledge. Finally, we find no evidence of positive or negative selection after performing dN/dS analyses (two different approaches). While we find some evidence of skews in genetic diversity (both high and low), we hesitate to make claims of positive or balancing selection based on rampant IGC, which complicates such analyses (see below). We updated Figure 3 with the retimed tree as well as updating the results and methods as follows:

Edited:

"Using orangutan as an outgroup, and known human–orangutan (15.2 MYA), human–gorilla (8.6 MYA), and human–chimpanzee (6.4 MYA) divergence timepoints, we estimate the timing of the duplications or IGC events (Methods) in the human lineage (Figure 3A). Our analysis predicts that the human lineage of NOTCH2NL copies emerged early in human evolution, around 4.9 MYA (4.3-5.7 MYA), after African ape speciation, and that such duplications were also occurring among the other ape lineages (albeit independently or subsequently derived from a larger initiating ancestral ape duplication). Approximately 3.0 MYA (2.2-3.7 MYA), the human-specific copies begin to diverge, distinguishing NOTCH2NLC from NOTCH2NLA/B. NOTCH2NLA and NOTCH2NLB appear to have diverged around 1.7 MYA (1.2-2.3 MYA), although once again IGC may have homogenized these loci since there is ample evidence of ongoing gene conversion (see Patterns of NOTCH2NL human genetic variation) in presentday humans for these two copies, which map in closest proximity to one another."

Edited:

"Notwithstanding this proclivity to duplicate in the common ancestor of great apes, the apparently functional human copies of NOTCH2NL arose much later in human evolution. We estimate that the human-specific expansions (or IGC events) occurred around 4.9 MYA and diversified over a range of 3.0-1.7 MYA (Figure 3A)."

Edited:

"IQTREE estimated phylogenetic dating using LSD2 to build a time tree based on orangutan–human divergence time being 15.2 MYA, gorilla-human divergence time being 8.6 MYA, and chimpanzeehuman divergence being 6.4 MYA."

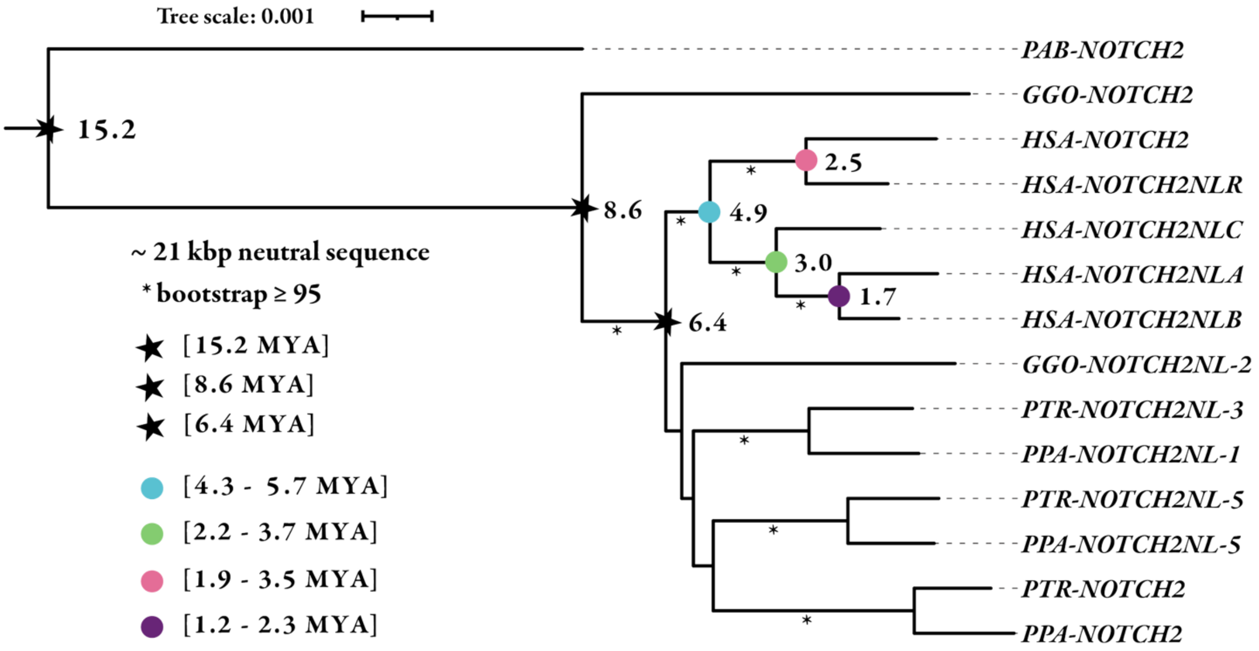

Updated Figure 3A.

Next, we investigated signatures of selection at two different timescales, using branch-site tests of PAML: 1) general selection signature across three lineages (human, Pan, and gorilla), and 2) for each of the human NOTCH2NL paralogs one at a time. For approach #1 (Review Figure 4), we found no significant evidence of selection (p>0.05) 1) in any of the lineages examined (human, Pan and gorilla).

Approach 1 – Between-species signature

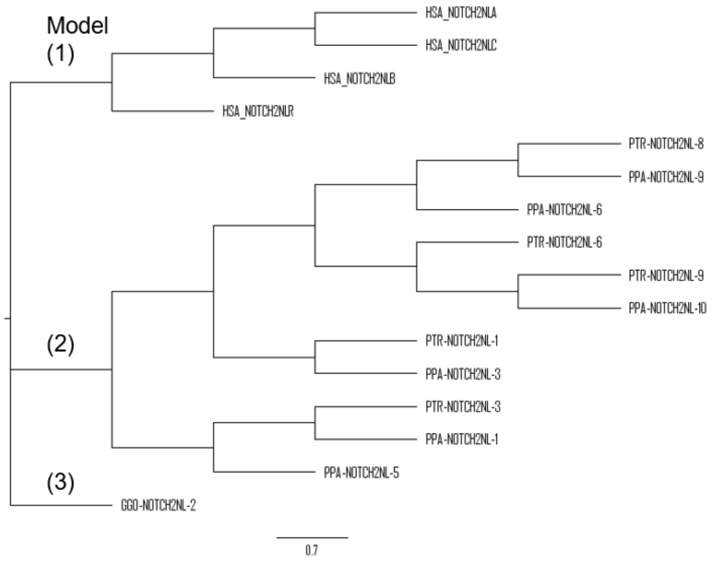

Test	(1) Human	(2) *Pan* lineage	(3) Gorilla
tstv	2.10727	2.10728	2.1066
dnds	1	6.53097	4.1021
h0lnL	-1468.46	-1468.46	-1468.45
h1lnL	-1468.46	-1468.45	-1468.44
p-value (chisqare test)	1.0	0.90	0.86

Review Figure 5. dN/dS approach 1.

Investigating the dN/dS for each of the human paralog copies individually (Review Figure 5), we do find evidence of dN/dS of greater than 1, specifically for the human NOTCH2NLR pseudogene, consistent

14

with relaxed selection. However, it is important to note that none of the tests show significantly greater likelihood over the alternative hypothesis (h1lnL compared to h0), dN/dS>1 model (likelihood ratio test pvalue > 0.05). Thus, we find no evidence for positive selection using dN/dS tests.

Approach 2 – Paralog copy

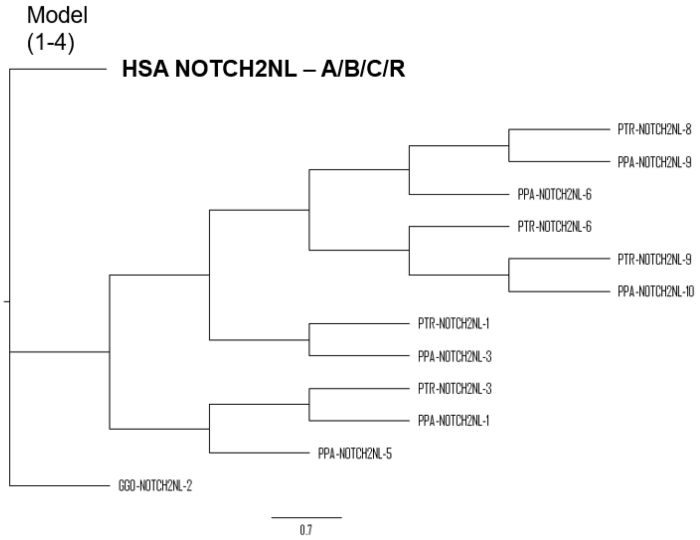

Human copy	A	B	C	R
tstv	1.66427	2.32912	1.88078	2.1756
dnds	1	1	1	1.75825
h0lnL	-1336.3	-1330.68	-1326.88	-1353.84
h1lnL	-1336.3	-1330.68	-1326.88	-1353.77
P-value (likelihood ratio test)	1.0	1.0	1.0	0.71

Review Figure 6. dN/dS approach 2.
Performing a site-model test of PAML to check for conservation or accumulation of nonsynonymous mutation, we found that the likelihood ratio test between models of nearly neutral (M1a or M7) compared to positive selection (M2a or M8) indicate greater likelihood of model for positive selection, with p<0.05 (0.044 for two tests, M1a vs. M2a and M7 vs. M8) (Review Table 1). This suggests accumulation of nonsynonymous mutation in subset of sequences of all NOTCH2NL copies being compared, although the branch level tests were insignificant.

Review Table 1. PAML site-model test of selection

Model comparison	lnL(null)	lnL(alt)	LRT	dF	p-value
M1a (nearly neutral) vs M2a (positive selection)	-1383.98362	-1380.855648	6.255944	2	0.043807
M7 (beta) vs M8 (beta positive)	-1384.00585	-1380.879686	6.25232	2	0.043886

5. The argument regarding trade-offs and selective forces presented in the conclusion is interesting. However, is there quantitative or model-based evidence supporting the assertion that genomic instability from gene duplications generates a significant fitness cost? If such studies exist, they should be cited.
In a population study of copy number variants (CNVs) examining the allele frequency distribution, Itsara et al. 2010 found that large CNV events (both deletions and duplications) are typically under strong purifying selection in the genome and that distortion of allele frequency increases with the size of the duplication event suggesting a fitness consequence. This observation was supported by survey of duplications in Drosophila (Langley et al. 2012). A follow-up study in humans focused on smaller CNVs from the 1KG using WGS read depth (Sudmant et al. 2015) attempted to refine this model and showed a strong effect for deletions as a function of size but less so for duplications. Empirical data from Salmonella, however, suggests that duplications of the genome can reduce fitness by as much as 3-16%; but this effect appears to be context dependent (Reams et al. 2010). In humans the primary observations
15
regarding negative fitness stem from the more than 35 genomic disorders mediated by NAHR between existing segmental duplications where children are born with neurodevelopmental delay and seldomly reproduce (Lupski and Stankiewicz 2005, Cooper et al. 2011, Coe et al. 2019). For one of these loci (chromosome 16p11.2 segmental duplication fixation and recurrent deletion associated with autism), the relationship was modeled by simulation (Nuttle et al. 2016). That study shows that for BOLA2B segmental duplication (which mediates one of the most common autism genomic disorder regions) to reach the current allele frequency seen in humans, moderate positive selection (s = 0.0015) must have acted/be acting on the segmental duplication. While we find no signals of positive selection using traditional analysis like dN/dS in our data, most selection tests are ill-suited to analyze recently duplicated gene families such as NOTCH2NL. Similar to BOLA2B, the expansion of NOTCH2NL to 3-4 copies was

associated with Mbp of additional duplicated DNA, which now contributes to one of the most common genomic disorders in the human population (1q21 deletion/duplication syndrome) suggesting that the tradeoff conferred by NOTCH2NL offsets this mutational liability. We added additional description to the discussion to cite these studies:

Edited:

"The fact that this region is among the most frequently rearranged regions of the human genome (Cooper et al. 2011) is a testament to the evolutionary instability that continues to persist in the human population. It is well established that the presence of these human-specific duplications has phenotypic consequences associated with recurrent rearrangement and developmental delay (Mefford et al. 2008, Brunetti et al. 2008). Consistent with the core duplicon hypothesis (Marques-Bonet and Eichler, 2009) and population studies that suggest large duplications should be under purifying selection unless some other type of selective force is acting (Itsara et al. 2010, Reams et al. 2010, Langley et al. 2012, and Nuttle et al. 2016), the mutational lability of chromosome 1q21.1 and the emergence of novel NOTCH2NL genes likely represents a significant trade-off between positive and negative selection during human evolution."

6. The term "functional copies" should be clearly defined. Does this refer specifically to protein-coding copies? If so, "protein-coding copies" would be a more accurate term. Indeed, the authors' own previous work has shown that long non-coding RNAs derived from incomplete duplications can have important functional roles and evolutionary impacts.

Functional copies in this context do indeed refer specifically to evidence of protein-coding genes. We edited several instances as follows for clarity:

Edited:

"Using nearly complete long-read assemblies generated from 70 human and 12 ape haploid genomes, we show independent recurrent duplication among apes with protein-coding copies emerging in humans ~2.1 million years ago."

Edited:

"Using comparative data from other great apes, we construct an evolutionary model for its origin and spread over the last 10 million years, providing evidence of recurrent duplication in different apes while showing that protein-coding copies began to emerge and spread ~2 million years ago (MYA) in the human lineage."

Edited:

"In summary, we hypothesize that the dramatic restructuring of the NOTCH2NL loci during human evolution led to the only ape lineage with protein-coding copies."

16

7. The term "first unique genes" in the figure legend of Figure 1 is unclear, representing another example of the vague expressions present throughout the manuscript.

The first unique genes outside the SD block refer to the first non-duplicated genes found outside the SD block. To clarify, we edited the Figure 1 legend as follows:

Edited:

"A subset of genes is depicted, including NOTCH2NL (purple), NBPF genes that are directly downstream of NOTCH2NL (pink), non-duplicated genes (gray) immediately adjacent to the SD blocks, and others (black)."

Reviewer #2: The recurrent incomplete duplications of the NOTCH2/NOTCH2NL genes are an illuminating example of a human-specific evolutionary change that contributed to the adaptation of a hallmark human feature - our large brain. By integrating long-read sequencing data generated from both non-human apes and individuals of diverse human populations, the authors uncover the timing, order, and structure of these duplications. They further reveal that the copy number of various NOTCH2NL paralogs varies within humans. Through investigation of the regulatory features and expression patterns of these paralogs, the authors attempt to shed light on their functionality and suggest selective constraint.

We'd like to thank Reviewer #2 for taking the time to read our manuscript and provide clear, specific criticism that helped us more clearly organize our manuscript for understanding and highlight its novelties. We have edited the text to accommodate their feedback.

Major points

1. From my perspective, the study of NOTCH2/NOTCH2NL has the potential to deepen our understanding in three key ways: (a) shedding light on the role and function of these genes in human

evolution, (b) serving as a case study to understand the broader mechanisms and impacts of SDs, and (c) introducing novel approaches and tools to advance the study of SDs. While the authors conduct thorough and impressive analyses of these loci, resulting in a high-resolution map of their evolutionary history, I am not convinced that their findings advance any of the three aspects mentioned above in a meaningful way, especially in light of the findings of the Fiddes et al. 2018 paper.

We apologize that we failed to highlight the significance and novelty of this work more clearly. This study is relatively unique because it places these three aspects into the context of human and nonhuman ape variation revealing the challenges of assessing genic constraint, the role of interlocus gene conversion, and methodologies that will have to be developed to more systematically assess these going forward. Thus, it contributes to advancing our understanding in all three areas highlighted by the referee. Having said that, we should stress that this work is primarily a human genetics study into the complex patterns of variation, transcription, and regulation of the gene family. Thus, this study represents the first detailed analysis of human genetic variation at the level of haplotypes. This required the generation and analysis of long-read sequence data and placing this variation into context by beginning to interrogate its effect on regulation of individual NOTCH2NL copies.

More specifically, Fiddes et al. 2018 was a foundational paper for our study of NOTCH2NL; however, it relied on technologies that were severely limited in duplicated regions. Further, the quality of the chimpanzee and gorilla genomes at the time made the assertion that these apes lacked human-like NOTCH2NL genes provisional and based on what we knew was incomplete data. A major finding of this study was the realization that in the Gorilla and Pan lineages there has been extensive duplication of genomic segments containing a fragment of the NOTCH2 gene fused to an NBPF gene. The number and variety of these ape duplicons is much more extensive than what we were able to report in our 2018 paper, likely due to assembly errors and gaps. Consequently, this study highlights a critical random event that led to the emergence of a version of this NOTCH2NL-NBPF duplicon that had the potential to encode a stable protein ONLY in the human lineage—thus shedding new insight into functionalization of NOTCH2NL during human evolution. The nature of this NOTCH2NL-NBPF duplicon makes it unsurprising that additional copies emerged subsequently including one that appears to be a pseudogene (NOTCH2NLR) and the three functional paralogs. Second, the high-quality long-read assembly from multiple human genomes allowed us to correct the major configuration and organization of the NOTCH2NL locus as reported in Fiddes et al. 2018 and propagated for more than a decade on the browser. Beyond this, we were able to refine the timing of each human-specific duplicate and demonstrate unequivocally recurrent duplication in different ape lineages. Leveraging human variation

data, we were able to employ an assembly-based method for detecting gene conversion instead of read depth, thus developing novel haplotype-based methods to advance the study of variation at this locus and more broadly other SD regions. Finally, we explored similarities and differences in the regulatory architecture of the human NOTCH2NL paralogs, which have never before been addressed. Using a Fiber-seq brain organoid model, we were able to create a novel regulatory architecture map of each paralog in early brain development that showed the degree of chromatin accessibility for all regulatory elements in NOTCH2NL regions. These maps suggest ~14% of these regulatory sites are paralogspecific and of interest for studying regulatory differences that impact this gene family. With the same brain organoid model, we uniquely quantified NOTCH2NL paralog-specific expression data, which indicate distinct abundance differences in paralog transcripts. Most striking, we find that NOTCH2NLA, the paralog under the greatest copy number constraint, possesses unique regulatory architectures and the greatest number of full-length transcripts.

We added a paragraph to the discussion commenting on these three aspects and specifically how this paper is an advance over Fiddes et al. 2018:

"While Fiddes et al. 2018 was a foundational paper for our study of NOTCH2NL gene duplications, it relied on technologies that were severely limited in duplicated regions. Further, the quality of the chimpanzee and gorilla genomes at the time made the assertion that these apes lacked human-like NOTCH2NL genes provisional and based on the lack of what we knew was incomplete data. Our study represents the first detailed analysis of human genetic variation at the level of haplotypes obtained from long-read sequence data from apes and >50 human references. We find multiple copies of NOTCH2NL among all African apes and show that the NOTCH2NL genes began duplicating before and after (independently) speciation in different ape lineages. These copy number changes accompanied one of the most dramatic karyotypic differences (the pericentric restructuring of human chromosome 1) distinguishing humans from all other apes. Secondly, while

we demonstrate that transcriptional potency in both humans and other nonhuman apes exists, only in humans did a subsequent frameshift result in a modified carboxy terminus essential to creating a stable/functional protein product. Third, our investigation of human genetic variation shows rampant gene conversion events between human haplotypes, including the creation of the previously reported, but uncharacterized, human gene NOTCH2tv, present in ~10% of humans. The data also allow us to now predict which of the many duplicate genes are under the greatest copy number constraint (NOTCH2NLA). Finally, by applying a novel long-read sequencing-based approach, Fiber-seq, to human brain organoids generated from iPSC, we provide the first detailed comparative open chromatin accessibility map and show that ~14% of these sites are paralog-specific providing a roadmap to exploring transcriptional regulatory differences for the different members of this gene family."

2. More specifically, it remains unclear whether the various NOTCH2NL paralogs and their combinations (haplotypes) truly differ in function and phenotypic impact. The authors propose that NOTCH2NLA may be particularly important, given its fixation in multiple haplotypes. To support this claim—as well as the idea that NOTCH2NLA repeatedly serves as the preferred donor locus—an ancestry-aware analysis is warranted, rather than just counting the number of individuals with each haplotype. I suggest examining whether the losses and biased conversions are recurrent events versus arising from a single origin, which would help clarify whether these patterns truly indicate biased directions or simply reflect one ancestral event.

We thank the reviewer for this important point. Unfortunately, we do not have deep ancestry information on a large number of samples. However, we attempted to address the comment by considering African vs. non-African samples. Interestingly, we find that out of 23 total NOTCH2NLB to NOTCH2NLA gene conversion events, the majority are found in African haplotypes (19/23, 83%) with the remaining four

occurring in admixed Puerto Rican samples from the 1KG. This degree of stratification is statistically significant (p=0.0008, Fisher's exact test) and suggests that the NOTCH2NLB to NOTCH2NLA conversion was ancient property of the human lineage. In terms of whether this event is recurrent or arising from a single origin, we have no strong evidence of either, as it could represent a recurrent event near exclusive to the African population. Overall, we agree with the reviewer that we cannot claim NOTCH2NLA is the preferred donor locus; however, NOTCH2NLA still remains the only human-specific paralog seen in all samples. We updated Figure 5A and the following text accordingly:

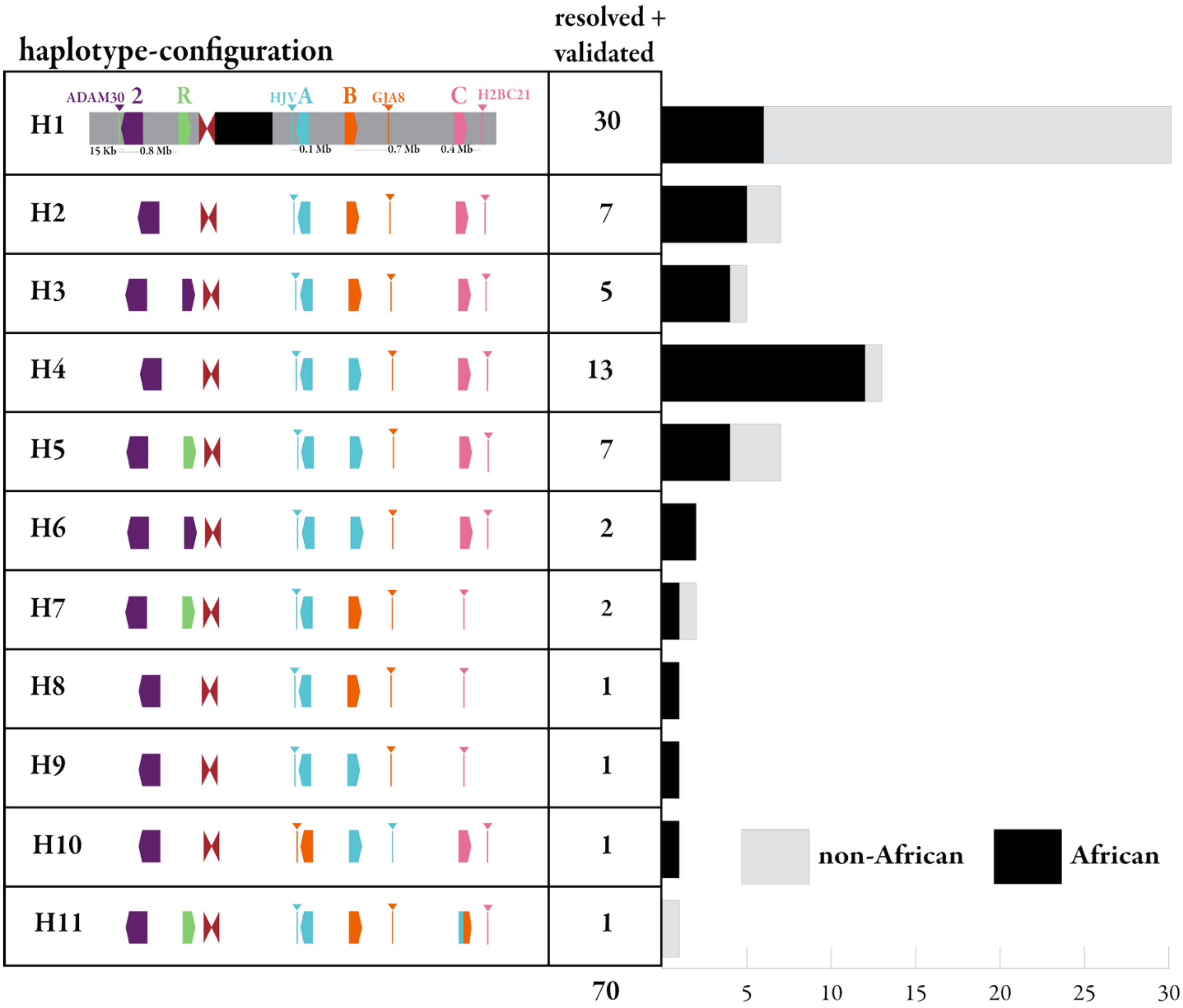

Updated Figure 5A.
Original:
"The analysis suggests that NOTCH2NLA is the preferred donor locus as we never observe NOTCH2NLA converted to a NOTCH2NLB identity."
Edited:
"In our analysis we never observe NOTCH2NLA converted to NOTCH2NLB identity. However, this event is almost exclusive to the African superpopulation (p=0.0008, Fisher's exact test) *suggesting it is an ancient event. It is currently unclear whether this conversion event is recurrent or the product of a single*
20
ancestral event, but we predict it was first seen around 2.8 MYA. This is consistent with the timing of divergence of NOTCH2NLC from NOTCH2NLA/B."
Edited:
"The biased gene conversion that is potentially driving the fixation of NOTCH2NLA as well as the high level of fourth intron retention transcripts of NOTCH2tv and NOTCH2NLR may be examples of ongoing evolution in this gene family, such as specification of the functional NOTCH2NL protein and potential acquisition of a novel carboxy terminus."
**Note: this statement was also edited for comment below.*

3. The authors put a special emphasis on mapping the regulatory differences of these loci. While this is an important direction, the analyses fall short of providing meaningful insight into regulatory evolution and differences.

Given that these are large duplications that on one hand included the potential regulatory elements of these genes, but on the other hand, now exist in a new genomic context, one could expect both similarities and differences from the original chromatin pattern. The authors describe both but do not quantify whether these similarities and differences are more or less than expected by chance. Moreover, given the observation of transcription from these paralogs, the accessibility of their promoters is again expected. Thus, it is unclear what these comparisons teach us.

We appreciate this point brought up by the reviewer and sought to quantify whether the similarities and differences in actuation we see in NOTCH2NL paralogs are significant or just what is expected by chance. To address this, we used the HG02630 organoid Fiber-seq data to build a background model of what we would expect for differences in chromatin accessibility to be between haplotypes within a cell (under the assumption that if the different NOTCH2NL paralogs are simply different by chance, these differences would be on the same order of magnitude as differences seen between the same element on opposite haplotypes). Using this approach, we quantified the haplotype differences in actuation of all FIRE peaks in non-duplicated regions on chromosome 1 (which we defined as the expected amount of variability one would see when you quantify an element that is present along two separate DNA fibers within the nucleus). We then compared this distribution to what we observed by comparing the chromatin accessibility at NOTCH2NL FIRE peaks between different paralogs. This revealed that NOTCH2NL paralogs do indeed show a larger difference in chromatin actuation than would be expected, consistent with either positional effects or paralog-specific genetic variants modulating the chromatin actuation of the different NOTCH2NL paralogs. We added a new Figure S9 and added new text to address this point (as well as the next reviewer comment):

"To further refine the scope of regulatory elements more directly associated with each NOTCH2NL paralog, or at least their respective promoters, we applied FiberFold (Dubocanin et al. 2025) to our brain organoid data to predict topologically associated domains (TADs) in NOTCH2NL paralog regions in a haplotype-aware manner. FiberFold predicts each NOTCH2NL paralog occupies a distinct 3D genomic environment despite their highly identical underlying sequence and, as expected, each NOTCH2NL TSS specifically is surrounded by unique 3D landscapes (Figure S9A). These specific paralog patterns are largely consistent across haplotypes, and we find differences between paralogs are more pronounced than differences between haplotypes (Figure S9B). This point is further supported by our independent analysis to quantify haplotype differences in actuation against pairwise differences between NOTCH2NL paralogs (Figure S10). Next, by overlaying Fiber-seq data on predicted 3D contact plots for NOTCH2, NOTCH2NLA, and NOTCH2NLB, we are able to observe which elements fall within TAD boundaries associated with the TSS (Figure S9C). Specifically, we find that a

21

NOTCH2NLA/B specific cluster of elements, which are found in a region syntenic to NOTCH2 as well, are within the predicted TAD boundaries associated with NOTCH2NL transcription."

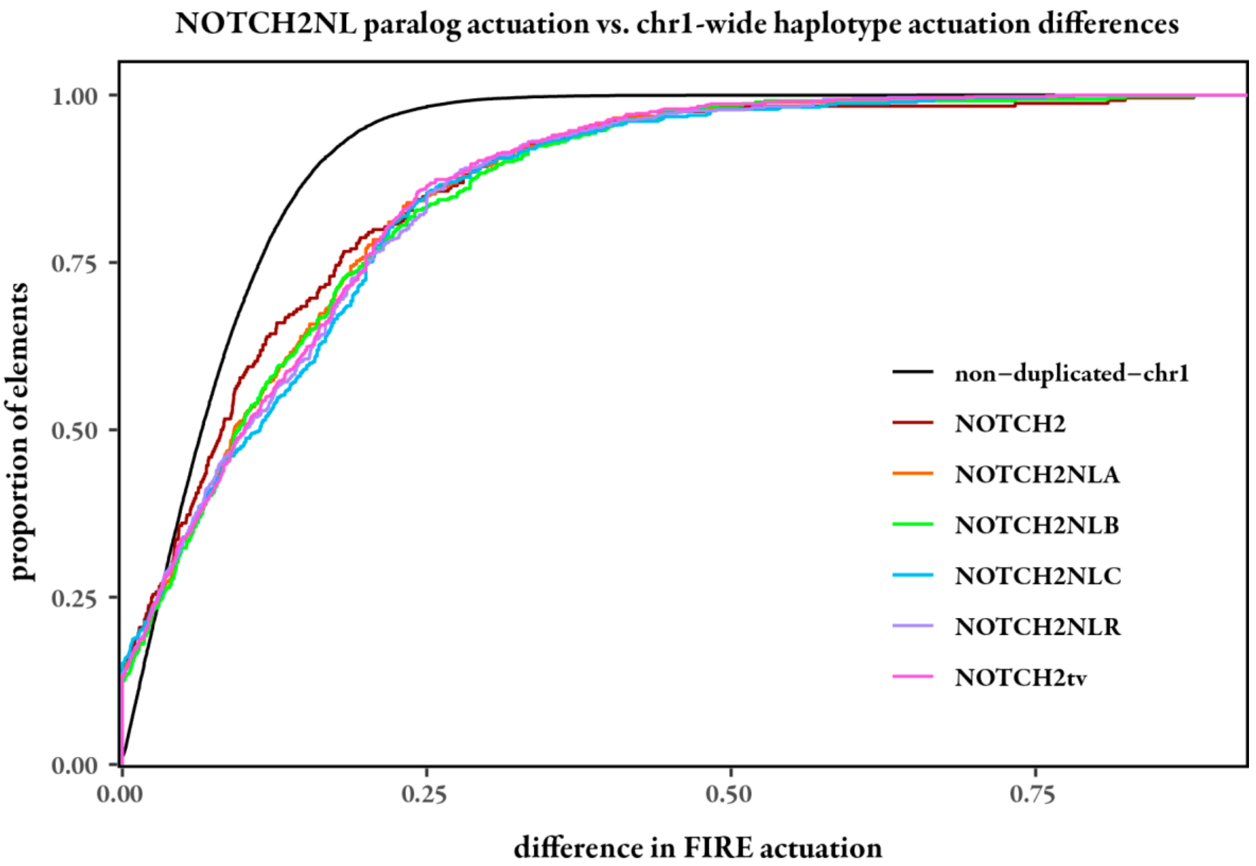

Figure S10. Comparison of NOTCH2NL paralog and haplotype actuation. The model of expected haplotype differences on chromosome 1 using brain organoid data mapped to T2T-CHM13 (black) shows a higher proportion of regulatory elements with less actuation difference than what is seen for the different NOTCH2NL paralogs.

4. The authors put a special emphasis on the number of peaks around these paralogs (503-634). Most studies estimate fewer than 10 regulatory elements per gene. Thus, the vast majority of these peaks (>98%) are expected to be non-functional and the focus on the number of peaks has little meaning. This is further exacerbated by the fact that only two samples are used for this, and thus, the differences observed could simply reflect transient dynamics, power differences or noise. To identify the likely regulatory elements of these genes, and to gain insight into the evolutionary differences that are of potential importance, more in-depth analyses of these loci are called for. I acknowledge the fact that given the nature of these loci, reliable chromatin maps are hard to find/generate, and I appreciate the use of Fiber-seq. However, without further regulatory data, such as histone modification marks, TF binding, reporter assays, 3D contact maps etc, there is not much one can conclude from the data.

We appreciate the reviewer pointing out this limitation in the regulation section of the manuscript. The intent of the statement below was to characterize the general regulatory landscape surrounding each paralog, including the total number of accessible sequences called as regulatory elements in our regions of interest; however, we see how this can mislead the reader to assign more importance to it. We removed the portion about the number of elements to clarify:

Edited:

22

"Overall, this revealed that each NOTCH2NL paralog shares a promoter with accessible chromatin within CHM13 cells, and that each has between 503 and 634 accessible chromatin elements within 300 kbp of their TSS."

As the reviewer alludes to, our fundamental understanding of gene and genome regulation relies on the interplay between DNA accessibility, DNA-binding proteins, and their coordination in three-dimensional (3D) space; thus, while our current data provides a map of the accessible elements within the vicinity of the NOTCH2NL genes, further data is needed to disentangle which ones may actually be regulating

NOTCH2NL paralogs. The gold-standard approach for accomplishing this is through genome-editing approaches. However, due to the high sequence similarity between the five paralogs, it would be quite challenging to uniquely edit only one of the paralogs. 3D chromatin maps have frequently been used to infer regulatory elements that may be pertinent for a gene but are often limited based on the resolution of these 3D approaches, and their reliance on short-read sequencing, which cannot uniquely map reads to the different NOTCH2NL paralogs. In the absence of Hi-C data, we leveraged a recent deep learningbased approach called FiberFold (Dubocanin et al. 2025), which achieves near-experimental accuracy in predicting high-resolution (10 kbp) 3D chromatin structures directly from Fiber-seq data in a haplotypeaware manner.

Using HG02630 brain organoids, we applied FiberFold across all paralogs on both haplotypes and found that each paralog occupies a distinct predicted 3D genomic environment within its respective contig, despite their highly similar underlying sequences. Moreover, when examining 786 kbp windows centered on transcription start sites (TSS), we observed unique 3D landscapes around each TSS, despite high synteny in the surrounding sequences (Figure S9A). These patterns remain consistent across haplotypes, with differences between paralogs being more pronounced than differences between haplotypes (Figure S9B). By overlaying our Fiber-seq data with these predicted 3D contact plots, we can also identify which of the regulatory elements surrounding each NOTCH2NL paralog are predicted to be contained within the same topologically associating domain as the NOTCH2NL paralog promoter (Figure S9C). As noted above, although this does not prove that these regulatory elements are in fact regulating NOTCH2NL, it does provide further evidence as to the biological impact of each element. We edited both the results and discussion and added Figure S9 to the manuscript to include this analysis:

"To further refine the scope of regulatory elements more directly associated with each NOTCH2NL paralog, or at least their respective promoters, we applied FiberFold (Dubocanin et al. 2025) to our brain organoid data to predict topologically associated domains (TADs) in NOTCH2NL paralog regions in a haplotype-aware manner. FiberFold predicts each NOTCH2NL paralog occupies a distinct 3D genomic environment despite their highly identical underlying sequence and, as expected, each NOTCH2NL TSS specifically is surrounded by unique 3D landscapes (Figure S9A). These specific paralog patterns are largely consistent across haplotypes, and we find differences between paralogs are more pronounced than differences between haplotypes (Figure S9B). This point is further supported by our independent analysis to quantify haplotype differences in actuation against pairwise differences between NOTCH2NL paralogs (Figure S10). Next, by overlaying Fiber-seq data on predicted 3D contact plots for NOTCH2, NOTCH2NLA, and NOTCH2NLB, we are able to observe which elements fall within TAD boundaries associated with the TSS (Figure S9C). Specifically, we find that a NOTCH2NLA/B specific cluster of elements, which are found in a region syntenic to NOTCH2 as well, are within the predicted TAD boundaries associated with NOTCH2NL transcription."

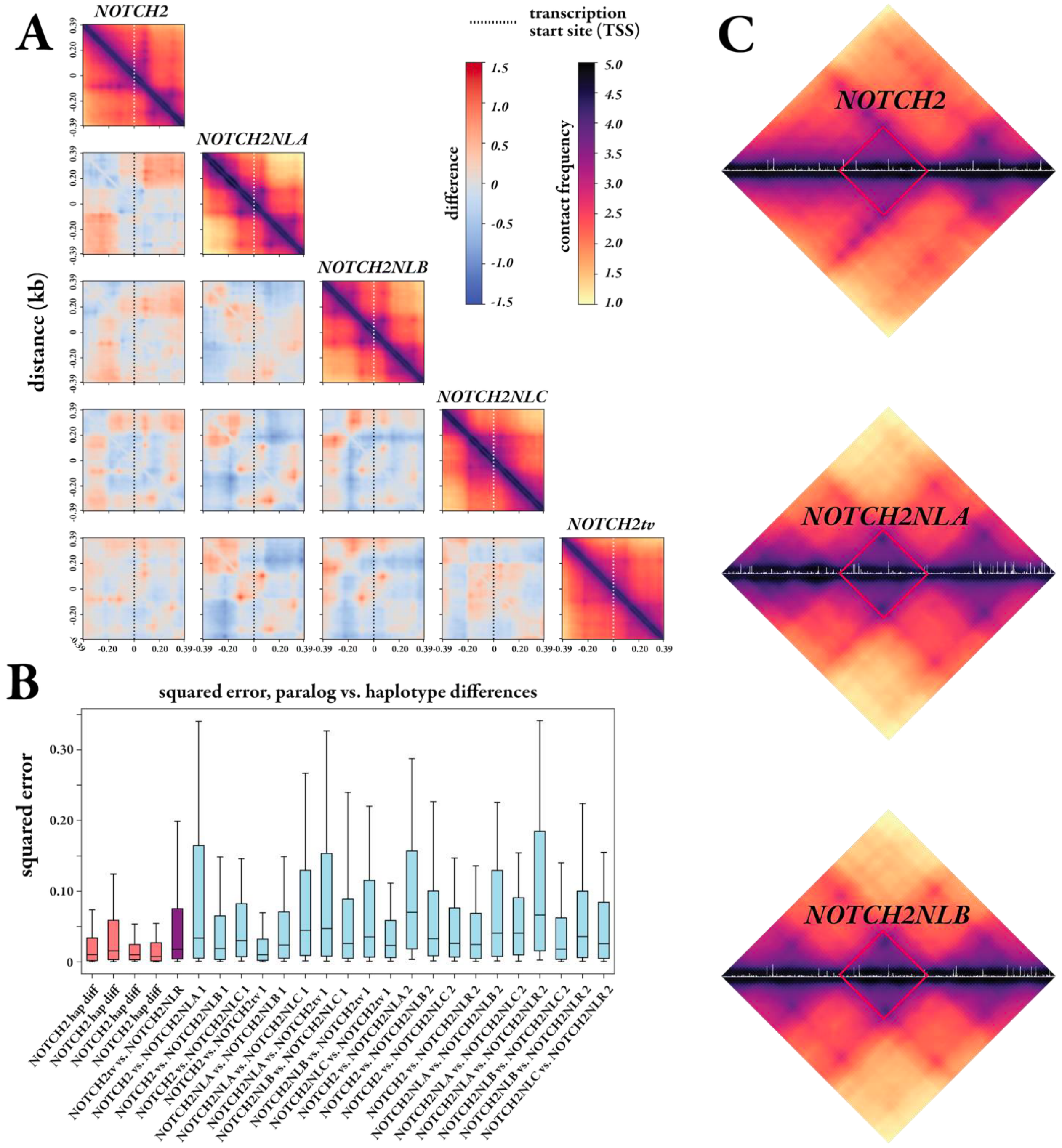

Figure S9. Predicted TADs and associated regulatory architectures in NOTCH2NL paralog regions. a) FiberFold TAD NOTCH2NL paralog predictions and pairwise heatmap of contact similarities and differences. Dotted lines represent gene TSS. b) Squared error chromatin accessibility differences between haplotypes and pairwise paralog comparisons. c) Fiber-seq chromatin actuation overlaid on TAD predictions for NOTCH2, NOTCH2NLA, and NOTCH2NLB to show which regulatory peaks are included in contact regions.

24

5. I acknowledge that the findings of the authors are complex and challenging to visualize. However, the figures are currently unclear and lacking important information. The first sections of the text describe a

very long list of genetic changes between the lineages. While these findings are important, they are very hard to follow and could be described much more succinctly and largely replaced with clearer figures. More specifically: (a) The paper would especially benefit from a timecourse fig depicting the full story of duplications and conversions. The current figs show some of these aspects, but a summary fig is missing, and (b) Many figs are hard to follow, packed, and visually messy.

To more clearly present the full story of duplications and conversions, we created a summary time-course figure. This describes the initial inversion and duplication events that positioned the current organization in several great ape lineages, including human. We also summarize how this unique organization predisposes the region to gene conversion and structural variation caused by unequal crossing over, which is the responsible for the wide range of genetic variation we report. We replaced the original Figure 2 with this figure, moving the previous one to the Supplementary Figures:

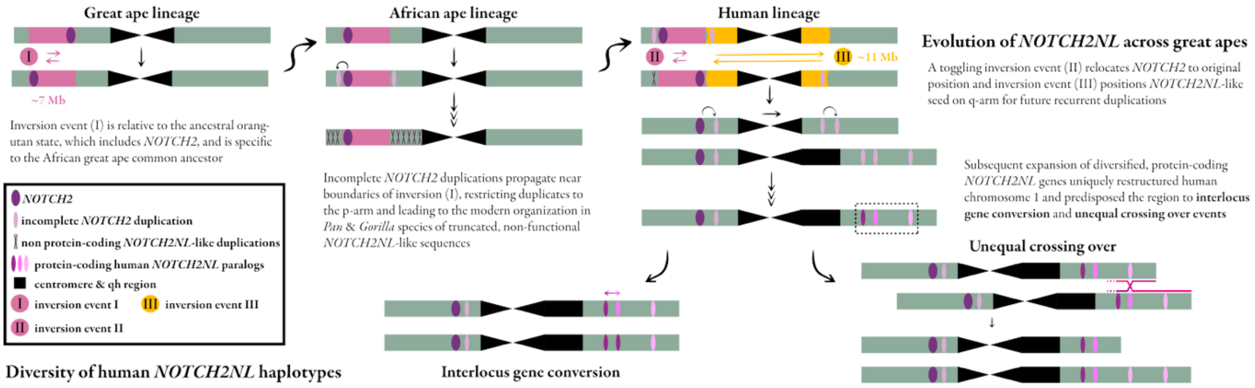

Updated Figure 2. Summary of chromosome 1 rearrangement and NOTCH2NL evolution. NOTCH2NLlike genes have duplicated independently in three separate ape lineages, with protein-coding ability exclusively in the human lineage. Three inversion events, beginning in the African ape common ancestor, in addition to the subsequent duplication events, helped position NOTCH2NL to the current human genomic configuration. The organization and high homology maintained between human NOTCH2NL paralogs and surrounding regions promotes structural variation associated with gene conversion and unequal crossing over.

Minor points

6. The introduction is very clear and covers the key points. The only thing missing there, in my opinion, is a clearer presentation of the motivation and goal of the study. The authors describe the importance of NOTCH2 and NOTCH2NL in evolution and disease, and the challenges in studying SDs, but I think readers would benefit from a sentence summarizing precisely how solving these challenges would advance our understanding of human evolution and disease.

To more clearly present the motivation and goal, we added additional text to the end of the introduction:
Edited:
"Finally, we apply Fiber-seq and long-read transcript sequencing to a dorsal forebrain organoid model, providing an initial assessment of potential regulatory regions that are shared and those that distinguish paralogs specifically during human neurodevelopment. Characterizing the NOTCH2NL gene family will add to our limited understanding of how highly identical gene duplications have shaped human evolution by promoting the formation of novel genes unique to humans. Our goal was to annotate

25

structural differences, expression and regulatory changes in the context of human genetic variation—a feat only possible with completely resolved haplotype sequence. Understanding variation in patterns of the human haplotypes will have the added benefit of helping to define breakpoints associated with patients harboring chromosome 1q21 deletions and duplications in the future (Mefford et al. 2008, Brunetti et al. 2008)."

7. "NOTCH2NLC appears the most structurally divergent; most paralogs share the bulk of synteny upstream of the gene model, yet the opposite is true for NOTCH2NLC, which has the most homologous alignments with NOTCH2NLR." - What is the point the authors are trying to convey here? That C is most homologous to R or that its synteny is mainly downstream as

opposed to upstream? In addition, according to Table 1, the lowest pairwise homology is actually with NOTCH2NLR. Figure 3 also shows that C is closer to A and B than it is to R or NOTCH2.

To make this clearer, we revised the sentence as follows:

Edited:

"NOTCH2NLC appears the most structurally divergent; most paralogous regions share the bulk of synteny upstream of the gene model, yet the opposite is true for NOTCH2NLC. Overall, NOTCH2NLC shares the largest number of homologous alignments with NOTCH2NLR."

8. "Though the difference in average SD lengths between NHA and human (311 kbp) is not significant (p=0.21; t-test one-sided)..." - Are they checking the overall difference in size or specifically if NHA SDs are shorter? If it's the former, it should be a two-sided test, if it's latter, it should be one-sided and the text should be changed accordingly. In any event, it is not clear what the authors aim to achieve by conducting this test. Is it to determine whether there is a common source for these SDs?

The test was performed to check if the extensive synteny observed in humans is statistically significant compared to that of nonhuman apes. We edited the sentence as follows:

Edited:

"Though the difference in average SD lengths between NHA and human (311 kbp) is not significant (p=0.42; t-test two-sided)..."

9. "Subsequently, in the human lineage, this region reverted to its ancestral configuration (inversion II) but expanded in size. We estimate that this region is 17 Mbp larger than the syntenic region in chimpanzee and orangutan." - Do the authors mean to refer to inversion III here? Because according to figure 2, it does not seem as if inversion II has an addition of this size.

Yes, we thank the reviewer for catching this miswording issue. The region's size is meant to refer to the combined region that is represented by both inversions II and III. The section has been edited as follows:

Original:

"The first inversion (I) occurred in the ancestor of African great apes, flipping the region into its current orientation in gorilla, chimpanzee, and bonobo. Subsequently, in the human lineage, this region reverted to its ancestral configuration (inversion II) but expanded in size. We estimate that this region is 17 Mbp larger than the syntenic region in chimpanzee and orangutan. Additionally, an expansion of SDs in humans coincided with a human-specific pericentric inversion (III), corresponding to the rearrangement originally described by Yunis and Prakash (Yunis and Prakash 1982) and subsequently refined to 154

26

kbp and 562 kbp breakpoint intervals at chromosome 1p11.2 and 1q21.3, respectively (Szamalek et al. 2016)."

Edited:

"The first inversion (I) occurred in the ancestor of African great apes, flipping the region into its current orientation in gorilla, chimpanzee, and bonobo. Subsequently, in the human lineage, this region reverted to its ancestral configuration (inversion II). Additionally, an expansion of SDs in humans coincided with a human-specific pericentric inversion (III), corresponding to the rearrangement originally described by Yunis and Prakash (Yunis and Prakash 1982) and subsequently refined to 154 kbp and 562 kbp breakpoint intervals at chromosome 1p11.2 and 1q21.3, respectively (Szamalek et al. 2016). We estimate that the region encompassing inversions II and III is 17 Mbp larger than the syntenic region in chimpanzee and orangutan."

10. "In addition to the inversions, structural variants, including 308-829 insertions and 312-816 deletions (>50 bp), were observed across the NHAs (Supplementary Table 2), significantly enriched at the breakpoints of inversions (p<0.05), in chimpanzee, bonobo, and gorilla (Supplementary Table 2). Assessing the region in terms of duplicated sequences, we also found that the locus is enriched with SDs (p<0.001, in T2T-CHM13 space);" - p-values are stated but not the statistical test/s that were used.

"All species harbor four shared NOTCH2NL homologs..." *- According to figure 2, seems like orangutans do not.*

We thank the reviewer for catching this confusing wording; "all species" should refer to all African ape species. We edited the statement to reflect this:

Edited:

"All African ape species harbor four shared NOTCH2NL homologs, while three additional homologs are specific to gorilla, two are specific to the Pan genus, and three are specific to chimpanzee and bonobo, respectively."

The test performed here refers to the one-sided simulation test in which we shuffled the region to a random location in the genome (excluding centromere), to obtain distribution of null which was compared with the observed intersection with structural variants. The p-value computed here refers to the proportion of the null distribution with more extreme statistics. We edited the statement as follows:
Edited:
"In addition to the inversions, structural variants (SVs), including 308-829 insertions and 312-816 deletions (>50 bp), were observed across the NHAs and significantly enriched at the breakpoints of inversions (one-sided simulation test, p<0.05) in chimpanzee, bonobo, and gorilla (Table S2). Assessing the region in terms of duplicated sequences, we also found that the locus is enriched with SDs (one-sided simulation test, p<0.001, in T2T-CHM13 space);"
We also added the following description of the test to the methods:
"Simulation test for enrichment of SVs and SDs at inversion breakpoints
Coordinates of inversions and SVs identified by a previous study were utilized (Yoo et al. 2025), which used Syri (v1.6.3) (Goel et al. 2019) and PAV (v2.3.2) (Ebert et al. 2021) pipelines. We first quantified SVs/SDs at the inversion breakpoints found within 110-160 Mbp regions by intersecting 100 kbp of inversion coordinates with SVs/SDs to get observed bp of SVs/SDs at the inversion breakpoints. To test enrichment of variants at the inversion breakpoints, we generated random distribution of SVs/SDs by
27
shuffling the breakpoints, while restricting the region to 110-160 Mbp, and excluding centromere satellites. The null statistics distribution was generated by quantifying the SVs/SDs at the randomly shuffled regions. The one-sided simulation p-value was computed by quantifying the proportion of null distribution that show more extreme statistics (greater than observed SVs/SDs)."

11. "In contrast, all the NHA NBPF genes have a high copy number of the CON1, CON2 and CON3 Olduvai domains." - What is considered high? Many have 3, which doesn't seem like a lot in comparison to others and in comparison to HLS copy number in humans.

This statement was misworded. We thank the reviewer for flagging this. We edited as follows, also replacing Olduvai with DUF1220:
Edited:
"In addition, some of the NHA NBPF genes have a high copy number of the CON1, CON2 and CON3 DUF1220 domains, with all having at least one of each."

12. "While most transcripts predict fusions of NOTCH2NL with other genes (24/26), some transcripts (14/26) predict proteins most similar to human NOTCH2NLR-like sequence with ORF lengths ranging between 235-246 amino acids (AAs). These predicted proteins are similar (p=0.1; t-test one-sided) to human NOTCH2NL ORF lengths (236-274 AAs) (Supplementary Table 3)." Why do the authors propose the test should be one-sided? In addition, do the authors have sufficient statistical power for this test? (sample size of the human group contains 5 observations). Most importantly, a non-significant P-value does not mean that H0 is accepted ("these proteins are similar"), only that H0 cannot be rejected. Moreover, according to Table 3, PTR-NOTCH2NL-5 is predicted to be 146 aa long. In another issue, is the use of the word "like" needed here?

This is a fair point. The numbers are few and tests of significance really do not have much meaning with so few observations. PTR-NOTCH2NL-5 was not included because the copies that predict truncated proteins due to early stop codons were excluded from this analysis. Additionally, "like" is ultimately not needed in this specific instance though we used the word "like" because the copies in NHA are missing at least 1 exon compared to HSA-NOTCH2NLR. We reworded this to remove "like" and the test of significance altogether:
Edited:
"While most transcripts predict fusions of NOTCH2NL with other genes (24/26), some transcripts (14/26) predict proteins most similar to human NOTCH2NLR sequence with ORF lengths ranging between 235-246 amino acids (AAs). These predicted proteins are similar (p=0.1; t-test one-sided) to human NOTCH2NL ORF lengths (236-274 AAs) (Table S3)."

13. "We confirmed that these SDs and gene copies are present in the alternate haplotype of each NHA individual with a small amount of expected copy number variation due to the complexity and large number of duplications in the region (n=9 in chimpanzee, n=9 in bonobo, and n=9 in gorilla)." - It is not clear to me what these numbers represent. Is it the number of different NOTCH2NL SDs in African great apes? If yes, these numbers don't sum up to 26 and differ from the numbers stated earlier in the text.

These numbers represent the number of NOTCH2NL SDs in the alternate haplotype of the NHAs we looked at and do not add up to 26. In the previous section (Independent NOTCH2NL duplications and large-scale restructuring of ape chromosome 1) the count for the primary haplotype is separately

28

reported: "Within NHA SDs we identified 26 NOTCH2NL-sequence-containing gene homologs in chimpanzee (n=9), bonobo (n=10), and gorilla (n=7) in addition to NOTCH2..."

14. "The vast majority (19/26) of these NHA NOTCH2NL-like loci create fusion transcripts between NOTCH2NL at the 5' end and NBPF at the 3' end (Figure 3B), which is similar to what is seen in all human paralogs..." - But in humans it seems as if NOTCH2NL is fused at its 3' to NBPF's 5' (opposite fusion).

This was a mistake of poor wording. In humans the site of fusion for NOTCH2NL-NBPF is at the 3' end of the NOTCH2NL gene and at the 5' end of the NBPF gene because the NOTCH2NL promoter is used, and the same is true for NHA. Thus, the fusion gene itself has a 5' NOTCH2NL end and an NBPF 3' end. To clarify we amend the statement as follows:

Edited:

"The vast majority (19/26) of NHA NOTCH2NL-like loci create fusion transcripts where the site of fusion is at the 3' end of NOTCH2NL and the 5' end of NBPF (Figure 3B), which is similar to what is seen in all human paralogs (not including NOTCH2) (see Transcriptional expression and protein stability of NOTCH2NL paralogs)."

15. The authors use the terms NOTCH2NL-like and NOTCH2NLR-like interchangeably and it's not clear whether the latter is used purposely to refer to a subset of the NOTCH2NL-like transcripts.

In addition to the amended use of NOTCH2NLR-like from one of the above comments, NOTCH2NLR-like is used in the following statements, which have been edited for clarity:

Edited:

"All transcripts and predicted proteins similar in structure to NOTCH2NLR (specifically without additional gene fusion) in NHAs are distinct from human NOTCH2NLR because they have lost either exon 1 (containing the secretory pathway signal sequence) or exon 2 (Figure 3C)."

*Note: this statement was also edited for comments from Reviewer #3.

Edited:

"NHA transcripts also maintain reasonable ORFs ranging between 235-246 AAs for transcripts similar in structure to NOTCH2NLR (without fusion) and 783-4694 AAs for fusion transcripts."

16. "H2, H4, H8, and H9 configurations strictly represent deletion events, which occur in 30% (21/69) of haploid assemblies..." - Seems like there are 23 total individuals in these 4 populations, making it 23/69=33%.

Yes, we appreciate the reviewer catching this miscount. The four haplotypes add up to 23 total individuals, not 21. We are correcting the statement as follows:

Edited:

"H2, H4, H8, and H9 configurations strictly represent deletion events, which occur in 33% (23/69) of haploid assemblies."

17. "with the majority arising from fusion transcripts, and an incorrectly spliced d exon." - Is the "d" here purposely? If yes, then I'm not sure this sentence is clear.

29

We amended the spelling mistake and edited as follows:

Edited:

"However, only ~30% of these transcripts represented canonical full-length transcripts, with the majority arising from fusion transcripts, and an incorrectly spliced exon."

18. "The biased gene conversion that appears to be driving the fixation of NOTCH2NLA and the high level of fourth intron retention among transcripts of NOTCH2tv and NOTCH2NLR may be examples of both refinement and continued evolution of a novel carboxy terminus." - What do the authors mean by "refinement"? And what do they mean by "novel carboxy terminus", if it has been shown that these two paralogs are pseudogenes?

In this context refinement refers to NOTCH2NLA fixation; it has been suggested that combined dosage of any four NOTCH2NLA/B paralogs is more important than possessing strictly two of each for typical neurodevelopment (Fiddes et al. 2018). However, the preference for NOTCH2NLA conversion may indicate a preference for the NOTCH2NL protein in downstream function.

Though NOTCH2NLR and NOTCH2tv are most likely pseudogenes, we suggest these paralogs–particularly NOTCH2tv–may be in the process of becoming stable protein-coding genes. A series of conversion events led to NOTCH2NLA/B/C paralogs as well as a 4 bp deletion, which modified the final fifth exon (relative to NOTCH2NLR), creating an expressed protein with a novel carboxy-terminus compared to NOTCH2 and the prediction for NOTCH2NLR (Fiddes et al. 2018). Other duplicated genes, like TBC1D3, have also gone through similar modifications to the carboxy-terminus in the process of becoming functional (Guitart et al. 2024). NOTCH2tv has gone through conversion with the ancestor, is capable of transcription, and has a single amino acid difference from NOTCH2NLR in the fifth exon; even though there is an excess of fourth intron retention transcripts, which may be considered junk, they could also lead to further carboxy-terminus modification.
Edited:
"The biased gene conversion that is potentially driving the fixation of NOTCH2NLA as well as the high level of fourth intron retention transcripts of NOTCH2tv and NOTCH2NLR may be examples of ongoing evolution in this gene family, such as specification of the functional NOTCH2NL protein and potential acquisition of a novel carboxy-terminus."
**Note: this statement was also edited for comment above.*
19. Figure 1B - It is not clear what the colors represent.
The duplicon locus barcoding is used for visualizing and identifying duplicated regions by location. This is difficult because paralog identity is dynamic, yet using the configuration seen in the majority of haplotypes, we can designate canonical mapping locations for each one using the greater sequence context and DupMasker. DupMasker assigns a color to all duplicated segments and then creates a visual track of the pattern of duplications in a given region. Even though NOTCH2NL paralogs are in SD regions that share high identity, the accumulation pattern of duplications can be quite unique and used as an identifying colorful "barcode." We added this additional text to the Methods section NOTCH2NL-CHM13 region duplication barcodes to clarify our duplicon barcoding approach:
Edited:
"NOTCH2NL-CHM13 region duplication barcodes
30
The duplicon locus barcoding is a computational approach that can readily be used to assess the content and organization of the SDs for any given paralogous locus. It takes advantage of the fact that all SDs can be decomposed into smaller evolutionary units that have accumulated into larger higher-order structures based on their juxtaposition and accumulation over time (Jiang et al. 2007). Based on this repeat graph annotation, the tool DupMasker (Jiang et al. 2008) will encode any SD region into these evolutionary subunits–the orientation and juxtaposition of each becomes a unique identifier that can be color-coded to create a visual track of the pattern of duplications in a given region. Even though NOTCH2NL paralogs are in SD regions that share high identity, the accumulation pattern is effectively unique allowing assignment to specific copies based on the majority-rule consensus for this DupMasker "barcode".
In this study, NOTCH2NL paralog sequences plus 1 Mbp surrounding each gene were used as input for DupMasker. The .duplicons output file was processed into a .txt file to create visual tracks of duplicon barcodes for each region."
20. Figure 1C - What is the meaning of the direction of the long black arrows that are within the gene? Is it its direction? According to the CHM13-T2T assembly on the genome browser, the NBPF gene is downstream of the NOTCH2NLA gene.
The colored tracks of arrows represent all the different duplicated sequences present in a given region. These segments, also known as duplicons, are independent of genes; some do not even contain gene sequences. In DupMasker these duplicons are defined using multiple lineages, so the directionality is likely because they are inferred based on an ancestral sequence position.
21. Figure 2 - What is the meaning of the "N" in the dark red circle? In addition, the figure is a little overwhelming. I suggest removing the NBPF circles, and possibly the red & green arrows.
N in the dark red circle is there to represent the neocentromere, a centromere repositioning event in the Old World monkey ancestor. To clarify we added an additional sentence to the Figure 2 legend:
Edited:
"The circled numbers represent previous ancestral states of chromosome 1. The circled N represents a centromere repositioning event (N, neocentromere). Three distinct evolutionary inversions are

predicted (I, II, III)."

22. Figure 3B - It is not always clear which gene name corresponds to which gene schematic.

Using group 1 as an example, each gene name doesn't correspond to a different transcript, but all five NHA genes produce these two types of transcripts. To clarify we edited Figure 3B and the legend as follows:

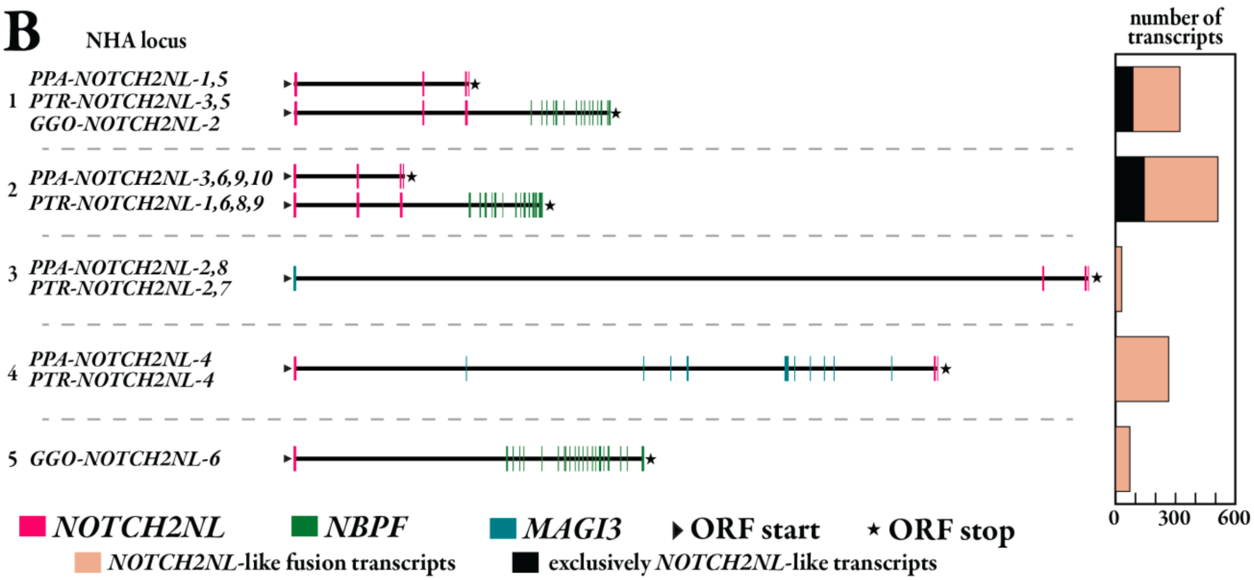

Updated Figure 3B.

Edited:

"b) Examples and abundance of five different sets of transcripts, which names the locus (across multiple species) (left) and illustrates a schematic (middle) of all the transcripts that can be produced by the groups of loci. These sets are representative of 20/26 NOTCH2NL-like loci in NHA from testis, fibroblast/lymphoblastoid cell lines, iPSCs, neuroepithelium, and neural progenitor cells. The histogram to the right of each set of models represents the number of Iso-Seq transcripts in support of the different predicted models at the locus. Groups with more than one type of transcript being produced have a section for exclusively NOTCH2NL-like transcripts (black) in addition to the NOTCH2NLlike fusions (orange)."

23. And if NBPF is fused at its 3' end to NOTCH2's 5' end, then shouldn't the ORF start and end be reversed?

NBPF is not fused as its 3' end to NOTCH2's 5' end. The 5' end of NBPF is fused to NOTCH2NL's 3' end, thus NOTCH2 is the 5' end and NBPF the 3' end of the gene fusion.

24. And what is the size of each window? Units should be added to the right bar plot. And what is the meaning of the right bar plot if each bar represents a different number of genes? And the use of the exact same colors as in A, when there's no connection between the two, can be confusing.

We appreciate the reviewer bringing this up as the right barplot should be labeled as the raw count of each transcript group instead of the abundance and doesn't require units. Each transcript group (which may be shared across multiple apes) for the most part has one type of transcript produced. However, the first two groups make an additional type of transcript, which is represented by the second color in the histogram. The response to comment 22 above also addresses this clarification. To further address these confusions, we edited all of Figure 3 for clarity:

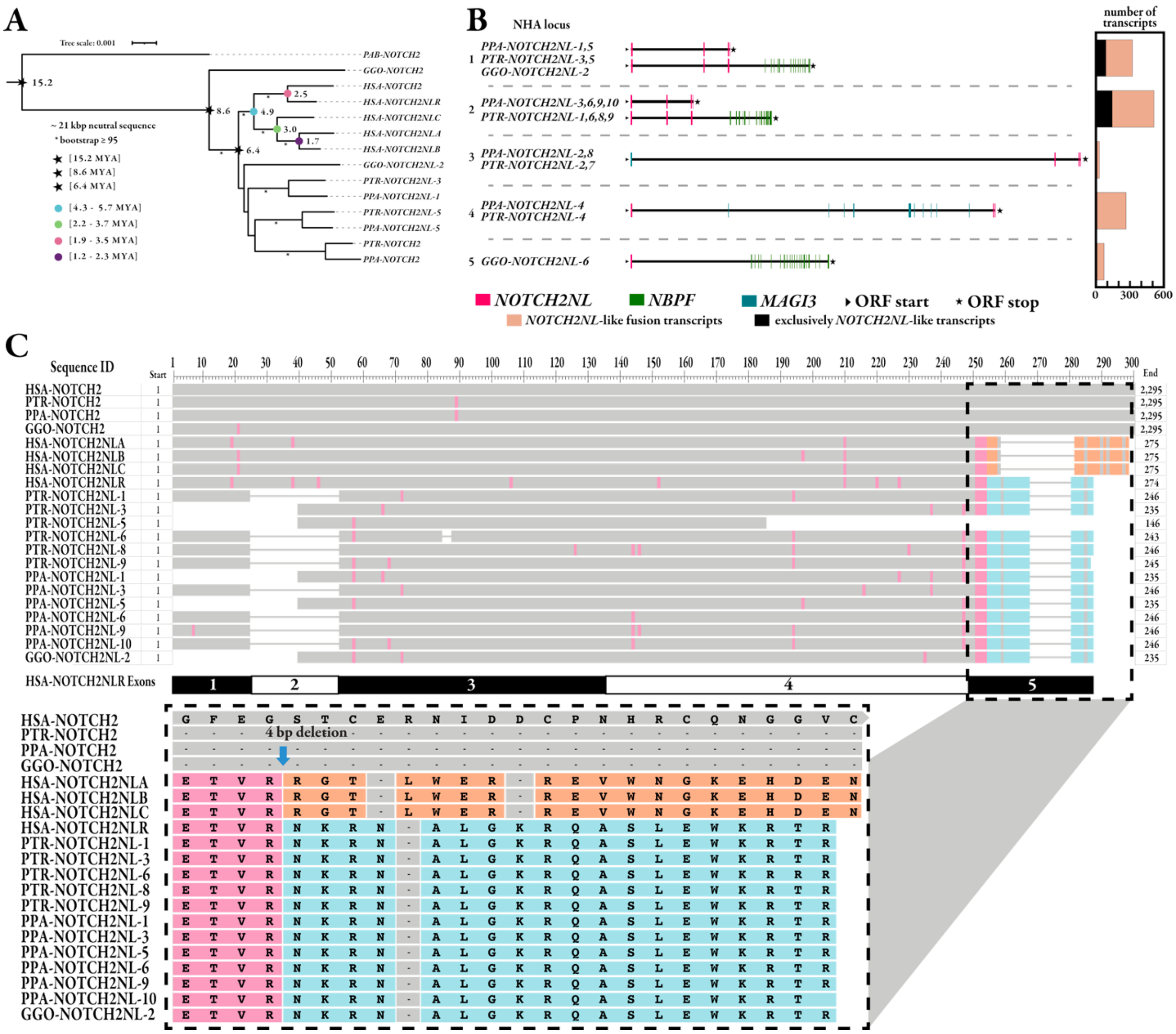

Updated Figure 3.

25. *Figure 4B - I think this is better suited as a supplementary figure.*

While we understand that this figure contains a lot of data, we're hesitant to remove panel B and move it to the supplement because it contains fundamental data that is important to the reader.

26. *Figure 5A - How do these configurations distribute among the various populations?*

In a previous response to Reviewer #1, we included an addition to Figure 5A (below), which addresses the distribution of these configurations across Africans and non-Africans. With the large number of populations, we assess a small sample size relative to that, and a population-aware analysis would not be powerful enough for significance. However, we do see several haplotypes are enriched in African ancestry suggesting they could potentially be ancestral haplotypes.

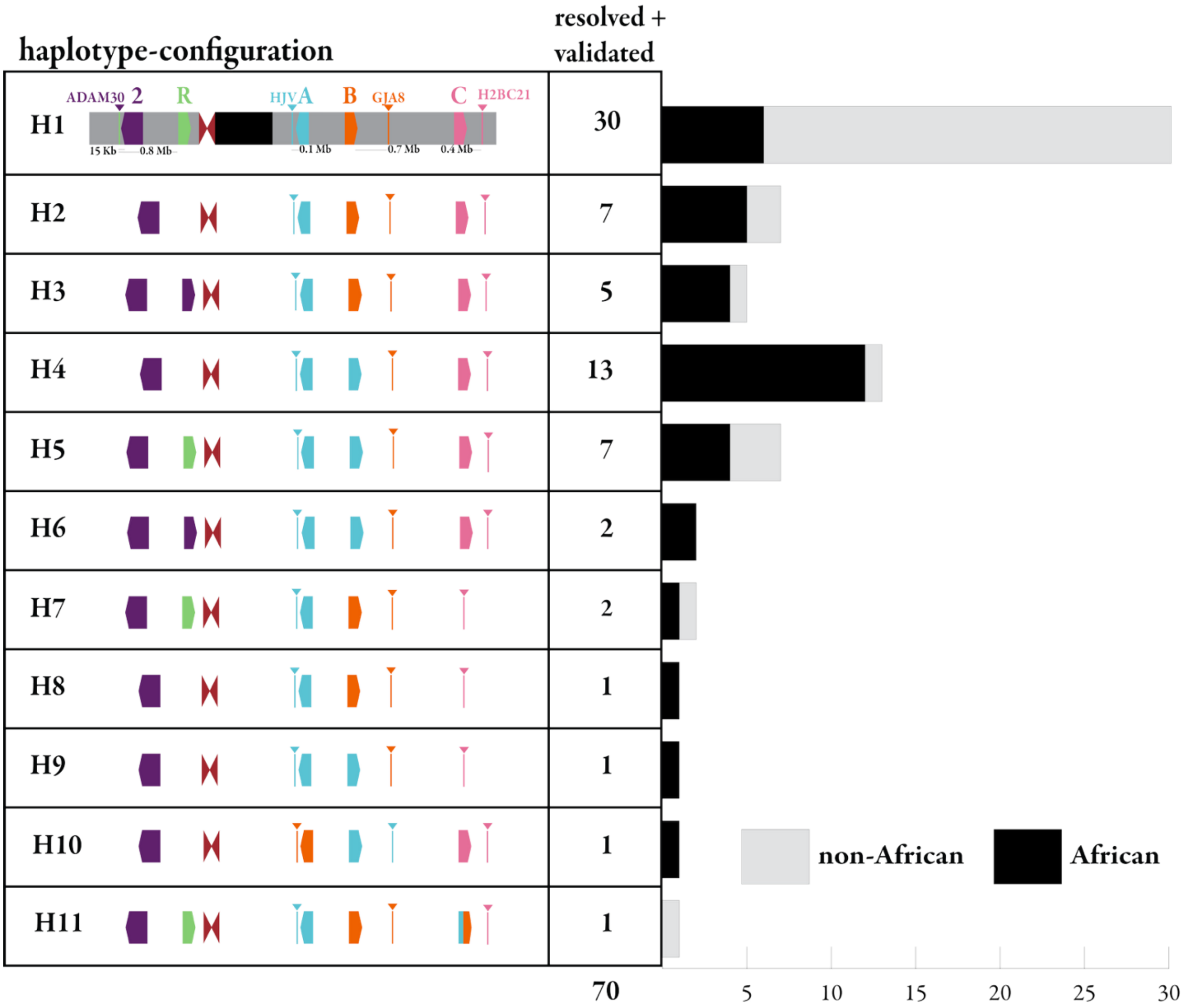

Updated Figure 5A.

27. In the figure text - "A simplified schematic summary of the NOTCH2NL haplotype organization and frequency based on 66 sequence-resolved HPRC genomes and the T2T-CHM13 reference." - Isn't it 69 though?

Yes, the final HPRC count should be 69. We edited the Figure 5 legend as follows:

Edited:

"a) A simplified schematic summary of the NOTCH2NL haplotype organization and frequency based on 69 sequence-resolved HPRC genomes and the T2T-CHM13 reference."

34

Reviewer #3: We found the manuscript, "Genetic diversity of regulatory features of human-specific NOTCH2NL duplications," to be an informative and enjoyable read. This manuscript represents a thorough analysis of the gene family containing NOTCH2NL, using a variety of recently published and nearly complete genomes from the non-human apes, and scores of humans. Additionally, the authors use Fiber-seq to annotate regulatory elements in the paralogs of this family, which could not be done efficiently with previous methods. The authors: find that this family independently expanded in great ape lineages, discovered a new member of the family (NOTCH2tv), and showed that the degree of open chromatin over a regulatory element is often variable across paralogs in the family.

Our overall enthusiasm for this work is high, but we do have some comments that we think should be addressed in a revised version of the manuscript.

We would like to thank Reviewer #3 for their kind comments and appreciating the novel aspects of the work. We have revised the manuscript based on their feedback.

Comments:
1) As mentioned in the manuscript (Figure 6), the different NOTCH2 paralogs have very different transcript abundances, with NLA and NLB being the highest. However, all of the NOTCH paralogs have similar numbers of open chromatin regions in the Fiber-seq data. Can you speculate on why this might be (e.g. mixture of enhancers and repressors, some regulatory elements don't act on a NOTCH2 paralog)?
Chromatin accessibility, even for unique genes, is not always a perfect proxy for transcript abundance. As pointed out by the referee, both repressor and enhancer domains in noncoding DNA, are accessible by Fiber-seq. We added a note to this effect in the limitations section, which was also added in response to Reviewer #1:
"Gene regulation and Fiber-seq. Fiber-seq is a measure of chromatin accessibility, which is only a proxy for noncoding regulatory regions; it does not distinguish between element types, such as repressors and enhancers, nor address which genes these elements would be regulating. Although FiberFold will generate predictions of which genes may be regulated by these accessible chromatin peaks, further efforts are needed to functionally characterize these predictions.
Iso-Seq analyses. Iso-Seq is a measure of steady state transcripts, which is going to be influenced by both the potency of regulatory elements as well as the stability of the full-length transcript. Given the marked divergence between NOTCH2NL paralogs in terms of the surrounding regulatory elements as well as their 3' UTR structure, it is anticipated that both of these are contributing to the final steady state measurements of transcript abundance."
However, as shown in response to Reviewer #2, we used FiberFold to predict TADs associated with the TSS of NOTCH2NL paralogs in HG02630 brain organoid data. This revealed that each highly identical paralog resides in distinct predicted 3D genomic environments. Specifically, in nearly 800 kbp windows centered around TSSs, we find 3D landscapes unique to specific paralogs (Figure S9A). Overall, our FiberFold analysis gives a more fine-scale map of the putative regulatory architecture of NOTCH2NL paralogs and suggests that the most relevant accessible regions are paralog-specific in size and the number of chromatin accessible elements.
2) It is not clear from figure 1B how the locus barcoding works.
SD barcoding is a strategy that we developed in the Eichler lab to track the organization of segmental duplication blocks based on content rather than sequence identity matches which can be readily
35
confounded by the process of gene conversion. In brief, it encodes content based on ancestral minimal *shared segments called "duplicons" defined based on synteny mapping among mammals. Realizing this is probably foreign to most readers, We edited the following text in the Methods as described to be much more explicit:*
Edited:
"NOTCH2NL-CHM13 region duplication barcodes
The duplicon locus barcoding is a computational approach that can readily be used to assess the content and organization of the SDs for any given paralogous locus. It takes advantage of the fact that all SDs can be decomposed into smaller evolutionary units that have accumulated into larger higher-order structures based on their juxtaposition and accumulation over time (Jiang et al. 2007). Based on this repeat graph annotation, the tool DupMasker (Jiang et al. 2008) will encode any SD region into these evolutionary subunits–the orientation and juxtaposition of each becomes a unique identifier that can be color-coded to create a visual track of the pattern of duplications in a given region. Even though NOTCH2NL paralogs are in SD regions that share high identity, the accumulation pattern is effectively unique allowing assignment to specific copies based on the majority-rule consensus for this DupMasker "barcode".
In this study, NOTCH2NL paralog sequences plus 1 Mbp surrounding each gene were used as input for DupMasker. The duplicons output file was processed into a .txt file to create visual tracks of duplicon barcodes for each region."
3) It appears by cross-referencing Figure 3B and Figure 6C, that NHA NOTCH2 paralog transcripts are fusion transcripts more often than human NOTCH2 paralog transcripts?
Yes, this is correct, and we thank the reviewer for catching this point. We added details to the text to address this comment:
Edited:
"We found distinct differences within the transcript abundance of each of the NOTCH2/NL paralogs

(Figure 6C), indicating that these paralog-specific accessible chromatin elements may be creating unique gene regulatory environments for each of the NOTCH2/NL paralogs. Specifically, NOTCH2NLA and NOTCH2NLB had ~3-fold higher steady-state transcript abundance than the other NOTCH2/NL paralogs. Ancestral NOTCH2 has a low abundance of full-length transcripts; however, NOTCH2 also has very few other transcript types, including fusions. In fact, NHAs appear to show higher levels of NOTCH2NL fusion transcripts than seen in humans (Figure 3B, 6C)."

4) On page 9, the authors reference Figure 3B to make the point that either exon 1 or 2 of the NOTCH2NL transcripts are lost. Perhaps the authors should reference Figure 3C here instead?

We thank the reviewer for this suggestion and agree that 3C makes this point clearer. We edited the statement as follows:

Edited:

"All transcripts and predicted proteins similar in structure to NOTCH2NLR (specifically without additional gene fusion) in NHAs are distinct from human NOTCH2NLR because they have lost either exon 1 (containing the secretory pathway signal sequence) or exon 2 (Figure 3C)."

**Note: this statement was also edited for comments from Reviewer #2.*

5) The start of the results section describes the similarities and differences between the genetic structure of the paralogs. This is well described using figures and tables, and the authors can likely make this part of the text more concise.

We appreciate this reviewer's insight, so we tried to streamline the text to make the beginning of the results section more concise:

Edited:

"Structure of the NOTCH2NL gene family in a complete human genome assembly

To establish a framework for studying the NOTCH2NL gene family in humans, we first characterized its structure of this gene family along the fully assembled T2T-CHM13 haploid genome (Nurk et al. 2021). In T2T-CHM13, NOTCH2 and NOTCH2NLR are clustered on the p-arm of chromosome 1 within 1.5 Mbp of the centromere (Figure 1A) NOTCH2NLA, NOTCH2NLB, and NOTCH2NLC map to the q-arm (Figure 1A) and define the functional human-specific duplications. All daughter duplications are approximately 10-11 kbp proximal upstream of an NPBF gene, which has been implicated as a partner in creating NOTCH2NL fusion transcripts (Figure 1A) (Fiddes et al. 2019). While all NOTCH2NL genes structurally have five exons, In T2T-CHM13, NOTCH2NLA and NOTCH2NLC gene models are distinct from the other paralogs because their first exon is suggested by gene annotation to be untranslated (Figure 1B), due to unique mutations that remove the canonical NOTCH2 initiator methionine and secretory signal (Fiddes et al. 2018). However, it has been suggested in both paralogs that a nonstandard CTG initiation allows transcription and translation of nearly the entire canonical protein (Lodewijk et al. 2020). Unlike the previous assembly, GRCh38, we find that NOTCH2NLB shares the same initiator methionine disruption typically attributed to NOTCH2NLA. A striking structural difference between T2T-CHM13 and GRCh38 is a nearly 2.5 Mbp inversion of sequence around NOTCH2NLB, which changes the orientation of the gene (Figure S1).

To characterize the extent of synteny among the NOTCH2NL SDs, we performed a selfalignment of T2T-CHM13 defining the length and percent identity of each paralogous locus (Methods) to all others (Table 1, Figure 1C). We find the largest stretch of synteny (~557 kbp) occurs between NOTCH2NLB and the ancestral copy NOTCH2. However, the most identical stretch and second largest segment of shared sequence is between NOTCH2NLA and NOTCH2NLB (99.7% identity over 416 kbp). Across all NOTCH2NL paralog regions, the average longest length of synteny and percent identity is ~287 kbp and 99.4%. Each NOTCH2NL copy has unique single-nucleotide variants (SNVs) that distinguish copies on T2T-CHM13, but the high degree of sequence identity over large swaths of the genome creates ample opportunities for IGC as well as non-allelic homologous recombination (NAHR) (Dumont and Eichler 2013, Vollger et al. 2022, Vollger et al. 2023, Sharp et al. 2006) in other human haplotypes.

We hypothesized that using the larger sequence context surrounding each paralog would uniquely identify the reference position of each NOTCH2NL gene due to the accumulated pattern of duplications/duplicons over time in each paralog's history (Marques-Bonet and Eichler 2009). We, therefore, used the order and orientation of duplicons flanking each NOTCH2NL copy as defined by DupMasker (Jiang et al. 2008) to generate a "barcode" of each locus to readily identify regions in other

human genomes when compared to T2T-CHM13 (Figure 1B, Methods). Representing the surrounding sequence in terms of the higher-order duplication content over 1 Mbp regions helped define orthologous locations in the presence of the homogenizing effects of IGC (Vollger et al. 2023).
Specific pairs of paralogs show extended homology (Figure 1C) as well as structural differences of potential functional consequence. NOTCH2 and NOTCH2NLR, for example, show extended homology that is maintained between NOTCH2NLA and NOTCH2NLB, where synteny extends even farther into the corresponding NBPF region that follows the NOTCH2NL copies. Importantly, all copies of NOTCH2NL show one breakpoint with respect to the ancestral NOTCH2 corresponding to the NBPF duplicon that

37

demarcates the 3' end of each derived-duplicated gene (Figure 1C). Additionally, the presumptive pseudogene NOTCH2NLR is missing the upstream nongenic ancestral NOTCH2 sequence present in NOTCH2NLA and NOTCH2NLB. NOTCH2NLC appears the most structurally divergent; most paralog regions share the bulk of synteny upstream of the gene model, yet the opposite is true for NOTCH2NLC. Overall, NOTCH2NLC shares the largest number of homologous alignments with NOTCH2NLR."

6) Is it possible to speculate on the function/consequence of the fusion proteins between NOTCH2NL and LRIG2 or SORT1?

LRIG2 is a pleiotropic gene associated with cell proliferation, like NOTCH2NL, while SORT1 has a role in brain lysosomal trafficking associated with dementia. However, we only have evidence that fusion transcripts form as a result of the juxtaposition of flanking SDs and the discovery of long-read transcripts. Based on our analysis of chimpanzee as compared to human where we have little evidence that the fusion transcripts are functional, we speculate that the purpose of these fusions is to act as an intermediary between an immature gene duplication and one that has evolved protein-coding function. In the case of human, for example, a greater fraction of transcripts is no longer associated with fusions. We added additional explanation to the discussion to expand on the role of fusion transcripts.
Edited:
"The basis for this recurrence or genomic instability is unknown, but it is interesting that most NOTCH2NL ape copies are also associated with the NBPF duplicon—an association that is postulated to have coevolved both in terms of structure and transcriptional regulation, especially in humans (Fiddes et al. 2019). NBPF is one of about a dozen core duplicons (along with TBC1D3, LRRC37, and NPIP) implicated as a potential driver of interspersed SDs in the primate lineage (Jiang et al. 2007, Dumas and Sikela 2009, O'Bleness et al. 2012). Our study finds evidence of fusion transcripts between NOTCH2NL and NBPF across multiple ape species (particularly in NHAs), supporting co-evolution, but finds no particular evidence of gene/protein function. The same is true for the newly discovered fraction of transcripts fused with LRIG2 and SORT1, which are associated with cell proliferation and lysosomal trafficking in the brain, respectively. Thus, we hypothesize that the role of these fusions is to act as an intermediary between immature gene duplications and one that has evolved protein-coding function. Of note, a comparative analysis of NBPF associated with NOTCH2NL reveals species-specific expansions of different portions of the NBPF DUF1220 in different apes (Figure S5), so it is possible that NBPF plays a more general role in gene innovation in ape species other than human."

7) When the authors state that polarized signals of gene conversion suggest selection, is the idea that gene conversion is likely to be happening in both directions, but we only observe one direction because one is deleterious/advantageous? If so, perhaps that could be explicitly stated.

Yes, we hypothesize that gene conversion is equally likely in either direction, but that a functional advantage of a copy like NOTCH2NLA may lead to polarized signal of gene conversion. However, without any strong signals of selection in our data, as well as no clear findings that the gene conversion event is recurrent, it is difficult to speculate on the forces that lead to polarization. To clarify we edited this section of discussion as follows:
Edited:
"We also encounter polarized signals of gene conversion, which seems especially significant in the case of NOTCH2NLA, the only paralog that has expanded and is present in all assemblies, seemingly at the expense of NOTCH2NLB. This may be a consequence of either an unknown mechanistic or selective bias favoring NOTCH2NLA as a donor."

Referees' reports, second round of review

Reviewer #1: I thank the authors for thoroughly addressing my earlier concerns. In principle, I am satisfied with the overall study and support its publication. I have a few additional comments and suggestions that may help improve flow and readability for the general audience. These suggestions are optional and should not delay acceptance of the manuscript.

Readability and Glossary: The readability of the paper has improved substantially. That said, the study remains complex, with multiple moving parts. The glossary is an excellent idea; the authors might even consider moving it into the main text to aid readers navigating the manuscript.

High-Level Summary Figure: The authors should consider adding a high-level summary or overview diagram as the first figure. A stepwise schematic—from the initial duplication event, through subsequent sequence and evolutionary changes, to the major regulatory outcomes—would help readers situate the main results. A graphical abstract could also serve this purpose.

Figure 1 Clarity: Figure 1 is difficult to follow and visually crowded. While the genomic regions involved are large and inherently complex, the presentation could be simplified. Panel A in particular would benefit from clearer gene model representation, even simple arrows or schematic icons would help. Additionally, the legend for Panel B should be expanded with full explanations of colors, symbols, groupings, and highlights. Improving annotation and labeling in both the figure and legend would make the results more accessible. Figure 2 is especially clear and could serve as a guide for refining Figure 1.

Phylogeny Resolution (Figure 4B): Providing a higher-resolution, fully annotated version of the phylogeny shown in Figure 4B as a supplementary figure would strengthen the presentation and improve interpretability.

Clarification of Gene Conversion Statement (Page 3): The sentence "Mutational processes such as interlocus gene conversion (IGC) (Chen et al. 2007) make paralogous sequence variants unreliable as tags unless such IGC patterns in diverse humans are fully characterized" should be revised for accuracy. Even if IGC patterns are well characterized in population-scale datasets, individual genomes may contain recent or private IGC tracts that are absent from population catalogs and therefore remain undetectable.

Reviewer #2: I appreciate the authors' efforts in revising the manuscript and addressing my earlier comments. Several important issues remain:

Major points:

1. Interpretation of paralog-specific regulatory sequences. Figure 6D suggests that NOTCH2NLA harbors many regulatory sequences that are both sequence- and accessibility-specific, whereas NOTCH2NLB does not. It is not fully clear where these NLA paralog-specific sites lie relative to the gene body, but Figure 6B implies that most are located approximately 100-200 kb downstream. Given that NLA and NLB exhibit similar transcription levels (Fig. 6C), the distance of these putative regulatory elements raises concerns about their specificity and the likelihood of interaction with NLA. Furthermore, the FiberFold analysis (Figure S9C) indicates that these accessible sites fall outside the TAD containing NLA, which appears inconsistent with the authors' interpretation. These points should be reconciled.

2. Claims regarding copy-number constraint and paralog-specific regulatory elements. The Discussion concludes with the statement "The data also allow us to now predict which of the many duplicate genes are under the greatest copy number constraint (NOTCH2NLA). Finally, by applying a novel long-read sequencing-based approach, Fiber-seq, to human brain organoids generated from iPSC, we provide the first detailed comparative open chromatin accessibility map and show that ~14% of these sites are paralog-specific providing a roadmap to exploring transcriptional regulatory differences for the different members of this gene family.". By "copy number constraint" do the authors refer to the fact that all examined human genomes contain an NLA paralog? The data indicate that due to the NLB-to-NLA IGC, this paralog does not appear to be under copy number constraint. In addition, the identification of paralog-specific open

chromatin peaks appears to rely on an arbitrary distance cutoff around each paralog. Given both this choice and the FiberFold structural results, the strength of the claim should be moderated or the authors should adopt more biologically meaningful boundaries, such as TAD limits.

3. Null hypothesis for regulatory divergence between paralogs. The comparison of regulatory variation between paralogs to variation between alleles of a single locus is conceptually inappropriate. Alleles share genomic context and are nearly identical in sequence, while paralogs differ in both sequence and genomic environment; regulatory divergence is therefore expected. Demonstrating that paralogs differ from alleles does not test a meaningful null hypothesis, as such differences are expected. A more informative analysis would compare regulatory divergence between NOTCH2NL paralogs to divergence among other paralog pairs genome-wide. If the authors wish to argue for accelerated functional divergence within the NOTCH2NL family, this should be quantified relative to an appropriate background distribution.

4. Figure clarity. The time-course figure is valuable, but the overall clarity of most figures did not improve despite suggestions, and remains suboptimal. Improving visual clarity would greatly enhance readability.

Minor points:

*** Figure S9B: The labels on the x-axis appear incorrect for the first four entries, and the figure overall is difficult to interpret.**
*** Figure S10: The distinction being shown is unclear (haplotypes?). If so, comparisons across paralogs should also be displayed.**
*** Summary/abstract - "we show independent recurrent duplication among apes with protein-coding copies emerging in humans ~2.1 million years ago" - For consistency, I think this number should match the number in the introduction (~2).**
*** The manuscript introduces the human NOTCH2NL paralogs for the first time in the context of disease associations. A brief overview of the paralog set and current knowledge should precede discussion of phenotypic consequences.**
*** It is unclear how the authors infer that the NLB-to-NLA conversion occurred ~2.8 MYA, especially given that the authors also claim that these two paralogs diverged ~1.7 MYA (figure 3A) and that the last common ancestor of modern humans lived much later. (Results, Patterns of NOTCH2NL human genetic variation, end of 4th paragraph).**
*** Figure S9A: The y-axis should likely read "distance (Mb).**
*** Point 7 (previous) - After editing, it is still unclear to me what we learn from this statement. What is the conclusion regarding the emergence and modification of the various NOTCH2NL paralogs? More specifically, the authors claim that NLC arose from a duplication independent of NLR (Fig. 3A). Therefore, how do the authors explain the observation that NLC shares the largest number of homologous alignments with NLR?**
*** Point 8 (previous) - the response remains unclear. In addition, the text in the response does not match the text in the manuscript ("are" should be singular).**

Reviewer #3: The authors have addressed our comments. We congratulate the authors on a great manuscript!

Authors' response to the second round of review

Reviewer #1:
I thank the authors for thoroughly addressing my earlier concerns. In principle, I am satisfied with the overall study and support its publication. I have a few additional comments and suggestions that may help improve flow and readability for the general audience. These suggestions are optional and should not delay acceptance of the manuscript.
Readability and Glossary: The readability of the paper has improved substantially. That said, the

study remains complex, with multiple moving parts. The glossary is an excellent idea; the authors might even consider moving it into the main text to aid readers navigating the manuscript.

We thank the reviewer for this suggestion and, pending the editor's approval, we have moved the glossary to the main text of the paper.

High-Level Summary Figure: The authors should consider adding a high-level summary or overview diagram as the first figure. A stepwise schematic—from the initial duplication event, through subsequent sequence and evolutionary changes, to the major regulatory outcomes—would help readers situate the main results. A graphical abstract could also serve this purpose.

We agree that the reviewer's suggestion that a graphical abstract would help summarize the main results and have created one, which is below:

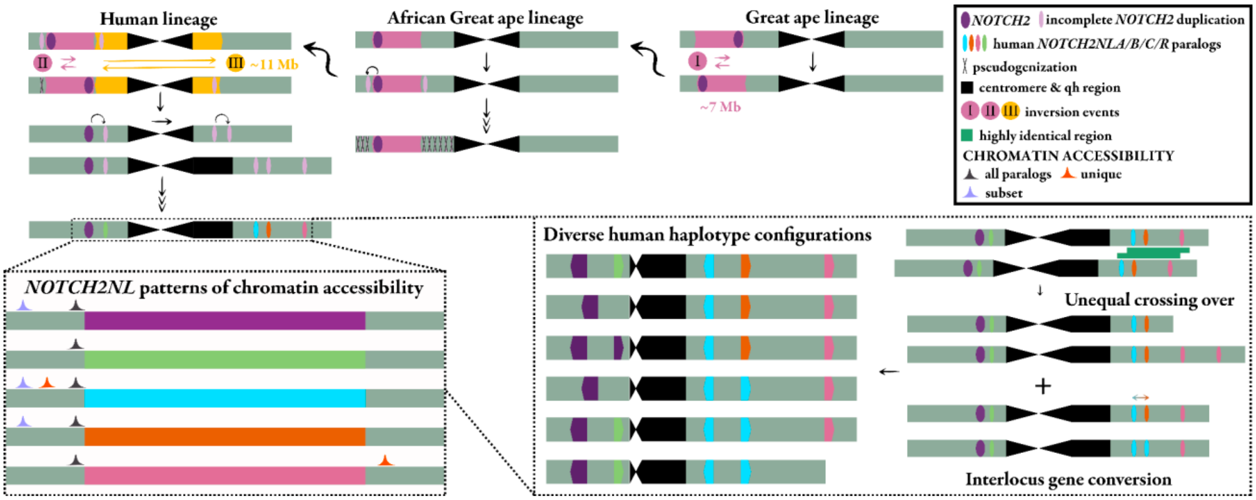

Graphical Abstract: Structural variation, evolution, and chromatin accessibility of NOTCH2NL loci. Schematic showing large-scale structural variation during ape evolution associated with the duplication of incomplete NOTCH2NL copies (top panel). Continued unequal crossover and interlocus gene conversion in humans (bottom right panel) creates a diverse set of haplotypes at different locations on chromosome 1 with distinct patterns of

chromatin accessibility (bottom left panel) as potential sites for future investigation into the regulatory architecture of the NOTCH2NL gene family.

Figure 1 Clarity: Figure 1 is difficult to follow and visually crowded. While the genomic regions involved are large and inherently complex, the presentation could be simplified. Panel A in particular would benefit from clearer gene model representation, even simple arrows or schematic icons would help. Additionally, the legend for Panel B should be expanded with full explanations of colors, symbols, groupings, and highlights. Improving annotation and labeling in both the figure and legend would make the results more accessible. Figure 2 is especially clear and could serve as a guide for refining Figure 1.

Per the reviewer's suggestion, we simplified Figure 1A by changing the exons/introns to simple arrows and removed the middle transcript track. To avoid overcrowding of Panel B, we pointed referees to a new supplementary table (attached, Table S6) where we define the DupMasker duplicon number, associated color, and ancestral location in the database.

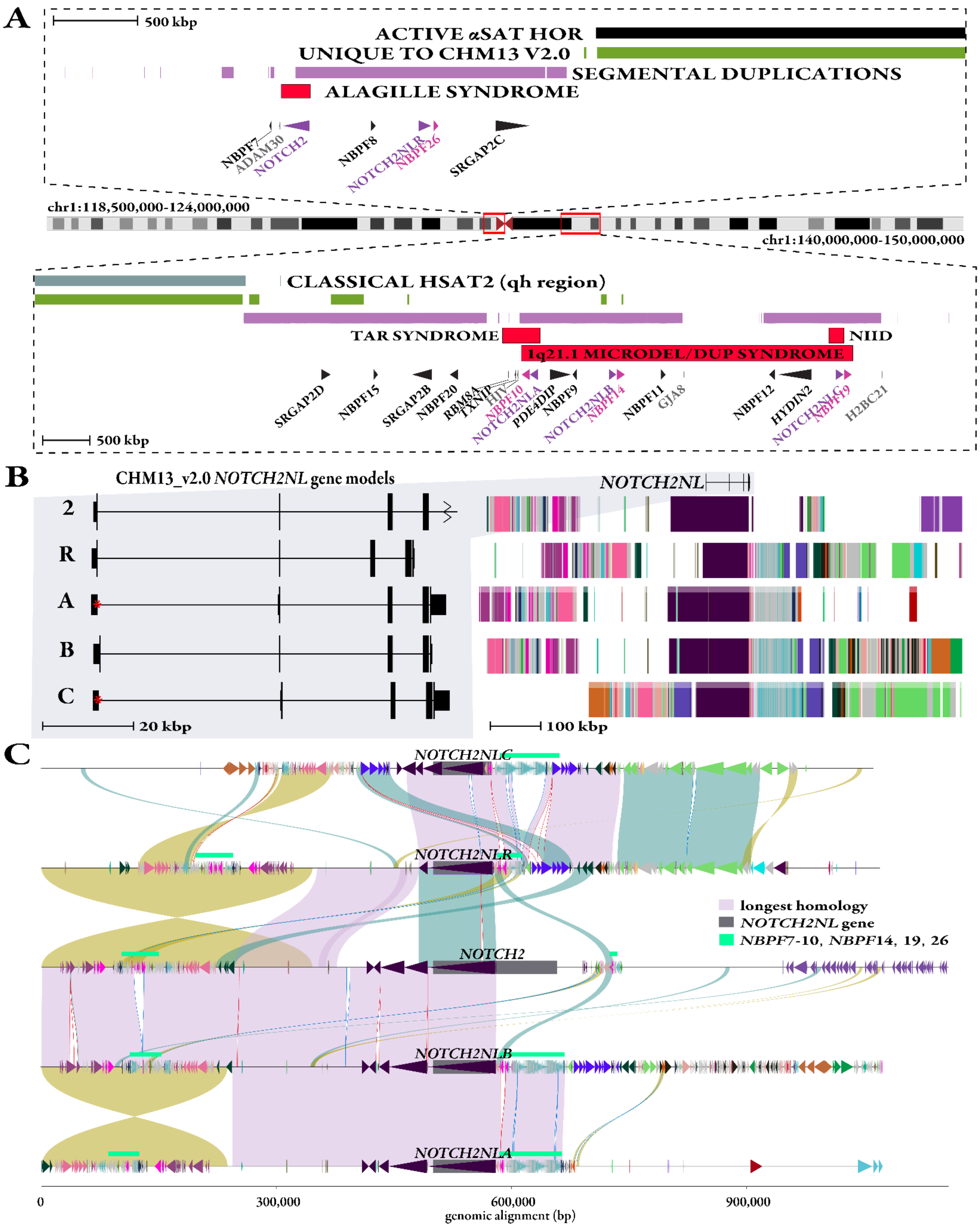

Updated Figure 1. Genome structure and organization of the NOTCH2NL gene family.

Phylogeny Resolution (Figure 4B): Providing a higher-resolution, fully annotated version of the phylogeny shown in Figure 4B as a supplementary figure would strengthen the presentation and improve interpretability.

We added a high resolution PDF of the phylogeny in Figure 4B, including annotation of the longrange duplicon structures that surround the paralogs, to the supplemental materials.

Clarification of Gene Conversion Statement (Page 3): The sentence "Mutational processes such as interlocus gene conversion (IGC) (Chen et al. 2007) make paralogous sequence variants unreliable as tags unless such IGC patterns in diverse humans are fully characterized" should be revised for accuracy. Even if IGC patterns are well characterized in population-scale datasets, individual genomes may contain recent or private IGC tracts that are absent from population catalogs and therefore remain undetectable.

We edited the text to qualify that these are common IGC patterns and distinguish from rarer private events.

Edited:

"Mutational processes such as interlocus gene conversion (IGC) (Chen et al. 2007) make paralogous sequence variants unreliable as tags unless such common IGC patterns in diverse humans are characterized. However, even if such common patterns of IGC are resolved, based on the frequency of IGC (Vollger et al. 2024) we expect rarer events, including private stretches of IGC not readily identified in population datasets, to still complicate associations."

Reviewer #2:

I appreciate the authors' efforts in revising the manuscript and addressing my earlier comments. Several important issues remain:

Major points:

1. Interpretation of paralog-specific regulatory sequences. Figure 6D suggests that NOTCH2NLA harbors many regulatory sequences that are both sequence- and accessibilityspecific, whereas NOTCH2NLB does not. It is not fully clear where these NLA paralog-specific sites lie relative to the gene body, but Figure 6B implies that most are located approximately 100-200 kb downstream. Given that NLA and NLB exhibit similar transcription levels (Fig. 6C), the distance of these putative regulatory elements raises concerns about their specificity and the likelihood of interaction with NLA. Furthermore, the FiberFold analysis (Figure S9C) indicates that these accessible sites fall outside the TAD containing NLA, which appears inconsistent with the authors' interpretation. These points should be reconciled.

We appreciate the reviewer's point about the distinction between regions of chromatin accessibility and regulatory elements that specifically control NOTCH2NL expression. We wish to clarify that our investigation is meant to generally characterize chromatin accessibility around each copy providing potential candidates for further functional characterization as opposed to functional characterization of the role of each region with respect to NOTCH2NL gene regulation. As we discuss in the manuscript, given the deficiency of short-read approaches for mapping to SDs, the fundamental map of both chromatin accessibility regions and potential regulatory elements along these SDs is still almost completely unknown. We agree it is unlikely that many of the unique NOTCH2NLA region elements located far downstream are directly regulating the NOTCH2NLA gene, but it was still important to our study to fully characterize the chromatin architecture of these SDs. Thus, we don't consider our addition and interpretation of FiberFold inconsistent, as it was meant to define a region that can be physically associated with the NOTCH2NL promoters within the context of candidate enhancer regions. To make this clearer, we revised the text to specifically highlight the region, its relationship to the TSS, and the overall TAD boundary for this locus making it clear that further experiments will be required to functionally characterize which elements are directly regulating NOTCH2NL genes. In addition, we added a supplemental figure of selected regulatory element analyses restricted by the TAD boundaries:

Edited:

"FiberFold predicts each NOTCH2NL paralog occupies a distinct 3D genomic environment

despite their highly identical underlying sequence, with each NOTCH2NL TSS surrounded by a unique 3D landscape (Figure S9A). These paralog-specific 3D landscape patterns are largely consistent across haplotypes, with differences between paralogs more pronounced than differences between haplotypes (Figure S9B). Consistent with this, we also find that chromatin actuation differences between paralogs are more pronounced than differences between haplotypes (Figure S10), despite the high sequence identity between paralogs. Limiting this analysis to accessible chromatin elements that fall within the predicted TADs for each of the NOTCH2NL paralogs confirmed the above findings (Figure S11) and exposed a cluster of elements 63 kbp upstream of the NOTCH2, NOTCH2NLA, and NOTCH2NLB TSSs, which are selectively active on the NOTCH2NLA/B SDs but inactive on the NOTCH2 SD (Figure S9C). Overall, this analysis uncovers marked SD-specific accessible chromatin architectures and nominates specific elements that may be resulting in the paralog-specific regulation of the NOTCH2NL genes. However, further experiments will be needed to characterize the functional role of elements within TAD boundaries."

Edited to add:

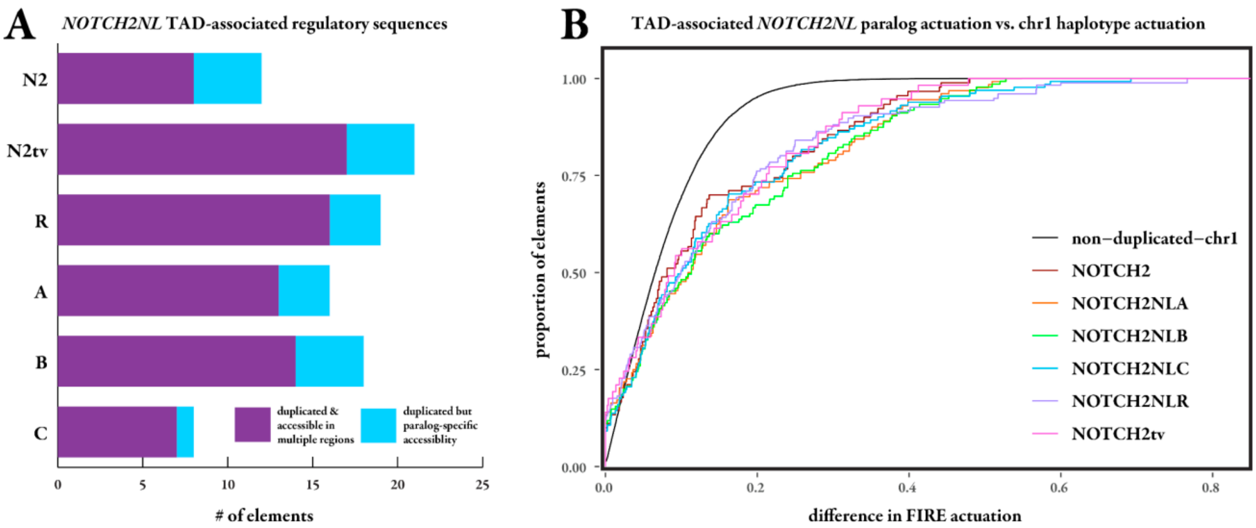

Figure S11. TAD-restricted regulatory analysis. a) Bar graph showing categorization of the TAD-restricted accessible elements surrounding each NOTCH2NL paralog based on the presence of duplicate sequence and accessibility at that sequence on the different paralogs. b) A TAD-restricted model of expected haplotype differences on chromosome 1 using brain organoid data mapped to T2T-CHM13 (black) also shows a higher proportion of regulatory elements with less actuation difference than what is seen for the different NOTCH2NL paralogs.

2. Claims regarding copy-number constraint and paralog-specific regulatory elements. The Discussion concludes with the statement "The data also allow us to now predict which of the many duplicate genes are under the greatest copy number constraint (NOTCH2NLA). Finally, by applying a novel long-read sequencing-based approach, Fiber-seq, to human brain organoids generated from iPSC, we provide the first detailed comparative open chromatin accessibility map and show that ~14% of these sites are paralog-specific providing a roadmap to exploring transcriptional regulatory differences for the different members of this gene family.". By "copy number constraint" do the authors refer to the fact that all examined human genomes contain an NLA paralog? The data indicate that due to the NLB-to-NLA IGC, this paralog does not appear to be under copy number constraint. In addition, the identification of paralog-specific open chromatin peaks appears to rely on an arbitrary distance cutoff around each paralog.

Given both this choice and the FiberFold structural results, the strength of the claim should be moderated or the authors should adopt more biologically meaningful boundaries, such as TAD limits.

This is also a fair point. The original statement that NOTCH2NLA is the paralog under the most copy number constraint refers to the fact that it is present in all genomes we examined, irrespective of copy number. We agree that the way it is written is a bit confusing, as NOTCH2NLA is not truly fixed because it does increase in copy number as a result of gene conversion of NOTCH2NLB, but it never decreases such that a haplotype is found with less than one copy. We adjusted the text as follows:

Edited:

"Aside from the NOTCH2-like IGC events, the metazoan conserved developmental ancestral gene, NOTCH2, is invariant with respect to copy number, however, so too is NOTCH2NLA, which is present in all sequenced human haplotypes but sometimes as more than one copy due to gene conversion."

Edited:

"The data also allows us to now address which copies of NOTCH2NL are present or absent, and to identify copy-specific directional biases with respect to gene conversion (e.g., NOTCH2NLB is only found converted to NOTCH2NLA but never vice versa)."

Regarding how we defined the boundaries of NOTCH2NL SD regulatory regions: because these complex regions have large segments of homology, it was necessary to use extensive surrounding sequence to capture the degree of rearrangement we observe in the element position. We agree the TAD boundaries are potentially more biologically relevant, so as mentioned above, we added a supplementary figure (Figure S11, see above) that reinterprets some aspects of our original analysis but is limited to those boundaries.

3. Null hypothesis for regulatory divergence between paralogs. The comparison of regulatory variation between paralogs to variation between alleles of a single locus is conceptually inappropriate. Alleles share genomic context and are nearly identical in sequence, while paralogs differ in both sequence and genomic environment; regulatory divergence is therefore expected. Demonstrating that paralogs differ from alleles does not test a meaningful null hypothesis, as such differences are expected. A more informative analysis would compare regulatory divergence between NOTCH2NL paralogs to divergence among other paralog pairs genome-wide. If the authors wish to argue for accelerated functional divergence within the NOTCH2NL family, this should be quantified relative to an appropriate background distribution.

We understand the reviewer's point and hope to eliminate any confusion regarding the point of the null hypothesis that we are trying to test regarding regulatory divergence. Our goal was to show that chromatin accessibility patterns between paralogs change more significantly than the allelic background divergence one would see between the maternal and paternal haplotypes (of chromosome 1). There have been no previous tests of the null hypothesis that chromatin between paralogs differ more than alleles, so we do not believe this was to be expected without formal testing. While such differences between paralogs may be expected, it is equally likely, in our opinion, that because so little time has passed (only a few million years) and the duplicated segments are so large (400-500 kbp) that the chromatin accessibility would remain essentially identical and this was the purpose of this null hypothesis. In addition, there are relatively few paralog pairs with the level of NOTCH2NL sequence identity. In fact, the degree of paralog sequence divergence (<1%) approaches allelic diversity for NOTCH2NLA and NOTCH2NLB, so we believe from a coalescent perspective it is comparable and appropriate.

In response to the referee's comment, however, we went through the text and removed any occurrences of functional acceleration. In addition, as part of this revision, we retested our null hypothesis using accessible elements that are contained within the TAD regions for each of the NOTCH2NL paralogs (new Figure S11B above). The goal of this analysis is to focus on the

most biologically relevant peak patterns as requested above by the reviewer. We also revised this paragraph of the text to introduce some of the caveats brought up by the referee as well as future work:
Edited:
"FiberFold predicts each NOTCH2NL paralog occupies a distinct 3D genomic environment despite their highly identical underlying sequence, with each NOTCH2NL TSS surrounded by a unique 3D landscape (Figure S9A). These paralog-specific 3D landscape patterns are largely consistent across haplotypes, with differences between paralogs more pronounced than differences between haplotypes (Figure S9B). Consistent with this, we also find that chromatin actuation differences between paralogs are more pronounced than differences between haplotypes (Figure S10), despite the high sequence identity between paralogs. Limiting this analysis to accessible chromatin elements that fall within the predicted TADs for each of the NOTCH2NL paralogs confirmed the above findings (Figure S11) and exposed a cluster of elements 63 kbp upstream of the NOTCH2, NOTCH2NLA, and NOTCH2NLB TSSs, which are selectively active on the NOTCH2NLA/B SDs but inactive on the NOTCH2 SD (Figure S9C). Overall, this analysis uncovers marked SD-specific accessible chromatin architectures and nominates specific elements that may be resulting in the paralog-specific regulation of the NOTCH2NL genes. However, further experiments will be needed to characterize the functional role of elements within TAD boundaries."

4. Figure clarity. The time-course figure is valuable, but the overall clarity of most figures did not improve despite suggestions, and remains suboptimal. Improving visual clarity would greatly enhance readability.

We spent the last few weeks revising/editing the figures to improve the clarity and readability–although confess that the many large-scale and small-scale structural changes among humans and other apes that have occurred make this task daunting. We simplified Figure 1A (below), produced a supplementary table (attached, Table S6) to aid with the definition of the duplicons presented as colored bars (Figure 1B), included a PDF in the supplementary materials of the phylogeny from Figure 4B (attached, Figure S13), and created a graphical abstract to help readers follow the evolution of the NOTCH2NL loci including changes in structure and chromatin accessibility (below).

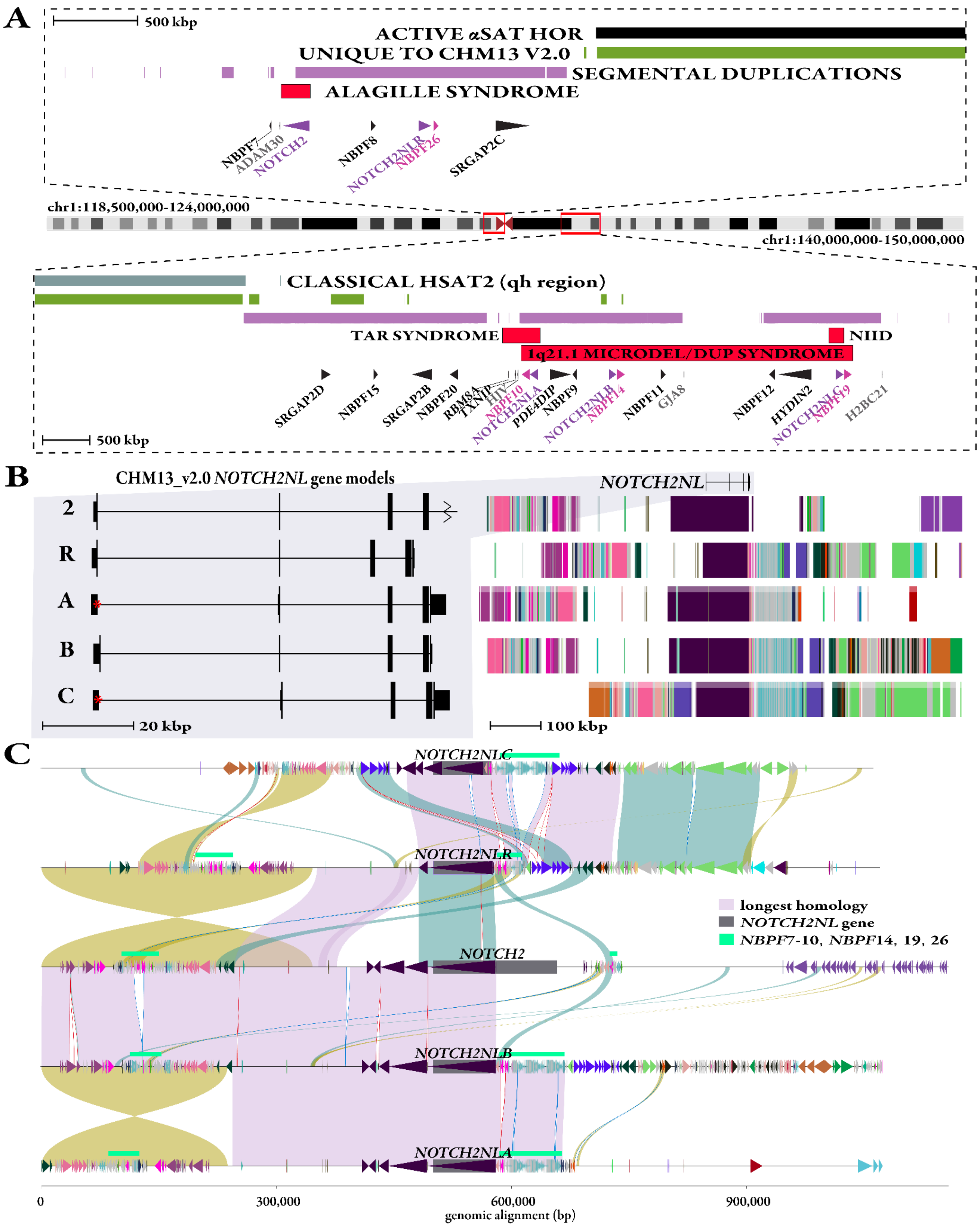

Updated Figure 1. Genome structure and organization of the NOTCH2NL gene family.

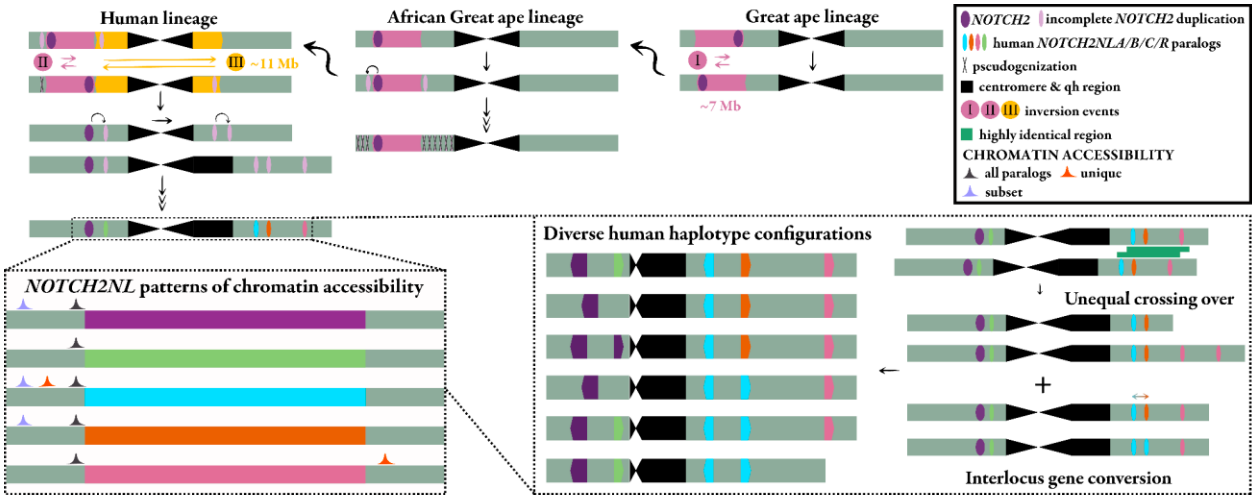

Graphical Abstract: Structural variation, evolution, and chromatin accessibility of NOTCH2NL loci. Schematic showing large-scale structural variation during ape evolution associated with the duplication of incomplete NOTCH2NL copies (top panel). Continued unequal crossover and interlocus gene conversion in humans (bottom right panel) creates a diverse set of haplotypes at different locations on chromosome 1 with distinct patterns of chromatin accessibility (bottom left panel) as potential sites for future investigation into the regulatory architecture of the NOTCH2NL gene family.

Minor points:

** Figure S9B: The labels on the x-axis appear incorrect for the first four entries, and the figure overall is difficult to interpret.*

We thank the reviewer for catching the labeling error and have edited Figure S9B to fix it. We have also edited Figure S9A to accommodate a comment below.

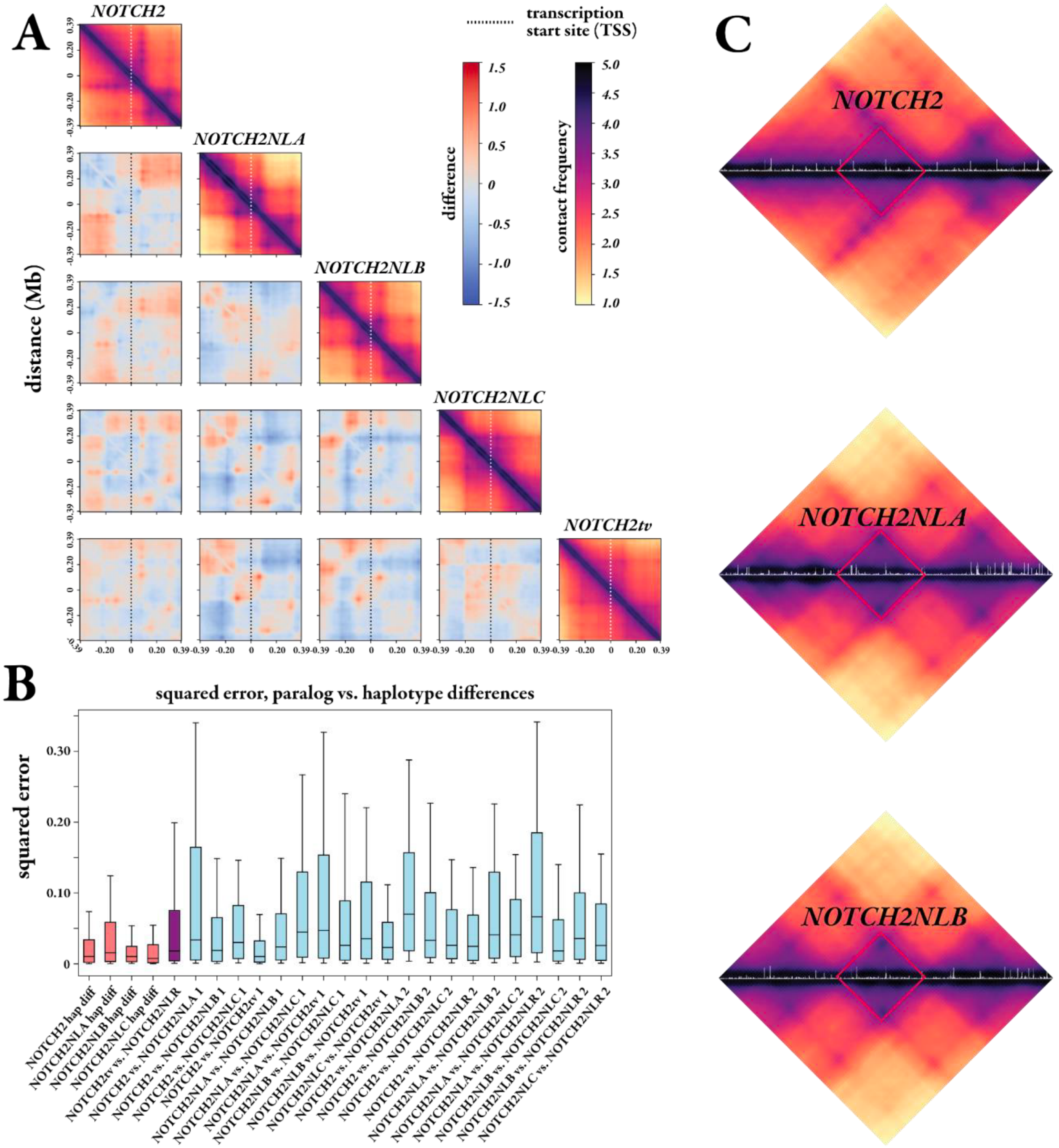

Figure S9. Predicted TADs and associated regulatory architectures in NOTCH2NL paralog regions.

** Figure S10: The distinction being shown is unclear (haplotypes?). If so, comparisons across paralogs should also be displayed.*

Related to our response in comment #3, the haplotype in Figure S10 (below) refers to the allelic background divergence of chromatin accessibility between maternal and paternal haplotypes. Each line is a pairwise comparison of all the paralogous elements from each paralog region conditioned on a specific paralog.

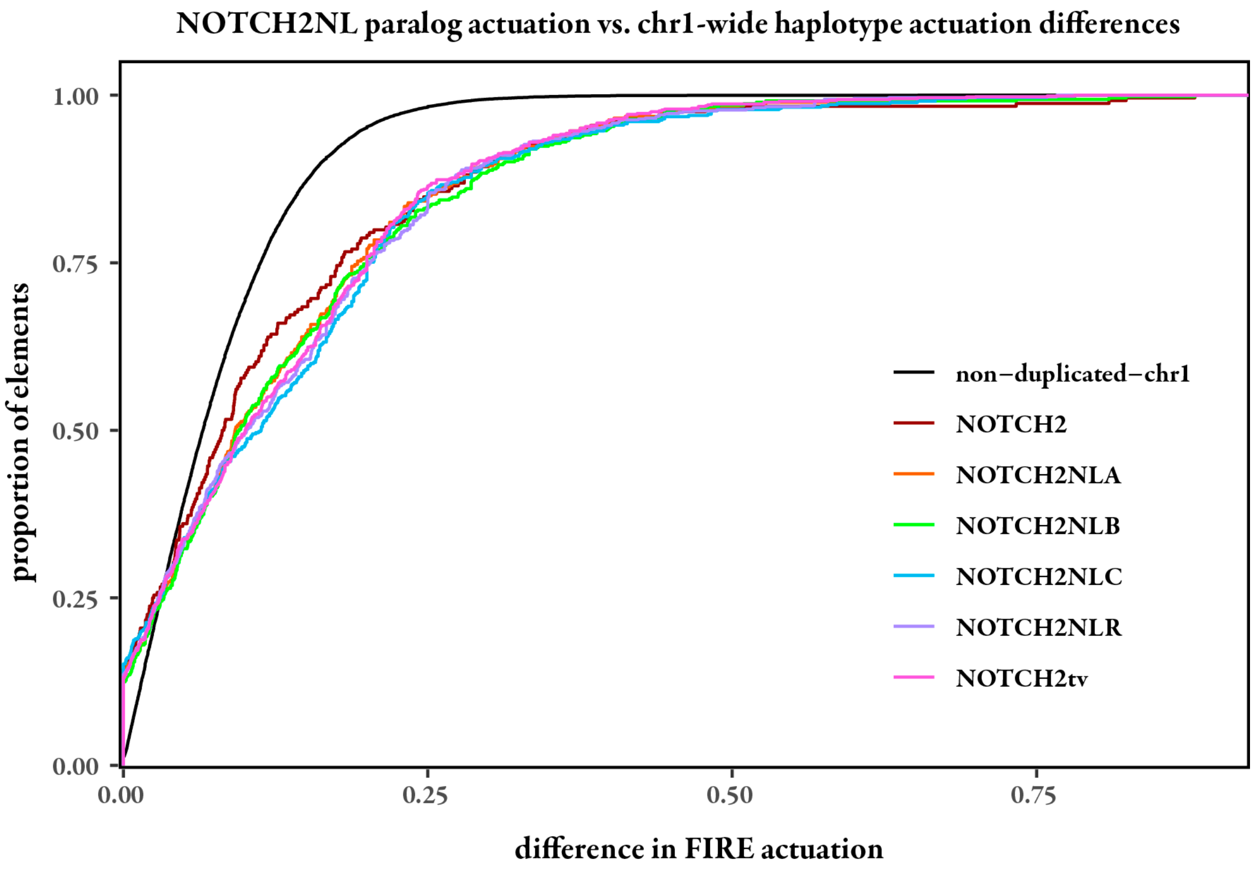

Figure S10. Comparison of NOTCH2NL paralog and haplotype actuation. The model of expected haplotype differences on chromosome 1 using brain organoid data mapped to T2TCHM13 (black) shows a higher proportion of regulatory elements with less actuation difference than what is seen for the different NOTCH2NL paralogs.

** Summary/abstract - "we show independent recurrent duplication among apes with proteincoding copies emerging in humans ~2.1 million years ago" - For consistency, I think this number should match the number in the introduction (~2).*

We changed both timepoints to reflect our most recent timing estimate in addition to matching the numbers.

Edited:

"Using near-complete long-read assemblies generated from 70 human and 12 ape haploid genomes, we show independent recurrent duplication among apes with protein-coding copies emerging in humans 2.2-3.7 million years ago."

Edited:

"Using comparative data from other great apes, we construct an evolutionary model for its origin and spread over the last 10 million years, providing evidence of recurrent duplication in different apes while showing that protein-coding copies began to emerge and spread 2.2-3.7 million years ago (MYA) in the human lineage."

** The manuscript introduces the human NOTCH2NL paralogs for the first time in the context of disease associations. A brief overview of the paralog set and current knowledge should precede discussion of phenotypic consequences.*

Based on the referee's suggestion, we introduced the members of the NOTCH2NL gene family earlier in the preceding paragraph.

Edited:

"Notch signaling, a mechanism of cell communication conserved throughout the metazoan

kingdom, is uniquely altered in humans due to a recent ape segmental duplication of the NOTCH2 gene (Fiddes et al. 2018, Suzuki et al. 2018). Segmental duplications (SDs) have both restructured primate genomes as well as led to the emergence of lineage-specific gene families resulting in potentially new functions, including distinct developmental fates (Yoo et al. 2025, Florio et al. 2018). In the case of NOTCH2NL (NOTCH2-N-terminus-like), the gene family is a ~70 kbp SD encompassing the four N-terminal exons of NOTCH2 and includes a unique final fifth exon exapted from the fourth intron of NOTCH2. While NOTCH2NL-like sequences exist in several primates, the NOTCH2NL protein appears to only be expressed in the developing human brain, suggesting it is functionally human specific (Fiddes et al. 2018). In humans, there are three truncated paralogs, NOTCH2NLA, NOTCH2NLB and NOTCH2NLC; all are expressed and the predicted proteins only differ by a few amino acids (Fiddes et al, 2018). Experimental work has shown that NOTCH2NL interacts with NOTCH2 and modulates the NOTCH2-signaling pathway (Fiddes et al. 2018, Suzuki et al. 2018). Specifically, NOTCH2NL increases the number of self-renewal divisions of progenitor radial glia while delaying differentiation of these cells into neurons. Prioritizing self-renewal over differentiation has been proposed to enable the human brain to increase neuronal mass during cortical neurogenesis (Fiddes et al. 2018, Suzuki et al. 2018). The functional impact of NOTCH2NL may be one explanation for the expansion of the human ancestral brain compared to our closest living relatives, the chimpanzees."

* *It is unclear how the authors infer that the NLB-to-NLA conversion occurred ~2.8 MYA, especially given that the authors also claim that these two paralogs diverged ~1.7 MYA (figure 3A) and that the last common ancestor of modern humans lived much later. (Results, Patterns of NOTCH2NL human genetic variation, end of 4th paragraph).*

We apologize for this confusion and have revised the text to make this clearer and consistent with the display:

Edited:

"In our analysis we never observe NOTCH2NLA converted to NOTCH2NLB identity *and, thus, the direction of IGC appears to be directional. We note that this event is almost exclusive to individuals of African descent* (p=0.0008, Fisher's exact test) suggesting it is an ancient *event. It is currently unclear whether this conversion event is recurrent or the product of a single ancestral event, but we do predict that NOTCH2NLA and NOTCH2NLB began to diverge ~1.7 MYA and that the duplication from NOTCH2NLC to NOTCH2NLA/B occurred even earlier (~3.0 MYA) (Figure 3B), long before modern humans emerged as a species."*

* *Figure S9A: The y-axis should likely read "distance (Mb).*

We thank the reviewer for catching this mistake and have edited Figure S9A to fix it, which can be seen above.

* *Point 7 (previous) - After editing, it is still unclear to me what we learn from this statement. What is the conclusion regarding the emergence and modification of the various NOTCH2NL paralogs? More specifically, the authors claim that NLC arose from a duplication independent of NLR (Fig. 3A). Therefore, how do the authors explain the observation that NLC shares the largest number of homologous alignments with NLR?*

While there is a general phylogenetic topology suggesting a temporal order to the duplications during human evolution, including an independent duplication of NOTCH2NLR and a NOTCH2NLC duplication preceding NOTCH2NLA and NOTCH2NLB, there are still inconsistencies with respect to the synteny that likely suggest subsequent rearrangements, including deletions during evolution. Given that we have not yet discussed the phylogeny at this point in the text, we opted to remove those sentences from the results to avoid confusion.

Edited:

"Importantly, all copies of NOTCH2NL show one breakpoint with respect to the ancestral NOTCH2 corresponding to the NBPF duplicon that demarcates the 3' end of each derivedduplicated

gene (Figure 1C). Additionally, the presumptive pseudogene NOTCH2NLR is missing the upstream nongenic ancestral NOTCH2 sequence present in NOTCH2NLA and NOTCH2NLB. NOTCH2NLC appears the most structurally divergent; most paralogous regions share the bulk of synteny upstream of the gene model, yet the opposite is true for NOTCH2NLC. Overall, NOTCH2NLC shares the largest number of homologous alignments with NOTCH2NLR."

** Point 8 (previous) - the response remains unclear. In addition, the text in the response does not match the text in the manuscript ("are" should be singular).*

We tested whether the mean length of the NOTCH2NL SDs in humans were significantly different than the mean lengths observed in the other great apes so either significantly larger or smaller (two-sided t-test). We revised the text to make this clearer, including changing the statement of significance to singular:

Edited:

"We find that the mean SD length between NOTCH2NL SDs in humans (311 kbp) and other NHAs (215) kbp is not significantly different (p=0.42; t-test two-sided) indicating that this locus was unstable and began to duplicate in the common ancestor of the great apes. Nevertheless, NHA SDs do not share extensive synteny with humans when considering sequence flanking the human NOTCH2NL paralogs (Figure S3B) due to either independent events or subsequent extensive rearrangement."

Reviewer #3: The authors have addressed our comments. We congratulate the authors on a great manuscript!

We thank the reviewer for their time and effort in rechecking our manuscript.

Referees' reports, third round of review

Reviewer #2: This study addresses an important question in evolutionary biology and applies cutting-edge methodologies to shed light on it. Some of the points I raised could have been addressed with deeper engagement with the underlying conceptual issues, for example the comparison between regulatory divergence of paralogs that split millions of years ago and alleles that diverged only thousands of years ago. In addition, the presentation could still benefit from further clarification. Nonetheless, these issues do not, in my view, detract substantially from the overall contribution. I therefore recommend acceptance and congratulate the authors on successfully tackling such a challenging genomic locus.

Authors' response to the third round of review

Reviewer #2: This study addresses an important question in evolutionary biology and applies cutting-edge methodologies to shed light on it. Some of the points I raised could have been addressed with deeper engagement with the underlying conceptual issues, for example the comparison between regulatory divergence of paralogs that split millions of years ago and alleles that diverged only thousands of years ago. In addition, the presentation could still benefit from further clarification. Nonetheless, these issues do not, in my view, detract substantially from the overall contribution. I therefore recommend acceptance and congratulate the authors on successfully tackling such a challenging genomic locus.

We thank the reviewer for all their previous revisions, especially those that helped develop and strengthen the regulatory section of this manuscript. As a final addition to acknowledge that there underlying conceptual issues in our study of paralog regulatory divergence, we added an additional sentence to the Gene regulation and Fiber-seq subsection of the Limitations section of the manuscript:

Edited to add:
"Gene regulation and Fiber-seq. Fiber-seq is a measure of chromatin accessibility, which is only a proxy for noncoding regulatory regions; it does not distinguish between element types, such as repressors and enhancers, nor address which genes these elements would be regulating. Further work is needed to resolve how paralog-specific regulatory architectures diverge relative to SD sequence identity. Although FiberFold will generate predictions of which genes may be regulated by these accessible chromatin peaks, further efforts are needed to functionally characterize these predictions."
